# National, regional, and global statistics on alcohol consumption and associated burden of disease 2000–20: a modelling study and comparative risk assessment

**DOI:** 10.1016/S2468-2667(25)00174-4

**Published:** 2025-08-27

**Authors:** Kevin Shield, Ari Franklin, Ashley Wettlaufer, Ivneet Sohi, Meera Bhulabhai, Elizabeth K Farkouh, Ilinca-Gabriela Radu, Iman Kassam, Mikayla Munnery, Ronaz Remtulla, Sarah Richter, Farhana Safa, Sara Tasnim, Abhinav Thakral, Mavra Qamar, Jürgen Rehm

**Affiliations:** a Institute for Mental Health Policy Research, Centre for Addiction and Mental Health, Toronto, ON, Canada; b Dalla Lana School of Public Health, University of Toronto, Toronto, ON, Canada; c Epidemiology and Biostatistics, Schulich School of Medicine and Dentistry, Western University, London, ON, Canada; d Mayo Clinic Alix School of Medicine, Rochester, MN, USA; e Department of Psychiatry, University of Toronto, Toronto, ON, Canada; f Institute of Medical Science, University of Toronto, Toronto, ON, Canada; g Centre for Interdisciplinary Addiction Research, University Medical Center Hamburg-Eppendorf, Hamburg, Germany; h Program on Substance Abuse & WHO European Region Collaboration Centre, Public Health Agency of Catalonia, Barcelona, Spain

## Abstract

**Background:**

Data on alcohol consumption and associated health harms are essential to evaluate progress in achieving global health goals. This study aims to estimate global alcohol consumption from 2000 to 2020, and the global burden of alcohol-attributable harms from 2000 to 2019.

**Methods:**

In this global analysis, adult per capita consumption data estimates were modelled on the basis of sales, survey, and traveller data. Drinking status and past 30-day heavy episodic drinking were estimated through regression analyses of 540 surveys from 174 countries. Alcohol-attributable harms were estimated using a comparative risk assessment methodology by combining alcohol consumption data with corresponding relative risks obtained from meta-analyses and cohort studies. Mortality and morbidity data were obtained from WHO Global Health Estimates.

**Findings:**

Globally, average alcohol consumption in 2019 among adults was 5·5 L (95% uncertainty interval 4·9–6·2), which increased from 5·1 L (4·6–5·7) in 2000. From 2019 to 2020 alcohol consumption decreased to 4·9 L (4·3–5·6). In 2019, alcohol consumption was associated with 2·6 (2·3–3·1) million deaths (4·7% of all deaths) and 116·0 million disability-adjusted life-years (DALYs) lost (4·6% of all DALYs lost). In contrast to alcohol consumption, the number of alcohol-attributable deaths decreased by 31·0% and DALYs lost per 100 000 people decreased by 27·4% from 2000 to 2019.

**Interpretation:**

Alcohol is attributed to a large burden of disease, which disproportionately affects people in Eastern Europe and in Central and Southern Sub-Saharan Africa, and young people. Accordingly, these regions should implement policies such as alcohol taxation increases, availability reductions, and marketing restrictions to reduce alcohol-related harms.

**Funding:**

WHO.

## Introduction

Alcohol consumption has created, and continues to create, substantial health, social, and economic burdens, representing a barrier to achieving the Sustainable Development Goals globally.^1^ Although the negative effects of alcohol on health, social wellbeing, and economies have been well established and documented, alcohol remains the most widely consumed psychoactive substance of substantial public health importance.^2^ High consumption persists despite the inclusion of alcohol reduction as a key target within the Noncommunicable Disease Global Monitoring Framework and the Sustainable Development Goals,^3^ as well as the availability of numerous effective policies to reduce alcohol-attributable harms.^4^

To prioritise the implementation of evidence-based alcohol control policies, including treatment strategies, it is important to understand which countries and regions experience relatively high levels of alcohol consumption and are disproportionately affected by its consequences. This study aimed to provide a regional and global overview of alcohol consumption as a risk factor for burden of disease from 2000 to 2020, by country, sex, and age. The consumption estimates encompass all relevant dimensions necessary to model the alcohol-related burden of disease, including: alcohol adult per capita consumption (APC)—measured in litres of pure alcohol consumed per adult (≥15 years) annually; drinking status (ie, prevalence of past-year drinkers, former drinkers, and lifetime abstainers); and the prevalence of heavy episodic drinking (HED), defined in this study as consuming 60 g or more of ethanol on at least one occasion in the past 30 days. We also aimed to examine the changes in APC from 2019 to 2020 to examine the effect of the COVID-19 pandemic and associated policies on APC. Furthermore, the study estimated the alcohol-attributable burden of disease from 2000 to 2019.

**Research in context**

**Evidence before this study**

A literature search for publications on the burden of disease attributable to alcohol published in English between Jan 1, 2000, and Sept 17, 2024, was conducted on the Global Health data exchange, UNICEF Multiple Indicator Cluster Surveys, Demographic and Health surveys, WHO Multi-Country Studies Data Archive, The Health Navigator, International Household Survey Network, PubMed, Google, and Google Scholar using the term list: (“alcohol” OR “health”) AND “study” AND “country name” AND “year”. We also manually searched the WHO Global Health Observatory and the Institute for Health Metrics and Evaluation research article database. Alcohol is a leading contributor to the global burden of disease. Previous estimates of the alcohol-attributable burden of disease enabled cross-country comparisons of alcohol consumption and its harms using data on alcohol sales, the prevalence of drinking and abstention, and self-reports of consumption, combined with corresponding relative risk functions. These previous studies observed large differences in the levels of alcohol consumed and resulting alcohol-attributable harms between countries, sexes, and age groups.

**Added value of this study**

This study confirms that alcohol is still a leading risk factor for communicable diseases in addition to non-communicable diseases and injuries, and that most countries will not meet the WHO Global Alcohol Action Plan 2022–30 goal of reducing alcohol consumption by 20% unless additional policies are implemented. Geographical and temporal differences in alcohol consumption and the impact of alcohol were observed in this study. In particular, alcohol-attributable harms were high in Eastern Europe and, despite a relatively low level of alcohol consumption, the alcohol-attributable burden of disease in Sub-Saharan Africa was also observed to be high. Furthermore, despite decreases in alcohol-attributable mortality in all other regions, the alcohol-attributable burden of disease increased in the South Asia region, due to increases in both consumption and alcohol-attributable harms in India.

**Implications of all the available evidence**

Variations in estimates of alcohol consumption and the alcohol-attributable burden of disease indicate that a large proportion of these burdens is preventable. Although policies affecting consumption have been implemented in some countries (eg, China, Lithuania, and Russia), such policies, in particular high-impact policies, remain underdeveloped in many countries. Accordingly, efforts to implement high-impact policies must be intensified.

## Methods

### Indicators of alcohol consumption

A deterministic modelling approach was employed to estimate global alcohol consumption and the resulting health burdens. This study adheres to the Guidelines for Accurate and Transparent Health Estimates Reporting statement (appendix 1 pp 6–7). The analyses of alcohol consumption estimates were preregistered with PROSPERO (CRD42020186514). The analyses of alcohol-attributable harms were not preregistered. As the study was a secondary analysis of publicly available data, ethics approval and consent were not required.

To characterise alcohol consumption, we used APC, drinking status, and HED. The definitions of drinking status and HED were based on those outlined by WHO.^2^ Data on alcohol consumption were estimated for 194 countries (appendix 1 pp 9–15). APC was calculated as the sum of recorded consumption (alcohol consumption captured through taxation and sales databases) and unrecorded consumption (alcohol consumption not captured though taxation or sales databases, eg, homemade or informally produced alcohol, smuggled alcohol, and surrogate alcohol [ie, substances that contain ethanol but are not produced for human consumption, such as mouthwash and perfumes]) and corrected for the consumption of both inbound and outbound travellers for each country (appendix 1 pp 9–15).^5^ Presented APC values from 2000 to 2019 were 3-year moving averages. APC estimates for 2020 were estimated based on the calendar year. Data on drinking status and HED were estimated using a modelling approach. The modelled estimates were based on survey data collected through a systematic review. The electronic databases MEDLINE and Embase were searched with publication dates restricted to between Jan 1, 2010, and Aug 6, 2019. As we do not know the global effect of the COVID-19 pandemic and associated social distancing policies, or the effect of policy changes regarding alcohol sales (ie, the expansion of alcohol sales using online stores, and the temporary closing of alcohol stores due to social distancing policies^6^) on either drinking status or the prevalence of HED, we did not report drinking status and HED for 2020. The web search engines Google and Google Scholar were searched using the search terms “alcohol population survey [country] [year]”, where the subsequent country or year combination was searched when no relevant results were obtained after 50 consecutive results. The international databases Global Health Data Exchange, UNICEF Multiple Indicator Cluster Surveys, Demographic and Health Surveys, International Household Survey Network, WHO Multi-Country Studies Data Archive, and International Labour Organization Microdata Repository, and the national statistical services and ministry of health websites for all countries were searched. In addition, reference lists of all relevant reports were reviewed. The results from both published survey reports and individual-level survey datasets were combined with data from a previous systematic review done by Manthey and colleagues.^7^ From published reports, data were extracted from 560 surveys. The combined systematic review found sex-stratified and age-stratified aggregate estimates of drinking status or HED for 179 countries (appendix 1 pp 16–51).

To model drinking status, a Dirichlet regression was used. To model HED among past-year drinkers, a fractional response regression was used. In both models, APC, gross domestic product based on purchasing power parity (time-varying across all years), Global Burden of Disease (GBD) regions, Muslim population size (time-varying across all years), and Muslim-majority countries with alcohol prohibitions were entered as covariates. The prevalence of past-year abstainers (obtained from the Dirichlet regression) was also used to predict the prevalence of HED. Regression models for drinking status and HED also included covariates for sex (male or female) and age (15–19, 20–24, 25–34, 35–49, 50–64, and ≥65 years). Interaction terms in both models were included to account for the hypothesised interaction between age and sex. To account for differences in the timeframe used to define current and former drinking (with the past year as the reference category) and HED (with the past month or 28 days as the reference category), covariates were used in the regression models. A covariate for the threshold used to define HED (≥60 g per occasion as the reference category) was also used.

To model alcohol consumption among current drinkers, APC was separated by age and sex (appendix 1 p 52). Average daily alcohol consumption among current drinkers was modelled using a gamma distribution in accordance with the methodology outlined by Kehoe and colleagues, whereby the distribution of alcohol consumption is predicted on the basis of the mean consumption.^8^ A correction factor of 0·8 was applied to APC data when modelling the alcohol-attributable burden of disease to account for alcohol that was not consumed and the under-reporting of alcohol consumption in observation studies from which the relative risks (RRs) were obtained. The exact level of alcohol not consumed and under-reporting of alcohol in observation studies are currently unknown, but 0·8 is in line with the undercoverage observed in a systematic review by Stockwell and colleagues of alcohol consumption in cohort studies compared with APC estimates.^9^

### Alcohol-attributable burden of disease

The alcohol-attributable burden of disease for 2000 to 2019 was modelled using a Levin-based population-attributable fraction method based on the theoretical minimum risk exposure level of lifetime abstention from alcohol (appendix 1 p 54). Both the detrimental and protective health effects of alcohol were modelled. In estimating the alcohol-attributable burden of disease, no lag time between alcohol consumption and outcome was assumed except for cancer, where a 10-year lag between consumption and outcomes was modelled.

The inclusion of diseases was based on a causal association of alcohol, which was assessed by the WHO Technical Advisory Group on Alcohol and Drug Epidemiology. RR estimates were obtained from meta-analyses and cohort studies (appendix 1 pp 60–94).

### Mortality, morbidity, and population data

Data on mortality, years of life lost (YLL), and morbidity (years lived with disability [YLD] and disability-adjusted life-years [DALYs] lost) were obtained from the WHO Global Health Estimates;^10^ data were available by year (2000–19), country, age, and sex, as well as by cause of mortality and morbidity.

Population data by country, age, sex, and year were obtained from the UN Population Division (2022 revisions).^11^ Age-standardised rates were estimated on the basis of the WHO standard population.^12^ To match age-standardisation data, deaths, YLL, YLD, and DALYs lost were aggregated into 5-year age groups, from 0 years to 84 years, with a final category of 85 years and older. The alcohol population-attributable fractions were applied to mortality and morbidity age groupings, which were encompassed within the alcohol population-attributable fraction age groupings.

Data were aggregated by GBD regions and according to the 2019 Human Development Index (HDI) categories (appendix 1 pp 95–97).^13^, ^14^

### Estimates of uncertainty

Estimates of uncertainty (ie, 95% uncertainty intervals) were constructed using 1000 simulated estimates generated through a Monte Carlo-type approach. Each simulated estimate of alcohol consumption was derived from its respective underlying uncertainty distribution. Simulated estimates of the alcohol-attributable burden of disease were based on simulated alcohol consumption estimates, combined with corresponding simulated RR functions. Because the WHO Global Health Estimates and UN population estimates do not include measures of uncertainty, the uncertainty in the burden of disease estimates and population data are not reflected in the 95% uncertainty intervals. To construct the 95% uncertainty intervals, the 2·5th and 97·5th percentiles of the 1000 simulations were used. The number of simulations was chosen based on a simulation study that examined the stability of 95% uncertainty intervals constructed using 1000 simulations.^15^

### Role of the funding source

The funder of the study had no role in study design, data collection, data analysis, interpretation of data, writing of the report, or the decision to submit the paper for publication.

## Results

**Figure 1:**
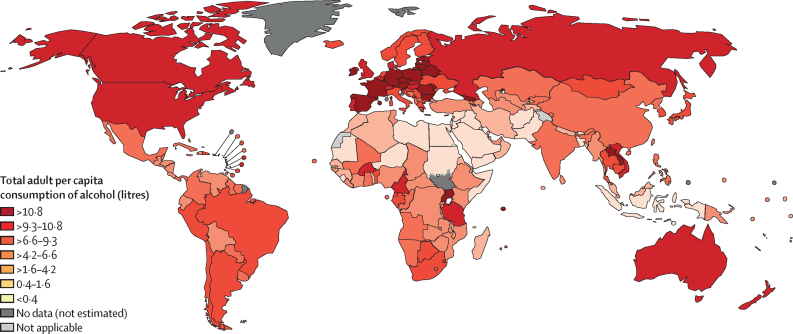
Per capita consumption of alcohol among adults in 2019, by country

Globally, APC was 5·5 L (95% uncertainty interval 4·9–6·2) in 2019, with an estimated 89·9% of APC coming from recorded sources (4·3 L [3·8–4·8]) and 11·1% coming from unrecorded sources (1·2 L [0·8–1·6]). The highest levels of alcohol consumption were observed in Central Europe (11·7 L [10·3–13·2]), Eastern Europe (10·1 L [7·7–12·5]), and Australasia (10·1 L [7·6–12·5]); alcohol consumption was lowest in North Africa and the Middle East (0·6 L [0·4–1·0]), Oceania (1·8 L [0·9–3·0]), and Central Sub-Saharan Africa (3·4 L [2·3–4·6]; figure 1, appendix 1 p 103). At the country level, APC was highest in Romania (17·0 L [12·6–21·4]), followed by Georgia (14·3 L [9·9–18·8]) and Czechia (13·3 L [10·1–16·6]). APC also showed a clear gradient by HDI grouping, ranging from 2·9 L (2·5–3·5) for low-HDI countries to 8·7 L (8·0–9·5) for high-HDI countries (appendix 2 sheets 1–2).

**Figure 2:**
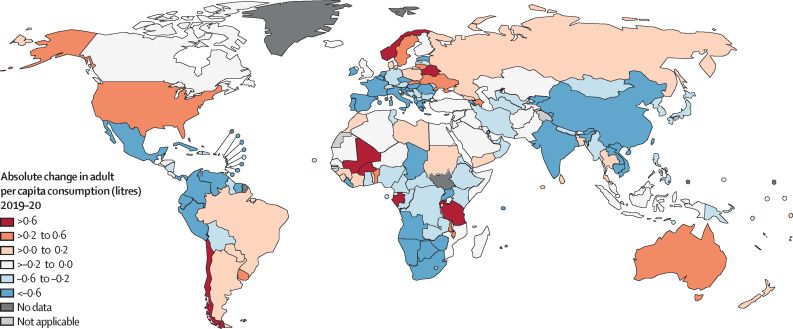
Change in per capita consumption of alcohol among adults from 2019 to 2020, by country

Globally, from 2000 to 2019, APC increased by 17·4%, from 5·1 L (95% uncertainty interval 4·6–5·7) to 5·5 L (4·9–6·2). At the regional level, the largest increases were observed for South Asia (149·1% increase; 1·6 L [0·4–2·9] to 3·9 L [2·1–5·8]), East Asia (57·1% increase; 3·6 L [1·9–5·5] to 5·7 L [3·5–7·9]), and Southeast Asia (46·2% increase; 2·6 L [2·1–3·3] to 3·9 L [3·2–4·7]), whereas Central Sub-Saharan Africa (51·6% decrease; 7·1 L [5·0–9·2] to 3·4 L [2·3–4·6]), Western Sub-Saharan Africa (26·8% decrease; 5·8 L [4·4–7·2] to 4·2 L [3·2–5·3]), and Eastern Europe (21·3% decrease; 12·9 L [10·2–15·5] to 10·1 L [7·7–12·5]) had the largest decreases in APC. In 2020, the COVID-19 pandemic had a marked effect on APC, with alcohol consumption decreasing by 11·1% since 2019 to 4·9 L (4·3–5·6). However, from 2019 to 2020, 23 (12%) of 189 countries had increases in APC of 0·1 L or greater, 116 (61%) countries had a decrease in APC of 0·1 L or greater, and 50 (26%) countries had a change in APC of less than 0·1 L (figure 2; appendix 1 pp 99, 103; appendix 2 sheets 1–2).

Globally, in 2019, most people did not consume alcohol in the past year (current drinking 43·8% [95% uncertainty interval 42·8–44·8]) or engage in HED (16·7% [14·5–18·9]; appendix 1 pp 103–04, appendix 2 sheets 4–5). The prevalence of current drinking was higher among male (52·2% [51·3–53·2]) compared with female (35·4% [34·3–36·5]) individuals. Similarly, the prevalence of HED was higher among male (23·5% [20·4–26·6]) compared with female (9·7% [8·4–10·9]) individuals. The prevalence of current drinkers and HED in the adult population varied widely across the globe. The highest prevalence of current drinking was in Western Europe (77·4% [77·0–77·8]), high-income North America (70·9% [69·7–72·2]), and Central Europe (70·7% [70·1–71·3]), whereas the lowest prevalence of current drinkers was in North Africa and the Middle East (6·2% [5·9–6·4]), Central Asia (21·4% [20·3–22·5]), and South Asia (25·5% [23·4–27·6]). The highest prevalence of HED was in high-income Asia–Pacific (43·2% [42·1–44·4]), Australasia (39·5% [37·0–42·1]), and Central Sub-Saharan Africa (36·7% [35·4–38·1]), whereas the lowest prevalence of HED was in North Africa and the Middle East (1·3% [1·2–1·4]), Central Asia (7·9% [7·6–8·1]), and South Asia (9·9% [9·0–10·8]). The prevalence of current drinking and HED also showed a clear association with HDI groupings, ranging from a prevalence of 19·8% (19·3–20·3) for current drinkers and 9·9% (9·7–10·1) for HED in low-HDI countries to a prevalence of 64·0% (63·6–64·4) for current drinkers and 29·4% (28·9–29·9) for HED in very high-HDI countries (appendix 1 pp 100, 102, 104).

**Figure 3:**
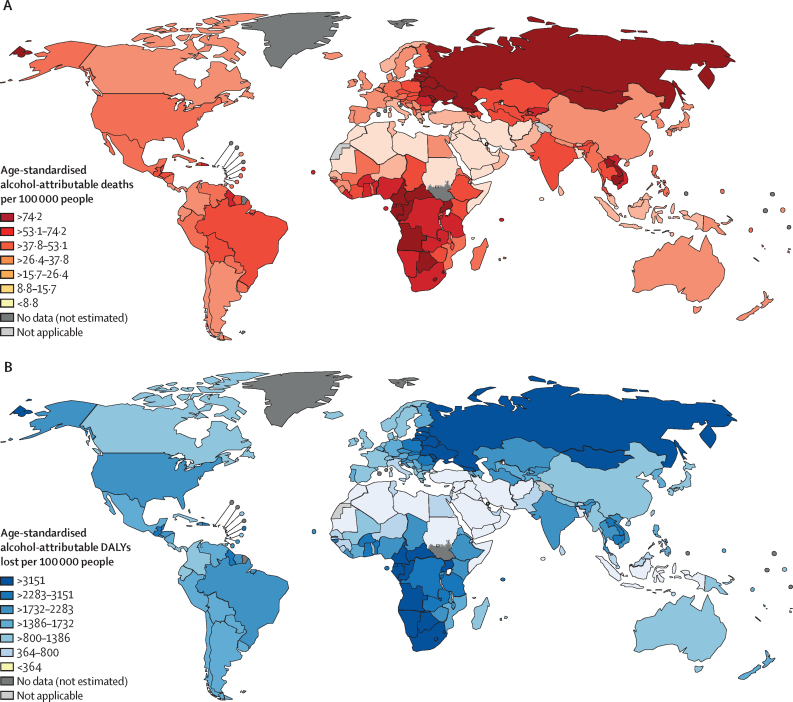
Age-standardised alcohol-attributable deaths per 100 000 people (A) and population attributable fraction for the proportion of deaths attributable to alcohol consumption (B) in 2019, by country DALY=disability-adjusted life-year.

Alcohol consumption resulted in an estimated 2·6 (95% uncertainty interval 2·3–3·1) million deaths (4·7% [4·1–5·6] of all deaths) and 116·0 million DALYs lost (4·6% [4·1–5·3] of all DALYs lost) in 2019 (table, figure 3). Deconstructed, 89·6 (78·0–105·3) million DALYs lost were attributed to premature mortality (ie, YLL), representing 5·3% (4·6–6·2) of all YLL, and 26·4 (24·1–29·8) million DALYs lost were attributed to morbidity (ie, YLD), representing 3·2% (2·9–3·6) of all YLD (appendix 1 pp 110–12). Male individuals had a larger number of deaths attributable to alcohol (2·0 [1·7–2·4] million, representing 6·7% [5·6–7·9] of all deaths) compared with female individuals (0·6 [0·5–0·8] million, representing 2·4% [1·8–3·2] of all deaths). Similarly, male individuals had a larger number of DALYs lost attributable to alcohol (92·7 [79·6–108·0] million DALYs lost, representing 6·9% [5·9–8·0] of all DALYs lost) compared with female individuals (23·2 [20·6–28·4] million DALYs lost, representing 2·0% [1·8–2·4] of all DALYs lost; appendix 1 pp 105–06).

**Table:** Alcohol-attributable burden of disease in 2019, by cause

	Alcohol-attributable burden*		Age-adjusted alcohol-attributable burden per 100 000 people*		Population-attributable fraction (%)*	
	Deaths (1000s)	DALYs lost (100 000s)	Deaths	DALYs lost	Deaths	DALYs lost
All causes	2624·4 (2288·1 to 3072·8)	1158·9 (1023·8 to 1346·3)	32·3 (28·2 to 37·8)	1474·8 (1304·1 to 1715·6)	4·7 (4·1 to 5·6)	4·6 (4·1 to 5·3)
Communicable, maternal, perinatal, and nutritional conditions	284·1 (130·4 to 447·5)	118·3 (50·5 to 195·3)	3·5 (1·6 to 5·6)	152·6 (64·5 to 252·7)	2·8 (1·3 to 4·4)	1·7 (0·7 to 2·9)
Tuberculosis	190·0 (50·0 to 351·9)	92·0 (25·2 to 169·0)	2·4 (0·6 to 4·5)	119·6 (32·9 to 219·8)	15·8 (4·2 to 29·2)	14·0 (3·8 to 25·7)
Sexually transmitted diseases excluding HIV	0·1 (0·0 to 0·1)	0·2 (0·1 to 0·3)	0·0 (0·0 to 0·0)	0·3 (0·2 to 0·4)	0·1 (0·1 to 0·2)	0·4 (0·3 to 0·6)
HIV/AIDS	15·4 (10·2 to 21·0)	8·4 (5·6 to 11·4)	0·2 (0·1 to 0·3)	10·9 (7·3 to 14·9)	2·3 (1·5 to 3·2)	2·1 (1·4 to 2·9)
Lower respiratory infections	78·5 (32·2 to 125·8)	17·7 (7·9 to 27·5)	0·9 (0·4 to 1·5)	21·7 (9·7 to 33·7)	3·0 (1·2 to 4·9)	1·7 (0·8 to 2·6)
Non-communicable diseases	1616·8 (1371·3 to 1905·2)	604·7 (549·4 to 659·9)	19·6 (16·7 to 22·9)	757·9 (690·3 to 825·4)	4·0 (3·4 to 4·7)	3·8 (3·5 to 4·2)
All malignant neoplasms	400·9 (350·2 to 449·3)	106·6 (92·8 to 119·3)	4·8 (4·2 to 5·4)	130·0 (113·1 to 145·6)	4·3 (3·8 to 4·8)	4·4 (3·8 to 4·9)
Lip and oral cavity malignant neoplasms	52·8 (42·8 to 61·9)	15·5 (12·4 to 18·2)	0·6 (0·5 to 0·7)	19·0 (15·2 to 22·4)	27·5 (22·3 to 32·3)	27·5 (22·1 to 32·4)
Other pharynx malignant neoplasms	42·4 (33·6 to 49·6)	12·4 (9·7 to 14·5)	0·5 (0·4 to 0·6)	15·2 (11·9 to 17·8)	34·8 (27·6 to 40·8)	35·3 (27·7 to 41·3)
Oesophageal cancer	83·0 (64·3 to 100·2)	21·3 (16·6 to 25·6)	1·0 (0·8 to 1·2)	25·8 (20·1 to 31·0)	17·9 (13·9 to 21·7)	18·6 (14·5 to 22·3)
Colon and rectum cancers	105·9 (87·7 to 126·2)	25·0 (20·6 to 30·1)	1·3 (1·0 to 1·5)	30·3 (25·0 to 36·4)	11·6 (9·6 to 13·8)	11·6 (9·5 to 13·9)
Liver cancer	64·2 (43·3 to 93·4)	16·4 (10·9 to 23·9)	0·8 (0·5 to 1·1)	20·1 (13·4 to 29·2)	11·1 (7·5 to 16·2)	10·5 (7·0 to 15·2)
Breast cancer	27·6 (20·5 to 35·5)	8·8 (6·5 to 11·3)	0·3 (0·2 to 0·4)	10·9 (8·1 to 14·0)	4·3 (3·2 to 5·6)	4·5 (3·3 to 5·7)
Cervix uteri cancer	1·4 (0·8 to 2·4)	0·5 (0·3 to 0·9)	0·0 (0·0 to 0·0)	0·7 (0·4 to 1·2)	0·5 (0·3 to 0·8)	0·5 (0·3 to 0·9)
Larynx cancer	23·7 (17·9 to 29·6)	6·6 (4·9 to 8·2)	0·3 (0·2 to 0·4)	8·0 (6·0 to 9·9)	21·0 (15·9 to 26·3)	21·5 (16·0 to 26·7)
Diabetes	−5·2 (−23·2 to 16·3)	−4·4 (−13·9 to 6·9)	−0·1 (−0·3 to 0·2)	−5·3 (−16·9 to 8·5)	−0·3 (−1·6 to 1·1)	−0·6 (−2·0 to 1·0)
Alcohol use disorders	156·5 (156·5 to 156·5)	193·1 (193·1 to 193·1)	2·0 (2·0 to 2·0)	248·6 (248·6 to 248·6)	100·0 (100·0 to 100·0)	100·0 (100·0 to 100·0)
Epilepsy	13·2 (10·1 to 16·2)	11·9 (9·2 to 14·5)	0·2 (0·1 to 0·2)	15·4 (11·9 to 18·8)	11·4 (8·7 to 14·0)	9·1 (7·1 to 11·1)
All cardiovascular diseases	473·6 (252·9 to 743·4)	103·0 (61·7 to 149·0)	5·6 (3·0 to 8·6)	125·1 (75·4 to 179·2)	2·7 (1·4 to 4·2)	2·7 (1·6 to 3·8)
Hypertensive heart disease	57·1 (41·0 to 71·9)	11·9 (8·7 to 14·8)	0·7 (0·5 to 0·8)	14·3 (10·4 to 17·8)	5·0 (3·6 to 6·3)	5·4 (3·9 to 6·8)
Ischaemic heart disease	208·3 (−6·4 to 422·2)	31·7 (−3·8 to 66·1)	2·4 (−0·1 to 4·8)	37·1 (−4·9 to 77·6)	2·3 (−0·1 to 4·8)	1·8 (−0·2 to 3·7)
Ischaemic stroke	−65·5 (−144·6 to 40·8)	−14·7 (−27·2 to 3·0)	−0·7 (−1·6 to 0·5)	−17·5 (−31·9 to 2·9)	−2·1 (−4·7 to 1·3)	−2·4 (−4·5 to 0·5)
Haemorrhagic stroke	244·7 (172·9 to 330·4)	64·6 (46·1 to 86·3)	2·9 (2·1 to 3·9)	79·1 (56·5 to 105·5)	7·8 (5·5 to 10·6)	8·2 (5·9 to 11·0)
Alcoholic cardiomyopathy	29·0 (29·0 to 29·0)	9·6 (9·6 to 9·6)	0·4 (0·4 to 0·4)	12·0 (12·0 to 12·0)	100·0 (100·0 to 100·0)	100·0 (100·0 to 100·0)
All digestive diseases	578·0 (490·8 to 660·3)	194·4 (165·1 to 219·6)	7·1 (6·0 to 8·1)	244·1 (207·2 to 275·6)	23·6 (20·0 to 26·9)	22·2 (18·9 to 25·1)
Cirrhosis of the liver†	550·3 (465·7 to 632·6)	184·6 (155·9 to 210·3)	6·8 (5·7 to 7·8)	231·7 (195·6 to 263·9)	41·9 (35·5 to 48·2)	43·2 (36·5 to 49·2)
Pancreatitis	27·7 (20·2 to 36·3)	9·8 (7·1 to 12·8)	0·3 (0·2 to 0·4)	12·4 (9·0 to 16·2)	25·5 (18·6 to 33·4)	27·9 (20·3 to 36·4)
Injuries	723·5 (563·0 to 948·6)	435·9 (342·8 to 571·6)	9·2 (7·2 to 12·1)	564·3 (444·2 to 739·5)	16·4 (12·8 to 21·6)	16·8 (13·2 to 22·0)
Unintentional injuries	520·7 (401·6 to 709·7)	330·8 (257·1 to 443·6)	6·6 (5·1 to 9·0)	426·6 (331·2 to 572·0)	16·5 (12·7 to 22·5)	17·3 (13·4 to 23·2)
Road injury	297·5 (198·1 to 454·0)	187·5 (125·1 to 284·6)	3·8 (2·5 to 5·8)	244·0 (162·6 to 370·4)	23·3 (15·5 to 35·5)	23·8 (15·9 to 36·1)
Poisonings	10·8 (7·9 to 14·6)	5·1 (3·7 to 6·9)	0·1 (0·1 to 0·2)	6·6 (4·8 to 8·9)	12·8 (9·4 to 17·3)	10·8 (7·8 to 14·4)
Falls	75·1 (49·1 to 110·0)	55·3 (38·2 to 76·1)	0·9 (0·6 to 1·3)	69·4 (47·9 to 95·4)	11·0 (7·2 to 16·1)	14·5 (10·0 to 19·9)
Fire, heat, and hot substances	12·4 (9·4 to 16·2)	8·9 (6·4 to 11·9)	0·2 (0·1 to 0·2)	11·4 (8·3 to 15·3)	10·9 (8·2 to 14·3)	10·9 (7·9 to 14·6)
Drowning	27·2 (18·0 to 38·1)	12·3 (8·1 to 17·3)	0·3 (0·2 to 0·5)	16·1 (10·6 to 22·6)	11·6 (7·6 to 16·2)	9·2 (6·1 to 12·9)
Exposure to mechanical forces	15·8 (9·9 to 22·8)	19·2 (12·8 to 26·5)	0·2 (0·1 to 0·3)	24·6 (16·4 to 33·9)	13·0 (8·2 to 18·8)	16·0 (10·7 to 22·1)
Other unintentional injuries	82·0 (55·1 to 113·2)	42·5 (28·6 to 58·1)	1·0 (0·7 to 1·4)	54·5 (36·7 to 74·6)	13·0 (8·7 to 18·0)	12·1 (8·2 to 16·6)
Intentional injuries	202·8 (93·9 to 310·9)	105·1 (47·7 to 161·2)	2·6 (1·2 to 4·0)	137·8 (62·3 to 211·5)	16·3 (7·5 to 24·9)	15·3 (6·9 to 23·4)
Self-harm	123·2 (62·7 to 184·1)	56·8 (28·8 to 84·2)	1·6 (0·8 to 2·4)	74·0 (37·5 to 109·6)	17·5 (8·9 to 26·2)	17·8 (9·1 to 26·5)
Interpersonal violence	79·6 (32·4 to 126·9)	48·3 (19·8 to 76·9)	1·0 (0·4 to 1·7)	63·8 (26·0 to 101·7)	16·8 (6·8 to 26·8)	16·0 (6·5 to 25·5)

Data are median (95% uncertainty interval). DALY=disability-adjusted life-year.

* Negative values represent deaths and DALYs avoided due to alcohol consumption (ie, negative numbers indicate there would be an increase in the number of deaths or DALYs lost under the counterfactual scenario of everyone being a lifetime abstainer).

† Includes ICD-10 codes K70 (alcoholic liver disease) and K74 (fibrosis and cirrhosis of the liver).

Globally, alcohol consumption was a large contributor to the burden of communicable, maternal, perinatal, and nutritional conditions, with 0·3 (95% uncertainty interval 0·1–0·4) million alcohol-attributable deaths (2·8% [1·3–4·4] of total) and 11·8 (5·1–19·5) million alcohol-attributable DALYs lost (1·7% [0·7–2·9] of total); to the burden of non-communicable diseases, alcohol contributed 1·6 (1·4–1·9) million deaths (4·0% [3·4–4·7] of total) and 60·5 (54·9–66·0) million DALYs lost (3·8% [3·5–4·2] of total); and to the burden of injuries, alcohol contributed 0·7 (0·6–0·9) million deaths (16·4% [12·8–21·6] of total) and 43·6 million (34·3–57·2) DALYs lost (16·8% [13·2–22·0] of total; table).

**Figure 4:**
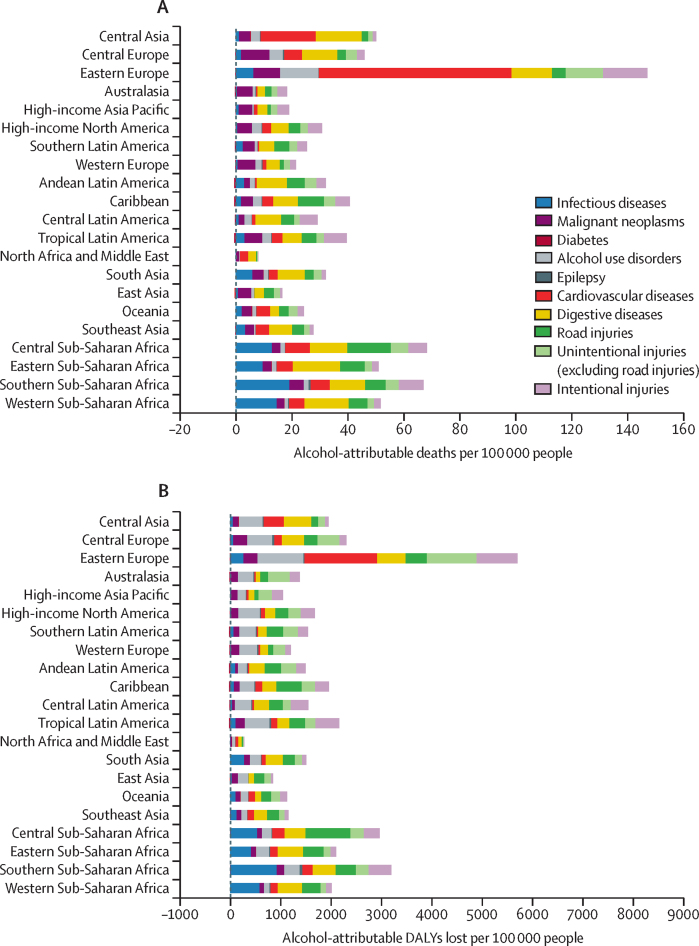
Age-standardised rates of alcohol-attributable deaths and DALYs lost, by cause and region DALY=disability-adjusted life-year.

At the regional level, the alcohol-attributable age-standardised death and DALYs lost rates were highest in the Eastern Europe and Central and Southern Sub-Saharan Africa regions, and lowest in the North Africa and Middle East region (figure 4). At the country level, the alcohol-attributable age-standardised death rates were highest in Belarus (164·3 [147·5–179·4] per 100 000), Moldova (162·3 [142·2–181·3] per 100 000), and Ukraine (156·9 [135·4–173·8] per 100 000). The numbers of alcohol-attributable age-standardised DALYs lost were highest in Russia (5772·4 [5153·6–6196·3] per 100 000), Ukraine (5667·9 [4961·9–6170·6] per 100 000), and Belarus (5596·2 [5146·3–5972·4] per 100 000; appendix 2 sheets 5–6).

In 2000, 2·5 (95% uncertainty interval 2·2–2·9) million deaths and 113·2 (101·8–130·5) million DALYs lost globally were attributable to alcohol, representing 4·9% (4·3–5·7) of all deaths and 4·2% (3·8–4·9) of all DALYs lost globally. From 2000 to 2019, the alcohol-attributable age-standardised rates decreased by 31·0% for deaths (from 46·8 [41·9–55·1] to 32·3 [28·2–37·8] deaths per 100 000 people) and by 27·4% for DALYs lost (from 2030·8 [1832·0–2330·3] to 1474·8 [1304·1–1715·6] DALYs lost per 100 000 people); these decreases were similar in magnitude to the relative decreases in the age-standardised rates of all deaths of 29·8% (944·5 to 663·3 deaths per 100 000 people) and all DALYs lost of 29·2% (45 083·5 to 31 910·6 DALYs per 100 000 people).^10^ From 2000 to 2019, alcohol-attributable cardiovascular deaths (48·9% decrease, from 10·9 [7·7–15·6] to 5·6 [3·0–8·6] deaths per 100 000 people) and DALYs lost (49·0% decrease, from 245·1 [190·9–322·9] to 125·1 [75·4–179·2] DALYs lost per 100 000 people) proportionally changed more than any other alcohol-related disease or injury. At the regional level, the largest decreases in alcohol-attributable deaths per 100 000 people were observed for the Eastern European (46·3% decrease, from 268·6 [251·3–284·8] to 147·3 [133·5–158·3] deaths per 100 000 people), Central Sub-Saharan Africa (45·2% decrease, from 120·5 [93·5–144·8] to 68·5 [54·0–89·9] deaths per 100 000 people), and high-income Asia–Pacific (43·2% decrease, from 35·6 [30·3–41·5] to 19·1 [15·9–22·4] deaths per 100 000 people) regions. Unlike other regions, which all saw a decrease, the South Asia region had a 23·2% increase in alcohol-attributable deaths per 100 000 people (from 26·3 [18·4–42·0] to 32·4 [23·4–41·6] deaths per 100 000 people), which was primarily due to an increase in the deaths attributable to alcohol consumption per 100 000 people in India (26·3% increase, from 30·5 [20·5–49·4] to 38·5 [27·2–49·5] deaths per 100 000 people). This contrasts with a 33·2% decrease in all-cause mortality in the South Asia region (from 1251·1 to 835·3 deaths per 100 000 people) and a 35·1% decrease in all-cause mortality in India (from 1268·7 to 823·4 deaths per 100 000 people) during the same time period (appendix 2 sheets 5–6).

As a sensitivity analysis, we examined the effect of using a 1-year estimate of APC for 2019 when compared with 2020. A similar estimate was observed for the change in APC from 2019 to 2020 when APC in 2019 was based on a 1-year estimate, with alcohol consumption decreasing by 7·6% from 5·3 L (4·7–6·0) in 2019 to 4·9 L (4·3–5·6) in 2020.

## Discussion

This study provides a comprehensive overview of the changing landscape in both alcohol consumption and the associated health harms. Globally, most adults do not consume alcohol, and most people who do consume alcohol do not engage in HED; however, current levels of alcohol consumption and HED still cause substantial health harms globally. The results of this study also highlight substantial sex, geographical, and temporal differences in alcohol consumption and the attributable burden of disease.

Notable decreases in alcohol consumption were observed in Eastern Europe and Central Sub-Saharan Africa. The factors driving changes in alcohol consumption in Central Sub-Saharan Africa are not well documented and warrant further investigation. In Eastern Europe, the decline has largely been attributed to alcohol policies, such as restrictions on marketing and availability, increased excise taxes, minimum pricing for vodka, and efforts to reduce unrecorded consumption in Russia since 2004.^16^ Policies in Lithuania and Estonia, including regulations on availability, advertising, taxation, and drink-driving, have also contributed to reduced consumption and alcohol-related harms.^17^

Despite decreases in consumption, the alcohol-attributable burden in 2019 was highest in Eastern Europe, Central Sub-Saharan Africa, and Southern Sub-Saharan Africa. In Eastern Europe, the burden is largely driven by HED, with prolonged periods of heavy alcohol intake and negative effects on infectious diseases, cardiovascular diseases, and injuries.^18^ In Central and Southern Sub-Saharan Africa, although alcohol consumption is relatively low, the high prevalence of infectious diseases and liver cirrhosis amplifies the burden of alcohol.^10^

At the country level, stronger alcohol-related policies have been associated with lower rates of chronic liver disease, hepatocellular carcinoma, other neoplasms, and cardiovascular disease.^19^ These policies include the WHO “best buys” of increases in taxation, restrictions in availability, and restrictions in marketing, and WHO SAFER, which includes the “best buys” of screening and brief interventions for harmful use of alcohol and the enforcement of drink-driving laws.^4^ However, unlike other psychoactive substances that exert substantial effects on global health, alcohol is not regulated at the international level by legally binding regulatory instruments.

In the present study, we observed a strong gradient in alcohol consumption, where consumption was lowest in low-HDI regions and highest in high-HDI regions. Using these data, we hypothesise that as countries develop there will be an increase in alcohol consumption in most countries, and the harms caused by the increase in alcohol consumption will, in part, offset some of the health gains from development (how development will affect alcohol consumption in Muslim-majority countries is unknown). Of note is the large increase in alcohol consumption and alcohol-related harms in countries such as India and Viet Nam, with alcohol-attributable mortality rates increasing despite a decrease in overall mortality rates.

Although not discussed in this paper, alcohol disproportionately affects certain populations, including adolescents (who are often targeted by alcohol advertisements^20^ and face higher risks of injury and brain development issues^21^), young women (who have rising alcohol-related liver disease and alcohol use disorders^22^), and people of lower socioeconomic status.^23^ Accordingly, alcohol policies—such as restricting advertisements,^20^ zoning laws for retail establishments,^24^ and taxation^25^—should be prioritised to achieve health equity for these groups.

The alcohol consumption and alcohol-attributable burden of disease estimates presented in this study are limited by multiple factors. Drinking status and HED estimates are based on surveys. Accordingly, we expect the prevalence of HED to be underestimated owing to populations excluded due to the design of the survey, participation bias, and social desirability bias.^26^ The measure of HED is limited by the binary categorisation of yes and no, which does not consider variations in intensity or frequency. This is especially relevant for Eastern European countries, where people engage in dynamic drinking patterns of continuous alcohol intake (markedly exceeding 60 g of alcohol per day) over several days or weeks.^27^

The APC data from 2000 to 2019 are 3-year averages. Thus, the data might hide yearly fluctuations in alcohol consumption. The choice of a correction factor of 0·8 applied to APC data, although in line with a systematic review by Stockwell and colleagues,^9^ could have an effect on our findings, although whether this effect would cause an underestimation or overestimation is unknown. More research is needed here to determine what the appropriate correction factor should be. Moreover, the burden of disease estimates presented in this study are limited by current knowledge of the causal relationship between alcohol consumption and the development of disease. Future causality assessments could lead to additions or exclusions of diseases and injuries attributable to alcohol consumption.

The RR estimates used in this study were selected by the WHO Technical Advisory Group on the basis of majority consensus. This approach might bias the selection of the RRs. To reduce the random error introduced by expert decisions, ideally, a Delphi study or other systematic methods to deal with decision making under uncertainty can assist in making important judgements.^28^ The RR estimates used were obtained from studies that used lifetime abstention or abstainers as the reference category. People choose to be lifetime abstainers from alcohol consumption for various potentially confounding reasons, including religion and health.^29^ The use of abstainers as the reference category is also problematic as abstainers often include people who are so-called sick quitters, ie, people who have stopped drinking for health reasons. Therefore, this use of reference category might lead to an underestimation of the risk of disease among drinkers.^30^

The WHO's Global Health Estimates combine data for both type 1 and type 2 diabetes. Type 2 diabetes accounts for the majority of diabetes cases globally.^31^ The present study modelled the effect of alcohol use on diabetes by applying an RR function for type 2 diabetes to an aggregated category, which includes both type 1 and type 2 diabetes.^32^ Although alcohol consumption is a known causal risk factor for the development of type 2 diabetes,^32^ less is known about its effects on type 1 diabetes. Alcohol can affect glucose metabolism and could have adverse effects in individuals with type 1 diabetes, however, it remains unclear whether its effect on type 1 diabetes mirrors that observed for type 2 diabetes.^33^ As such, the estimated health effect of alcohol on diabetes in this study might represent an overestimate.

The presented estimates are not separated by race or ethnicity. This limits the results of our study as people with the aldehyde dehydrogenase 2*2 allele (prevalent in East Asian populations) have a higher risk of upper aerodigestive tract cancers.^32^ Furthermore, racialised minorities in some countries are disproportionately affected by alcohol use compared with people from other ethnicities.^34^

The uncertainty intervals are likely to underestimate the true error, as error estimates for global health estimates of mortality, morbidity, and population data were unavailable and not considered. Additionally, the consumption and burden estimates do not account for systematic errors (eg, those introduced by human judgement in selecting regression models, covariates, and RRs^35^) that could affect the overall uncertainty.

Finally, when comparing our estimates with those of the Global Burden of Diseases, Injuries, and Risk Factors Study (GBD) 2021,^36^ the following points deserve attention. The estimate of 2·6 million deaths attributable to alcohol presented in this study is different from that presented in the Institute for Health Metrics and Evaluation (IHME) GBD Study, which estimates that 1·8 million deaths in 2019 were attributable to high alcohol consumption (appendix 1 pp 105–06).^36^ Estimates of alcohol-attributable infectious diseases were appreciably different between studies (284 100 in this study compared with the IHME estimate of 136 800). This is in large part due to GBD estimates not including alcohol-attributable deaths from lower respiratory infections (78 500 deaths in this study), sexually transmitted diseases excluding HIV (100 deaths in this study), and HIV/AIDS (15 400 deaths in this study). Additionally, our study estimated 723 500 alcohol-attributable injury deaths and the IHME GBD study estimated 187 900 alcohol-attributable injury deaths. In both cases, the largest category of alcohol-attributable injuries was from road injuries, with 297 500 alcohol-attributable deaths in the present study and 45 400 alcohol-attributable deaths estimated by IHME. Injury registry data from Brazil (10 900 alcohol-attributable deaths in 2021), China (48 800 alcohol-attributable deaths; yearly average from 2001 to 2016), and the USA (10 100 alcohol-attributable deaths in 2019) suggest that the burden estimated by IHME is underestimated.^37^, ^38^, ^39^ In addition, IHME GBD basing injury outcomes on the average level of drinking is potentially problematic, as injuries are strongly linked to HED.^32^

Our RR approach to model the burden of injuries attributable to alcohol consumption also has limitations. Policy and contextual factors, such as drink-driving laws and enforcement, affect the burden of alcohol-related road injuries, but these factors are not accounted for in the RR model.^40^ Accordingly, implementing a global injury registration (eg, road injuries or falls) system is essential to better assess the effect of alcohol on injury outcomes.

In conclusion, although at the global level there has been a reduction in health harms, alcohol consumption has not decreased, indicating that the reduction in harms is likely to be driven by a decrease in the underlying risk of diseases, conditions, and injuries causally related to alcohol. Accordingly, there remains a need for policies to reduce the disease burden attributable to alcohol, including but not limited to regulating alcohol at the international level by legally binding regulatory instruments, taxation, reductions in availability, and restrictions in marketing.

### Contributors

### Data sharing

Data that underlie the results reported in this article (text, tables, figures, and appendices), as well as the study protocol, statistical analysis plan, and analytic code, will be made available upon request following publication, with no end date, to anyone who wishes to access the data for any purpose. Proposals should be directed to the corresponding author to gain access.

## Declaration of interests

We declare no competing interests.
