# Supplementary material for: National, regional, and global statistics on alcohol consumption and associated burden of disease 2000–20: a modelling study and comparative risk assessment
Source: Lancet Public Health. 2025 Aug 27;10(9):e751–61. doi: 10.1016/S2468-2667(25)00174-4 (PMC12394792; doi:10.1016/S2468-2667(25)00174-4)
Supplement: Supplementary appendix 1 [file mmc1.pdf]

THE LANCET
Public Health

Supplementary appendix

This appendix formed part of the original submission and has been peer reviewed. We post it as supplied by the authors.

Supplement to: Shield K, Franklin A, Wettlaufer A, et al. National, regional, and global statistics on alcohol consumption and associated burden of disease 2000–20: a modelling study and comparative risk assessment. *Lancet Public Health* 2025; **10:** e751–61.

SUPPLEMENT

National, Regional, and Global Statistics on Alcohol Consumption and the Resulting Burdens of Disease 2000-2020: A Comparative Risk Assessment Study

Table of Contents

Table S1. Sources of alcohol per capita consumption (APC) data by WHO Member State 9
Table S2. Step-wise search in Ovid (MEDLINE and Embase) 16
Table S3. Intergovernmental organizations and global health databases searched in the systematic review 16
Table S4. Inclusion and exclusion criteria 17
Table S5. Surveys included in the modelling of drinking statuses and heavy episodic drinking 19
Table S6. Causes and sources of relative risks and causality.. 60
Table S7. List of countries by global burden of disease region 95
Table S8. List of countries by human development index grouping.. 97
Table S9. Alcohol consumption in 2019, by region 103
Table S10. Alcohol consumption by sex in 2019 104
Table S11. Alcohol-attributable burden of disease in 2019, by cause for males... 105
Table S12. Alcohol-attributable burden of disease in 2019, by cause for females 106
Table S13. Burden of disease in the present study as compared to the IHME global burden of disease study...118

Figure S1. PRISMA flow diagram for the systematic search of surveys of population surveys on drinking status 18
Figure S2. Relative risk for tuberculosis among males 61
Figure S3. Relative risk for tuberculosis among females 61
Figure S4. Relative risk for sexually transmitted diseases among males 62
Figure S5. Relative risk for sexually transmitted diseases among females 62
Figure S6. Relative risk for lower respiratory infections among males 63
Figure S7. Relative risk for lower respiratory infections among females 63
Figure S8. Relative risk for mouth and oropharynx cancers among males 64
Figure S9. Relative risk for mouth and oropharynx cancers among females 64
Figure S10. Relative risk for mouth and oesophagus cancer among males 65
Figure S11. Relative risk for mouth and oesophagus cancer among females.. 65
Figure S12. Relative risk for colon and rectal cancer among males.. 66
Figure S13. Relative risk for colon and rectal cancer among females 66
Figure S14. Relative risk for liver cancer among males.. 67
Figure S15. Relative risk for liver cancer among females.. 67
Figure S16. Relative risk for breast cancer among females 68
Figure S17. Relative risk for larynx cancer among males.. 69
Figure S18. Relative risk for larynx cancer among females.. 69
Figure S19. Relative risk for diabetes mellitus among males.. 70
Figure S20. Relative risk for diabetes mellitus among females 70

Figure S21. Relative risk for epilepsy among males ... 71
Figure S22. Relative risk for epilepsy among females ... 71
Figure S23. Relative risk for hypertensive heart disease among males ... 72
Figure S24. Relative risk for hypertensive heart disease among females ... 72
Figure S25. Relative risk for ischaemic heart disease among males 15 to 34 years of age ... 73
Figure S26. Relative risk for ischaemic heart disease among females 15 to 34 years of age ... 73
Figure S27. Relative risk for ischaemic heart disease among males 35 to 64 years of age ... 74
Figure S28. Relative risk for ischaemic heart disease among females 35 to 64 years of age ... 74
Figure S29. Relative risk for ischaemic heart disease among males 65 years of age and older ... 75
Figure S30. Relative risk for ischaemic heart disease among females 65 years of age and older ... 75
Figure S31. Relative risk for ischaemic stroke among males 15 to 34 years of age ... 76
Figure S32. Relative risk for ischaemic stroke among females 15 to 34 years of age ... 76
Figure S33. Relative risk for ischaemic stroke among males 35 to 64 years of age ... 77
Figure S34. Relative risk for ischaemic stroke among females 35 to 64 years of age ... 77
Figure S35. Relative risk for ischaemic stroke among males 65 years of age and older ... 78
Figure S36. Relative risk for ischaemic stroke among females 65 years of age and older ... 78
Figure S37. Relative risk for haemorrhagic stroke among males ... 79
Figure S38. Relative risk for haemorrhagic stroke among females ... 79
Figure S39. Relative risk for cirrhosis of the liver among males ... 80
Figure S40. Relative risk for cirrhosis of the liver among females ... 80
Figure S41. Relative risk for pancreatitis among males ... 81
Figure S42. Relative risk for pancreatitis among females ... 81
Figure S43. Relative risk for road injury among males and females ... 82
Figure S44. Relative risk for other unintentional injuries among males and females ... 82
Figure S45. Relative risk for intentional injuries among males and females ... 83
Figure S46. Relative risk for tuberculosis among males for Russia ... 84
Figure S47. Relative risk for tuberculosis among females for Russia ... 84
Figure S48. Relative risk for lower respiratory infections among males for Russia ... 85
Figure S49. Relative risk for lower respiratory infections among females for Russia ... 85
Figure S50. Relative risk for ischaemic stroke among males for Russia ... 86
Figure S51. Relative risk for ischaemic stroke among females for Russia ... 86
Figure S52. Relative risk for ischaemic heart disease among males for Russia ... 87
Figure S53. Relative risk for ischaemic heart disease among females for Russia ... 87
Figure S54. Relative risk for haemorrhagic stroke among males for Russia ... 88
Figure S55. Relative risk for haemorrhagic stroke among females for Russia ... 88
Figure S56. Relative risk for pancreatitis among males for Russia ... 89

Figure S57. Relative risk for pancreatitis among females for Russia 89
Figure S58. Relative risk for cirrhosis of the liver among males for Russia 90
Figure S59. Relative risk for cirrhosis of the liver among females for Russia 90
Figure S60. Relative risk for road injury among males for Russia 91
Figure S61. Relative risk for road injury among females for Russia 91
Figure S62. Relative risk for other unintentional injuries among males for Russia 92
Figure S63. Relative risk for other unintentional injuries among females for Russia 92
Figure S64. Relative risk for suicide among males 93
Figure S65. Relative risk for suicide among females 93
Figure S66. Relative risk for assault among males 94
Figure S67. Relative risk for assault among females 94
Figure S68. Recorded adult per capita consumption of alcohol in 2019 99
Figure S69. Unrecorded adult per capita consumption of alcohol in 2019 99
Figure S70. Change in *per capita* consumption of alcohol among adults from 2000 to 2020 100
Figure S71. Prevalence of current drinkers among adults in 2019 by country 100
Figure S72. Prevalence of heavy episodic drinking in 2019, by country 101
Figure S73. Adult per capita consumption of alcohol in 2019 among drinkers by country 102
Figure S74. Percent of all deaths attributable to alcohol consumption by age 107
Figure S75. Alcohol-attributable deaths per 100 000 people, and the percent of all deaths attributable to alcohol consumption by country in 2019 108
Figure S76. Alcohol-attributable years of life lost per 100 000 people, and the percent of all years of life lost attributable to alcohol consumption by country in 2019 109
Figure S77. Alcohol-attributable years lived with disability per 100 000 people, and the percent of all years lived with disability attributable to alcohol consumption by country in 2019 110
Figure S78. Alcohol-attributable disability adjusted years of life lost per 100 000 people, and the percent of all disability adjusted years of life lost attributable to alcohol consumption by country in 2019 111
Figure S79. Alcohol-attributable years of life lost (YLL) and years with disability (YLD) per 100 000 people in 2019 by region and cause 112
Figure S80. Alcohol-attributable deaths and disability adjusted years of life lost (DALYs) per 100 000 people in 2019 by human development index group and cause 113
Figure S81. Alcohol-attributable years of life lost (YLL) and years with disability (YLD) per 100 000 people in 2019 by human development index group and cause 114
Figure S82. Alcohol-attributable deaths per 100 000 people in 2019 by human development index score 115
Figure S83. Alcohol-attributable years of life lost (YLL) per 100 000 people in 2019 by human development index score 115

Figure S84. Alcohol-attributable years with disability (YLD) per 100 000 people in 2019 by human development index score 116
Figure S85. Alcohol-attributable disability adjusted years of life lost (DALYs) per 100 000 people in 2019 by human development index score 116
Figure S86. Alcohol-attributable deaths and disability adjusted years of life lost (DALYs) per 100 000 people by year and cause..117

GATHER checklist

Item #	Checklist item	Place Reported
Objectives and funding		
1	Define the indicator(s), populations (including age, sex, and geographic entities), and time period(s) for which estimates were made.	Methods
2	List the funding sources for the work.	Abstract
Data Inputs		
For all data inputs from multiple sources that are synthesized as part of the study:		
3	Describe how the data were identified and how the data were accessed.	Methods section
4	Specify the inclusion and exclusion criteria. Identify all ad-hoc exclusions.	Supplement – methods section
5	Provide information on all included data sources and their main characteristics. For each data source used, report reference information or contact name/institution, population represented, data collection method, year(s) of data collection, sex and age range, diagnostic criteria or measurement method, and sample size, as relevant.	Methods section<br><br>Supplement – methods section
6	Identify and describe any categories of input data that have potentially important biases (e.g., based on characteristics listed in item 5).	Discussion – limitations section
For data inputs that contribute to the analysis but were not synthesized as part of the study:		
7	Describe and provide sources for any other data inputs.	Methods section<br><br>Supplement – methods
For all data inputs:		
8	Provide all data inputs in a file format from which data can be efficiently extracted (e.g., a spreadsheet rather than a PDF), including all relevant meta-data listed in item 5. For any data inputs that cannot be shared because of ethical or legal reasons, such as third-party ownership, provide a contact name or the name of the institution that retains the right to the data.	Supplement – methods section
Data analysis		
9	Provide a conceptual overview of the data analysis method. A diagram may be helpful.	Methods section<br><br>Supplement – methods section (diagram not used)
10	Provide a detailed description of all steps of the analysis, including mathematical formulae. This description should cover, as relevant, data cleaning, data pre-processing, data adjustments and weighting of data sources, and mathematical or statistical model(s).	Methods section
11	Describe how candidate models were evaluated and how the final model(s) was/were selected.	Methods
12	Provide the results of an evaluation of model performance, if done, as well as the results of any relevant sensitivity analysis.	Discussion – Comparison with the 2021 Global Burden of Disease study results for 2019
13	Describe the methods used for calculating the uncertainty of the estimates. State which sources of uncertainty were, and were not, accounted for in the uncertainty analysis.	Methods
14	State how to access the analytic or statistical source code used to generate the estimates.	Supplement – methods section
Results and Discussion		
15	Provide published estimates in a file format from which data can be efficiently extracted.	Supplementary excel files
16	Report a quantitative measure of the uncertainty of the estimates (e.g., uncertainty intervals).	Results, Supplement, and supplementary excel files

17	Interpret results in light of existing evidence. If updating a previous set of estimates, describe the reasons for changes in the estimates.	Discussion – comparison with the 2021 Global Burden of Disease study results for 2019
18	Discuss limitations of the estimates. Include a discussion of any modelling assumptions or data limitations that affect interpretation of the estimates.	Discussion – limitations section

SUPPLEMENTAL METHODS

Statistical analysis code and input files

All statistical code (i.e., R code) and input files used to produce the results presented in this paper are available to the general public. To obtain the code and input files, please contact the corresponding author, Kevin Shield Ph.D. (Kevin.Shield@camh.ca).

Data on adult per capita consumption

Data on recorded APC were obtained from multiple data sources, with a clear algorithm of preference, [11] including data from government statistics, GlobalData, IWSR-International Wine and Spirit Research, Wine Institute (historically World Drink Trends), International Organisation of Vine and Wine (OIV) and the statistical database of the Food and Agriculture Organization of the United Nations (FAOSTAT). To estimate unrecorded APC data, a modelling approach was taken utilizing data from representative empirical surveys (e.g., STEPS survey) and periodic surveys of the opinions of experts at the country level using a modified Delphi-technique (nominal group technique).[12] In cases where no data were available, unrecorded APC was estimated using a fractional response random intercepts regression model (urbanization, migration rates, malnutrition, sanitation, education levels, and *per capita* gross domestic product adjusted for purchasing power parity (GDP-PPP) were used as predictors).[13]

Travellers' APC estimates were based on United Nations statistics,[14] and data were provided by the Institute for Health Metrics and Evaluation. For 2020, traveller consumption for each of Antigua and Barbuda, Bahamas, Barbados, Belize, Cook Islands, Dominica, Grenada, Kiribati, Maldives, Seychelles, St Kitts and Nevis, St Lucia, St Vincent and the Grenadines, and Vanuatu was estimated by comparing the decrease in recorded APC to the decrease in tourist visits to the country in 2020 (as obtained from the World Tourism Organization (UNWTO)).[14] The decrease in recorded alcohol consumption was attributed entirely to a decrease in forgiven traveller visits.

Table S1. Sources of alcohol per capita consumption (APC) data by WHO Member State

Country	Data source recorded
Afghanistan	1961-1999: FAO
	2000-2010: merged (FAO, OIV)
	2010-2020: merged (FAO, OIV, IWSR)
Albania	1962-1999: FAO;
	2000-2020: merged (GlobalData, IWSR, OIV)
Algeria	1961-1999: WDT
	2000-2020: merged (FAO, GlobalData, OIV)
Andorra	2000-2020: Average of France and Spain
Angola	1961-1999: FAO
	2000-2020: merged (GlobalData, IWSR, OIV)
Antigua and Barbuda	1962-1999: FAO
	2000-2020: merged (FAO, GlobalData, IWSR, OIV)
Argentina	1961-1979: FAO
	1980-1999: WDT
	2000-2010: WHO Global Surveys on Alcohol and Health
	2011-2020: Instituto Nacional de Estadistica y Censos Republica Argentina [National Institute of Statistics and Census of Argentina]
Armenia	1990-1999: FAO
	2000-2020: merged (GlobalData, IWSR, OIV)
Australia	1961-1989: World Drink Trends (WDT)
	1990-1999: National Drug Research Institute (NDRI)
	2000-2019: Australian Bureau of Statistics
Austria	1960-1962: FAO
	1963-1999: WDT
	2000-2017: Handbook on Alcohol (Anton Proksch Institute)
	2019: WHO Global Survey on Alcohol and Health
Azerbaijan	1990-1999: FAO
	2000-2008: merged (GlobalData, IWSR, OIV)
	2009-2020: Statistical Yearbook
Bahamas	1961-1999: FAO
	2000-2018: merged (GlobalData, IWSR)
Bahrain	1970-1999: FAO
	2000-2020: merged (GlobalData, IWSR)
Bangladesh	1961-1999: FAO
	2000-2019: merged (FAO, OIV)
Barbados	1961-1999: FAO
	2000-2020: merged (GlobalData, IWSR, OIV)
Belarus	1980-1999: FAO
	2000-2004: WHO GSAH
	2005-2020: Yearbook of Statistics
Belgium	1963-1999: WDT
	2000-2007: FAO
	2008-2020: merged (Belgium Tax Administration Department, WHO GSAH)
Belize	1961-1999: FAO
	2000-2020: merged (GlobalData, IWSR, OIV)
Benin	1961-1999: FAO
	2000-2020: merged (FAO [2018 to 2020 missing]* **, GlobalData, Wine Institute)
Bhutan	1961-1999: FAO
	2000-2017: merged (FAO [2018 to 2020 missing] * **, WI [2019 and 2020 missing] * **)
Bolivia	1961-1999: FAO
	2000-2020: merged (GlobalData, IWSR, OIV)
Bosnia and Herzegovina	1987-1999 FAO
	2000-2020: merged (GlobalData, IWSR, OIV)
Botswana	1961-1999: FAO
	2000-2020: merged (FAO [2020 missing] **, GlobalData, IWSR)
Brazil	1963-1999: WDT
	2000-2019: merged (GlobalData, IWSR, OIV)
Brunei Darussalam	1961-1999: FAO
	2000-2017: merged (FAO [2015 to 2020 missing] * **, OIV)
Bulgaria	1963-1999: WDT
	2000-2020: merged (GlobalData, IWSR, OIV)
Burkina Faso	1961-1999: FAO
	2000-2020: merged (FAO [2020 missing] **, GlobalData, OIV)
Burundi	1961-1999: FAO
	2000-2020: merged (FAO [2020 missing] **, GlobalData, OIV)

Country	Data source recorded
Cabo Verde	1961-1999: FAO
	2000-2017: merged (FAO [2020 missing] **, WI)
Cambodia	1961-1999: FAO
	2000-2018: merged (GlobalData, IWSR)
Cameroon	1961-1999: FAO
	2000-2020: merged (FAO, GlobalData, OIV)
Canada	1961-1992: WDT
	1993-2020: Statistics Canada
Central African Republic	1961-1999: FAO
	2000-2020: merged (FAO [2020 missing] **, GlobalData, OIV)
Chad	1961-1999: FAO
	2000-2020: merged (FAO [2020 missing] **, GlobalData, OIV)
Chile	1961-1999: WDT
	2000-2020: WHO GSAH
	2016-2019: merged (GlobalData, WHO GSAH)
China	1961-1984: FAO
	1985-1999: WDT
	2000-2020: National Bureau of Statistics- Trade data
Colombia	1961-1962: FAO
	1963-1999: WDT
	2000-2020: merged (GlobalData, IWSR, OIV)
Comoros	1961-2000: FAO
	2001-2017: merged (FAO [2020 missing] **, OIV)
Congo	1961-1999: FAO
	2000-2020: merged (GlobalData, IWSR, OIV)
Cook Islands	2000-2015: merged (FAO [2014 missing] *, WI)
Costa Rica	1961-1999: FAO
	2000-2020: Instituto sobre Alcoholismo y Farmacodependencia
Côte d'Ivoire	1961-1999: FAO
	2000-2020: merged (FAO [2020 missing] **, GlobalData, IWSR, OIV)
Croatia	1987-1999: FAO
	2000-2020: merged (GlobalData, IWSR, OIV)
Cuba	1961-1999: WDT
	2000-2020: merged (GlobalData, IWSR, OIV)
Cyprus	1961-1999: WDT
	2000-2015: Statistics Cyprus
Czechia	1993-1999: WDT
	2000-2018: Czech Statistical Office
Democratic People's Republic of Korea	1961-2019: FAO
Democratic Republic of the Congo	1961-1999: FAO
	2000-2020: merged (GlobalData, IWSR, OIV)
Denmark	1961-1989: WDT
	1990-2020: Statistics Denmark
Djibouti	1961-1999: FAO
	2000-2020: IWSR, OIV
Dominica	1990-1999: FAO
	2000-2020: merged (FAO [2020 missing] **, IWSR, WI [2016 to 2020 missing] * **)
Dominican Republic	1961-1999: FAO
	2000-2018: merged (GlobalData, IWSR, OIV)
Ecuador	1961-1999: FAO
	2000-2020: merged (GlobalData, IWSR)
Egypt	1961-1999: FAO
	2000-2020: merged (GlobalData, IWSR, OIV)
El Salvador	1961-1999: FAO
	2000-2020: merged (GlobalData, IWSR, OIV)
Equatorial Guinea	1983-1999: FAO
	2000-2018: merged (GlobalData, IWSR [2000 to 2004 missing]*)
Eritrea	1961-1999: FAO
	2000-2020: merged (FAO [2014 to 2020 missing]* **, GlobalData)
Estonia	1990-1999: WDT
	2000-2020: Estonian Institute of Economic Research
Eswatini	1990-1999: FAO
	2000-2017: merged (FAO [2020 missing] **, GlobalData, OIV)
Ethiopia	1961-1999: FAO
	2000-2018: merged (FAO [2020 missing]**, GlobalData, IWSR [2000 to 2006 missing]*, OIV)
Fiji	1961-1999: FAO

Country	Data source recorded
	2000-2017: merged (FAO [2020 missing]**, GlobalData, WI)
Finland	1961-1989: National Research and Development Centre for Welfare and Health (STAKES)
	1990-2020: Statistics Finland
France	1961-1999: WDT
	2000-2020: INSEE [National Institute of Statistics and Economic Studies]/Observatoire Français des drogues et des toxicomanies [French Monitoring Centre for Drugs and Drug Addiction]
Gabon	1961-1999: FAO
	2000-2020: merged (GlobalData, IWSR, OIV)
Gambia	1961-1999: FAO
	2000-2020: merged (FAO [2020 missing]**, GlobalData, WI)
Georgia	1990-1999: FAO
	2000-2020: merged (GlobalData, IWSR, OIV)
Germany	1961-1990: WDT
	1991-1999: German Statistical Office (DeStatis)
	2000-2004: WHO GSAH
	2005-2010: Statistics Germany (DeStatis)
	2011-2020: Federal Office for Information Security (BSI)
Ghana	1961-1999: FAO
	2000-2018: merged (FAO [2020 missing]**, GlobalData, IWSR, WI)
Greece	1961-1999: WDT
	2000-2020: merged (GlobalData, IWSR, OIV)
Grenada	1961-1999: FAO
	2000-2020: merged (GlobalData, IWSR, OIV)
Guatemala	1961-1999: FAO
	2000-2020: merged (GlobalData, IWSR, OIV)
Guinea	1961-1999: FAO
	2000-2020: merged (FAO [2020 missing]**, GlobalData, OIV)
Guinea-Bissau	1961-1999: FAO
	2000-2020: merged (FAO[2020 missing]**, OIV)
Guyana	1961-1989: FAO
	1990-1999: WDT
	2000-2020: merged (GlobalData, IWSR, OIV)
Haiti	1961-1999: FAO
	2000-2020: merged (GlobalData, IWSR, OIV)
Honduras	1961-1999: FAO
	2000-2020: merged (GlobalData, IWSR, OIV)
Hungary	1961-1999: WDT
	2000-2004: WHO GSAH
	2005-2019: Hungarian Central Statistical Office
Iceland	1961-1999: WDT
	2000-2020: Statistics Iceland
India	1961-1999: FAO
	2000-2020: merged (GlobalData, IWSR, OIV)
Indonesia	1961-1999: FAO
	2000-2020: merged (GlobalData, IWSR, OIV)
Iran (Islamic Republic of)	1961-1999: FAO
	2000-2020: merged (FAO [2020 missing]**, IWSR)
Iraq	1961-1999: FAO
	2000-2020: merged (FAO, IWSR, OIV)
Ireland	1961-1999: WDT
	2000-2020: Irish Revenue Commissioners Reports
Israel	1961-1999: FAO
	2000-2020: merged (GlobalData, IWSR, OIV)
Italy	1961-1999: WDT
	2000-2004: AssoBirra Annual Report
	2005-2014: WHO GSAH
	2015-2020: AssoBirra Annual report
Jamaica	1961-1999: FAO
	2000-2020: merged (GlobalData, IWSR, OIV)
Japan	1961-1988: World Drink Trends (WDT)
	1989-2019: National Tax Agency of Japan
Jordan	1961-1999: FAO
	2000-2018: merged (GlobalData, IWSR, OIV)
Kazakhstan	1988-1999: FAO
	2000-2006: merged (GlobalData, IWSR, OIV)
	2007-2020: Statistical Yearbook
Kenya	1961-1999: FAO
	2000-2020: merged (FAO [2020 missing]**, GlobalData, IWSR)

Country	Data source recorded
Kiribati	1961-1999: FAO
	2000-2020: merged (FAO [2020 missing]**, WI)
Kuwait	1961-1999: FAO
	2000-2020: Alcohol prohibition
Kyrgyzstan	1985-1999: FAO
	2000-2020: National Statistic Committee of the Kyrgyz Republic
Lao People's Democratic Republic	1961-1999: FAO
	2000-2020: merged (FAO [2020 missing]**, GlobalData, IWSR)
Latvia	1980-1999: WDT
	2000-2020: Slimību profilakses un kontroles centrs
Lebanon	1961-1999: FAO
	2000-2020: merged (GlobalData, IWSR, OIV)
Lesotho	1961-1999: FAO
	2000-2020: merged (FAO [2020 missing]**, GlobalData, OIV)
Liberia	1961-1999: FAO
	2000-2020: merged (FAO [2020 missing]**, GlobalData, OIV)
Libya	1961-1999: FAO
	2000-2020: merged (FAO [2020 missing]**, OIV)
Lithuania	1984-1999: FAO
	2000-2004: WHO GSAH
	2005-2020: Statistics Lithuania
Luxembourg	1961-1999: FAO
	2000-2020: Average of France and Germany consumption
Madagascar	1961-1999: FAO
	2000-2020: merged (FAO [2020 missing]**, GlobalData, OIV)
Malawi	1961-1999: FAO
	2000-2020: merged (FAO [2020 missing]**, GlobalData, OIV)
Malaysia	1961-1979: FAO
	1980-1999: WDT
	2000-2020: merged (GlobalData, IWSR, OIV)
Maldives	1961-1999: FAO
	2000-2017: merged (FAO, Wine Institute)
Mali	1961-1999: FAO
	2000-2020: merged (FAO [2020 missing]**, GlobalData, OIV)
Malta	1961-1987: FAO
	1988-1999: WDT
	2000-2020: merged (GlobalData, IWSR, OIV)
Marshall Islands	No data
Mauritania	1961-2000: FAO
	2001-2020: Alcohol prohibition
Mauritius	1961-1999: FAO
	2000-2018: merged (FAO, GlobalData, IWSR [2000 to 2004 missing]*)
Mexico	1961-1989: WDT
	1990-1999: Consultores Internacionales, S.C. [International Consultants, S.C.]
	2000-2006: merged (GlobalData, IWSR, OIV)
	2007-2020: National Institute of Statistics and Geography
Micronesia (Federated States of)	1961-1999: FAO
	2000-2009: merged (FAO, WHO GSAH)
	2010-2015: WHO GSAH
Monaco	No data
Mongolia	1961-1999: FAO
	2000-2020: National Statistical Office of Mongolia
Montenegro	2006-2018: Merged (GlobalData, IWSR, OIV, Statistical Yearbook)
Morocco	1961-1999: WDT
	2000-2020: merged (FAO, GlobalData, IWSR, OIV)
Mozambique	1961-1999: FAO
	2000-2020: merged (FAO [2020 missing]**, GlobalData, IWSR , OIV)
Myanmar	1961-1999: FAO
	2000-2020: merged (GlobalData, IWSR, OIV)
Namibia	1961-1999: FAO
	2000-2020: merged (FAO, GlobalData, IWSR)
Nauru	2000-2013: FAO
Nepal	1961-1999: FAO
	2000-2020: merged (FAO [2020 missing]**, GlobalData, OIV)
Netherlands	1961-1999: WDT
	2000-2020: STAP-Dutch Institute for Alcohol Policy

Country	Data source recorded
New Zealand	1963-1996: WDT
	1997-2020: Statistics New Zealand
Nicaragua	1961-1999: FAO
	2000-2020: merged (GlobalData, IWSR)
Niger	1961-1999: FAO
	2000-2020: merged (FAO [2020 missing]**, GlobalData, OIV)
Nigeria	1961-1999: FAO
	2000-2020: merged (FAO, GlobalData, IWSR [2000 to 2004 missing]*, OIV)
Niue	1990-1999: FAO
	2000-2017: merged (FAO [2014 to 2017 missing]*, Wine Institute)
North Macedonia	1992-1999: FAO
	2000-2020: merged (GlobalData, IWSR, OIV)
Norway	1961-1966: WDT
	1967-1980: Norwegian Institute for Alcohol and Drug Research (SIRUS)
	1981-1999: Statistics Norway
	2000-2004 WHO GSAH
	2005-2020: Statistics Norway
Oman	1961-1999: FAO
	2000-2020: merged (GlobalData, IWSR, Wine Institute [2018 to 2020 missing]* **)
Pakistan	1961-1999: FAO
	2000-2012: merged (FAO, GlobalData, IWSR)
	2013-2018: merged (FAO [2020 missing]**, GlobalData, IWSR, OIV)
Palau	No data
Panama	1961-1999: FAO
	2000-2020: merged (GlobalData, IWSR)
Papua New Guinea	1961-1999: FAO
	2000-2020: merged (FAO [2020 missing]**, GlobalData, OIV)
Paraguay	1961-1999: WDT
	2000-2020: merged (GlobalData, IWSR, OIV)
Peru	1961-1999: WDT
	2000-2020: merged (FAO [2020 missing]**, GlobalData, IWSR, OIV)
Philippines	1961-1999: FAO
	2000-2018: merged (GlobalData, IWSR, OIV)
Poland	1961-1999: WDT
	2000-2004: WHO GSAH
	2005-2020: Statistics Poland
Portugal	1963-1999: WDT
	2000-2016: Statistics Portugal
Qatar	1961-1999: FAO
	2000-2020: merged (GlobalData, IWSR)
Republic of Korea	1961-1999: FAO
	2000-2020: WHO GSAH
Republic of Moldova	1992-1999: FAO
	2000-2020: merged (GlobalData, IWSR, OIV, Statistical Yearbook of the Republic of Moldova [2020 missing]**)
Romania	1963-1999: WDT
	2000-2019: National Institute of Statistics
Russian Federation	1963-1999: WDT
	2000-2020: Russian Statistical Office (ROSSTAT)
Rwanda	1961-1999: FAO
	2000-2020: merged (FAO [2020 missing]**, GlobalData, OIV)
Saint Kitts and Nevis	1990-1999: FAO
	2000-2020: merged (GlobalData, IWSR)
Saint Lucia	1961-1999: FAO
	2000-2020: merged (GlobalData, IWSR)
Saint Vincent and the Grenadines	1990-1999: FAO
	2000-2020: merged (GlobalData, IWSR, OIV)
Samoa	1961-1999: FAO
	2000-2017: merged (FAO [2020 missing]**,, GlobalData, Wine Institute)
San Marino	No data
Sao Tome and Principe	1961-2000: FAO
	2001-2017: merged (FAO [2020 missing]**, Wine Institute)
Saudi Arabia	1961-1999: FAO
	2000-2020: Alcohol prohibition
Senegal	1961-1999: FAO

Country	Data source recorded
	2000-2020: merged (FAO, GlobalData, IWSR)
Serbia	2006-2020: merged (GlobalData, IWSR, OIV)
Seychelles	1961-1999: FAO
	2000-2020: merged (FAO, GlobalData, IWSR)
Sierra Leone	1990-1999: FAO
	2000-2017: merged (FAO [2020 missing]**, GlobalData, Wine Institute)
Singapore	1961-1984: FAO
	1985-2002: WDT
	2003-2020: Singapore National Statistics
Slovakia	1961-1999: WDT
	2000-2020: Statistics Slovakia (SlovStat)
Slovenia	1981-1999: FAO
	2000-2005: WHO GSAH
	2006-2020: National Institute of Public Health
Solomon Islands	1961-1999: FAO
	2000-2020: merged (FAO [2020 missing]**, GlobalData, OIV)
Somalia	2000-2020: Alcohol prohibition
South Africa	1961-1999: WDT
	2000-2020: South African Wine Industry Information and Systems (SAWIS)
South Sudan	No data
Spain	1962-1999: WDT
	2000-2004: Agencia Tributaria [Spanish Tax Agency]
	2005-2009: WHO GSAH
	2010: merged (Spanish Tax Agency, WHO Global Survey on Alcohol and Health)
	2011-2014: merged (Spanish Tax Agency, Ministry of Agriculture, Fisheries and Food)
	2015-2020: Spanish Tax Agency
Sri Lanka	1961-1999: FAO
	2000-2011: merged (Excise Data, WHO Global Surveys on Alcohol and Health)
	2012-2017: Excise Data
Sudan	1961-2016: merged (FAO [2020 missing]**, OIV)
Suriname	1961-1999: FAO
	2000-2020: merged (GlobalData, IWSR, OIV)
Sweden	1961-1999: WDT
	2000-2020: Central Association for Alcohol and Drug Information
Switzerland	1961-1999: WDT
	2000-2020: Federal Customs Administration
Syrian Arab Republic	1961-1999: FAO
	2000-2020: merged (IWSR, OIV)
Tajikistan	1992-1999: FAO
	2000-2020: merged (GlobalData, IWSR, OIV)
Thailand	1961-1984: FAO
	1985-1999: WDT
	2000-2007: merged (GlobalData, IWSR)
	2008-2013: WHO Global Surveys on Alcohol and Health
	2014-2020: Ministry of Public Health
Timor-Leste	1961-2006: FAO
	2007-2020: Merged (FAO [2020 missing]**, OIV)
Togo	1990-1999: FAO
	2000-2020: merged (FAO [2020 missing]**, GlobalData, IWSR, OIV)
Tonga	1990-1999: FAO
	2000-2020: merged (FAO [2014 to 2020 missing]* **, GlobalData, WI)
Trinidad and Tobago	1961-1999: FAO
	2000-2020: merged (GlobalData, IWSR)
Tunisia	1961-1999: WDT
	2000-2020: merged (GlobalData, IWSR, OIV)
Turkey	1961-1999: WDT
	2000-2004: merged (GlobalData, IWSR, OIV)
	2005-2020: Turkish Statistical Institute
Turkmenistan	1992-1999: FAO
	2000-2020: merged (GlobalData, IWSR, OIV)
Tuvalu	1990-2017: merged (FAO [2014 to 2017 missing]*, WI)
Uganda	1961-1999: FAO
	2000-2020: merged (FAO, GlobalData, IWSR)
Ukraine	1975, 1980-1990: WDT

Country	Data source recorded
	1991-1999: FAO
	2000-2018: merged (GloablData, IWSR, OIV, Statistics Ukraine)
United Arab Emirates	1972-1999: Food and Agriculture Organization of the UN (FAO)
	2000-2020: IWSR
United Kingdom of Britain and Northern Ireland	1961-1999: WDT
	2000-2020: Alcohol Bulletins (HM Revenue and Customs)
United Republic of Tanzania	1990-1999: FAO
	2000-2020: merged (FAO [2020 missing]**, GlobalData, IWSR)
United States of America	1961-2020: National Institute on Alcohol Abuse and Alcoholism (NIAAA)
Uruguay	1961-1999: WDT
	2000-2020: merged (Direccion General Impositiva [General Tax Directorate], Instituto Nacional de Vitivinicultura [National Institute for Viticulture])
Uzbekistan	1992-1999: FAO
	2000-2020: merged (GlobalData, IWSR, OIV)
Vanuatu	1961-1999: FAO
	2000-2020: merged (FAO [2020 missing]**, GlobalData, WI)
Venezuela (Bolivarian Republic of)	1961-1999: WDT
	2000-2020: merged (GlobalData, IWSR, OIV)
Viet Nam	1961-1979: FAO
	1980-1999: WDT
	2000-2020: merged (GlobalData, IWSR, OIV)
Yemen	1961-2012: FAO
	2013-2017: merged (FAO [2020 missing]**, OIV)
Zambia	1990-1999: FAO
	2000-2017: merged (FAO[2020 missing]**, GlobalData, OIV)
Zimbabwe	1961-1999: FAO
	2000-2020: merged (WHO GSAH, Delta Corp., FAO [2020 missing]**, OIV)

* Missing data for 2000 to 2019 were imputed using a last value carried forward method
** Missing data for 2020 were estimated using country data; if country data were not available, regional level data were used

Countries with a total ban on alcohol consumption

Afghanistan, Brunei Darussalam, Iran (Islamic Republic of), Libya, Maldives, Mauritania, Saudi Arabia, Somalia, Sudan, Yemen.

Literature search strategies to identify relevant records for drinking status and heavy episodic drinking

Table S2. Step-wise search in Ovid (MEDLINE and Embase)

**Ovid MEDLINE: Epub Ahead of Print, In-Process & Other Non-Indexed Citations, Ovid MEDLINE® Daily and Ovid MEDLINEand _Embase Classic+Embase**: January 1, 2010 to current (August 6, 2020)	
1	drinking behavior/ or alcohol drinking/ or binge drinking/
2	(alcohol* adj3 (consum* or drink* or intake or behavio?r* or trend* or abstain* or abstinen*)).ti,ab,kf,kw,hw.
3	(drink* adj2 (alcohol* or liquor* or wine or beer or spirits or habit* or behavio?r or binge or heavy or episodic or former* or current* or daily or level* or rate or rates or frequen*)).ti,ab,kf,kw,hw.
4	(lifetime abstainer* or lifetime abstinen*).ti,ab,kf,kw,hw.
5	1 OR 2 OR 3 OR 4
6	national* adj3 (survey* or population* or general population* or data* or study or studies or rate or rates)).ti,ab,kf,kw,hw.
7	5 AND 6
8	limit 7 to yr="2010 -Current"

Google and Google Scholar

Search terms: alcohol population survey [*country*] [*year]*

The search term [country] was substituted with each of the countries in Table 1 and [year] was substituted with years 2010 to 2020. Once 50 consecutive irrelevant results for a given country and year were found, the next country and/or year were searched. For example, "alcohol population survey Canada 2020" was updated to "alcohol population survey Central African Republic 2010" after 50 consecutive hits were irrelevant.

Intergovernmental organizations and global health databases

Search terms: "alcohol", "current drinking", "current drinker", "abstain", "abstention" "abstainer", "lifetime abstention", "lifetime abstainer", "former drinking", "former drinker"

Databases that allowed searches to be restricted to specific years (i.e., 2010 to 2020) or countries were applied, where relevant. If reports were not available, statistical tables were used if survey methods were reported.

Table S3. Intergovernmental organizations and global health databases searched in the systematic review

**Database**	**Website**
Demographic and Health Surveys	https://dhsprogram.com/publications/index.cfm
Global Health Data Exchange	http://ghdx.healthdata.org/
International Labour Organization Microdata Repository	https://www.ilo.org/surveyLib/index.php/home
International Household Survey Network	https://catalog.ihsn.org/catalog
UNICEF Multiple Indicator Cluster Surveys	https://mics.unicef.org/surveys
World Health Organization Multi-Country Studies Data Archive	https://apps.who.int/healthinfo/systems/surveydata/index.php/catalog

Country-level Ministry of Health and Statistical Services websites

Search terms: "alcohol", "current drinking", "current drinker", "abstain", "abstention" "abstainer", "lifetime abstention", "lifetime abstainer", "former drinking", "former drinker"

Search terms were applied in national Ministry of Health and Statistical Services websites, with restrictions to specific years (i.e., 2010 to 2020), where relevant. A list of country-specific websites are available respectively through https://www.gfmer.ch/Medical_search/Ministry_health.htm and

https://en.wikipedia.org/wiki/Category:National_statistical_services. If reports were not available, statistical tables were used if survey methods were reported.

Individual-level survey datasets

Individual-level datasets from international and national general population survey initiatives were obtained through requests to investigators or by accessing publicly available microdata (Table S2). Data extractions were completed using *R* statistical software and included sample sizes, years of survey administrations, as well as weighted sex-specific and age-specific point estimates of drinking status and heavy episodic drinking, with standard errors or 95% uncertainty intervals, if available.

Once extracted, this data was added to our database of individual-level survey datasets (see Manthey and colleagues for previous data collection), resulting in aggregate estimates for 120 countries. The analysis was based on 824,653 surveyed individuals between 1993 and 2019 and the total database of individual-level datasets includes: 'STEPwise approach to surveillance' (87 countries), 'Gender, Alcohol and Culture: An international Study/ Gender and Alcohol's Harm to Others' (39 countries), 'Canadian Tobacco and Drugs Survey' 2015 and 2017 (country: Canada), 'National Epidemiologic Survey on Alcohol and Related Conditions' Wave 2 and Wave 3 (country: United States of America), and the 'Russian Longitudinal Monitoring Survey' (country: Russian Federation). This data was then combined with the published survey-derived estimates as part of the modelling strategy.

Table S4. Inclusion and exclusion criteria

Inclusion criteria:	
	Data are from any survey representative of the general population at the national or regional level
	Data were reported for adults (any age group 15 years of age and older)
	Data reported the time frame over which the prevalence of current drinkers, former drinkers, and lifetime abstainers and heavy episodic drinkers were measured over.
	For heavy episodic drinkers the prevalence was reported among the total population or among current drinkers
Exclusion criteria:	
	Surveys of specific populations (i.e., veterans, homeless etc)

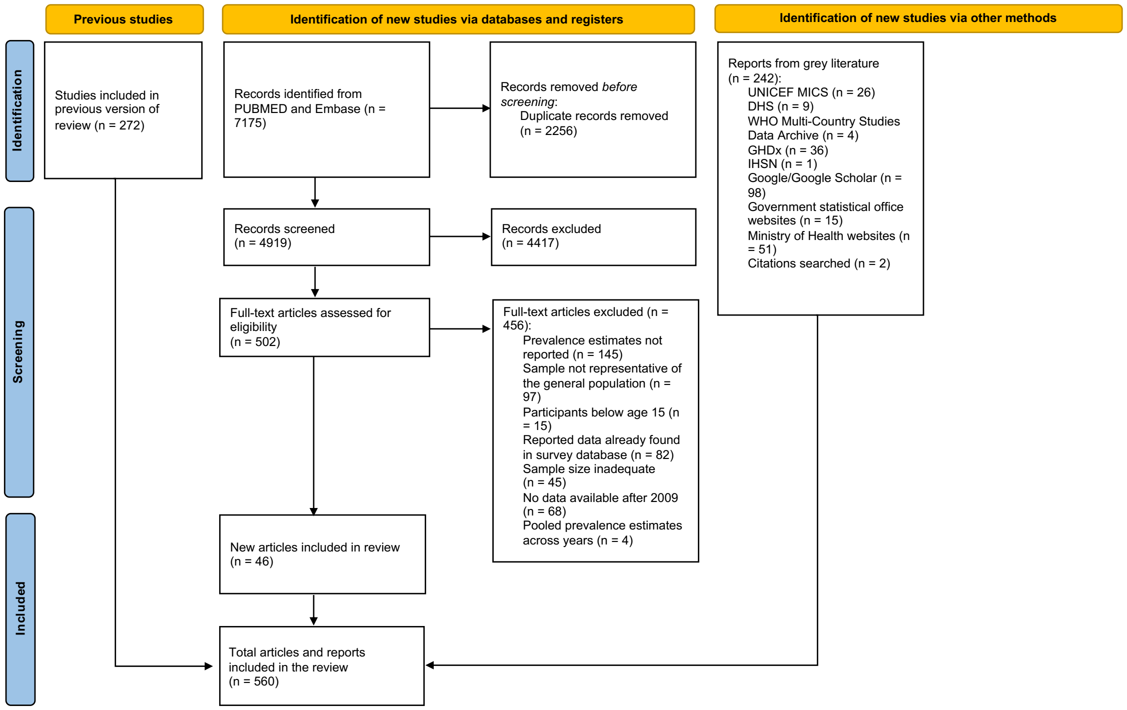

Figure S1. PRISMA flow diagram for the systematic search of surveys of population surveys on drinking status

Table S5. Surveys included in the modelling of drinking statuses and heavy episodic drinking

Region	Country	Sources of alcohol patterns of consumption data by WHO Member State
AFR	Algeria	World Health Organization (2003a). STEPwise approach to surveillance (STEPS) Survey. (http://www.who.int/chp/steps/en/. Accessed: 01/11/2018).
AFR	Angola	-
AFR	Benin	Institut National De La Statistique Et De L'analyse Economique (INSAE) (2015). Enquête par grappes à indicateurs multiples (MICS) 2014. Résultats clés. Cotonou, Bénin: Institut National de la Statistique et de l'Analyse Economique.
		World Health Organization (2007). STEPwise approach to surveillance (STEPS) Survey. (http://www.who.int/chp/steps/en/. Accessed: 01/11/2018).
		World Health Organization (2008). STEPwise approach to surveillance (STEPS) Survey. (http://www.who.int/chp/steps/en/. Accessed: 01/12/2018).
		World Health Organization (2015). STEPwise approach to surveillance (STEPS) Survey. (https://extranet.who.int/ncdsmicrodata/index.php/catalog/STEPS. Accessed: 07/02/2020).
		World Health Organization, Centers for Disease Control and Prevention (2020). Global School-based Student Health Survey – Benin 2016 Fact Sheet. (https://extranet.who.int/ncdsmicrodata/index.php/catalog/627. Accessed: 06/11/2020).
AFR	Botswana	Central Statistics Office, National AIDS Coordinating Agency (2008). Botswana AIDS Impact Survey 2008. Gaborone, Botswana: Central Statistics Office.
		Letamo G, Bowelo M, Majelantle RG (2016). Prevalence of substance use and correlates of multiple substance use among school-going adolescents in Botswana, African journal of drug and alcohol studies.15(2):75-89.
		Machisa M, van Dorp R (2012). The Gender Based Violence Indicators Study Botswana. GL Botswana: Gaborone, Botswana.
		World Health Organization (2007). STEPwise approach to surveillance (STEPS) Survey. (http://www.who.int/chp/steps/en/. Accessed: 01/11/2018).
		World Health Organization (2014b). STEPwise approach to surveillance (STEPS) Survey. (http://www.who.int/chp/steps/en/. Accessed: 01/12/2018).
AFR	Burkina Faso	World Health Organization (2005a). Burkina Faso World Health Survey 2002-2003. Geneva, Switzerland: World Health Organization.
		World Health Organization (2013c). STEPwise approach to surveillance (STEPS) Survey. (http://www.who.int/chp/steps/en/. Accessed: 01/15/2018).
AFR	Burundi	Ministère à la Présidence chargé de la Bonne Gouvernance et du Plan [Burundi] (MPBGP), Ministère de la Santé Publique et de la Lutte contre le Sida [Burundi] (MSPLS), Institut de Statistiques et d'Études Économiques du Burundi (ISTEEBU), et ICF (2017). Troisième Enquête Démographique et de Santé au Burundi 2016-2017. ISTEEBU, MSPLS, et ICF. Bujumbura, Burundi.
AFR	Cabo Verde	World Health Organization (2007). STEPwise approach to surveillance (STEPS) Survey. (http://www.who.int/chp/steps/en/. Accessed: 01/11/2018).
AFR	Cameroon	Institut National de la Statistique (2015). Enquête par grappes à indicateurs multiples (MICS5), 2014, Rapport Final. Yaoundé, Cameroun: Institut National de la Statistique.
		World Health Organization (2003a). STEPwise approach to surveillance (STEPS) Survey. (http://www.who.int/chp/steps/en/. Accessed: 01/11/2018).
AFR	Central African Republic	Pilleron S, Desport JC, Jesus P, Mbelesso P, Ndamba-Bandzouzi B , et al. (2015). Diet, alcohol consumption and cognitive disorders in Central Africa: A study from the EPIDEMCA Program, The Journal of Nutrition, Health & Aging. 19:657-67.
		World Health Organization (2010). STEPwise approach to surveillance (STEPS) Survey. (http://www.who.int/chp/steps/en/. Accessed: 01/12/2018).
AFR	Chad	Institut National de la Statistique, des Études Économiques Et Démographiques, Ministère De La Santé Publique, ICF International (2015). Enquête démographique et de santé et à Indicateurs multiples (EDS-MICS 2014-2015). Maryland, USA: Institut National de la Statistique des Études Économiques et Démographiques, Ministère de la Santé Publique, ICF International.
		World Health Organization (2008). STEPwise approach to surveillance (STEPS) Survey. (http://www.who.int/chp/steps/en/. Accessed: 01/12/2018).
AFR	Comoros	World Health Organization (2011b). STEPwise approach to surveillance (STEPS) Survey. (http://www.who.int/chp/steps/en/. Accessed: 01/12/2018).
AFR	Congo	Institut National de la Statistique Et De L'analyse Economique (INSAE) (2015). Enquête par grappes à indicateurs multiples (MICS), 2014, Résultats clés. Cotonou, Bénin: Institut National de la Statistique et de l'Analyse Economique.
		Pilleron S, Desport JC, Jesus P, Mbelesso P, Ndamba-Bandzouzi B, Dartigues JF, et al. (2015). Diet, Alcohol Consumption and Cognitive Disorders in Central Africa: A Study from the EPIDEMCA Program, The Journal of Nutrition, Health & Aging.19:657-67.
		World Health Organization (2004). STEPwise approach to surveillance (STEPS) Survey. (http://www.who.int/ncds/surveillance/steps/en/. Accessed: 01/12/2018).
AFR	Côte d'Ivoire	Ministre du plan et du developpement (2017). MICS la situation des femmes et des enfants en cote d'ivoire 2016. (https://www.unicef.org/cotedivoire/media/981/file/civ-Rapportfe2016.pdf.pdf. Accessed: 06/26/2019)
		N'cho SD, Zengbé-Acray P, Ahoussou EMK, Ekou FK, Kouassi DP, Sablé PS, et al. (2014). Consommation d'alcool en milieu urbain chez lesélèves du secondaire en Côte d'Ivoire, Santé Publique.26:107-114.
		World Health Organization (2005b). STEPwise approach to surveillance (STEPS) Survey. (http://www.who.int/ncds/surveillance/steps/en/. Accessed: 01/12/2018)
AFR	Democratic Republic of the Congo	World Health Organization (2005b). STEPwise approach to surveillance (STEPS) Survey. http://www.who.int/ncds/surveillance/steps/en/. Accessed: 01/12/2018
AFR	Equatorial Guinea	-
AFR	Eritrea	World Health Organization (2004). STEPwise approach to surveillance (STEPS) Survey. (http://www.who.int/ncds/surveillance/steps/en/. Accessed: 01/12/2018).
		World Health Organization (2010). STEPwise approach to surveillance (STEPS) Survey. (http://www.who.int/chp/steps/en/. Accessed: 01/12/2018)

Region	Country	Sources of alcohol patterns of consumption data by WHO Member State
AFR	Eswatini	Central Statistical Office (2016). Swaziland Multiple Indicator Cluster Survey 2014. Mbabane, Swaziland: Central Statistical Office & UNICEF.
		World Health Organization (2007). STEPwise approach to surveillance (STEPS) Survey. (http://www.who.int/chp/steps/en/. Accessed: 01/11/2018).
		World Health Organization (2014b). STEPwise approach to surveillance (STEPS) Survey. (http://www.who.int/chp/steps/en/. Accessed: 01/12/2018).
AFR	Ethiopia	Central Statistical Agency (CSA) [Ethiopia], ICF (2016). Ethiopia Demographic and Health Survey 2016. Addis Ababa, Ethiopia and Rockville, Maryland, USA: Central Statistical Agency and ICF.
		Central Statistical Agency [Ethiopia], ICF International (2012). Ethiopia Demographic and Health Survey 2011. Addis Ababa, Ethiopia and Calverton, Maryland, USA: Central Statistical Agency and ICF International.
		Erulkar A, Ferede A, Ambelu W, Girma W, Amdemikael A, GebreMedhin B, et al. (2010). Ethiopia young adult survey: A study in seven regions. Addis Ababa, Ethiopia: Population Council.
		World Health Organization (2006c). STEPwise approach to surveillance (STEPS) Survey. (http://www.who.int/chp/steps/en/. Accessed: 01/12/2018).
		World Health Organization (2015). STEPwise approach to surveillance (STEPS) Survey. (http://www.who.int/chp/steps/en/. Accessed: 07/02/2020).
AFR	Gabon	Mimbila-Mayi M, Vierin YN, Biloghe A, Moussavou-Mouyama A (2011). Enquête épidémiologique sur la consommation des substances addictives par les adolescents du Gabon, Sante.21:149-152.
		World Health Organization (2009b). STEPwise approach to surveillance (STEPS) Survey. (http://www.who.int/chp/steps/en/. Accessed: 01/12/2018).
AFR	Gambia	The Gambia Bureau of Statistics (2019). The Gambia Multiple Indicator Cluster Survey 2018, Survey Findings Report. Banjul, The Gambia: The Gambia Bureau of Statistics.
		World Health Organization (2010). STEPwise approach to surveillance (STEPS) Survey. (http://www.who.int/chp/steps/en/. Accessed: 01/12/2018).
AFR	Ghana	Biritwum R, Mensah G, Yawson A, Minicuci N (2013). Study on global AGEing and adult health (SAGE) Wave 1: The Ghana National Report. (https://www.who.int/healthinfo/sage/national_reports/en/. Accessed: 08/11/2020).
		Ghana Statistical Service (2018). Ghana Multiple Indicator Cluster Survey 2017/2018, Survey Findings Report. Accra, Ghana: Ghana Statistical Service.
		Ghana Statistical Service (GSS), Ghana Health Service (GHS), ICF Macro (2009). Ghana Demographic and Health Survey 2008. (https://dhsprogram.com/pubs/pdf/FR221/FR221[13Aug2012].pdf. Accessed: 06/16/2017).
		World Health Organization (2006c). STEPwise approach to surveillance (STEPS) Survey. (http://www.who.int/chp/steps/en/. Accessed: 01/12/2018).
AFR	Guinea	Institut National de la Statistique (2017). Enquête par grappes à indicateurs multiples (MICS, 2016), Rapport final. (https://microdata.worldbank.org/index.php/catalog/3483/related-materials. Accessed: 08/13/2020).
		World Health Organization (2009b). STEPwise approach to surveillance (STEPS) Survey. (http://www.who.int/chp/steps/en/. Accessed: 01/12/2018).
AFR	Guinea-Bissau	República Da Guiné-Bissau, Ministério Da Economia E Finanças, Secretaria De Estado Do Plano (2016). Inquérito aos Indicadores Múltiplos (MICS5) 2014, Relatório Final. Bissau, Guiné-Bissau: Ministério da Economia e Finanças e Direcção Geral do Plano & Instituto Nacional de Estatística (INE).
AFR	Kenya	Kamenderi M, Muteti J, Okjoma V, Kimani S, Kanana F, Kahiu C (2019). Status of Drugs and Substance Abuse among the General Population in Kenya, African Journal of Alcohol and Drug Abuse. 1:54-59.
		Kenya National Bureau of Statistics, Ministry of Health/Kenya, National AIDS Control Council/Kenya, Kenya Medical Research Institute, National Council for Population and Development/Kenya, et. Al (2015). Kenya Demographic and Health Survey 2014. Rockville, MD, USA: Kenya National Bureau of Statistics, Ministry of Health/Kenya, National AIDS Control Council/Kenya, Kenya Medical Research Institute, National Council for Population and Development/Kenya, ICF International.
		Kenya National Bureau of Statistics, Population Studies and Research Institute, United Nations Children's Fund (2016a). Bungoma County Multiple Indicator Cluster Survey 2013/14, Final Report. Nairobi, Kenya: Kenya National Bureau of Statistics, Population Studies and Research Institute, United Nations Children's Fund.
		Kenya National Bureau of Statistics, Population Studies and Research Institute, United Nations Children's Fund (2016b). Turkana County Multiple Indicator Cluster Survey 2013/14, Final Report. Nairobi, Kenya: Kenya National Bureau of Statistics, Population Studies and Research Institute, United Nations Children's Fund.
		Kenya National Bureau of Statistics, Population Studies and Research Institute, United Nations Children's Fund (2015). Kakamega county multiple indicator cluster survey 2013/14, Key Findings. Nairobi, Kenya: Kenya National Bureau of Statistics, Population Studies and Research Institute & United Nations Children's Fund.
		National Authority for the Campaign against Alcohol and Drug Abuse (2012). Rapid situation assessment of the status of drug and substance abuse in Kenya, 2012. Kenya: NACADA Authority.
		World Health Organization (2015). STEPwise approach to surveillance (STEPS) Survey. (http://www.who.int/chp/steps/en/. Accessed: 01/12/2018).
AFR	Lesotho	Ministry of Health and Social Welfare (MOHSW) [Lesotho], Bureau of Statistics (BOS) [Lesotho], ORC Macro (2005). Lesotho Demographic and Health Survey 2004. Calverton, Maryland: MOH, BOS, and ORC Macro.
		World Health Organization (2012b). STEPwise approach to surveillance (STEPS) Survey. (http://www.who.int/chp/steps/en/. Accessed: 01/12/2018).
AFR	Liberia	Liberia Institute of Statistics and Geo-Information Services (Lisgis), Ministry of Health and Social Welfare, National Aids Control Program, ICG International (2014). Liberia Demographic and Health Survey 2013. Monrovia, Liberia: Liberia Institute of Statistics and GeoInformation Services (LISGIS) & ICF International.
		World Health Organization (2011b). STEPwise approach to surveillance (STEPS) Survey. (http://www.who.int/chp/steps/en/. Accessed: 01/12/2018).
AFR	Madagascar	INSTAT, UNICEF (2019). Enquête par grappes à indicateurs multiples-MICS Madagascar, 2018, Rapport final. Antananarivo, Madagascar: INSTAT et UNICEF.

Region	Country	Sources of alcohol patterns of consumption data by WHO Member State
		Institut National de la Statistique (INSTAT), Fonds des Nations Unies pour l'Enfance (UNICEF) (2013). MICS Madagascar - Enquête par Grappes à Indicateurs Multiples, Sud 2012. (https://microdata.worldbank.org/index.php/catalog/3484/study-description. Accessed: 07/10/2020).
		Razanamihaja N, Befinoana, Marie-Laure B (2013). Alcohol Consumption by School-Going Adolescents in Madagascar: Prevalence and Associated Risk Factors, Journal of Alcoholism and Drug Dependence.2(1). doi:10.4172/2329-6488.1000145.
		World Health Organization (2005b). STEPwise approach to surveillance (STEPS) Survey. (http://www.who.int/ncds/surveillance/steps/en/. Accessed: 01/12/2018).
AFR	Malawi	National Statistical Office (2015). Malawi MDG Endline Survey 2014. Zomba, Malawi: National Statistical Office
		Price AJ, Crampin AC, Amberbir A, Kayuni-Chihana N, Musicha C, Tafatatha T, et. Al, (2018) . Prevalence of obesity, hypertension, and diabetes, and cascade of care in sub-Saharan Africa: a cross-sectional, population-based study in rural and urban Malawi. The Lancet Diabetes & endocrinology 6(3):208-222.
		World Health Organization (2009b). STEPwise approach to surveillance (STEPS) Survey. (http://www.who.int/chp/steps/en/. Accessed: 01/12/2018).
AFR	Mali	Institut National De La Statistique Et De L'analyse Economique (INSAE) (2016). Enquête par grappes à indicateurs multiples (MICS), 2015, Résultats clés. Cotonou, Bénin: Institut National de la Statistique et de l'Analyse Economique.
		World Health Organization (2007). STEPwise approach to surveillance (STEPS) Survey. (http://www.who.int/chp/steps/en/. Accessed: 01/11/2018).
AFR	Mauritania	-
AFR	Mauritius	Magliano D, Shaw J, Zimmet P, Pauvaday K, Deelchand A, Kowlessur S, et al. (2015). The trends in diabetes and cardiovascular disease risk in mauritius - The mauritius non communicable diseases survey 2015. (http://health.govmu.org/English/Statistics/Documents/Mauritius%20NCD%20Survey%202015%20Report.pdf. Accessed: 06/26/2017).
AFR	Mozambique	World Health Organization (2005b). STEPwise approach to surveillance (STEPS) Survey . (http://www.who.int/ncds/surveillance/steps/en/. Accessed: 01/12/2018).
AFR	Namibia	The Namibia Ministry of Health and Social Services, ICF International (2014). The Namibia demographic and health survey 2013. Rockville, MD: The Namibia Ministry of Health and Social Services & ICF International.
AFR	Niger	-
AFR	Nigeria	Adewuya AO, Ola BA, Coker O, Atilola O, Olugbile O, Ajomale T, et al. (2020). Descriptive Epidemiology of Alcohol Use in the Lagos State Mental Health Survey (LSMHS), Nigeria. International Journal of Mental Health and Addiction. 18:1-2.
		Greenfield TK, Bloomfield K, Wilsnack SC (2013b). GENAHTO Project (Gender and Alcohol's Harm to Others). (http://genahto.org/. Accessed: 07/02/2020).
		National Bureau of Statistics, United Nations Children's Fund (2017). Multiple Indicator Cluster Survey 2016-17, Survey Findings Report. Abuja, Nigeria: National Bureau of Statistics and United Nations Children's Fund.
		Ogah OS, Madukwe OO, Onyeonoro UU, Chukwuonye I I, Uke (2013). Cardiovascular risk factors and non-communicable diseases in Abia state. Nigeria: report of a community based survey, International Journal of Medicine and Biomedical Research.2:57-68.
		The International GENACIS-Project (2003). GENACIS - gender, alcohol and culture: an international study. (http://www.genacis.org/. Accessed: 01/10/2018).
		United Nations Office on Drugs and Crime (2018). Drug Use in Nigeria 2018. Vienna: United Nations Office on Drug Use and Crime.
AFR	Rwanda	Kanyoni M, Gishoma D, Ndahindwa V (2015). Prevalence of psychoactive substance use among youth in Rwanda. BMC research notes.8:190. https://doi.org/10.1186/s13104-015-1148-2.
		World Health Organization (2012b). STEPwise approach to surveillance (STEPS) Survey. (http://www.who.int/chp/steps/en/. Accessed: 01/12/2018)
AFR	Sao Tome and Principe	De Santiago, I, Ribeiro, R, Nicolau, LB, Marinho, RT, Pereira-Miguel J (2020). Consumption of Alcohol and Drugs in the School Population of Sao Tome and Principe, Acta medica portuguesa. 33(4), 237–245. https://doi.org/10.20344/amp.11876.
		World Health Organization (2009b). STEPwise approach to surveillance (STEPS) Survey. (http://www.who.int/chp/steps/en/. Accessed: 01/12/2018)
AFR	Senegal	-
AFR	Seychelles	World Health Organization (2004). STEPwise approach to surveillance (STEPS) Survey. (http://www.who.int/ncds/surveillance/steps/en/. Accessed: 01/12/2018).
		World Health Organization (2013). STEPwise approach to surveillance (STEPS) Survey. (http://www.who.int/chp/steps/en/. Accessed: 07/02/2020).
		World Health Organization, Centers for Disease Control and Prevention (2015). Global School-based Student Health Survey – Seychelles 2015 Fact Sheet. (https://www.who.int/ncds/surveillance/gshs/gshs_fs_seychelles_2015.pdf. Accessed: 08/10/2020).
AFR	Sierra Leone	Statistics Sierra Leone (2018). Sierra Leone Multiple Indicator Cluster Survey 2017, Survey Findings Report. Freetown, Sierra Leone: Statistics Sierra Leone.
		World Health Organization (2009b). STEPwise approach to surveillance (STEPS) Survey. (http://www.who.int/chp/steps/en/. Accessed: 01/12/2018)
AFR	South Africa	Human Sciences Research Council (2012). Study on global AGEing and adult health (SAGE) Wave 1: South Africa National Report. (http://www.hsrc.ac.za/en/research-outputs/view/6320/. Accessed: 08/16/2020).
		National Department of Health (NDoH), Statistics South Africa (Stats SA), South African Medical Research Council (SAMRC), ICF (2019). South Africa Demographic and Health Survey 2016. Pretoria, South Africa, and Rockville, Maryland, USA: NDoH, Stats SA, SAMRC, and ICF.
		Probst C, Shuper PA, Rehm J (2017). Coverage of alcohol consumption by national surveys in South Africa, Addiction.112(4):705-10. https://dx.doi.org/10.1111/add.13692.
		Shisana O, Labadarios D, Rehle T, Simbayi L, Zuma K, Dhansay A, et al. (2013). South African national health and nutrition examination survey (SANHANES-1). Cape Town, South Africa: HSRC Press.

Region	Country	Sources of alcohol patterns of consumption data by WHO Member State
		Shisan O, Rehle T, Simbayi LC, Zuma K, Jooste S, Zungu N, et al. (2014). South African national HIV prevalence, incidence and behaviour survey 2012. Cape Town: HSRC Press.
		Vellios NG, Van Walbeek CP (2017). Self-reported alcohol use and binge drinking in South Africa: Evidence from the National Income Dynamics Study, 2014 - 2015, South African medical journal = Suid-Afrikaanse tydskrif vir geneeskunde.108(1):33-9. https://dx.doi.org/10.7196/SAMJ.2017.v108i1.12615.
AFR	South Sudan	Lien L, Hauff E, Martinez P, Eide AH, Swarts L, Ayazi T (2016). Alcohol use in South Sudan in relation to social factors, mental distress and traumatic events, BMC Public Health 16:937. https://doi.org/10.1186/s12889-016-3605-9.
AFR	Togo	World Health Organization (2010). STEPwise approach to surveillance (STEPS) Survey. (http://www.who.int/chp/steps/en/. Accessed: 01/12/2018).
AFR	Uganda	Kavishe B, Biraro S, Baisley K, Vanobberghen F, Kapiga S, Munderi P, et al. (2015). High prevalence of hypertension and of risk factors for non-communicable diseases (NCDs): a population based cross-sectional survey of NCDS and HIV infection in Northwestern Tanzania and Southern Uganda, BMC Medicine.13:126.
		The International GENACIS-Project (2003). GENACIS - gender, alcohol and culture: an international study. (http://www.genacis.org/. Accessed: 01/10/2018).
		World Health Organization (2014b). STEPwise approach to surveillance (STEPS) Survey. (http://www.who.int/chp/steps/en/. Accessed: 01/12/2018).
AFR	United Republic of Tanzania	Francis JM, Weiss HA, Mshana G, Baisley K, Grosskurth H, Kapiga SH (2015). The epidemiology of alcohol use and alcohol use disorders among young people in northern Tanzania, PLOS One.10:e0140041.
		Kavishe B, Biraro S, Baisley K, Vanobberghen F, Kapiga S, Munderi P, et al. (2015). High prevalence of hypertension and of risk factors for non-communicable diseases (NCDs): a population based cross-sectional survey of NCDS and HIV infection in Northwestern Tanzania and Southern Uganda, BMC Medicine.13:126.
		World Health Organization (2011b). STEPwise approach to surveillance (STEPS) Survey. (http://www.who.int/chp/steps/en/. Accessed: 01/12/2018).
		World Health Organization (2012b). STEPwise approach to surveillance (STEPS) Survey. (http://www.who.int/chp/steps/en/. Accessed: 01/12/2018).
		World Health Organization, Centers for Disease Control and Prevention (2014). Global School-based Student Health Survey – Tanzania 2014 Fact Sheet. (https://www.who.int/ncds/surveillance/gshs/2014_Tanzania_GSHS_Fact_Sheet.pdf. Accessed: 08/15/2020).
AFR	Zambia	World Health Organization (2008). STEPwise approach to surveillance (STEPS) Survey. (http://www.who.int/chp/steps/en/. Accessed: 01/12/2018).
		World Health Organization (2017). STEPwise approach to surveillance (STEPS) Survey. (https://extranet.who.int/ncdsmicrodata/index.php/catalog/STEPS. Accessed: 07/02/2020).
AFR	Zimbabwe	Zimbabwe National Statistics Agency (ZIMSTAT) (2015). Zimbabwe Multiple Indicator Cluster Survey 2014, Final Report. (http://microdata.worldbank.org/index.php/catalog/2527. Accessed: 07/19/2017).
		Yaya S, Bishwajit G. (2019). Alcohol and tobacco use among men in Zambia and Zimbabwe. Journal of lifestyle medicine. 9(1):67.
AMR	Antigua and Barbuda	Inter-American Drug Abuse Control Commission (2016). A report on students' drug use in 13 Caribbean Countries : Antigua and Barbuda, The Bahamas , Barbados, Belize, Dominica, Grenada, Guyana, Haiti, Jamaica, St. Kitts and Nevis, St. Lucia, St. Vincent and the Grenadines, Trinidad and Tobago. (http://www.cicad.oas.org/Main/Template.asp?File=/oid/pub_eng.asp. Accessed: 06/17/2020).
		Inter-American Drug Abuse Control Commission, Organization of American States, National Drug Council (2008). Results from the Antigua and Barbuda national survey of substance abuse among secondary school students 2005. Antigua and Barbuda: National Drug Council.
		Swift JJ (2013). Antigua and Barbuda Secondary School Drug Prevalence Survey 2013. Antigua and Barbuda: Organization of American States, Inter-American Drug Abuse Control Commission.
AMR	Argentina	Ministerio De Salud De La Nación, Instituto Nacional De Estadísticas Y Censos (2015). Impreso en Argentina. (http://www.msal.gob.ar/images/stories/bes/graficos/0000000544cnt-2015_09_04_encuesta_nacional_factores_riesgo.pdf. Accessed: 06/16/2017).
		Ministerio De Salud De La Nación, Instituto Nacional De Estadísticas Y Censos (2018). 4° Encuesta Nacional de Factores de Riesgo. Informe Definitivo. (https://fagran.org.ar/wp-content/uploads/2019/11/4ta-encuesta-nacional-factores-riesgo.pdf. Accessed: 10/30/2020).
		Ministerio de Salud y Desarrollo Social de la Nación, el Instituto Nacional de Estadística y Censos (2009). Segunda encuesta nacional de factores de riesgo. Informe de Resultados. Capítulo 8: Alcohol. (https://bancos.salud.gob.ar/recurso/2da-encuesta-nacional-de-factores-de-riesgo-2009-alcohol. Accessed: 08/07/2019).
		Obot IS, Room R (2005). Alcohol, gender and drinking problems: perspectives from low and middle income countries. Geneva, Switzerland: World Health Organization.
		Observatorio Argentino de Drogas (2010). Estudio nacional en población de 12 a 65 años, sobre consumo de sustancias psicoactivas. (http://www.observatorio.gov.ar/media/k2/attachments/EstudioZNacionalZsobreZConsumoZdeZSustanciasZPsicoactivasZenZPoblacionZdeZ12ZaZ65ZAniosZAnioZ2010.pdf. Accessed: 07/09/2019).
		Observatorio Argentino de Drogas (2016). Sexto estudio nacional sobre consumo de sustancias psicoactivas en estudiantes de enseñanza media 2014. Análisis del consumo de alcohol en población Escolar. (http://www.observatorio.gov.ar/media/k2/attachments/2016-03-22ZInformeZConsumoZAlcoholZenZEscolares.pdf. Accessed: 07/09/2019).
		Observatorio Argentino de Drogas (2017). Estudio nacional en población de 12 a 65 años, sobre consumo de sustancias psicoactivas. . (https://observatorio.gob.ar/media/k2/attachments/INFORMEZGNEROZHOGARESZWEBZ1-3-19.pdf. Accessed: 07/09/2019).
		Salud Mental Adicciones, Ministerio De Salud (2011). Encuesta nacional sobre prevalencias de consumo de sustancias psicoactivas. (http://www.msal.gob.ar/saludmental/images/stories/info-equipos/pdf/2015-01-05_encuesta-nacional-sobre-prevalencias1.pdf. Accessed: 06/16/2017).

Region	Country	Sources of alcohol patterns of consumption data by WHO Member State
		The International GENACIS-Project (2003). GENACIS - gender, alcohol and culture: an international study. (http://www.genacis.org/. Accessed: 01/10/2018).
		Urribarri S, Valiero D (2011). Encuesta nacional sobre prevalencia de consumo de sustancias psicoactivas 2008. (https://www.entrerios.gov.ar/dec/publicaciones/SALUD/enprecosp%202008_2011.pdf. Accessed: 06/21/2017).
AMR	Bahamas	Inter-American Drug Abuse Control Commission (2016). A report on students' drug use in 13 Caribbean Countries : Antigua and Barbuda, The Bahamas , Barbados, Belize, Dominica, Grenada, Guyana, Haiti, Jamaica, St. Kitts and Nevis, St. Lucia, St. Vincent and the Grenadines, Trinidad and Tobago. (http://www.cicad.oas.org/Main/Template.asp?File=/oid/pub_eng.asp. Accessed: 06/17/2020).
		Ministry of Health, the Commonwealth of the Bahamas, Inter-American Drug Abuse Control Commission, Organization of American States (2018). Bahamas National Household Drug Prevalence Survey Report 2018. (http://www.cicad.oas.org/oid/pubs/BahamasNationalHouseholdDrugPrevalenceSurvey%20Report-2018.pdf. Accessed: 07/09/2019).
AMR	Barbados	Barbados Statistical Service (2014). Barbados Multiple Indicator Cluster Survey 2012. Bridgetown, Barbados: Barbados Statistical Service.
		Inter-American Drug Abuse Control Commission (2016). A report on students' drug use in 13 Caribbean Countries : Antigua and Barbuda, The Bahamas , Barbados, Belize, Dominica, Grenada, Guyana, Haiti, Jamaica, St. Kitts and Nevis, St. Lucia, St. Vincent and the Grenadines, Trinidad and Tobago. (http://www.cicad.oas.org/Main/Template.asp?File=/oid/pub_eng.asp. Accessed: 06/17/2020).
		Unwin N, Rose AMC, George KS, Hambleton IR, Howitt C (2015). The Barbados health of the nation survey: Core Findings. St Michael, Barbados: Chronic Disease Research Centre, The University of the West Indies, The Barbados Ministry of Health.
		World Health Organization (2007). STEPwise approach to surveillance (STEPS) Survey. (http://www.who.int/chp/steps/en/. Accessed: 01/11/2018).
AMR	Belize	Inter-American Drug Abuse Control Commission (2016). A report on students' drug use in 13 Caribbean Countries : Antigua and Barbuda, The Bahamas , Barbados, Belize, Dominica, Grenada, Guyana, Haiti, Jamaica, St. Kitts and Nevis, St. Lucia, St. Vincent and the Grenadines, Trinidad and Tobago. (http://www.cicad.oas.org/Main/Template.asp?File=/oid/pub_eng.asp. Accessed: 06/17/2020).
		National Drug Abuse Control Council (2014). National secondary school drug prevalence survey report - Report on Drug Use in Belize, Belize: National Drug Abuse Control Council.
		Statistical Institute of Belize, UNICEF Belize (2017). Belize Multiple Indicator Cluster Survey, 2015-2016, Final Report. Belmopan, Belize: Statistical Institute of Belize, UNICEF Belize.
		The Central America Diabetes Initiative (CAMDI) (2011). Survey of diabetes, hypertension and chronic disease risk factors - Belize, San José, San Salvador, Guatemala City, Managua and Tegucigalpa. Washington, DC: Pan American Health Organization. (http://www.paho.org/hq/index.php?gid=16710&option=com_docman&task=doc_view. Accessed: 01/11/2018).
		The International GENACIS-Project (2005) GENACIS - gender, alcohol and culture: an international study. (http://www.genacis.org/. Accessed: 01/10/2018).
AMR	Bolivia	Alcaraz F, Del C, Sempertegui Savatier S (2010). Consumo de drogas en Bolivia 1992-2010. Bolivia: Centro Latino Americano de Investigacion Cientfica.
		Consejo Nacional de Lucha Contra el Tráfico Ilícito de Drogas (2014). II Estudio Nacional de Prevalencia y Características del Consumo<br>de Drogas en Hogares Bolivianos de nueve Ciudades Capitales de Departamento, más la ciudad de El Alto 2014. La Paz, Bolivia: CONALTID.
		Instituto Nacional de Estadística (2012). Encuesta Nacional de Hogares 2012. (https://www.ilo.org/surveyLib/index.php/catalog/1550. Accesssed: 08/09/2019).
		Instituto Nacional de Estadística (2013). Encuesta Nacional de Hogares 2013. (https://www.ilo.org/surveyLib/index.php/catalog/1551. Accesssed: 08/09/2019).
		Instituto Nacional de Estadística (2014). Encuesta Nacional de Hogares 2014. (https://www.ilo.org/surveyLib/index.php/catalog/2172. Accessed: 08/09/2019).
		Instituto Nacional de Estadística (2015). Encuesta Nacional de Hogares 2015.(https://www.ilo.org/surveyLib/index.php/catalog/2171. Accesssed: 08/09/2019).
		Instituto Nacional de Estadística (2016). Encuesta Nacional de Hogares 2016. (https://www.ilo.org/surveyLib/index.php/catalog/1840/. Accesssed: 08/09/2019).
		Instituto Nacional de Estadística (2017). Encuesta Nacional de Hogares 2017. (http://censosbolivia.ine.gob.bo/ANDA4_3/index.php/catalog/499. Accesssed: 08/09/2019).
		Instituto Nacional de Estadística (2018). Encuesta Nacional de Hogares 2018. (http://anda.ine.gob.bo/index.php/catalog/78. Accessed: 08/09/2019).
AMR	Brazil	Brasil, Ministério da Saúde, Secretaria de Vigilância em Saúde, Secretaria de Gestão Estratégica e Participativa (2011). Vigitel Brasil 2010: vigilância de fatores de risco e proteção para doenças crônicas por inquérito telefônico. (http://bvsms.saude.gov.br/bvs/publicacoes/vigitel_2010.pdf. Accessed: 06/08/2019).
		Brasil, Ministério da Saúde, Secretaria de Vigilância em Saúde (2012). Vigitel Brasil 2011: Vigilância de Fatores de Risco e Proteção para Doenças Crônicas por Inquérito Telefônico. (http://bvsms.saude.gov.br/bvs/publicacoes/vigitel_brasil_2011_fatores_risco_doencas_cronicas.pdf. Accessed: 06/08/2019).
		Brasil, Ministério da Saúde, Secretaria de Vigilância em Saúde, Departamento de Vigilância de Doenças e Agravos não Transmissíveis e Promoção de Saúde (2013). Vigitel Brasil 2012: vigilância de fatores de risco e proteção para doenças crônicas por inquérito telefônico. (http://bvsms.saude.gov.br/bvs/publicacoes/vigitel_brasil_2012_vigilancia_risco.pdf. Accessed: 06/08/2019).
		Brasil, Ministério da Saúde, Secretaria de Vigilância em Saúde, Departamento de Vigilância de Doenças e Agravos não Transmissíveis e Promoção de Saúde (2014). Vigitel Brasil 2013: vigilância de fatores de risco e proteção para doenças crônicas por inquérito telefônico. (http://bvsms.saude.gov.br/bvs/publicacoes/vigitel_brasil_2013.pdf. Accessed: 06/08/2019).

Region	Country	Sources of alcohol patterns of consumption data by WHO Member State
		Brasil, Ministério da Saúde, Secretaria de Vigilância em Saúde, Departamento de Vigilância de Doenças e Agravos não Transmissíveis e Promoção da Saúde (2015). Vigitel Brasil 2014: vigilância de fatores de risco e proteção para doenças crônicas por inquérito telefônico. (https://bvsms.saude.gov.br/bvs/publicacoes/vigitel_brasil_2014.pdf. Accessed: 06/08/2019).
		Brasil, Ministério da Saúde. Secretaria de Vigilância em Saúde, Departamento de Vigilância de Doenças e Agravos não Transmissíveis e Promoção da Saúde (2016). Vigitel Brasil 2015: vigilância de fatores de risco e proteção para doenças crônicas por inquérito telefônico: estimativas sobre frequência e distribuição sociodemográfica de de fatores de risco e proteção para doenças crônicas nas capitais dos 26 estados brasileiros e no Distrito Federal em 2015. (https://bvsms.saude.gov.br/bvs/publicacoes/vigitel_brasil_2015.pdf. Accessed: 06/08/2019).
		Brasil, Ministério da Saúde, Secretaria de Vigilância em Saúde, Departamento de Vigilância de Doenças e Agravos não Transmissíveis e Promoção da Saúde (2017). Vigitel Brasil 2016: vigilância de fatores de risco e proteção para doenças crônicas por inquérito telefônico: estimativas sobre frequência e distribuição sociodemográfica de fatores de risco e proteção para doenças crônicas nas capitais dos 26 estados brasileiros e no Distrito Federal em 2016. (https://portalarquivos2.saude.gov.br/images/pdf/2018/marco/02/vigitel-brasil-2016.pdf. Accessed: 06/08/2019).C183
		Brasil, Ministério da Saúde. Secretaria de Vigilância em Saúde, Departamento de Vigilância de Doenças e Agravos não Transmissíveis e Promoção da Saúde (2018). Vigitel Brasil 2017: vigilância de fatores de risco e proteção para doenças crônicas por inquérito telefônico: estimativas sobre frequência e distribuição sociodemográfica de fatores de risco e proteção para doenças crônicas nas capitais dos 26 estados brasileiros e no Distrito Federal em 2017. (https://bvsms.saude.gov.br/bvs/publicacoes/vigitel_brasil_2017_vigilancia_fatores_riscos.pdf. Accessed: 06/08/2019).
		Brasil, Ministério da Saúde, Secretaria de Vigilância em Saúde, Departamento de Análise em Saúde e Vigilância de Doenças não Transmissíveis (2019). Vigitel Brasil 2018: vigilância de fatores de risco e proteção para doenças crônicas por inquérito telefônico: estimativas sobre frequência e distribuição sociodemográfica de fatores de risco e proteção para doenças crônicas nas capitais dos 26 estados brasileiros e no Distrito Federal em 2018. (https://portalarquivos2.saude.gov.br/images/pdf/2019/julho/25/vigitel-brasil-2018.pdf. Accessed: 06/08/2019).
		Coutinho ES, França-San+C187tos D, Magliano ED, Bloch KV, Barufaldi LA, Cunha CD, et al. (2016). ERICA: patterns of alcohol consumption in Brazilian adolescents, Revista de saude publica.50:8s. doi:10.1590/S01518-8787.2016050006684.
		Instituto Brasileiro de Geografia e Estatística - IBGE (2013). National health survey 2013 - Perception of health status, lifestyles and chronic diseases Brazil, Major Regions and Federation Units. (http://www.ibge.gov.br/home/estatistica/populacao/pns/2013/. Accessed: 06/23/2017).
		Instituto Brasileiro de Geografia e Estatística - IBGE (2016). Pesquisa nacional de saúde do escolar: 2015. Rio de Janeiro, Brasil: IBGE.
		Laranjeira R (2012). Brazilian National Alcohol and Drugs Survey. (http://inpad.org.br/lenad/in-english/mainresults/. Accessed: 06.23.2017).
		Macinko J, Mullachery P, Jimenez G, Neto OLM (2015). Patterns of alcohol consumption and related behaviors in Brazil: evidence from the 2013 National Health Survey (PNS 2013), PLos ONE.10(7): e0134153. https://doi.org/10.1371/journal.pone.0134153.
		Malta DC, Machado IE, Porto DL, Silva MM, Freitas PC, Costa AW, et al. (2014). Alcohol consumption among Brazilian Adolescents according to the National Adolescent School-based Health Survey (PeNSE 2012). Revista Brasileira de Epidemiologia.17(Suppl 1):203-214. https://doi.org/10.1590/1809-4503201400050016.
		The International GENACIS-Project (2002). GENACIS - gender, alcohol and culture: an international study. (http://www.genacis.org/. Accessed: 01/11/2018).
AMR	Canada	Health Canada (2012). Canadian Alcohol and Drug Use Monitoring Survey: Summary of 2011-2012 results. (https://www.canada.ca/en/health-canada/services/health-concerns/drug-prevention-treatment/drug-alcohol-use-statistics/canadian-alcohol-drug-use-monitoring-survey-summary-results-tables-2012.html#t6. Accessed 11/13/2020).
		Health Canada (2013b). Canadian Tobacco, Alcohol and Drugs Survey (CTADS): 2013 Summary. (http://healthycanadians.gc.ca/science-research-sciences-recherches/data-donnees/ctads-ectad/summary-sommaire-2013-eng.php. Accessed: 06/28/2017).
		Health Canada (2015). Canadian Tobacco, Alcohol and Drugs Survey (CTADS): 2015 Summary. (https://www.canada.ca/en/health-canada/services/canadian-tobacco-alcohol-drugs-survey/2015-summary.html. Accessed: 07/17/17).
		Health Canada (2017). Canadian Tobacco, Alcohol and Drugs (CTADS) Survey: 2017 detailed tables. (https://www.canada.ca/en/health-canada/services/canadian-tobacco-alcohol-drugs-survey/2017-summary/2017-detailed-tables.html#t17. Accessed 10/28/2020).
		Inchley J, Currie D, Budisavljevic S, Torsheim T, Jåstad A, Cosma A, et al. (2020). Spotlight on adolescent health and well-being. Findings from the 2017/2018 Health Behaviour in School-aged Children (HBSC) survey in Europe and Canada. International report. Volume 2. Key data. Copenhagen, Denmark: WHO Regional Office for Europe.
		NWT Bureau of Statistics, Statistics Canada (2015). Health Behaviour Indicators 2014, Aged 15 and Over (Smoking, Alcohol Use & Physical Activity). (https://www.statsnwt.ca/health/alcohol-drug-use/. Accessed: 06/16/2020).
		NWT Bureau of Statistics (2018). 2018 Tobacco, Alcohol & Drug Survey. (https://www.statsnwt.ca/health/alcohol-drug-use/. Accessed: 12/02/2020).
		Statistics Canada (2016). Table 13-10-0451-01 Health indicators, annual estimates, 2003 - 2014. (https://doi.org/10.25318/1310045101-eng. Accessed: 11/11/2020).
		Statistics Canada (2020). Table 13-10-0096-01 Health characteristics, annual estimates. (https://doi.org/10.25318/1310009601-eng. Accessed: 10/20/2020).
		The International GENACIS-Project (2004). GENACIS - gender, alcohol and culture: an international study. (http://www.genacis.org/. Accessed: 01/10/2018).
AMR	Chile	Greenfield TK, Bloomfield K, Wilsnack SC (2013a). GENAHTO Project (Gender and Alcohol's Harm to Others). (http://genahto.org/. Accessed: 07/02/2020).
		Instituto Nacional De Estadística & Ministerio De Sanidad Servicios Sociales E Igualdad (2012). National health survey 2011-2012. Chile: Insituto Nacional de Estadística & Ministerio de Sanidad Servicios Sociales e Igualdad.

Region	Country	Sources of alcohol patterns of consumption data by WHO Member State
		Mason-Jones AJ, Cabieses B (2015). Alcohol, binge drinking and associated mental health problems in young urban Chileans, PloS one.10(4):e0121116. https://dx.doi.org/10.1371/journal.pone.0121116.
		Ministerio de Desarrollo Social y Familia (2012). Septima Encuesta Nacional de Juventud. (http://www.injuv.gob.cl/storage/docs/7ma_ENJ_2012%283%29.pdf. Accessed: 08/16/2019).
		Ministerio de Desarrollo Social y Familia (2015). Octava Encuesta Nacional de Juventud. (http://www.injuv.gob.cl/storage/docs/Libro_Octava_Encuesta_Nacional_de_Juventud.pdf. Accessed: 08/16/2019).
		Observatorio Chileno De Drogas (2010). Noveno estudio nacional de drogas en población general de Chile. (https://www.senda.gob.cl/wp-content/uploads/2019/07/2010_Noveno_EstudioDrogasGeneral.pdf. Accessed: 08/16/2019).
		Observatorio Chileno De Drogas (2012). Décimo estudio nacional de drogas en población General de Chile. (https://www.senda.gob.cl/wp-content/uploads/2011/04/2012_Decimo_EstudioNacional.pdf. Accessed: 08/16/2019).
		Observatorio Chileno De Drogas (2013). Décimo estudio nacional de drogas en población Escolar de Chile. (http://www.senda.gob.cl/media/estudios/PE/DECIMO%20ESTUDIO%20NACIONAL%20ESCOLARES_2013.pdf. Accessed: 06/23/2017).
		Observatorio Chileno De Drogas (2014). Décimo primer estudio nacional de drogas en población General de Chile. (https://www.senda.gob.cl/wp-content/uploads/2019/07/2014_EstudioDrogas_Poblacion_General.pdf. Accessed: 08/16/2019).
		Observatorio Chileno De Drogas (2016). Décimo segundo estudio nacional de drogas en población General de Chile. (https://www.senda.gob.cl/wp-content/uploads/media/estudios/PG/2016_Estudio_Drogas_Poblacion_General.pdf. Accessed: 08/16/2019).
		Pena S, Makela P, Valdivia G, Helakorpi S, Markkula N, Margozzini P, et al. (2017). Socioeconomic inequalities in alcohol consumption in Chile and Finland, Drug and alcohol dependence.173:24-30. https://dx.doi.org/10.1016/j.drugalcdep.2016.12.014.
		World Health Organization, Centers for Disease Control and Prevention (2013). Global School-based Student Health Survey – Chile 2013 Fact Sheet. (https://www.who.int/ncds/surveillance/gshs/2013_Chile_GSHS_fact_sheet.pdf. Accessed: 06/22/2020).
AMR	Colombia	Gobierno Nacional de la República de Colombia (2011). Estudio Nacional de Consumo de Sustancias Psicoactivas en Población Escolar. (https://www.unodc.org/documents/colombia/2013/septiembre/Estudio_Poblacion_Escolar_2011.pdf. Accessed: 08/16/2019).
		Ministerio De Justicia Y Del Derecho (2014). Estudio Nacional de Consumo de Sustancias Psicoativas en Colombia 2013. (https://www.unodc.org/documents/colombia/2014/Julio/Estudio_de_Consumo_UNODC.pdf. Accessed: 06/23/2017).
		Ministerio de Salud (2015). Encuesta Nacional de Salud Mental. (http://www.odc.gov.co/Portals/1/publicaciones/pdf/consumo/estudios/nacionales/CO031102015-salud_mental_tomoI.pdf. Accessed: 08/16/2019).
		World Health Organization (2010). STEPwise approach to surveillance (STEPS) Survey. (http://www.who.int/chp/steps/en/. Accessed: 07/02/2020).
AMR	Costa Rica	Instituto sobre Alcoholismo y Farmacodependencia (2011). Juventud escolarizada y drogas. Encuesta nacional sobre consumo de drogas en población de educación secundaria, Costa Rica 2009. (https://www.iafa.go.cr/investigaciones/encuesta-nacional-sobre-consumo-de-drogas-en-poblacion-de-educacion-secundaria-costa-rica-2009. Accessed: 08/16/2019).
		Instituto sobre Alcoholismo y Farmacodependencia (2012). Consumo de drogas en Costa Rica. Encuesta Nacional 2010: Consumo de bebidas alcohólicas. (http://www.icd.go.cr/portalicd/images/docs/uid/investigaciones/EncuestaNac2010/Fascculo%202.%20Consumo%20de%20Alcohol.pdf. Accessed: 08/16/2019).
		Instituto sobre Alcoholismo y Farmacodependencia (2013). Juventud escolarizada y drogas. Encuesta nacional sobre consumo de drogas en población de educación secundaria, Costa Rica 2012. (https://www.bvs.sa.cr/tabaquismo/encuestaescolares.pdf. Accessed: 08/16/2019).
		Instituto sobre Alcoholismo y Farmacodependencia (2018). VI encuesta nacional 2015: Consumo de drogas en Costa Rica. (https://www.iafa.go.cr/investigaciones/540-vi-encuesta-nacional-2015-consumo-de-drogas-en-costa-rica). Accessed: 08/16/2019).
		Sojo C, FLACSO Costa Rica (2016). Alcohol Drinking Patterns in Latin America. Comparative Analysis of Nine Countries. (http://unpan1.un.org/intradoc/groups/public/documents/icap/unpan049539.pdf. Accessed: 06/15/2017).
		The International GENACIS-Project (2003). GENACIS - gender, alcohol and culture: an international study. (http://www.genacis.org/. Accessed: 01/10/2018).
		Unidad de Servicios Estadísticos de la Escuela de Estadística de la Universidad de Costa Rica (2012) Encuesta sobre patrones de consumo de bebidas alcohólicas en Costa Rica. (http://educalcoholcr.org/files/program/33_patronesdeconsumodebebidasalcoholicasencostaricaeducalcoholucr.pdf. Accessed: 08/16/2019).
AMR	Cuba	Bonet M, Varona P (2014). III Encuesta nacional de factores de riesgo y actividades preventivas de enfermedades no trasmisibles. Cuba 2010-2011. Editorial Ciencias Médicas. (https://www.researchgate.net/publication/325370475_III_Encuesta_Nacional_de_factores_de_riesgo_y_actividades_preventivas_de_enfermedades_no_trasmisibles_Cuba_2010-2011. Accessed: 08/20/2019).
		Dirección de Registros Médicos y Estadísticas de Salud, Ministerio de Salud Pública (2015). Encuesta de Indicadores Múltiples por Conglomerados. Cuba, 2014. Informe final. La Habana, Cuba. (https://www.unicef.org/cuba/sites/unicef.org.cuba/files/2020-04/MICS5%20encuesta-de-indicadores-multiples%202014_2015.pdf. Accessed: 08/20/2019).
		Varona P, Chang M, Garcia RG, Bonet M (2011). Tobacco and alcohol use in Cuban women, MEDICC Review.13(4):38-44.
AMR	Dominica	Inter-American Drug Abuse Control Commission (2016). A report on students' drug use in 13 Caribbean Countries : Antigua and Barbuda, The Bahamas , Barbados, Belize, Dominica, Grenada, Guyana, Haiti, Jamaica, St. Kitts and Nevis, St. Lucia, St. Vincent and the Grenadines, Trinidad and Tobago. (http://www.cicad.oas.org/Main/Template.asp?File=/oid/pub_eng.asp. Accessed: 06/17/2020).

Region	Country	Sources of alcohol patterns of consumption data by WHO Member State
		World Health Organization (2007). STEPwise approach to surveillance (STEPS) Survey. (http://www.who.int/chp/steps/en/. Accessed: 01/11/2018).<br>World Health Organization, Centers for Disease Control and Prevention (2009). Global School-based Student Health Survey – Dominica 2009 Fact Sheet. (http://www.who.int/chp/gshs/Dominica_2009_FS.pdf. Accessed: 07/18/17).
AMR	Dominican Republic	Centro De Estudios Sociales Y Demográficos (2014). Encuesta Demográfica y de Salud 2013. Santo Domingo, República Dominicana: CESDEM, ICF International.<br>Oficina Nacional De Estadística Y Unicef (2016). Encuesta Nacional de Hogares de Propósitos Múltiples - Encuesta de Indicadores Múltiples por Conglomerados 2014, Informe Final. Santo Domingo, República Dominicana: Oficina Nacional de Estadística.<br>Sojo C, FLACSO Costa Rica (2016). Alcohol Drinking Patterns in Latin America. Comparative Analysis of Nine Countries. (http://unpan1.un.org/intradoc/groups/public/documents/icap/unpan049539.pdf. Accessed: 06/15/2017).<br>World Health Organization, Centres for Disease Control and Prevention (2020). Global school based student health survey - Dominican Republic 2016 Fact Sheet. (https://extranet.who.int/ncdsmicrodata/index.php/catalog/673. Accessed: 06/15/2020)
AMR	Ecuador	Freire WB, RamíRez MJ, Belmont P, Mendieta MJ, Silva MK, Romero N, et al. (2013). RESUMEN EJECUTIVO. TOMO I. Encuesta Nacional de Salud y Nutrición del Ecuador. ENSANUT-ECU 2011-2013. Quita, Ecuador: Ministerio de Salud Pública/Instituto Nacional de Estadística y Censos.<br>Inter-American Drug Abuse Control Commission (2015). Report on Drug Use in the Americas 2015. Washington, D.C.: Organization of American States.<br>Ocana-Gordillo D, Kliewer W (2019). Risk and protective factors for heroin use in a nationally representative sample of Ecuadorian youth, Drug and Alcohol Dependence.204:107575.http://dx.doi.org/10.1016/j.drugalcdep.2019.107575.<br>World Health Organization (2018). STEPwise approach to surveillance (STEPS) Survey. (https://extranet.who.int/ncdsmicrodata/index.php/catalog/STEPS. Accessed: 07/02/2020).
AMR	El Salvador	Fondo Solidario Para La Salud, Ministerio De Economía (2014). Encuesta Nacional de alcohol y tabaco. San Salvador, El Salvador: Fondo Solidario para la Salud.<br>Gobierno de El Salvador (2014). Estudio nacional sobre el consumo de drogas en población general de El Salvador. (https://www.seguridad.gob.sv/cna/?p=1114. Accessed: 08/22/2019).<br>Gobierno de El Salvador (2017). Estudio nacional sobre el consumo de sustancias psicoactivas en población escolar de El Salvador, 2016. (http://ins.salud.gob.sv/wp-content/uploads/2018/06/Encuesta-Nacional-de-Drogas-2016.pdf. Accessed: 08/22/2019).<br>Gobierno de El Salvador (2019). Cuarta encuesta nacional sobre el consumo de sustancias psicoactivas en población escolar de El Salvador, 2018. (http://www.cicad.oas.org/oid/pubs/Cuarta%20encuesta%20sobre%20consumo%20drogas%20EL%20SALVADOR%202018%20(ISBN)%20(003).pdf. Accessed: 06/28/2019).<br>Ministerio de Salud, Instituto Nacional de Salud, Fondo de las Naciones Unidas para la Infancia (2014). Encuesta nacional de salud de Indicadores Múltiples por Conglomerados - ENS 2014. (https://mics-surveys-prod.s3.amazonaws.com/MICS5/Latin%20America%20and%20Caribbean/El%20Salvador/2014/Final/El%20Salvador%202014%20MICS%20Final%20Report_Spanish.PDF. Accessed: 08/22/2019).<br>Sojo C, FLACSO Costa Rica (2016). Alcohol Drinking Patterns in Latin America. Comparative Analysis of Nine Countries. (http://unpan1.un.org/intradoc/groups/public/documents/icap/unpan049539.pdf. Accessed: 06/15/2017).
AMR	Grenada	Inter-American Drug Abuse Control Commission (2016). A report on students' drug use in 13 Caribbean Countries : Antigua and Barbuda, The Bahamas , Barbados, Belize, Dominica, Grenada, Guyana, Haiti, Jamaica, St. Kitts and Nevis, St. Lucia, St. Vincent and the Grenadines, Trinidad and Tobago. (http://www.cicad.oas.org/Main/Template.asp?File=/oid/pub_eng.asp. Accessed: 06/17/2020).<br>World Health Organization (2010). STEPwise approach to surveillance (STEPS) Survey. (http://www.who.int/chp/steps/en/. Accessed: 01/12/2018).
AMR	Guatemala	Ministerio de Salud Pública y Asistencia Social (MSPAS), Instituto Nacional de Estadística (INE), ICF International (2017). Encuesta Nacional de Salud Materno Infantil 2014-2015. Informe Final. Guatemala: MSPAS, INE, ICF.<br>World Health Organization (2003b). World Health Survey 2003 - National Report Guatemala. Geneva, Switzerland: World Health Organization.
AMR	Guyana	Bureau of Statistics, Ministry of Public Health, United Nations Children's Fund (2015). Guyana Multiple Indicator Cluster Survey 2014, Final Report. Georgetown, Guyana: Bureau of Statistics, Ministry of Public Health, UNICEF.<br>Inter-American Drug Abuse Control Commission (2016). A report on students' drug use in 13 Caribbean Countries : Antigua and Barbuda, The Bahamas , Barbados, Belize, Dominica, Grenada, Guyana, Haiti, Jamaica, St. Kitts and Nevis, St. Lucia, St. Vincent and the Grenadines, Trinidad and Tobago. (http://www.cicad.oas.org/Main/Template.asp?File=/oid/pub_eng.asp. Accessed: 06/17/2020).<br>Ministry of Public Security (2016). Guyana National Household drug prevalence survey report 2016. (http://nana.gov.gy/Guyana-Household-Drug-Survey-Report-2016.pdf. Accessed: 08/14/2020).<br>World Health Organization, Centers for Disease Control and Prevention (2010). Global School-based Student Health Survey – Guyana 2010 Fact Sheet. (http://www.who.int/chp/gshs/Guyana_2010_GSHS_FS.pdf. Accessed: 07/18/2017).
AMR	Haiti	Inter-American Drug Abuse Control Commission (2016). A report on students' drug use in 13 Caribbean Countries : Antigua and Barbuda, The Bahamas , Barbados, Belize, Dominica, Grenada, Guyana, Haiti, Jamaica, St. Kitts and Nevis, St. Lucia, St. Vincent and the Grenadines, Trinidad and Tobago. (http://www.cicad.oas.org/Main/Template.asp?File=/oid/pub_eng.asp. Accessed: 06/17/2020).<br>Organization of American States, Inter-American Drug Abuse Control Commission (2010a). Comparative analysis of student drug use in Caribbean countries. (http://www.cicad.oas.org/Main/pubs/StudentDrugUse-Caribbean2011.pdf. Accessed: 06/28/2017).
AMR	Honduras	Sojo C, FLACSO Costa Rica (2016). Alcohol Drinking Patterns in Latin America. Comparative Analysis of Nine Countries. (http://unpan1.un.org/intradoc/groups/public/documents/icap/unpan049539.pdf. Accessed: 06/15/2017).
AMR	Jamaica	Inter-American Drug Abuse Control Commission (2016). A report on students' drug use in 13 Caribbean Countries : Antigua and Barbuda, The Bahamas , Barbados, Belize, Dominica, Grenada, Guyana, Haiti, Jamaica, St. Kitts and Nevis, St. Lucia,

Region	Country	Sources of alcohol patterns of consumption data by WHO Member State
		St. Vincent and the Grenadines, Trinidad and Tobago. (http://www.cicad.oas.org/Main/Template.asp?File=/oid/pub_eng.asp. Accessed: 06/17/2020).
		Ministry of Health (2018). Jamaica Health and Lifestyle Survey III (2016-2017) - Preliminary Key Findings. (https://www.moh.gov.jm/wp-content/uploads/2018/09/Jamaica-Health-and-Lifestyle-Survey-III-2016-2017.pdf. Accessed: 06/18/2020).
		National Council on Drug Abuse (2014). National Secondary School Survey 2013 - Fact Sheet. (http://ncda.org.jm/images/pdf/nss_2014_fact%20sheet.pdf. Accessed: 07/19/2017).
		Wilks R, Younger N, Tulloch-Reid M, McFarlane S, Francis D (2008). Jamaica Health and Lifestyle Survey 2007-8: Technical Report. Jamaica: National Health Fund.
		World Health Organization, Centers for Disease Control and Prevention (2017). Global School-based Student Health Survey – Jamaica 2017 Fact Sheet. (https://www.who.int/ncds/surveillance/gshs/Jamaica_2017_GSHS_FS.pdf?ua=1&ua=1. Accessed: 08/13/2020).
		Younger-Coleman N, Cumberbatch C, Campbell J, Ebanks C, Williams D, O'Meally V (2017). Jamaica National Drug Use Prevalence Survey 2016. (http://www.cicad.oas.org/oid/pubs/JamaicaNationalHouseholdDrugSurvey2017ENG.pdf. Accessed: 08/14/2019).
AMR	Mexico	Gobierno del Estado de México (2009). Encuesta del Estado de México sobre consumo de Alcohol, Tabaco y Drogas en Estudiantes, 2009. (https://salud.edomex.gob.mx/imca/documentos/difusion/encuestas/estatales/enestudiantes_2009.pdf. Accessed: 10/08/2019).
		Insituto Nacional De Psiquiatria Ramon De La Fuente Muniz, Instituto Nacional De Salud Pública, Comision Nacional Contra Las Adicciones & Secretaria De Salud (2017). Encuesta Nacional de Consumo de Drogas, Alcohol y Tabaco 2016-2017: Reporte de Alcohol. (https://www.gob.mx/salud%7Cconadic/acciones-y-programas/encuesta-nacional-de-consumo-de-drogas-alcohol-y-tabaco-encodat-2016-2017-136758. Accessed: 01/16/2018).
		Instituto Nacional de Salúd Publica (2014). Study on global AGEing and adult health (SAGE) Wave 1: Mexico National Report. (https://apps.who.int/healthinfo/systems/surveydata/index.php/catalog/67/download/2041. Accessed: 08/11/2020).
		Medina Mora ME, Villatoro-Velázquez JA, Fleiz-Bautista C, Tellez Rojo MM, Mendoza-Alvarado LR, Romero-Martínez M, et al. (2012). Encuesta Nacional de Adicciones 2011: Reporte de Alcohol. México: Instituto Nacional de Psiquiatría Ramón de la Fuente Muñiz & Instituto Nacional de Salud Pública; Secretaría de Salud.
		The International GENACIS-Project (1998). GENACIS - gender, alcohol and culture: an international study. (http://www.genacis.org/. Accessed: 01/18/2018).
AMR	Nicaragua	FLACSO Costa Rica (2015). EPCA Nicaragua 2015 - Población 18 a 65 años. (http://www.FLACSO.or.cr/images/epca/epca-nicaragua-adultos-2015.pdf. Accessed: 06/26/2017).
		Sojo C, FLACSO Costa Rica (2016). Alcohol Drinking Patterns in Latin America. Comparative Analysis of Nine Countries. (http://unpan1.un.org/intradoc/groups/public/documents/icap/unpan049539.pdf. Accessed: 06/15/2017).
		The International GENACIS-Project (2005). GENACIS - gender, alcohol and culture: an international study. (http://www.genacis.org/. Accessed: 01/10/2018).
AMR	Panama	Instituto Conmemorativo Gorgas de Estudios de la Salud (2021). Sistema de información de la Encuesta Nacional de Salud de Panamá (ENSPA) 2019-2021. (http://gorgas.gob.pa/SIGENSPA/Inicio.htm. Accessed: 18/02/2021).
		Organization of American States, Inter-American Drug Abuse Control Commission (2010b). Panama: Evaluation of Progress in Drug Control 2007-2009. Washington, D.C.: Organization of American States.
		Organization of American States, Inter-American Drug Abuse Control Commission (2011). Report on Drug Use in the Americas 2011. Washington, DC: Organization of American States, Inter-American Drug Abuse Control Commission.
		Secretario Ejecutivo Nacional de la CONAPRED y Observatorio Panameño de Drogas (2016). Segunda Encuesta Nacional de Hogares sobre Consumo de Drogas. Panamá 2015. (https://ministeriopublico.gob.pa/wp-content/uploads/2017/09/Encuesta-de-Hogares-2015-16-viii-17-publicaci%C3%B3n-digital.pdf. Accessed: 10/18/2019).
		Sojo C, FLACSO Costa Rica (2016). Alcohol Drinking Patterns in Latin America. Comparative Analysis of Nine Countries. (http://unpan1.un.org/intradoc/groups/public/documents/icap/unpan049539.pdf. Accessed: 06/15/2017).
AMR	Paraguay	Dirección General de Estadística, Encuestas y Censos (2016). Encuesta de indicadores múltiples por conglomerados. MICS Paraguay. (https://www.dgeec.gov.py/Publicaciones/Biblioteca/mics2016/Encuesta%20MICS%20Paraguay%202016.pdf. Acccessed: 10/23/2019).
		Organization of American States, Inter-American Drug Abuse Control Commission (2006). Paraguay, Evaluation of Progress in Drug Control 2005-2006. Washington, DC: Organization of American States.
		Organization of American States, Inter-American Drug Abuse Control Commission (2011). Report on Drug Use in the Americas 2011. Washington, DC: Organization of American States & Inter-American Drug Abuse Control Commission.
		Secretaría Nacional de la Juventud del Paraguay (2018). Diagnóstico del Perfil Socioeconómico y Expectativas de la Juventud Paraguaya. (https://www.juventud.gov.py/archivos/documentos/FINALCONACYT_mlm83l7y.pdf. 10/23/2019).
		Secretaría Técnica de Planificación del Desarrollo Económico y Social (2018). Encuesta de Salud, Bienestar y Envejecimiento. Asunción. Paraguay. (https://www.stp.gov.py/v1/wp-content/uploads/2019/04/Final_SABE-ASUNCION-REPORT_v2-1eng_spa.pdf. 10/23/2019).
		World Health Organization, Centers for Disease Control and Prevention (2017). Global School-based Student Health Survey – Paraguay 2017 Fact Sheet. (https://www.who.int/ncds/surveillance/gshs/PYH2017_fact_sheet.pdf. Accessed: 07/31/2020).
AMR	Peru	Inter-American Drug Abuse Control Commission, Secretariat for Multidimensional Security (2010). Peru - Evaluation of Progress in Drug Control 2007-2009. Peru: Organization of American States.
		Instituto Nacional de Estadística e Informática (2013). Perú: Encuesta Demográfica y de Salud Familiar - ENDES 2013. (https://www.inei.gob.pe/media/MenuRecursivo/publicaciones_digitales/Est/Lib1151/index.html. Accessed: 10/23/2019).
		Instituto Nacional de Estadística e Informática (2014). Perú: Encuesta Demográfica y de Salud Familiar - ENDES 2014. (https://www.inei.gob.pe/media/MenuRecursivo/publicaciones_digitales/Est/Lib1211/index.html. Accessed: 10/23/2019).
		Instituto Nacional de Estadística e Informática (2015). Perú: Encuesta Demográfica y de Salud Familiar - ENDES 2015. (https://www.inei.gob.pe/media/MenuRecursivo/publicaciones_digitales/Est/Lib1356/. Accessed: 10/23/2019).
		Instituto Nacional de Estadística e Informática (2016). Perú: Encuesta Demográfica y de Salud Familiar - ENDES 2016. (https://www.inei.gob.pe/media/MenuRecursivo/publicaciones_digitales/Est/Lib1433/index.html. Accessed: 10/23/2019).

Region	Country	Sources of alcohol patterns of consumption data by WHO Member State
		Instituto Nacional de Estadística e Informática (2017). Perú: Encuesta Demográfica y de Salud Familiar - ENDES 2017. (https://www.inei.gob.pe/media/MenuRecursivo/publicaciones_digitales/Est/Lib1525/index.html. Accessed: 10/23/2019).
		Instituto Nacional de Estadística e Informática (2018). Perú: Encuesta Demográfica y de Salud Familiar - ENDES 2018. (https://www.inei.gob.pe/media/MenuRecursivo/publicaciones_digitales/Est/Lib1656/index1.html. Accessed: 10/23/2019).
		Sojo C, FLACSO Costa Rica (2016). Alcohol Drinking Patterns in Latin America. Comparative Analysis of Nine Countries. (http://unpan1.un.org/intradoc/groups/public/documents/icap/unpan049539.pdf. Accessed: 06/15/2017).
		The International GENACIS-Project (2005). GENACIS - gender, alcohol and culture: an international study. (http://www.genacis.org/. Accessed: 01/10/2018).
AMR	Saint Kitts and Nevis	Inter-American Drug Abuse Control Commission (2016). A report on students' drug use in 13 Caribbean Countries : Antigua and Barbuda, The Bahamas , Barbados, Belize, Dominica, Grenada, Guyana, Haiti, Jamaica, St. Kitts and Nevis, St. Lucia, St. Vincent and the Grenadines, Trinidad and Tobago. (http://www.cicad.oas.org/Main/Template.asp?File=/oid/pub_eng.asp. Accessed: 06/17/2020).
		World Health Organization (2007). STEPwise approach to surveillance (STEPS) Survey. (http://www.who.int/chp/steps/en/. Accessed: 01/11/2018).
AMR	Saint Lucia	Inter-American Drug Abuse Control Commission (2016). A report on students' drug use in 13 Caribbean Countries : Antigua and Barbuda, The Bahamas , Barbados, Belize, Dominica, Grenada, Guyana, Haiti, Jamaica, St. Kitts and Nevis, St. Lucia, St. Vincent and the Grenadines, Trinidad and Tobago. (http://www.cicad.oas.org/Main/Template.asp?File=/oid/pub_eng.asp. Accessed: 06/17/2020).
		Ministry of Social Transformation, Local Government and Community Empowerment, Central Statistics Office (2014). Saint Lucia Multiple Indicator Cluster Survey 2012: Final Report. Castries, Saint Lucia: Government of Saint Lucia.
		World Health Organization (2012b). STEPwise approach to surveillance (STEPS) Survey. (http://www.who.int/chp/steps/en/. Accessed: 01/12/2018).
AMR	Saint Vincent and the Grenadines	Inter-American Drug Abuse Control Commission (2016). A report on students' drug use in 13 Caribbean Countries : Antigua and Barbuda, The Bahamas , Barbados, Belize, Dominica, Grenada, Guyana, Haiti, Jamaica, St. Kitts and Nevis, St. Lucia, St. Vincent and the Grenadines, Trinidad and Tobago. (http://www.cicad.oas.org/Main/Template.asp?File=/oid/pub_eng.asp. Accessed: 06/17/2020).
		Organization of American States, Inter-American Drug Abuse Control Commission (2010c). Saint Vincent and the Grenadines, Evaluation of Progress in Drug Control 2007-2009. Washington, DC: Organization of American States.
		World Health Organization (2014). STEPwise approach to surveillance (STEPS) Survey. (http://www.who.int/chp/steps/en/. Accessed: 07/02/2020).
AMR	Suriname	Organization of American States, Inter-American Drug Abuse Control Commission (2010d). Suriname, Evaluation of Progress in Drug Control 2007-2009. Suriname: Organization of American States & Inter-American Drug Abuse Control Commission.
		Organization of American States, Inter-American Drug Abuse Control Commission (2011). Report on Drug Use in the Americas 2011. Washington, DC: Organization of American States & Inter-American Drug Abuse Control Commission.
		Stroo E, Inter-American Drug Abuse Control Commission, Organization of America States (2015). 2013 Suriname National Household Drug Prevalence Survey. (http://www.cicad.oas.org/oid/pubs/SurinameHouseholdSurvey2013ENG.pdf. Accessed: 08/14/2020).
		World Health Organization, Centers for Disease Control and Prevention (2009). Global School-based Student Health Survey – Suriname 2009 Fact Sheet. (https://www.who.int/ncds/surveillance/gshs/Suriname_2009_FS.pdf. Accessed: 08/17/2018).
		World Health Organization, Centers for Disease Control and Prevention (2016). Global School-based Student Health Survey – Suriname 2016 Fact Sheet. (https://www.who.int/ncds/surveillance/gshs/Suriname_2016_FS_gshs.pdf?ua=1. Accessed: 08/15/2020).
AMR	Trinidad and Tobago	Inter-American Drug Abuse Control Commission (2016). A report on students' drug use in 13 Caribbean Countries : Antigua and Barbuda, The Bahamas , Barbados, Belize, Dominica, Grenada, Guyana, Haiti, Jamaica, St. Kitts and Nevis, St. Lucia, St. Vincent and the Grenadines, Trinidad and Tobago. (http://www.cicad.oas.org/Main/Template.asp?File=/oid/pub_eng.asp. Accessed: 06/17/2020).
		World Health Organization (2011b). STEPwise approach to surveillance (STEPS) Survey. (http://www.who.int/chp/steps/en/. Accessed: 01/12/2018).
		World Health Organization, Centers for Disease Control and Prevention (2011). Global School-based Student Health Survey - Trinidad and Tobago 2011 Fact Sheet. Trinidad, West Indies: Ministry of Health.
		World Health Organization, Centers for Disease Control and Prevention (2017). Global School-based Student Health Survey – Trinidad and Tobago 2017 Fact Sheet. (https://www.who.int/ncds/surveillance/gshs/Trinidad_and_Tobago__Tobago_2017_GSHS_FS.pdf?ua=1. Accessed: 07/31/2020).
AMR	United States of America	Esser MB, Sacks JJ, Sherk A, Karriker-Jaffe KJ, Greenfield TK, Pierannunzi C, et al. (2020). Distribution of Drinks Consumed by U.S. Adults by Average Daily Alcohol Consumption: A Comparison of 2 Nationwide Surveys, American journal of preventive medicine.59(5):669-677. https://doi.org/10.1016/j.amepre.2020.04.018.
		Grant BF, Dawson DA (2006).Introduction to the National Epidemiologic Survey on Alcohol and Related Conditions. Alcohol Research & Health.29:74-78.
		Grant BF, Chu A, Sigman R, Amsbary M, Kali J, Sugawara Y, et al. (2015). National Epidemiologic Survey on Alcohol and Related Conditions-III (NESARC-III) Source and Accuracy Statement. Rockville, MD: National Institute on Alcohol Abuse and Alcoholism.
		Kerr WC, Mulia N, Zemore SE (2014). U.S. trends in light, moderate, and heavy drinking episodes from 2000 to 2010, Alcoholism, clinical and experimental research.38(9):2496-501. https://dx.doi.org/10.1111/acer.12521.
		Kerr WC, Ye Y, Greenfield TK, Williams E, Lown EA, Lui CK (2016). Early Life Health, Trauma and Social Determinants of Lifetime Abstention from Alcohol, Alcohol and Alcoholism.51(5):576-83. https://dx.doi.org/10.1093/alcalc/agw041.
		National Institutes of Health, National Institute on Alcohol Abuse and Alcoholism (2010). Alcohol use and alcohol use disorders in the United States, A 3-year follow-up: Main findings from the 2004-2005 WAVE 2 national epidemiologic survey on alcohol and related conditions (NESARC). (https://pubs.niaaa.nih.gov/publications/nesarc_drm2/nesarc2drm.pdf. Accessed: 11/01/2017).

Region	Country	Sources of alcohol patterns of consumption data by WHO Member State
		Silveira ML, Green VR, Iannaccone R, Kimmel HL, Conway KP (2019). Patterns and correlates of polysubstance use among US youth aged 15-17 years: wave 1 of the Population Assessment of Tobacco and Health (PATH) Study, Addiction (Abingdon, England).114(5):907-16.
		Substance Abuse and Mental Health Services Administration (2020). Substance Abuse and Mental Health Data Archive - National Survey on Drug Use and Health (NSDUH 2010-2019 Studies) Series. (https://www.samhsa.gov/data/. Accessed: 10/30/2020).
		The International GENACIS-Project (2000). GENACIS - gender, alcohol and culture: an international study. (http://www.genacis.org/. Accessed: 01/11/2018).
		The International GENACIS-Project (2001). GENACIS - gender, alcohol and culture: an international study. (http://www.genacis.org/. Accessed: 01/11/2018).
		Wang K-S, Liu X, Wang L (2014). Associations of alcohol consumption and mental health with the prevalence of arthritis among US adults: data from the 2012 National Health Interview Survey, Rheumatology International.34(9):1241-9. https://dx.doi.org/10.1007/s00296-014-2992-4.
AMR	Uruguay	Junta Nacional de Drogas, Observatorio Uruguayo de Drogas (2012). V Encuesta Nacional de Hogares sobre Consumo de Drogas. (https://silo.tips/download/quinta-encuesta-nacional-en-hogares-sobre-consumo-de-drogas-informe-de-investiga. Accessed: 10/28/2019).
		Junta Nacional de Drogas, Observatorio Uruguayo de Drogas (2012). V Encuesta Nacional sobre Consumo de Drogas en Estudiantes de Enseñanza Media 2011. (http://www.codajic.org/sites/www.codajic.org/files/ENCUESTA%20NACIONAL%20DE%20DROGAS%20%20Uruguay.pdf. Accessed: 10/28/2019).
		Junta Nacional de Drogas, Observatorio Uruguayo de Drogas (2016). VII Encuesta Nacional sobre Consumo de Drogas en Estudiantes de Enseñanza Media 2016. (https://www.gub.uy/junta-nacional-drogas/sites/junta-nacional-drogas/files/documentos/publicaciones/20190225_JND_Encuesta_Nacional_sobre_consumo.pdf. Accessed: 10/28/2019).
		Ministerio de Salud Pública (2013) 2da Encuesta Nacional de factores de riesgo de enfermedades no transmisibles. (https://www.gub.uy/ministerio-salud-publica/sites/ministerio-salud-publica/files/documentos/publicaciones/2DA_ENCUESTA_NACIONAL_final2_digital.pdf. Accessed: 10/28/2019).
		Organization of American States, Inter-American Drug Abuse Control Commission (2010e). Uruguay, Evaluation of Progress in Drug Control 2007-2009. Washington, DC: Organization of American States.
		Organization of American States, Inter-American Drug Abuse Control Commission (2011). Report on Drug Use in the Americas 2011. Washington, DC: Organization of American States & Inter-American Drug Abuse Control Commission.
		Roballo JA, Olivera D (2016). VI Encuesta Nacional en Hogares sobre Consumo de Drogas, 2016 - Informe de investigación. (http://www.infodrogas.gub.uy/index.php?option=com_content&view=article&id=2600:presentacion-vi-encuesta-nacional-en-hogares-sobre-consumo-de-drogas&catid=31:encuestas&Itemid=65. Accessed: 07/19/2018).
		Suárez H,Ramírez J, Keuroglianü L (2019). Consume de alcohol en Uruguay – Informe especial Año 2019. (https://www.gub.uy/junta-nacional-drogas/sites/junta-nacional-drogas/files/documentos/publicaciones/Consumo_Alcohol_UY_2018.pdf. Accessed: 09/08/2020).
		The International GENACIS-Projec (2004). GENACIS - gender, alcohol and culture: an international study. (http://www.genacis.org/. Accessed: 01/10/2018).
		World Health Organization (2006c). STEPwise approach to surveillance (STEPS) Survey. (http://www.who.int/chp/steps/en/. Accessed: 01/12/2018).
		World Health Organization (2013). STEPwise approach to surveillance (STEPS) Survey. (https://extranet.who.int/ncdsmicrodata/index.php/catalog/STEPS. Accessed: 07/02/2020).
AMR	Venezuela (Bolivarian Republic of)	Sojo C, FLACSO Costa Rica (2016). Alcohol Drinking Patterns in Latin America. Comparative Analysis of Nine Countries. (http://unpan1.un.org/intradoc/groups/public/documents/icap/unpan049539.pdf. Accessed: 06/15/2017).
EMR	Afghanistan	Cottler LB, Ajinkya S, Goldberger BA, Ghani MA, Martin DM, Hu H, et al. (2014). Prevalence of drug and alcohol use in urban Afghanistan: epidemiological data from the Afghanistan national urban drug use study (ANUDUS), Lancet Global Health.2(10):e592-600.
		SGI Global, LLC (2015). Afghanistan National Drug Use Survey 2015 (https://colombo-plan.org/wp-content/uploads/2020/03/Afghanistan-National-Drug-Use-Survey-2015-compressed.pdf. Accessed: 10/09/2019).
		World Health Organization (2020b). STEPwise approach to surveillance (STEPS) Survey. (https://extranet.who.int/ncdsmicrodata/index.php/catalog/782. Accessed: 11/12/2020).
EMR	Bahrain	-
EMR	Djibouti	-
EMR	Egypt	Hamdi E, Gawad T, Khoweiled A, Sidrak AE, Amer D, Mamdouh R, et al. (2013). Lifetime prevalence of alcohol and substance use in Egypt: a community survey, Substance abuse.34(2):97-104.
		Rabie M, Shaker NM, Gaber E, El-Habiby M, Ismail D, El-Gaafary M, Lotfy A, Sabry N, Khafagy W, Muscat R. (2020). Prevalence updates of substance use among Egyptian adolescents. Middle East Current Psychiatry. 1;27(1):4.
		World Health Organization (2011b). STEPwise approach to surveillance (STEPS) Survey. (http://www.who.int/chp/steps/en/. Accessed: 01/12/2018).
		World Health Organization (2017). STEPwise approach to surveillance (STEPS) Survey. (http://www.who.int/chp/steps/en/. Accessed: 07/02/2020).
EMR	Iran	Amin-Esmaeili M, Rahimi-Movaghar A, Sharifi V, Hajebi A, Mojtabai R, Radgoodarzi R, et al. (2017). Alcohol use disorders in Iran: Prevalence, symptoms, correlates, and comorbidity, Drug and alcohol dependence.176:48-54. https://dx.doi.org/10.1016/j.drugalcdep.2017.02.018.
EMR	Iraq	Al-Hemiery N, Dabbagh R, Hashim MT, Al-Hasnawi S, Abutiheen A, Abdulghani EA, et al. (2017). Self-reported substance use in Iraq: findings from the Iraqi National Household Survey of Alcohol and Drug Use, 2014, Addiction.112(8):1470-9. https://dx.doi.org/10.1111/add.13800.
		World Health Organization, Iraq Ministry of Health (2009). Iraq Mental Health Survey 2006/7 Report. (http://applications.emro.who.int/dsaf/EMRPUB_2009_EN_1367.pdf?ua=1. Accessed: 06/16/2017).
		World Health Organization (2015). STEPwise approach to surveillance (STEPS) Survey. (https://extranet.who.int/ncdsmicrodata/index.php/catalog/STEPS. Accessed: 07/02/2020).

Region	Country	Sources of alcohol patterns of consumption data by WHO Member State
EMR	Jordan	World Health Organization (2019b). STEPwise approach to surveillance (STEPS) Survey. (http://www.who.int/chp/steps/en/. Accessed: 11/12/2020).
EMR	Kuwait	World Health Organization (2014b). STEPwise approach to surveillance (STEPS) Survey. (http://www.who.int/chp/steps/en/. Accessed: 01/12/2018).
EMR	Lebanon	Sibai AM, Hwalla N (2010). WHO STEPS Chronic Disease Risk Factor Surveillance: Data Book for Lebanon, 2009. Lebanon: American University of Beirut.
		World Health Organization (2017). STEPwise approach to surveillance (STEPS) Survey. (https://extranet.who.int/ncdsmicrodata/index.php/catalog/STEPS. Accessed: 07/02/2020).
		World Health Organization, Centers for Disease Control and Prevention (2017). Global School-based Student Health Survey – Lebanon 2017 Fact Sheet. (https://www.who.int/ncds/surveillance/gshs/Lebanon_2017_GSHS_FS.pdf?ua=1. Accessed: 06/28/2020).
EMR	Libya	World Health Organization (2009b). STEPwise approach to surveillance (STEPS) Survey. (http://www.who.int/chp/steps/en/. Accessed: 01/12/2018).
EMR	Morocco	World Health Organization (2013b). Morocco - World Health Survey 2003. (http://apps.who.int/healthinfo/systems/surveydata/index.php/catalog/91. Accessed: 06/28/2017).
		World Health Organization (2017). STEPwise approach to surveillance (STEPS) Survey. (https://extranet.who.int/ncdsmicrodata/index.php/catalog/STEPS. Accessed: 07/02/2020).
		World Health Organization, Centers for Disease Control and Prevention (2006b). Global School-based Student Health Survey – Morocco 2006 Fact Sheet. Rabat, Morocco: World Health Organization.
EMR	Oman	World Health Organization (2017). STEPwise approach to surveillance (STEPS) Survey. (https://extranet.who.int/ncdsmicrodata/index.php/catalog/STEPS. Accessed: 07/02/2020).
EMR	Pakistan	World Health Organization (2012d). Pakistan - World Health Survey 2003. (https://apps.who.int/healthinfo/systems/surveydata/index.php/catalog/99. Accessed: 06/28/2017).
EMR	Qatar	-
EMR	Saudi Arabia	Albuhairan FS, Tamim H, Al Dubayee M, Aldhukair S, Al Shehri S, Tamimi W, et al. (2015). Time for an adolescent health surveillance system in Saudi Arabia: findings from "Jeeluna", The Journal of Adolescent Health.57(3):263-269.
EMR	Somalia	-
EMR	Sudan	-
EMR	Syrian Arab Republic	-
EMR	Tunisia	Institut National de la Statistique, UNICEF (2019). Tunisie Enquête par grappes à indicateurs multiples (MICS), 2018, Rapport Final. Tunisie: le Ministère du Développement de l'Investissement et de la Coopération Internationale (MDICI).
		Ministère du Développement et de la Coopération Internationale, Institut National de la Statistique, Fonds des Nations Unies pour l'Enfance (2013). Suivi de la situation des enfants et des femmes en Tunisie- Enquête par grappes à indicateurs multiples 2011-2012, Rapport Final. (https://mics-surveys-prod.s3.amazonaws.com/MICS4/Middle%20East%20and%20North%20Africa/Tunisia/2011-2012/Final/Tunisia%202011-12%20MICS_French.pdf. Accessed: 06/26/2017).
		Saidi O, Zoghlami N, Aounallah-Skhiri H, Hsairi M, Skhiri A, Ben Mansour N, et al. (2019). La santé des Tunisiens Résultats de l'enquête "Tunisian Health Examination Survey-2016" (http://www.santetunisie.rns.tn/images/rapport-final-enquete2020.pdf. Accessed: 06/04/2020).
EMR	United Arab Emirates	Qawas A, Ahli S, Madi H, Mahagaonkar SB (2018). UAE National Health Survey Report 2017-2018. (https://www.mohap.gov.ae/Files/MOH_OpenData/1556/UAE_NHS_2018.pdf. Accessed: 06/04/2020).
		World Health Organization (2010). STEPwise approach to surveillance (STEPS) Survey. (http://www.who.int/chp/steps/en/. Accessed: 01/12/2018).
		World Health Organization (2012d). United Arab Emirates - World Health Survey 2003. (http://apps.who.int/healthinfo/systems/surveydata/index.php/catalog/128. Accessed: 01/22/2018).
EMR	Yemen	-
EUR	Albania	Inchley J, Currie D, Budisavljevic S, Torsheim T, Jåstad A, Cosma A, et al. (2020). Spotlight on adolescent health and well-being. Findings from the 2017/2018 Health Behaviour in School-aged Children (HBSC) survey in Europe and Canada. International report. Volume 2. Key data. Copenhagen, Denmark: WHO Regional Office for Europe.
		Institute of Statistics, Institute of Public Health (2018). Albania Demographic and Health Survey 2017-2018, Institute of Statistics, Institute of Public Health, and ICF. Tirana, Albania: Institute of Statistics, Institute of Public Health, ICF.
		Kraja F, Kraja B, Cakerri L, Burazeri G (2016). Socio-demographic and Lifestyle Correlates of Self-perceived Health Status in a Population-based Sample of Albanian Adult Men and Women, Materia socio-medica.28(3):173-7. https://dx.doi.org/10.5455/msm.2016.28.173-177.
		Qirjako G, Bani R, Ylli R, Burazeri G (2014). National Survey on substance use in the general population in Albania 2014. Tirana, Albania: European Monitoring Centre for Drugs and Drug Addiction.
EUR	Andorra	Ministeri de Salut I Benestar (2011). Enquesta Nacional De Salut D'Andorra 2011. Andorra: Ministeri de Salut I Benestar
EUR	Armenia	Abe S, Stickley A, Roberts B, Richardson E, Abbott P, Rotman D, et al. (2013). Changing patterns of fruit and vegetable intake in countries of the former Soviet Union, Public Health Nutrition.16(11):1924-1932.
		Inchley J, Currie D, Budisavljevic S, Torsheim T, Jåstad A, Cosma A, et al. (2020). Spotlight on adolescent health and well-being. Findings from the 2017/2018 Health Behaviour in School-aged Children (HBSC) survey in Europe and Canada. International report. Volume 2. Key data. Copenhagen, Denmark: WHO Regional Office for Europe.
		Ministry of Health, National Institute of Health (2014). National Report on Drugs. Yerevan, Armenia: Ministry of Health, National Institute of Health.
		World Health Organization (2016). STEPwise approach to surveillance (STEPS) Survey. (https://extranet.who.int/ncdsmicrodata/index.php/catalog/STEPS. Accessed: 07/02/2020).
EUR	Austria	Bloomfield K, Allamani A, Beck F, Bergmark KH, Csemy L, Eisenbach-Stangl I, et al. (2005). Gender, culture and alcohol problems : a multi-national study. An EU concerted Action. Project final report. Berlin, Germany: Charite Campus Benjamin Franklin.

Region	Country	Sources of alcohol patterns of consumption data by WHO Member State
		ESPAD Group (2020). ESPAD Report 2019: Results from the European School Survey Project on Alcohol and Other Drugs. Luxembourg: EMCDDA Joint Publications, Publications Office of the European Union.
		Inchley J, Currie D, Budisavljevic S, Torsheim T, Jåstad A, Cosma A, et al. (2020). Spotlight on adolescent health and well-being. Findings from the 2017/2018 Health Behaviour in School-aged Children (HBSC) survey in Europe and Canada. International report. Volume 2. Key data. Copenhagen, Denmark: WHO Regional Office for Europe.
		Moskalewicz J, Room R, Thom B (2016). Comparative monitoring of alcohol epidemiology across the EU: Baseline assessment and suggestions for future action. Synthesis report. Warszawa, Poland: Joint Action on Reducing Alcohol Related Harm (RARHA).
		Ramelow D, Teutsch F, Felder-Puig R, Ludwig B, Institut Health Promotion Research (2015). Gesundheit und Gesundheitsverhalten von österreichischen Schülerinnen und Schlülern - Ergebnisse des WHO-HSBC-Survey 2014. Wien: Bundesministerium für Gesundheit - Sektion III.
		Strizek J, Uhl A (2015). Bevölkerungserhebung zu Substanzgebrauch 2015 Band 1: Forschungsbericht. (http://www.praevention.at/fileadmin/user_upload/08_Sucht/Bevoelkerungserhebung_zu_Substanzgebrauch_2015.pdf. Accessed: 06/21/2017).
		Statistical Office of the European Union (Eurostat) (2019). European Health Interview Survey Wave 2 data table: Frequency of alcohol consumption by sex, age and educational attainment level. (https://ec.europa.eu/eurostat/databrowser/view/hlth_ehis_al1e/default/table?lang=en. Accessed: 08/17/2020).
		Statistical Office of the European Union (Eurostat) (2019). European Health Interview Survey Wave 2 data table: Frequency of heavy episodic drinking by sex, age and educational attainment level. (https://ec.europa.eu/eurostat/databrowser/view/hlth_ehis_al3e/default/table?lang=en. Accessed: 08/17/2020).
		The International GENACIS-Project (1993). GENACIS - gender, alcohol and culture: an international study. (http://www.genacis.org/. Accessed: 01/10/2018).
		Teutsch F, Ramelow D, Maier G, Felder-Puig R (2019). Lineartabellen zu den Ergebnissen des HBSC Berichtes 2018. Gesundheit und Gesundheitsverhalten von Schülerinnen und Schülern in Österreich. Wien, Österreich: BMASK.
EUR	Azerbaijan	Abe S, Stickley A, Roberts B, Richardson E, Abbott P, Rotman D, et al. (2013). Changing patterns of fruit and vegetable intake in countries of the former Soviet Union, Public Health Nutrition.16(11):1924-1932.
		Inchley J, Currie D, Budisavljevic S, Torsheim T, Jåstad A, Cosma A, et al. (2020). Spotlight on adolescent health and well-being. Findings from the 2017/2018 Health Behaviour in School-aged Children (HBSC) survey in Europe and Canada. International report. Volume 2. Key data. Copenhagen, Denmark: WHO Regional Office for Europe.
EUR	Belarus	Abe S, Stickley A, Roberts B, Richardson E, Abbott P, Rotman D, et al. (2013). Changing patterns of fruit and vegetable intake in countries of the former Soviet Union. Public Health Nutrition.16(11):1924-1932.
		National Statistical Committee of the Republic of Belarus, United Nations Children's Fund (UNICEF) (2013). Multiple Indicator Cluster Survey of the Situation of Children and Women in the Republic of Belarus (МИКС4), 2012. Final Report. Minsk, Belarus: National Statistical Committee of the Republic of Belarus, United Nations Children's Fund (UNICEF).
		World Health Organization (2017b). Prevalence of noncommunicable disease risk factors in Republic of Belarus. STEPS 2016. Minsk, Belarus: World Health Organization.
EUR	Belgium	European Monitoring Centre for Drugs and Drug Addiction (2020). Statistical Bulletin 2020 – prevalence of drug use: Alcohol Data tables - lifetime prevalence, last year prevalence (https://www.emcdda.europa.eu/data/stats2020/gps. Accessed: 07/21/2020).
		Gisle L (2014). La consommation d'alcool. Enquête de Santé 2013. Rapport 2: Comportements de Santé et Style de vie. Bruxelles, Belgique: Institut Scientifique de Santé Publique (WIW-ISP).
		Gisle L, Demarest S, Driekens S (2019). Enquête de santé 2018 : Consommation d'alcool. Bruxelles, Belgique: Sciensano.
		Inchley J, Currie D, Budisavljevic S, Torsheim T, Jåstad A, Cosma A, et al. (2020). Spotlight on adolescent health and well-being. Findings from the 2017/2018 Health Behaviour in School-aged Children (HBSC) survey in Europe and Canada. International report. Volume 2. Key data. Copenhagen, Denmark: WHO Regional Office for Europe.
		Statistical Office of the European Union (Eurostat) (2019). European Health Interview Survey Wave 2 data table: Frequency of alcohol consumption by sex, age and educational attainment level. (https://ec.europa.eu/eurostat/databrowser/view/hlth_ehis_al1e/default/table?lang=en. Accessed: 08/17/2020).
		Statistical Office of the European Union (Eurostat) (2019). European Health Interview Survey Wave 2 data table: Frequency of heavy episodic drinking by sex, age and educational attainment level. (https://ec.europa.eu/eurostat/databrowser/view/hlth_ehis_al3e/default/table?lang=en. Accessed: 08/17/2020).
EUR	Bosnia and Herzegovina	Agency for Statistics of Bosnia and Herzegovina, Federal Ministry of Health, Ministry of Health and Social Welfare of the Republic of Srpska, Institute for Public Health of the Federation of Bosnia and Herzegovina (2013). Bosnia and Herzegovina Multiple Indicator Cluster Survey (MICS) 2011–2012, Final Report. Sarajevo, Bosnia and Herzegovina: UNICEF.
		European Monitoring Centre for Drugs and Drug Addiction, Ipsos (2019). Substance use among the general population in Bosnia and Herzegovina in 2018. (https://www.emcdda.europa.eu/drugs-library/substance-use-among-general-population-bosnia-and-herzegovina-2018_en. Accessed: 07/21/2020)
EUR	Bulgaria	Balabanova D, Mckee M (1999). Patterns of alcohol consumption in Bulgaria, Alcohol & Alcoholism.34(4):622-628.
		ESPAD Group (2020). ESPAD Report 2019: Results from the European School Survey Project on Alcohol and Other Drugs. Luxembourg: EMCDDA Joint Publications, Publications Office of the European Union.
		European Monitoring Centre for Drugs and Drug Addiction (2016). ESPAD report 2015. Results from the European School Survey Project on Alcohol and Other Drugs. (http://www.espad.org/sites/espad.org/files/ESPAD_report_2015.pdf. Accessed: 06/15/2017)
		European Monitoring Centre for Drugs and Drug Addiction (2020). Statistical Bulletin 2020 – prevalence of drug use: Alcohol Data tables - lifetime prevalence, last year prevalence (https://www.emcdda.europa.eu/data/stats2020/gps. Accessed: 07/21/2020).
		Inchley J, Currie D, Budisavljevic S, Torsheim T, Jåstad A, Cosma A, et al. (2020). Spotlight on adolescent health and well-being. Findings from the 2017/2018 Health Behaviour in School-aged Children (HBSC) survey in Europe and Canada. International report. Volume 2. Key data. Copenhagen, Denmark: WHO Regional Office for Europe.

Region	Country	Sources of alcohol patterns of consumption data by WHO Member State
		Moskalewicz J, Room R, Thom B (2016). Comparative monitoring of alcohol epidemiology across the EU: Baseline assessment and suggestions for future action. Synthesis report. Warszawa, Poland: Joint Action on Reducing Alcohol Related Harm (RARHA).
		Statistical Office of the European Union (Eurostat) (2019). European Health Interview Survey Wave 2 data table: Frequency of alcohol consumption by sex, age and educational attainment level. (https://ec.europa.eu/eurostat/databrowser/view/hlth_ehis_al1e/default/table?lang=en. Accessed: 08/17/2020).
		Statistical Office of the European Union (Eurostat) (2019). European Health Interview Survey Wave 2 data table: Frequency of heavy episodic drinking by sex, age and educational attainment level. (https://ec.europa.eu/eurostat/databrowser/view/hlth_ehis_al3e/default/table?lang=en. Accessed: 08/17/2020).
EUR	Croatia	ESPAD Group (2020). ESPAD Report 2019: Results from the European School Survey Project on Alcohol and Other Drugs. Luxembourg: EMCDDA Joint Publications, Publications Office of the European Union.
		European Monitoring Centre for Drugs and Drug Addiction (2016). ESPAD Report 2015. Results from the European School Survey Project on Alcohol and Other Drugs. (http://www.espad.org/sites/espad.org/files/ESPAD_report_2015.pdf. Accessed: 06/15/2017)
		Glavak Tkalić, R., Miletić, G.-M., Maričić, J (2016). Uporaba sredstava ovisnosti u hrvatskom društvu: Istraživanje na općoj populaciji. Zagreb: Institut društvenih znanosti Ivo Pilar i Ured za suzbijanje zlouporabe droga Vlade Republike Hrvatske.
		Glavak Tkalić R, Miletić GM, Maričić J, Wertag A (2012). Substance Abuse among the General Population in the Republic of Croatia: Research Report. Zagreb: Institute of Social Sciences Ivo Pilar, Government of the Republic of Croatia – Office for Combating Drug Abuse.
		Inchley J, Currie D, Budisavljevic S, Torsheim T, Jåstad A, Cosma A, et al. (2020). Spotlight on adolescent health and well-being. Findings from the 2017/2018 Health Behaviour in School-aged Children (HBSC) survey in Europe and Canada. International report. Volume 2. Key data. Copenhagen, Denmark: WHO Regional Office for Europe.
		Moskalewicz J, Room R, Thom B (2016). Comparative monitoring of alcohol epidemiology across the EU: Baseline assessment and suggestions for future action. Synthesis report. Warszawa, Poland: Joint Action on Reducing Alcohol Related Harm (RARHA).
		Statistical Office of the European Union (Eurostat) (2019). European Health Interview Survey Wave 2 data table: Frequency of alcohol consumption by sex, age and educational attainment level. (https://ec.europa.eu/eurostat/databrowser/view/hlth_ehis_al1e/default/table?lang=en. Accessed: 08/17/2020).
		Statistical Office of the European Union (Eurostat) (2019). European Health Interview Survey Wave 2 data table: Frequency of heavy episodic drinking by sex, age and educational attainment level. (https://ec.europa.eu/eurostat/databrowser/view/hlth_ehis_al3e/default/table?lang=en. Accessed: 08/17/2020).
EUR	Cyprus	ESPAD Group (2020). ESPAD Report 2019: Results from the European School Survey Project on Alcohol and Other Drugs. Luxembourg: EMCDDA Joint Publications, Publications Office of the European Union.
		European Monitoring Centre for Drugs and Drug Addiction (2016). ESPAD Report 2015. Results from the European School Survey Project on Alcohol and Other Drugs. (http://www.espad.org/sites/espad.org/files/ESPAD_report_2015.pdf. Accessed: 06/15/2017).
		European Monitoring Centre for Drugs and Drug Addiction (2020). Statistical Bulletin 2020 – prevalence of drug use: Alcohol Data tables - lifetime prevalence, last year prevalence (https://www.emcdda.europa.eu/data/stats2020/gps. Accessed: 07/21/2020).
		Statistical Office of the European Union (Eurostat) (2019). European Health Interview Survey Wave 2 data table: Frequency of alcohol consumption by sex, age and educational attainment level. (https://ec.europa.eu/eurostat/databrowser/view/hlth_ehis_al1e/default/table?lang=en. Accessed: 08/17/2020).
		Statistical Office of the European Union (Eurostat) (2019). European Health Interview Survey Wave 2 data table: Frequency of heavy episodic drinking by sex, age and educational attainment level. (https://ec.europa.eu/eurostat/databrowser/view/hlth_ehis_al3e/default/table?lang=en. Accessed: 08/17/2020).
EUR	Czech Republic	Bloomfield K, Allamani A, Beck F, Bergmark KH, Csemy L, Eisenbach-Stangl I, et al. (2005). Gender, culture and alcohol problems : a multi-national study. An EU concerted Action. Project final report. Berlin, Germany: Charite Campus Benjamin Franklin.
		ESPAD Group (2020). ESPAD Report 2019: Results from the European School Survey Project on Alcohol and Other Drugs. Luxembourg: EMCDDA Joint Publications, Publications Office of the European Union.
		Csémy L, Dvořáková Z, Fialová A, Kodl M, Malý M, Skývová S (2020). Užívání tabáku a alkoholu v České republice 2019. (http://www.szu.cz/uploads/documents/szu/aktual/zprava_tabak_alkohol_cr_2019.pdf. Accessed: 11/12/2020).
		Inchley J, Currie D, Budisavljevic S, Torsheim T, Jåstad A, Cosma A, et al. (2020). Spotlight on adolescent health and well-being. Findings from the 2017/2018 Health Behaviour in School-aged Children (HBSC) survey in Europe and Canada. International report. Volume 2. Key data. Copenhagen, Denmark: WHO Regional Office for Europe.
		Mravčík V, Chomynová P, Nechanská B, Černíková T, Csémy L (2019). Alcohol use and its consequences in the Czech Republic, Central European journal of public health.27(Supplement):S15-28.
		Csémy L, Dvořáková Z, Fialová A, Kodl M, Skývová S (2019). Užívání tabáku a alkoholu v České republice 2018. (http://www.szu.cz/uploads/documents/szu/aktual/uzivani_tabaku_alkoholu_cr_2018.pdf. Accessed: 06/06/2020).
		Sovinová H, Csémy L (2013). The Use of Tobacco and Alcohol in the Czech Republic 2012. Czech Republic: National Institute of Public Health.
		Statistical Office of the European Union (Eurostat) (2019). European Health Interview Survey Wave 2 data table: Frequency of alcohol consumption by sex, age and educational attainment level. (https://ec.europa.eu/eurostat/databrowser/view/hlth_ehis_al1e/default/table?lang=en. Accessed: 08/17/2020).
		Statistical Office of the European Union (Eurostat) (2019). European Health Interview Survey Wave 2 data table: Frequency of heavy episodic drinking by sex, age and educational attainment level. (https://ec.europa.eu/eurostat/databrowser/view/hlth_ehis_al3e/default/table?lang=en. Accessed: 08/17/2020).
		The International GENACIS-Project (2002). GENACIS - gender, alcohol and culture: an international study. (http://www.genacis.org/. Accessed: 01/11/2018).

Region	Country	Sources of alcohol patterns of consumption data by WHO Member State
EUR	Denmark	Bloomfield K, Allamani A, Beck F, Bergmark KH, Csemy L, Eisenbach-Stangl I, et al. (2005). Gender, culture and alcohol problems : a multi-national study. An EU concerted Action. Project final report. Berlin, Germany: Charite Campus Benjamin Franklin.
		Bloomfield K, Grittner U, Kraus L, Piontek D (2017). Drinking patterns at the sub-national level: What do they tell us about drinking cultures in European countries?, Nordic Studies on Alcohol and Drugs.34(4):342-52. https://dx.doi.org/10.1177/1455072517712820.
		Christensen AI, Davidsen M, Ekholm P, Pedersen PV, Juel, K (2014). Danskernes Sundhed – Den Nationale Sundhedsprofil 2013. Copenhagen: Sundhedsstyrelsens publikationer - Rosendahls Distribution.
		ESPAD Group (2020). ESPAD Report 2019: Results from the European School Survey Project on Alcohol and Other Drugs. Luxembourg: EMCDDA Joint Publications, Publications Office of the European Union.
		Inchley J, Currie D, Budisavljevic S, Torsheim T, Jåstad A, Cosma A, et al. (2020). Spotlight on adolescent health and well-being. Findings from the 2017/2018 Health Behaviour in School-aged Children (HBSC) survey in Europe and Canada. International report. Volume 2. Key data. Copenhagen, Denmark: WHO Regional Office for Europe.
		Jensen HAR, Davidsen M, Ekholm O, Christensen AI. (2018). Danskernes Sundhed – Den Nationale Sundhedsprofil 2017. (https://www.sst.dk/-/media/Udgivelser/2018/Den-Nationale-Sundhedsprofil-2017.ashx?la=da&hash=421C482AEDC718D3B4846FC5E2B0EED2725AF517. Accessed: 06/20/2020).
		Moskalewicz J, Room R, Thom B (2016). Comparative monitoring of alcohol epidemiology across the EU: Baseline assessment and suggestions for future action. Synthesis report. Warszawa, Poland: Joint Action on Reducing Alcohol Related Harm (RARHA).
		Seid AK, Bloomfield K, Hesse M (2018). The relationship between socioeconomic status and risky drinking in Denmark: a cross-sectional general population study, BMC public health.18(1):743. https://dx.doi.org/10.1186/s12889-018-5481-y.
		Statistical Office of the European Union (Eurostat) (2019). European Health Interview Survey Wave 2 data table: Frequency of alcohol consumption by sex, age and educational attainment level. (https://ec.europa.eu/eurostat/databrowser/view/hlth_ehis_al1e/default/table?lang=en. Accessed: 08/17/2020).
		Statistical Office of the European Union (Eurostat) (2019). European Health Interview Survey Wave 2 data table: Frequency of heavy episodic drinking by sex, age and educational attainment level. (https://ec.europa.eu/eurostat/databrowser/view/hlth_ehis_al3e/default/table?lang=en. Accessed: 08/17/2020).
		The International GENACIS-Project (2003). GENACIS - gender, alcohol and culture: an international study. (http://www.genacis.org/. Accessed: 01/10/2018).
EUR	Estonia	ESPAD Group (2020). ESPAD Report 2019: Results from the European School Survey Project on Alcohol and Other Drugs. Luxembourg: EMCDDA Joint Publications, Publications Office of the European Union.
		Inchley J, Currie D, Budisavljevic S, Torsheim T, Jåstad A, Cosma A, et al. (2020). Spotlight on adolescent health and well-being. Findings from the 2017/2018 Health Behaviour in School-aged Children (HBSC) survey in Europe and Canada. International report. Volume 2. Key data. Copenhagen, Denmark: WHO Regional Office for Europe.
		Moskalewicz J, Room R, Thom B (2016). Comparative monitoring of alcohol epidemiology across the EU: Baseline assessment and suggestions for future action. Synthesis report. Warszawa, Poland: Joint Action on Reducing Alcohol Related Harm (RARHA).
		Reile R, Tekkel M, Veideman T (2019). Eesti täiskasvanud rahvastiku tervisekäitumise uuring, 2018. Health Behavior among Estonian Adult Population, 2018. (https://intra.tai.ee//images/prints/documents/155471416749_TKU2018_kogumik_28mar2019_1.pdf. Accessed: 07/24/2019).
		Statistical Office of the European Union (Eurostat) (2019). European Health Interview Survey Wave 2 data table: Frequency of alcohol consumption by sex, age and educational attainment level. (https://ec.europa.eu/eurostat/databrowser/view/hlth_ehis_al1e/default/table?lang=en. Accessed: 08/17/2020).
		Statistical Office of the European Union (Eurostat) (2019). European Health Interview Survey Wave 2 data table: Frequency of heavy episodic drinking by sex, age and educational attainment level. (https://ec.europa.eu/eurostat/databrowser/view/hlth_ehis_al3e/default/table?lang=en. Accessed: 08/17/2020).
		Tekkel M, Veideman T (2011). Eesti täiskasvanud rahvastiku tervisekäitumise uuring, 2010. Health Behavior among Estonian Adult Population, 2010. (https://intra.tai.ee//images/prints/documents/132091796870_Eesti_taiskasvanud_rahvastiku_tervisekaitumise_uuring_EST_ENG.pdf. Accessed: 07/30/2019).
		Tekkel M, Veideman T (2015). Eesti täiskasvanud rahvastiku tervisekäitumise uuring, 2014. Health Behavior among Estonian Adult Population, 2014. (https://intra.tai.ee//images/prints/documents/14274488161_T2iskasvanud_rahvastiku_tervisek2itumise_uuring_2014.pdf. Accessed: 01/23/2017).
		Tekkel M, Veideman T (2017). Eesti täiskasvanud rahvastiku tervisekäitumise uuring, 2016. Health Behavior among Estonian Adult Population, 2016. (https://intra.tai.ee/images/prints/documents/149069399613_Eesti_taiskasvanud_rahvastiku_tervisekaitumise_uuring_2016.pdf. Accessed: 07/24/2019).
		Vorbjob S, Salekešin M (2016). Uimastite tarvitamine koolinoorte seas: 15–16-aastaste õpilaste legaalsete ja illegaalsete narkootikumide kasutamine Eestis: Uuringu raport. Tallinn, Eesti: Tervise Arengu Instituut.
EUR	Finland	Bloomfield K, Allamani A, Beck F, Bergmark KH, Csemy L, Eisenbach-Stangl I (2005). Gender, culture and alcohol problems : a multi-national study. An EU concerted Action. Project final report. Berlin, Germany: Charite Campus Benjamin Franklin.
		ESPAD Group (2020). ESPAD Report 2019: Results from the European School Survey Project on Alcohol and Other Drugs. Luxembourg: EMCDDA Joint Publications, Publications Office of the European Union.
		European Monitoring Centre for Drugs and Drug Addiction (2020). Statistical Bulletin 2020 – prevalence of drug use: Alcohol Data tables - lifetime prevalence, last year prevalence (https://www.emcdda.europa.eu/data/stats2020/gps. Accessed: 07/21/2020).
		Härkönen J, Savonen J, Virtala E, Mäkelä P (2017). Suomalaisten alkoholinkäyttötavat 1968– 2016: Juomatapatutkimusten tuloksia. [Results from the Finnish Drinking Habits Surveys 1968–2016]. Helsinki, Finland: National Institute for Health and Welfare (THL).

Region	Country	Sources of alcohol patterns of consumption data by WHO Member State
		Helakorpi S, Anna-Leena H, Virtanen S, Uutela A (2012). Suomalaisen aikuisväestön terveyskäyttäytyminen ja terveys, kevät 2011 - Health Behaviour and Health among the Finnish Adult Population, Spring 2011. (http://urn.fi/URN:ISBN:978-952-245-566-6. Accessed: 08/28/2019).
		Helakorpi S, Pajunen T, Jallinoja, P, Virtanen S, Uutela A (2011). Suomalaisen aikuisväestön terveyskäyttäytyminen ja terveys, kevät 2010 - Health Behaviour and Health Among the Finnish Adult Population, Spring 2010. (http://urn.fi/URN:NBN:fi-fe201205085393. Accessed: 08/28/2019).
		Heldan A, Helakorpi S (2014). Suomalaisen aikuisväestön terveyskäyttäytyminen ja terveys, kevät 2014-Health Behaviour and Health among the Finnish Adult Population, Spring 2014. (http://urn.fi/URN:ISBN:978-952-302-447-2. Accessed: 08/28/2019).
		Helldán A, Helakorpi S, Virtanen S, Uutela A (2013a). Suomalaisen aikuisväestön terveyskäyttäytyminen ja terveys, kevät 2012 - Health Behavior and Health Among the Finnish Adult Population, Spring 2012. (http://urn.fi/URN:ISBN:978-952-245-931-2. Accessed: 08/28/2019).
		Helldán A, Helakorpi S, Virtanen S, Uutela A (2013b). Suomalaisen aikuisväestön terveyskäyttäytyminen ja terveys, kevät 2013 - Health Behavior and Health Among the Finnish Adult Population, Spring 2013. (http://urn.fi/URN:ISBN:978-952-302-051-1. Accessed: 08/28/2019).
		Inchley J, Currie D, Budisavljevic S, Torsheim T, Jåstad A, Cosma A, et al. (2020). Spotlight on adolescent health and well-being. Findings from the 2017/2018 Health Behaviour in School-aged Children (HBSC) survey in Europe and Canada. International report. Volume 2. Key data. Copenhagen, Denmark: WHO Regional Office for Europe.
		Kaikkonen R, Murto J, Pentala O, Koskela T, Viratala E, Härkänen T, et al. (2017). Regional health and well-being study (ATH) basic results 2010-2015. (www.thl.fi/ath. Accessed: 10/11/2017).
		Koskinen S, Lundqvist A, Ristiluoma N (2012). Terveys, toimintakysky ja hyvinvointi Suomessa 2011. Tampere: Juvenes Print - Suomen Yliopistopaino Oy.
		Moskalewicz J, Room R, Thom B (2016). Comparative monitoring of alcohol epidemiology across the EU: Baseline assessment and suggestions for future action. Synthesis report. Warszawa, Poland: Joint Action on Reducing Alcohol Related Harm (RARHA).
		Raitasalo K, Simonen J, Tigerstedt C, Mäkelä P, Tapanainen H (2018). What is going on in underage drinking? Reflections on Finnish European school survey project on alcohol and other drugs data 1999-2015, Drug and alcohol review.37(Suppl 1):76–S84. https://doi.org/10.1111/dar.12697.
		Statistical Office of the European Union (Eurostat) (2019). European Health Interview Survey Wave 2 data table: Frequency of alcohol consumption by sex, age and educational attainment level. (https://ec.europa.eu/eurostat/databrowser/view/hlth_ehis_al1e/default/table?lang=en. Accessed: 08/17/2020).
		Statistical Office of the European Union (Eurostat) (2019). European Health Interview Survey Wave 2 data table: Frequency of heavy episodic drinking by sex, age and educational attainment level. (https://ec.europa.eu/eurostat/databrowser/view/hlth_ehis_al3e/default/table?lang=en. Accessed: 08/17/2020).
		The International GENACIS-Project (2000). GENACIS - gender, alcohol and culture: an international study. (http://www.genacis.org/. Accessed: 01/11/2018).
EUR	France	Beck F, Richard J (2012). Epidémiologie de l'alcoolisation. EMC-endocrinologie-Nutrition.1-9
		Bloomfield K, Allamani A, Beck F, Bergmark KH, Csemy L, Eisenbach-Stangl I, et al. (2005). Gender, culture and alcohol problems : a multi-national study. An EU concerted Action. Project final report. Berlin, Germany: Charite Campus Benjamin Franklin.
		ESPAD Group (2020). ESPAD Report 2019: Results from the European School Survey Project on Alcohol and Other Drugs. Luxembourg: EMCDDA Joint Publications, Publications Office of the European Union.
		European Monitoring Centre for Drugs and Drug Addiction (2016). ESPAD report 2015. Results from the European School Survey Project on Alcohol and Other Drugs. (http://www.espad.org/sites/espad.org/files/ESPAD_report_2015.pdf. Accessed: 06/15/2017).
		Inchley J, Currie D, Budisavljevic S, Torsheim T, Jåstad A, Cosma A, et al. (2020). Spotlight on adolescent health and well-being. Findings from the 2017/2018 Health Behaviour in School-aged Children (HBSC) survey in Europe and Canada. International report. Volume 2. Key data. Copenhagen, Denmark: WHO Regional Office for Europe.
		Moskalewicz J, Room R, Thom B (2016). Comparative monitoring of alcohol epidemiology across the EU: Baseline assessment and suggestions for future action. Synthesis report. Warszawa, Poland: Joint Action on Reducing Alcohol Related Harm (RARHA).
		Nezet L (2015). Les drogues à 17 ans: analyse de l'enquête ESCAPAD 2014. Saint-Denis, France: Observatoire Français des Drogues et des Toxicomanies.
		Philippon A, Le Nézet O, Janssen E, Cogordan C, Andler R, Richard JB, et al (2019). Consommation et approvisionnement en alcool à 17 ans en France : résultats de l'enquête ESCAPAD 2017, Bulletin épidémiologique hebdomadaire.(5-6):109-15. http://invs.santepubliquefrance.fr/beh/2019/5-6/2019_5-6_3.html.
		Richard JB, Andler R, Cogordan C, Spilka S, Nguyen-Thanh V, le groupe Baromètre de Santé publique France 2017 (2019). La consommation d'alcool chez les adultes en France en 2017, Bulletin épidémiologique hebdomadaire.(5-6):89-97. http://invs.santepubliquefrance.fr/beh/2019/5-6/2019_5-6_1.html.
		Richard JB, Palle C, Guignard R, Nguyen Thanh V, Beck F, Arwidson P (2015). La consommation d'alcool en France en 2014. (http://inpes.santepubliquefrance.fr/CFESBases/catalogue/pdf/1632.pdf. Accessed: 07/18/2017).
		The International GENACIS-Project (1999). GENACIS - gender, alcohol and culture: an international study. (http://www.genacis.org/. Accessed: 01/11/2018).
		The International GENACIS-Project (2000). GENACIS - gender, alcohol and culture: an international study. (http://www.genacis.org/. Accessed: 01/11/2018).
EUR	Georgia	Abe S, Stickley A, Roberts B, Richardson E, Abbott P, Rotman D, et al. (2013). Changing patterns of fruit and vegetable intake in countries of the former Soviet Union. Public Health Nutrition.16(11):1924-1932.
		ESPAD Group (2020). ESPAD Report 2019: Results from the European School Survey Project on Alcohol and Other Drugs. Luxembourg: EMCDDA Joint Publications, Publications Office of the European Union.

Region	Country	Sources of alcohol patterns of consumption data by WHO Member State
		Inchley J, Currie D, Budisavljevic S, Torsheim T, Jåstad A, Cosma A, et al. (2020). Spotlight on adolescent health and well-being. Findings from the 2017/2018 Health Behaviour in School-aged Children (HBSC) survey in Europe and Canada. International report. Volume 2. Key data. Copenhagen, Denmark: WHO Regional Office for Europe.
		Kirtadze I, Otiashvili D, Tabatadze M (2016). National Survey on Substance Use in the General Population in Georgia, 2015. Tbilisi, Georgia: USAID & CzDA funded Addiction Research Development in Georgia Project.
		National Center for Disease Control and Public, Division of Reproductive Health, Centers for Disease Control and Prevention (2012). Georgia Reproductive Health Survey 2010. Tbilisi, Georgia: National Center for Disease Control and Public Health.
		Roberts B, Stickley A, Gilmore AB, Danishevski K, Kizilova K, Bryden A, et al. (2013). Knowledge of the health impacts of smoking and public attitudes towards tobacco control in the former Soviet Union. Tobacco Control.22(6):e12.
		Russell S, Sturua L, Li C, Morgan J, Topuridze M, Blanton C, et al. (2019). The burden of non-communicable diseases and their related risk factors in the country of Georgia, 2015, BMC public health.19(Suppl 3):479. https://dx.doi.org/10.1186/s12889-019-6785-2.
		Stickley A, Koyanagi A, Roberts B, Murphy A, Kizilova K, Mckee M (2015). Male solitary drinking and hazardous alcohol use in nine countries of the former Soviet Union, Drug and Alcohol Dependence.150:105-111.
		World Health Organization (2010). STEPwise approach to surveillance (STEPS) Survey. (http://www.who.int/chp/steps/en/. Accessed: 01/12/2018).
		World Health Organization (2016). STEPwise approach to surveillance (STEPS) Survey. (https://extranet.who.int/ncdsmicrodata/index.php/catalog/STEPS. Accessed: 07/02/2020).
EUR	Germany	Bloomfield K, Allamani A, Beck F, Bergmark KH, Csemy L, Eisenbach-Stangl I, et al. (2005). Gender, culture and alcohol problems : a multi-national study. An EU concerted Action. Project final report. Berlin, Germany: Charite Campus Benjamin Franklin.
		ESPAD Group (2020). ESPAD Report 2019: Results from the European School Survey Project on Alcohol and Other Drugs. Luxembourg: EMCDDA Joint Publications, Publications Office of the European Union.
		Inchley J, Currie D, Budisavljevic S, Torsheim T, Jåstad A, Cosma A, et al. (2020). Spotlight on adolescent health and well-being. Findings from the 2017/2018 Health Behaviour in School-aged Children (HBSC) survey in Europe and Canada. International report. Volume 2. Key data. Copenhagen, Denmark: WHO Regional Office for Europe.
		Kraus L, Pabst A, Gomes De Matos E, Piontek D (2014). Kurzbericht Epidemiologischer Suchtsurvey 2012; Tabellenband: Prävalenz des Alkoholkonsums, episodischen Rauschtrinkens und alkoholbezogener Störungen nach Geschlecht und Alter im Jahr 2012. München: IFT Institut für Therapieforschung
		Lange C, Manz K, Kuntz, B (2017). Alcohol consumption among adults in Germany: heavy episodic drinking, Journal of Health Monitoring.2(2):71-77. doi:10.17886/RKI-GBE-2017-045.
		Piontek D, Kraus L, De Matos EG, Atzendorf J (2016). Der Epidemiologische Suchtsurvey 2015. Sucht.62:259-269.
		Robert Koch Institute (2014). Gesundheit in Deutschland aktuell 2012. Berlin, Germany: Beiträge zur Gesundheitsberichterstattung des Bundes.
		Seitz N-N, John L, Atzendorf J, Rauschert C, Kraus L (2019). Kurzbericht Epidemiologischer Suchtsurvey 2018. Tabellenband: Alkoholkonsum, episodisches Rauschtrinken und Hinweise auf Konsumabhängigkeit und -missbrauch nach Geschlecht und Alter im Jahr 2018. München: IFT Institut für Therapieforschung.
		Statistical Office of the European Union (Eurostat) (2019). European Health Interview Survey Wave 2 data table: Frequency of alcohol consumption by sex, age and educational attainment level. (https://ec.europa.eu/eurostat/databrowser/view/hlth_ehis_al1e/default/table?lang=en. Accessed: 08/17/2020).
		Statistical Office of the European Union (Eurostat) (2019). European Health Interview Survey Wave 2 data table: Frequency of heavy episodic drinking by sex, age and educational attainment level. (https://ec.europa.eu/eurostat/databrowser/view/hlth_ehis_al3e/default/table?lang=en. Accessed: 08/17/2020).
		The International GENACIS-Project (2000). GENACIS - gender, alcohol and culture: an international study. (http://www.genacis.org/. Accessed: 01/11/2018).
EUR	Greece	ESPAD Group (2020). ESPAD Report 2019: Results from the European School Survey Project on Alcohol and Other Drugs. Luxembourg: EMCDDA Joint Publications, Publications Office of the European Union.
		European Monitoring Centre for Drugs and Drug Addiction (2016). ESPAD Report 2015. Results from the European School Survey Project on Alcohol and Other Drugs. (http://www.espad.org/sites/espad.org/files/ESPAD_report_2015.pdf. Accessed: 06/15/2017).
		European Monitoring Centre for Drugs and Drug Addiction (2020). Statistical Bulletin 2020 – prevalence of drug use: Alcohol Data tables - lifetime prevalence, last year prevalence (https://www.emcdda.europa.eu/data/stats2020/gps. Accessed: 07/21/2020).
		Inchley J, Currie D, Budisavljevic S, Torsheim T, Jåstad A, Cosma A, et al. (2020). Spotlight on adolescent health and well-being. Findings from the 2017/2018 Health Behaviour in School-aged Children (HBSC) survey in Europe and Canada. International report. Volume 2. Key data. Copenhagen, Denmark: WHO Regional Office for Europe.
		Moskalewicz J, Room R, Thom B (2016). Comparative monitoring of alcohol epidemiology across the EU: Baseline assessment and suggestions for future action. Synthesis report. Warszawa, Poland: Joint Action on Reducing Alcohol Related Harm (RARHA).
		Statistical Office of the European Union (Eurostat) (2019). European Health Interview Survey Wave 2 data table: Frequency of alcohol consumption by sex, age and educational attainment level. (https://ec.europa.eu/eurostat/databrowser/view/hlth_ehis_al1e/default/table?lang=en. Accessed: 08/17/2020).
		Statistical Office of the European Union (Eurostat) (2019). European Health Interview Survey Wave 2 data table: Frequency of heavy episodic drinking by sex, age and educational attainment level. (https://ec.europa.eu/eurostat/databrowser/view/hlth_ehis_al3e/default/table?lang=en. Accessed: 08/17/2020).
EUR	Hungary	Bloomfield K, Allamani A, Beck F, Bergmark KH, Csemy L, Eisenbach-Stangl I, et al. (2005). Gender, culture and alcohol problems : a multi-national study. An EU concerted Action. Project final report. Berlin, Germany: Charite Campus Benjamin Franklin.
		ESPAD Group (2020). ESPAD Report 2019: Results from the European School Survey Project on Alcohol and Other Drugs. Luxembourg: EMCDDA Joint Publications, Publications Office of the European Union.

Region	Country	Sources of alcohol patterns of consumption data by WHO Member State
		European Monitoring Centre for Drugs and Drug Addiction (2020). Statistical Bulletin 2020 – prevalence of drug use: Alcohol Data tables - lifetime prevalence, last year prevalence (https://www.emcdda.europa.eu/data/stats2020/gps. Accessed: 07/21/2020).
		Inchley J, Currie D, Budisavljevic S, Torsheim T, Jåstad A, Cosma A, et al. (2020). Spotlight on adolescent health and well-being. Findings from the 2017/2018 Health Behaviour in School-aged Children (HBSC) survey in Europe and Canada. International report. Volume 2. Key data. Copenhagen, Denmark: WHO Regional Office for Europe.
		József V, Péter V (2015). Egészségjelentés 2015 [Health Report 2015]. (http://www.egeszseg.hu/szakmai_oldalak/assets/files/news/egeszsegjelentes-2015.pdf. Accessed: 07/18/2017).
		Moskalewicz J, Room R, Thom B (2016). Comparative monitoring of alcohol epidemiology across the EU: Baseline assessment and suggestions for future action. Synthesis report. Warszawa, Poland: Joint Action on Reducing Alcohol Related Harm (RARHA).
		Statistical Office of the European Union (Eurostat) (2019). European Health Interview Survey Wave 2 data table: Frequency of alcohol consumption by sex, age and educational attainment level. (https://ec.europa.eu/eurostat/databrowser/view/hlth_ehis_al1e/default/table?lang=en. Accessed: 08/17/2020).
		Statistical Office of the European Union (Eurostat) (2019). European Health Interview Survey Wave 2 data table: Frequency of heavy episodic drinking by sex, age and educational attainment level. (https://ec.europa.eu/eurostat/databrowser/view/hlth_ehis_al3e/default/table?lang=en. Accessed: 08/17/2020).
		The International GENACIS-Project (2001). GENACIS - gender, alcohol and culture: an international study. (http://www.genacis.org/. Accessed: 01/11/2018).
EUR	Iceland	Bloomfield K, Allamani A, Beck F, Bergmark KH, Csemy L, Eisenbach-Stangl I, et al. (2005). Gender, culture and alcohol problems : a multi-national study. An EU concerted Action. Project final report. Berlin, Germany: Charite Campus Benjamin Franklin.
		ESPAD Group (2020). ESPAD Report 2019: Results from the European School Survey Project on Alcohol and Other Drugs. Luxembourg: EMCDDA Joint Publications, Publications Office of the European Union.
		European Monitoring Centre for Drugs and Drug Addiction (2016). ESPAD Report 2015. Results from the European School Survey Project on Alcohol and Other Drugs. (http://www.espad.org/sites/espad.org/files/ESPAD_report_2015.pdf. Accessed: 06/15/2017)
		Guðlaugsson JO, Magnússon KT, Jónsson SH (2014). Heilsa og líðan Íslendinga 2012: Framkvæmdaskýrsla. [Health and Wellbeing of Icelanders 2012. Project Report]. (http://www.landlaeknir.is/tolfraedi-og-rannsoknir/rannsoknir/heilsa-og-lidan-islendinga/. Accessed: 0717/2017).
		Inchley J, Currie D, Budisavljevic S, Torsheim T, Jåstad A, Cosma A, et al. (2020). Spotlight on adolescent health and well-being. Findings from the 2017/2018 Health Behaviour in School-aged Children (HBSC) survey in Europe and Canada. International report. Volume 2. Key data. Copenhagen, Denmark: WHO Regional Office for Europe.
		Moskalewicz J, Room R, Thom B (2016). Comparative monitoring of alcohol epidemiology across the EU: Baseline assessment and suggestions for future action. Synthesis report. Warszawa, Poland: Joint Action on Reducing Alcohol Related Harm (RARHA).
		Statistical Office of the European Union (Eurostat) (2019). European Health Interview Survey Wave 2 data table: Frequency of alcohol consumption by sex, age and educational attainment level. (https://ec.europa.eu/eurostat/databrowser/view/hlth_ehis_al1e/default/table?lang=en. Accessed: 08/17/2020).
		Statistical Office of the European Union (Eurostat) (2019). European Health Interview Survey Wave 2 data table: Frequency of heavy episodic drinking by sex, age and educational attainment level. (https://ec.europa.eu/eurostat/databrowser/view/hlth_ehis_al3e/default/table?lang=en. Accessed: 08/17/2020).
		The International GENACIS-Project (2001). GENACIS - gender, alcohol and culture: an international study. (http://www.genacis.org/. Accessed: 01/11/2018).
EUR	Ireland	Bloomfield K, Allamani A, Beck F, Bergmark KH, Csemy L, Eisenbach-Stangl I, et al. (2005). Gender, culture and alcohol problems : a multi-national study. An EU concerted Action. Project final report. Berlin, Germany: Charite Campus Benjamin Franklin.
		Department of Health (2020). Healthy Ireland Survey, 2018. Version 1. Irish Social Science Data Archive. (www.ucd.ie/issda/data/healthyireland. Accessed 10/19/2020).
		ESPAD Group (2020). ESPAD Report 2019: Results from the European School Survey Project on Alcohol and Other Drugs. Luxembourg: EMCDDA Joint Publications, Publications Office of the European Union.
		European Monitoring Centre for Drugs and Drug Addiction (2016). ESPAD report 2015. Results from the European School Survey Project on Alcohol and Other Drugs. (http://www.espad.org/sites/espad.org/files/ESPAD_report_2015.pdf. Accessed: 06/15/2017).
		European Monitoring Centre for Drugs and Drug Addiction (2020). Statistical Bulletin 2020 – prevalence of drug use: Alcohol Data tables - lifetime prevalence, last year prevalence (https://www.emcdda.europa.eu/data/stats2020/gps. Accessed: 07/21/2020).
		Inchley J, Currie D, Budisavljevic S, Torsheim T, Jåstad A, Cosma A, et al. (2020). Spotlight on adolescent health and well-being. Findings from the 2017/2018 Health Behaviour in School-aged Children (HBSC) survey in Europe and Canada. International report. Volume 2. Key data. Copenhagen, Denmark: WHO Regional Office for Europe.
		Ipsos MRBI (2016). Healthy Ireland survey 2016 - Summary of Findings. (http://health.gov.ie/wp-content/uploads/2016/10/Healthy-Ireland-Survey-2016-Summary-Findings.pdf. Accessed: 07/19/2017).
		Long J, Mongan D (2014). Alcohol consumption in Ireland 2013: analysis of a national alcohol diary survey. Dublin, Ireland: Health Research Board.
		Mongan D, Millar SR, O'Dwyer C, Long J, Galvin B (2020). Drinking in denial: a cross-sectional analysis of national survey data in Ireland to measure drinkers' awareness of their alcohol use, BMJ open.10(7):e034520. https://dx.doi.org/10.1136/bmjopen-2019-034520.
		Statistical Office of the European Union (Eurostat) (2019). European Health Interview Survey Wave 2 data table: Frequency of alcohol consumption by sex, age and educational attainment level. (https://ec.europa.eu/eurostat/databrowser/view/hlth_ehis_al1e/default/table?lang=en. Accessed: 08/17/2020).

Region	Country	Sources of alcohol patterns of consumption data by WHO Member State
		Statistical Office of the European Union (Eurostat) (2019). European Health Interview Survey Wave 2 data table: Frequency of heavy episodic drinking by sex, age and educational attainment level. (https://ec.europa.eu/eurostat/databrowser/view/hlth_ehis_al3e/default/table?lang=en. Accessed: 08/17/2020).
		The International GENACIS-Project (2002). GENACIS - gender, alcohol and culture: an international study. (http://www.genacis.org/. Accessed: 01/11/2018).
EUR	Israel	Israel Center for Disease Control, Ministry of Health (2012). Israel National Health Interview Survey INHIS-2, 2007-2010 – Selected Findings. Israel: Israel Center for Disease Control.
		The International GENACIS-Project (2001). GENACIS - gender, alcohol and culture: an international study. (http://www.genacis.org/. Accessed: 01/11/2018).
EUR	Italy	Bloomfield K, Allamani A, Beck F, Bergmark KH, Csemy L, Eisenbach-Stangl I, et al. (2005). Gender, culture and alcohol problems : a multi-national study. An EU concerted Action. Project final report. Berlin, Germany: Charite Campus Benjamin Franklin.
		ESPAD Group (2020). ESPAD Report 2019: Results from the European School Survey Project on Alcohol and Other Drugs. Luxembourg: EMCDDA Joint Publications, Publications Office of the European Union.
		Inchley J, Currie D, Budisavljevic S, Torsheim T, Jåstad A, Cosma A, et al. (2020). Spotlight on adolescent health and well-being. Findings from the 2017/2018 Health Behaviour in School-aged Children (HBSC) survey in Europe and Canada. International report. Volume 2. Key data. Copenhagen, Denmark: WHO Regional Office for Europe.
		Instituto Nazionale Di Statistica (2011). Use and abuse of alcohol in Italy 2010. (https://www.istat.it/it/files//2011/04/Alcol_2010_EN.pdf. Accessed: 06/16/2017).
		Instituto Nazionale Di Statistica (2013). L'uso e l'abuso di alcol in Italia. (http://www.istat.it/it/archivio/88167. Accessed: 06/16/2017).
		Moskalewicz J, Room R, Thom B (2016). Comparative monitoring of alcohol epidemiology across the EU: Baseline assessment and suggestions for future action. Synthesis report. Warszawa, Poland: Joint Action on Reducing Alcohol Related Harm (RARHA).
		Scafato E (2017a). RE: Data provided by the osservatorio nazionale alcol (ONA) - ISS and the WHO CC research on alcohol based on the ISTAT multipurpose database of the survey on households-aspects of daily life 2013. (Accessed: 01/10/2018).
		Scafato E (2017b). RE: Data provided by the osservatorio nazionale alcol (ONA) - ISS and the WHO CC research on alcohol based on the ISTAT multipurpose database of the survey on households-aspects of daily life 2016. (Accessed: 01/10/2018).
		Scafato E, Ghirini S, Gandin C, Vichi M, Matone A, Scipione R, et al. (2020). Epidemiologia e monitoraggio alcol-correlato in Italia e nelle Regioni. Valutazione dell'Osservatorio Nazionale Alcol sull'impatto del consumo di alcol ai fini dell'implementazione delle attività del Piano Nazionale Alcol e Salute. Rapporto 2020. Roma, Italia: Istituto Superiore di Sanità.
		Scafato E, Ghirini S, Gandin C, Vichi M, Scipione R, il gruppo di lavoro CSDA (Centro Servizi Documentazione Alcol) (2019). Epidemiologia e monitoraggio alcol-correlato in Italia e nelle Regioni. Valutazione dell'Osservatorio Nazionale Alcol sull'impatto del consumo di alcol ai fini dell'implementazione delle attività del Piano Nazionale Alcol e Salute. Rapporto 2019. Roma, Italia: Istituto Superiore di Sanità.
		Statistical Office of the European Union (Eurostat) (2019). European Health Interview Survey Wave 2 data table: Frequency of alcohol consumption by sex, age and educational attainment level. (https://ec.europa.eu/eurostat/databrowser/view/hlth_ehis_al1e/default/table?lang=en. Accessed: 08/17/2020).
		Statistical Office of the European Union (Eurostat) (2019). European Health Interview Survey Wave 2 data table: Frequency of heavy episodic drinking by sex, age and educational attainment level. (https://ec.europa.eu/eurostat/databrowser/view/hlth_ehis_al3e/default/table?lang=en. Accessed: 08/17/2020).
		The International GENACIS-Project (2000). GENACIS - gender, alcohol and culture: an international study. (http://www.genacis.org/. Accessed: 01/11/2018).
		The International GENACIS-Project (2002). GENACIS - gender, alcohol and culture: an international study. (http://www.genacis.org/. Accessed: 01/11/2018).
EUR	Kazakhstan	Abe S, Stickley A, Roberts B, Richardson E, Abbott P, Rotman D, et al. (2013). Changing patterns of fruit and vegetable intake in countries of the former Soviet Union, Public Health Nutrition.16(11):1924-1932.
		Inchley J, Currie D, Budisavljevic S, Torsheim T, Jåstad A, Cosma A, et al. (2020). Spotlight on adolescent health and well-being. Findings from the 2017/2018 Health Behaviour in School-aged Children (HBSC) survey in Europe and Canada. International report. Volume 2. Key data. Copenhagen, Denmark: WHO Regional Office for Europe.
		The International GENACIS-Project (2003). GENACIS - gender, alcohol and culture: an international study. (http://www.genacis.org/. Accessed: 01/10/2018).
		The Agency of Statistics, RK, United Nations Children's Fund (UNICEF) (2012). Multiple Indicator Cluster Survey (MICS) in the Republic of Kazakhstan, 2010-2011, Final Report. Astana, Kazakhstan: the Agency of Statistics, RK, Republican State Enterprise Information Computing Center.
		The Statistics Committee of the Ministry of National Economy of the Republic of Kazakhstan (Statistics Committee of the MNE RK), United Nations Children's Fund (UNICEF), the United Nations Population Fund (UNFPA) (2015). 2015 Kazakhstan Multiple Indicator Cluster Survey, Final Report. Astana, Kazakhstan: Statistics Committee of the MNE RK, UNICEF, UNFPA.
EUR	Kyrgyzstan	Abe S, Stickley A, Roberts B, Richardson E, Abbott P, Rotman D, et al. (2013). Changing patterns of fruit and vegetable intake in countries of the former Soviet Union, Public Health Nutrition.16(11):1924-1932.
		National Statistical Committee of the Kyrgyz Republic (NSC), Ministry of Health [Kyrgyz Republic], and ICF International (2013). Kyrgyz Republic Demographic and Health Survey 2012. (https://dhsprogram.com/pubs/pdf/fr283/fr283.pdf. Accessed: 06/24/2019).
		National Statistical Committee of the Kyrgyz Republic, UNICEF (2015). Kyrgyzstan Multiple Indicator Cluster Survey 2014, Final Report. Bishkek, Kyrgyzstan: National Statistical Committee of the Kyrgyz Republic, UNICEF.
		World Health Organization (2013c). STEPwise approach to surveillance (STEPS) Survey. (http://www.who.int/chp/steps/en/. Accessed: 01/15/2018).
EUR	Latvia	ESPAD Group (2020). ESPAD Report 2019: Results from the European School Survey Project on Alcohol and Other Drugs. Luxembourg: EMCDDA Joint Publications, Publications Office of the European Union.

Region	Country	Sources of alcohol patterns of consumption data by WHO Member State
		European Commission (2012). Eurobarometer 72.3 (Oct 2009). TNS OPINION & SOCIAL. Brussels: GESIS Data Archive.
		European Monitoring Centre for Drugs and Drug Addiction (2016). ESPAD report 2015. Results from the European School Survey Project on Alcohol and Other Drugs. (http://www.espad.org/sites/espad.org/files/ESPAD_report_2015.pdf. Accessed: 06/15/2017).
		Grīnberga D, Pudule I, Velika B, Gavare I, Villeruša A (2015). Latvijas iedzīvotāju veselību ietekmējošo paradumu pētijums, 2014: Health Behaviour among Latvian Adult Population, 2014. (https://www.spkc.gov.lv/lv/veselibu-ietekmejoso-paradumu-petijumi/latvijas-iedzivotaju-veselibu-ietekmejoso-paradumu-petijums-2014-i-un-ii-dala.pdf/latvijas-iedzivotaju-veselibu-ietekmejoso-paradumu-petijums-2014-i-un-ii-dala.pdf. Accessed: 09/03/2020).
		Grīnberga D, Velika B, Pudule I, Gavare I, Villeruša A (2017). Latvijas iedzīvotāju veselību ietekmējošo paradumu pētījums, 2016: Health Behaviour among Latvian Adult Population, 2016. (https://www.spkc.gov.lv/lv/veselibu-ietekmejoso-paradumu-petijumi/latvijas-iedzivotaju-veselibu-ietekmejoso-paradumu-petijums-2016-i-un-ii-dala.pdf/latvijas-iedzivotaju-veselibu-ietekmejoso-paradumu-petijums-2016-i-un-ii-dala.pdf. Accessed: 09/03/2020).
		Grīnberga D, Velika B, Pudule I, Gavare I, Villeruša A (2019). Latvijas iedzīvotāju veselību ietekmējošo paradumu pētījums, 2018: Health Behaviour among Latvian Adult Population, 2018. (https://www.spkc.gov.lv/lv/veselibu-ietekmejoso-paradumu-petijumi/latvijas-iedzivotaju-veselibu-ietekmejoso-paradumu-petijums-2018-i-un-ii-dala.pdf/latvijas-iedzivotaju-veselibu-ietekmejoso-paradumu-petijums-2018-i-un-ii-dala.pdf. Accessed: 09/03/2020).
		Inchley J, Currie D, Budisavljevic S, Torsheim T, Jåstad A, Cosma A, et al. (2020). Spotlight on adolescent health and well-being. Findings from the 2017/2018 Health Behaviour in School-aged Children (HBSC) survey in Europe and Canada. International report. Volume 2. Key data. Copenhagen, Denmark: WHO Regional Office for Europe.
		Pudule I, Grīnberga D, Velika B, Gavare I, Villeruša A (2013). Latvijas iedzīvotāju veselību ietekmējošo paradumu pētījums, 2012: Health Behaviour among Latvian Adult Population, 2012. (https://www.spkc.gov.lv/lv/veselibu-ietekmejoso-paradumu-petijumi/latvijas-iedzivotaju-veselibu-ietekmejoso-paradumu-petijums-2012-i-un-ii-dala.pdf/latvijas-iedzivotaju-veselibu-ietekmejoso-paradumu-petijums-2012-i-un-ii-dala.pdf. Accessed: 09/03/2020).
		Pudule I, Villeruša A, Grīnberga D, Velika B, Taube M, Behmane D, et al. (2011). Latvijas iedzīvotāju veselību ietekmējošo paradumu pētījums, 2010: Health Behaviour among Latvian Adult Population, 2010. (https://www.spkc.gov.lv/lv/veselibu-ietekmejoso-paradumu-petijumi/latvijas-iedzivotaju-veselibu-ietekmejoso-paradumu-petijums-2010-i-un-ii-dala.pdf/latvijas-iedzivotaju-veselibu-ietekmejoso-paradumu-petijums-2010-i-un-ii-dala.pdf. Accessed: 09/03/2020).
		Snikere S, Trapencieris M, Koroleva I, Mierina I, Priekule S, Aleksandrovs A, et al. (2011). Atkarību izraisoso vielu lietosana iedzīvotāju vidū 2011: Analītisks pārskats. Riga: Slimibu profilakses un kontroles centrs.
		Snikere S, Trapencieris M (2016). Atkarību izraisos o vielu lietos ana iedzīvotāju vidū 2015: Analītisks pārskats. (https://www.spkc.gov.lv/upload/Petijumi%20un%20zinojumi/Atkaribu%20slimibu%20petijumi/gps2015_final_ar_isbn.pdf Accessed: 01/25/2018).
		Statistical Office of the European Union (Eurostat) (2019). European Health Interview Survey Wave 2 data table: Frequency of alcohol consumption by sex, age and educational attainment level. (https://ec.europa.eu/eurostat/databrowser/view/hlth_ehis_al1e/default/table?lang=en. Accessed: 08/17/2020).
		Statistical Office of the European Union (Eurostat) (2019). European Health Interview Survey Wave 2 data table: Frequency of heavy episodic drinking by sex, age and educational attainment level. (https://ec.europa.eu/eurostat/databrowser/view/hlth_ehis_al3e/default/table?lang=en. Accessed: 08/17/2020).
EUR	Lithuania	ESPAD Group (2020). ESPAD Report 2019: Results from the European School Survey Project on Alcohol and Other Drugs. Luxembourg: EMCDDA Joint Publications, Publications Office of the European Union.
		European Commission (2012). Eurobarometer 72.3 (Oct 2009). TNS OPINION & SOCIAL. Brussels: GESIS Data Archive.
		European Monitoring Centre for Drugs and Drug Addiction (2020). Statistical Bulletin 2020 – prevalence of drug use: Alcohol Data tables - lifetime prevalence, last year prevalence (https://www.emcdda.europa.eu/data/stats2020/gps. Accessed: 07/21/2020).
		Inchley J, Currie D, Budisavljevic S, Torsheim T, Jåstad A, Cosma A, et al. (2020). Spotlight on adolescent health and well-being. Findings from the 2017/2018 Health Behaviour in School-aged Children (HBSC) survey in Europe and Canada. International report. Volume 2. Key data. Copenhagen, Denmark: WHO Regional Office for Europe.
		Lietuvos Statistikos Depaprtamentas Statistics Lithuania (2015). Results of the Health Interview Survey of the Population of Lithuania 2014. (https://osp.stat.gov.lt/services-portlet/pub-edition-file?id=20908. Accessed: 07/19/2017).
		Moskalewicz J, Room R, Thom B (2016). Comparative monitoring of alcohol epidemiology across the EU: Baseline assessment and suggestions for future action. Synthesis report. Warszawa, Poland: Joint Action on Reducing Alcohol Related Harm (RARHA).
		Statistical Office of the European Union (Eurostat) (2019). European Health Interview Survey Wave 2 data table: Frequency of alcohol consumption by sex, age and educational attainment level. (https://ec.europa.eu/eurostat/databrowser/view/hlth_ehis_al1e/default/table?lang=en. Accessed: 08/17/2020).
		Statistical Office of the European Union (Eurostat) (2019). European Health Interview Survey Wave 2 data table: Frequency of heavy episodic drinking by sex, age and educational attainment level. (https://ec.europa.eu/eurostat/databrowser/view/hlth_ehis_al3e/default/table?lang=en. Accessed: 08/17/2020).
EUR	Luxembourg	Inchley J, Currie D, Budisavljevic S, Torsheim T, Jåstad A, Cosma A, et al. (2020). Spotlight on adolescent health and well-being. Findings from the 2017/2018 Health Behaviour in School-aged Children (HBSC) survey in Europe and Canada. International report. Volume 2. Key data. Copenhagen, Denmark: WHO Regional Office for Europe.
		Ministère De La Santé Luxembourg (2017). European Health Interview Survey sur la consommation du tabac et d'alcool. (https://sante.public.lu/fr/espace-professionnel/informations-donnees/ehis/ehis-etat-determinants.html. Accessed: 01/12/2018).
		Ruiz-Castell M, Kandala NB, Kuemmerle A, Schritz A, Barré J, Delagardelle C, et al. (2016). Hypertension burden in Luxembourg: Individual risk factors and geographic variations, 2013 to 2015 European Health Examination Survey, Medicine.95(36):e4758.
		Statistical Office of the European Union (Eurostat) (2019). European Health Interview Survey Wave 2 data table: Frequency of alcohol consumption by sex, age and educational attainment level. (https://ec.europa.eu/eurostat/databrowser/view/hlth_ehis_al1e/default/table?lang=en. Accessed: 08/17/2020).

Region	Country	Sources of alcohol patterns of consumption data by WHO Member State
		Statistical Office of the European Union (Eurostat) (2019). European Health Interview Survey Wave 2 data table: Frequency of heavy episodic drinking by sex, age and educational attainment level. (https://ec.europa.eu/eurostat/databrowser/view/hlth_ehis_al3e/default/table?lang=en. Accessed: 08/17/2020).
EUR	Malta	ESPAD Group (2020). ESPAD Report 2019: Results from the European School Survey Project on Alcohol and Other Drugs. Luxembourg: EMCDDA Joint Publications, Publications Office of the European Union.
		European Monitoring Centre for Drugs and Drug Addiction, European School Survey Project on Alcohol and Other Drugs (ESPAD), Pompidou Group, Council of Europe, Swedish Council for Information on Alcohol and Other Drugs (Can) (2012). ESPAD Report 2011: Substance Use Among Students in 36 European Countries. Stockholm, Sweden: Swedish Council for Information on Alcohol and Other Drugs (CAN).
		Inchley J, Currie D, Budisavljevic S, Torsheim T, Jåstad A, Cosma A, et al. (2020). Spotlight on adolescent health and well-being. Findings from the 2017/2018 Health Behaviour in School-aged Children (HBSC) survey in Europe and Canada. International report. Volume 2. Key data. Copenhagen, Denmark: WHO Regional Office for Europe.
		Reitox National Focal Point (2014). Malta - New Developments and Trends. Malta: European Monitoring Centre for Drugs and Drug Addiction.
		Statistical Office of the European Union (Eurostat) (2019). European Health Interview Survey Wave 2 data table: Frequency of alcohol consumption by sex, age and educational attainment level. (https://ec.europa.eu/eurostat/databrowser/view/hlth_ehis_al1e/default/table?lang=en. Accessed: 08/17/2020).
		Statistical Office of the European Union (Eurostat) (2019). European Health Interview Survey Wave 2 data table: Frequency of heavy episodic drinking by sex, age and educational attainment level. (https://ec.europa.eu/eurostat/databrowser/view/hlth_ehis_al3e/default/table?lang=en. Accessed: 08/17/2020).
EUR	Monaco	ESPAD Group (2020). ESPAD Report 2019: Results from the European School Survey Project on Alcohol and Other Drugs. Luxembourg: EMCDDA Joint Publications, Publications Office of the European Union.
		World Health Organization (2017b). STEPwise approach to surveillance (STEPS). (http://www.who.int/chp/steps/en/. Accessed: 04/11/2017).
EUR	Montenegro	ESPAD Group (2020). ESPAD Report 2019: Results from the European School Survey Project on Alcohol and Other Drugs. Luxembourg: EMCDDA Joint Publications, Publications Office of the European Union.
		Institute of Public Health Montenegro, European Monitoring Centre for Drugs and Drug Addiction (2017). General Population Survey on Substance Use in Montenegro 2017: Survey Report. (https://www.emcdda.europa.eu/drugs-library/report-general-population-survey-substance-use-montenegro-2017_en. Accessed: 06/22/2020).
		Mugoša B, Đurišic T, Golubović L (2008). European School Survey Project on Tobacco, Alcohol and Drugs. Montenegro: Public Health Institute of Montenegro Podgorica.
		Statistical Office of Montenegro (MONSTAT), UNICEF (2013). 2013 Montenegro and Montenegro Roma Settlements Multiple Indicator Cluster Survey, Final Report. Podgorica, Montenegro: MONSTAT, UNICEF.
EUR	Netherlands	Abidi, L., Nilsen, P., Karlsson, N., Skagerström, J., O'Donnell, A. (2020). Conversations about alcohol in healthcare – cross-sectional surveys in the Netherlands and Sweden, BMC Public Health.20:283. https://doi.org/10.1186/s12889-020-8367-8.
		Bloomfield K, Allamani A, Beck F, Bergmark KH, Csemy L, Eisenbach-Stangl I, et al. (2005). Gender, culture and alcohol problems : a multi-national study. An EU concerted Action. Project final report. Berlin, Germany: Charite Campus Benjamin Franklin.
		de Looze ME, van Dorsselaer SAFM, Monshouwer K, Vollebergh WAM (2017). Trends in adolescent alcohol use in the Netherlands, 1992-2015: Differences across sociodemographic groups and links with strict parental rule-setting, The International journal on drug policy.50(9014759):90-101. https://dx.doi.org/10.1016/j.drugpo.2017.09.013.
		de Looze ME, Vermeulen-Smit E, ter Bogt TFM, van Dorsselaer SAFM, Verdurmen J, Schulten I, et al. (2014). Trends in alcohol-specific parenting practices and adolescent alcohol use between 2007 and 2011 in the Netherlands, The International journal on drug policy.25(1):133-41. https://dx.doi.org/10.1016/j.drugpo.2013.09.007.
		ESPAD Group (2020). ESPAD Report 2019: Results from the European School Survey Project on Alcohol and Other Drugs. Luxembourg: EMCDDA Joint Publications, Publications Office of the European Union.
		European Monitoring Centre for Drugs and Drug Addiction (2016). ESPAD report (2015). Results from the European School Survey Project on Alcohol and Other Drugs. (http://www.espad.org/sites/espad.org/files/ESPAD_report_2015.pdf. Accessed: 06/15/2017).
		European Monitoring Centre for Drugs and Drug Addiction (2020). Statistical Bulletin 2020 – prevalence of drug use: Alcohol Data tables - lifetime prevalence, last year prevalence (https://www.emcdda.europa.eu/data/stats2020/gps. Accessed: 07/21/2020).
		Garretsen HFL, Rodenburg G, Van De Goor LaM, Van Den Eijnden RJJM (2008). Alcohol consumption in the Netherlands in the last decade: sharp decreases in binge drinking, especially among youngsters, Alcohol and Alcoholism.43(4):477-480.
		Health Survey/Lifestyle Monitor, Statistics Netherlands (CBS), National Institute for Public Health and the Environment (RIVM), Trimbos-institute (2020). Life style and (preventive) health examination; personal characteristics – data table. (https://opendata.cbs.nl/#/CBS/en/dataset/83021ENG/table?dl=481FC. Accessed: 12/18/2020).
		Inchley J, Currie D, Budisavljevic S, Torsheim T, Jåstad A, Cosma A, et al. (2020). Spotlight on adolescent health and well-being. Findings from the 2017/2018 Health Behaviour in School-aged Children (HBSC) survey in Europe and Canada. International report. Volume 2. Key data. Copenhagen, Denmark: WHO Regional Office for Europe.
		Jonkman H, Steketee M, Tombourou JW, Cini K, Williams J (2014). Community variation in adolescent alcohol use in Australia and the Netherlands. Health Promotion International.29:109-117.
		The International GENACIS-Project (1999). GENACIS - gender, alcohol and culture: an international study. (http://www.genacis.org/. Accessed: 01/11/2018).
EUR	Norway	Bloomfield K, Allamani A, Beck F, Bergmark KH, Csemy L, Eisenbach-Stangl I, et al. (2005). Gender, culture and alcohol problems : a multi-national study. An EU concerted Action. Project final report. Berlin, Germany: Charite Campus Benjamin Franklin.
		Eliasen M, Kjær SK, Munk C, Nygård M, Sparén P, Tryggvadottir L, et al. (2009). The relationship between age at drinking onset and subsequent binge drinking among women, European Journal of Public Health.19(4):378-382.
		ESPAD Group (2020). ESPAD Report 2019: Results from the European School Survey Project on Alcohol and Other Drugs. Luxembourg: EMCDDA Joint Publications, Publications Office of the European Union.

Region	Country	Sources of alcohol patterns of consumption data by WHO Member State
		European Monitoring Centre for Drugs and Drug Addiction (2016). ESPAD report 2015. Results from the European School Survey Project on Alcohol and Other Drugs. (http://www.espad.org/sites/espad.org/files/ESPAD_report_2015.pdf. Accessed: 06/15/2017).
		European Monitoring Centre for Drugs and Drug Addiction (2020). Statistical Bulletin 2020 – prevalence of drug use: Alcohol Data tables - lifetime prevalence, last year prevalence (https://www.emcdda.europa.eu/data/stats2020/gps. Accessed: 07/21/2020).
		European Monitoring Centre for Drugs and Drug Addiction, European School Survey Project on Alcohol and Other Drugs (ESPAD), Pompidou Group, Council of Europe & Swedish Council for Information on Alcohol and Other Drugs (Can) (2012). ESPAD report 2011: Substance Use Among Students in 36 European Countries. Stockholm, Sweden: Swedish Council for Information on Alcohol and Other Drugs (CAN).
		Inchley J, Currie D, Budisavljevic S, Torsheim T, Jåstad A, Cosma A, et al. (2020). Spotlight on adolescent health and well-being. Findings from the 2017/2018 Health Behaviour in School-aged Children (HBSC) survey in Europe and Canada. International report. Volume 2. Key data. Copenhagen, Denmark: WHO Regional Office for Europe.
		Moskalewicz J, Room R, Thom B (2016). Comparative monitoring of alcohol epidemiology across the EU: Baseline assessment and suggestions for future action. Synthesis report. Warszawa, Poland: Joint Action on Reducing Alcohol Related Harm (RARHA).
		Rossow I, Mäkelä P, Kerr W (2014). The collectivity of changes in alcohol consumption revisited, Addiction.109(9):1447-1455.
		Skretting A, Vedøy TF, Lund KE, Bye EK (2016). Rusmidler i Norge 2016: Alkohol, tobakk, vanedannende legemidler, narkotika, snifng, doping og tjenestetilbudet. (https://www.fhi.no/globalassets/dokumenterfiler/rapporter/rusmidler_i_norge_2016.pdf. Accessed: 01/22/2018).
		Statistical Office of the European Union (Eurostat) (2019). European Health Interview Survey Wave 2 data table: Frequency of alcohol consumption by sex, age and educational attainment level. (https://ec.europa.eu/eurostat/databrowser/view/hlth_ehis_al1e/default/table?lang=en. Accessed: 08/17/2020).
		Statistical Office of the European Union (Eurostat) (2019). European Health Interview Survey Wave 2 data table: Frequency of heavy episodic drinking by sex, age and educational attainment level. (https://ec.europa.eu/eurostat/databrowser/view/hlth_ehis_al3e/default/table?lang=en. Accessed: 08/17/2020).
		Statistics Norway (2020). 12392: Use of alcohol, cannabis and addictive drugs (per cent), by sex, age, contents and year – data table. (https://www.ssb.no/en/statbank/table/12392/tableViewLayout1/. Accessed: 12/21/2020).
		The International GENACIS-Project (1999). GENACIS - gender, alcohol and culture: an international study. (http://www.genacis.org/. Accessed: 01/11/2018).
EUR	Poland	Andersson B, Hibell B (2003). Alcohol and drug use among European 17–18 year old students. ESPAD 2003. Stockholm, Sweden: Modintryckoffset AB.
		Bieńkuńskiej A, Armatys P, Ciecieląg P, Haponiuk M, Jachowicz I, Kolasa E, et al. (2017). Jakość życia osób starszych w Polsce – Na podstawie wyników badania spójności społecznej 2015. (https://stat.gov.pl/files/gfx/portalinformacyjny/pl/defaultaktualnosci/5486/26/1/1/jakosc_zycia_osob_starszych_w_polsce.pdf. Accessed: 06/28/2020).
		ESPAD Group (2020). ESPAD Report 2019: Results from the European School Survey Project on Alcohol and Other Drugs. Luxembourg: EMCDDA Joint Publications, Publications Office of the European Union.
		European Monitoring Centre for Drugs and Drug Addiction (2016). ESPAD report 2015. Results from the European School Survey Project on Alcohol and Other Drugs. (http://www.espad.org/sites/espad.org/files/ESPAD_report_2015.pdf. Accessed: 06/15/2017).
		European Monitoring Centre for Drugs and Drug Addiction (2020). Statistical Bulletin 2020 – prevalence of drug use: Alcohol Data tables - lifetime prevalence, last year prevalence (https://www.emcdda.europa.eu/data/stats2020/gps. Accessed: 07/21/2020).
		European Monitoring Centre for Drugs and Drug Addiction, European School Survey Project on Alcohol and Other Drugs (ESPAD), Pompidou Group, Council of Europe, Swedish Council for Information on Alcohol and Other Drugs (Can) (2012). ESPAD report 2011: Substance Use Among Students in 36 European Countries. Stockholm, Sweden: Swedish Council for Information on Alcohol and Other Drugs (CAN).
		Inchley J, Currie D, Budisavljevic S, Torsheim T, Jåstad A, Cosma A, et al. (2020). Spotlight on adolescent health and well-being. Findings from the 2017/2018 Health Behaviour in School-aged Children (HBSC) survey in Europe and Canada. International report. Volume 2. Key data. Copenhagen, Denmark: WHO Regional Office for Europe.
		Moskalewicz J, Room R, Thom B (2016). Comparative monitoring of alcohol epidemiology across the EU: Baseline assessment and suggestions for future action. Synthesis report. Warszawa, Poland: Joint Action on Reducing Alcohol Related Harm (RARHA).
		Statistical Office of the European Union (Eurostat) (2019). European Health Interview Survey Wave 2 data table: Frequency of alcohol consumption by sex, age and educational attainment level. (https://ec.europa.eu/eurostat/databrowser/view/hlth_ehis_al1e/default/table?lang=en. Accessed: 08/17/2020).
		Statistical Office of the European Union (Eurostat) (2019). European Health Interview Survey Wave 2 data table: Frequency of heavy episodic drinking by sex, age and educational attainment level. (https://ec.europa.eu/eurostat/databrowser/view/hlth_ehis_al3e/default/table?lang=en. Accessed: 08/17/2020).
EUR	Portugal	Balsa C, Vital C, Urbano C (2013). III Inquérito Nacional ao Consumo de Substâncias Psicoativas na População Portuguesa: Portugal 2013. Relatório Preliminar. Lisbon, Portugal: Ministry of Health.
		ESPAD Group (2020). ESPAD Report 2019: Results from the European School Survey Project on Alcohol and Other Drugs. Luxembourg: EMCDDA Joint Publications, Publications Office of the European Union.
		European Monitoring Centre for Drugs and Drug Addiction (2016). ESPAD report 2015. Results from the European School Survey Project on Alcohol and Other Drugs. (http://www.espad.org/sites/espad.org/files/ESPAD_report_2015.pdf. Accessed: 06/15/2017).
		European Monitoring Centre for Drugs and Drug Addiction, European School Survey Project on Alcohol and Other Drugs (ESPAD), Pompidou Group, Council of Europe, Swedish Council for Information on Alcohol and Other Drugs (Can) (2012).

Region	Country	Sources of alcohol patterns of consumption data by WHO Member State
		ESPAD report 2011: Substance Use Among Students in 36 European Countries. Stockholm, Sweden: Swedish Council for Information on Alcohol and Other Drugs (CAN).
		Inchley J, Currie D, Budisavljevic S, Torsheim T, Jåstad A, Cosma A, et al. (2020). Spotlight on adolescent health and well-being. Findings from the 2017/2018 Health Behaviour in School-aged Children (HBSC) survey in Europe and Canada. International report. Volume 2. Key data. Copenhagen, Denmark: WHO Regional Office for Europe.
		Instituto Nacional De Estatística (2016). Inquérito Nacional de Saúde 2014 - data tables generated online. (https://www.ine.pt/xportal/xmain?xpid=INE&xpgid=ine_indicadores&indOcorrCod=0008823&contexto=bd&selTab=tab2&xlang=en. Accessed: 06/26/2017).
		Instituto Nacional De Estatística (2020a). Resident population with 15 and more years old that consumed alcoholic beverages in the 12 months preceding the interview (No.) by Sex, Age group and Condition regarding risky single-occasion drinking; Quinquennial - Statistics Portugal, National health survey (series 2014) – data table generated online. (https://www.ine.pt/xportal/xmain?xpid=INE&xpgid=ine_indicadores&indOcorrCod=0008704&contexto=bd&selTab=tab2. Accessed: 06/30/2020).
		Instituto Nacional De Estatística (2020b). Resident population with 15 and more years old (No.) by Place of residence (NUTS - 2013), Sex, Age group and Condition regarding alcoholic beverages consumption in the 12 months preceding the interview; Quinquennial - Statistics Portugal, National health survey (series 2014) - data table generated online. (https://www.ine.pt/xportal/xmain?xpid=INE&xpgid=ine_indicadores&indOcorrCod=0010210&contexto=bd&selTab=tab2. Accessed: 10/05/2020).
		Instituto Nacional De Estadística (2020c). National Health Survey 2019 Press Release Tables - 5.4: Resident population aged 15 and over who consumed alcoholic beverages in the 12 months preceding the interview by condition regarding risky single-occasion drinking, sex and age group, Portugal, 2019. (https://www.ine.pt/xportal/xmain?xpid=INE&xpgid=ine_destaques&DESTAQUESdest_boui=414436388&DESTAQUESmodo=2. Accessed: 11/26/2020).
		Jorge R, Instituto Nacional de Saúde (2017). 1º Inquérito Nacional de Saúde com Exame Físico (INSEF 2015): Determinantes de Saúde. Lisboa, Portugal: INSA IP.
		Moskalewicz J, Room R, Thom B (2016). Comparative monitoring of alcohol epidemiology across the EU: Baseline assessment and suggestions for future action. Synthesis report. Warszawa, Poland: Joint Action on Reducing Alcohol Related Harm (RARHA).
		Statistical Office of the European Union (Eurostat) (2019). European Health Interview Survey Wave 2 data table: Frequency of alcohol consumption by sex, age and educational attainment level. (https://ec.europa.eu/eurostat/databrowser/view/hlth_ehis_al1e/default/table?lang=en. Accessed: 08/17/2020).
		Statistical Office of the European Union (Eurostat) (2019). European Health Interview Survey Wave 2 data table: Frequency of heavy episodic drinking by sex, age and educational attainment level. (https://ec.europa.eu/eurostat/databrowser/view/hlth_ehis_al3e/default/table?lang=en. Accessed: 08/17/2020).
EUR	Republic of Moldova	Abe S, Stickley A, Roberts B, Richardson E, Abbott P, Rotman D, et al. (2013). Changing patterns of fruit and vegetable intake in countries of the former Soviet Union, Public Health Nutrition.16(11):1924-1932.
		Inchley J, Currie D, Budisavljevic S, Torsheim T, Jåstad A, Cosma A, et al. (2020). Spotlight on adolescent health and well-being. Findings from the 2017/2018 Health Behaviour in School-aged Children (HBSC) survey in Europe and Canada. International report. Volume 2. Key data. Copenhagen, Denmark: WHO Regional Office for Europe.
		National Centre of Public Health of the Ministry of Health of the Republic of Moldova, United Nations Children's Fund (UNICEF) (2014). 2012 Republic of Moldova Multiple Indicator Cluster Survey, Final Report. Chișinău, Republic of Moldova: UNICEF.
		Studiu Kap (2014). Campania Nationala de Informare Privind Reducerea Consumului de alcool. Moldova: Magenta Consulting.
		World Health Organization (2013c). STEPwise approach to surveillance (STEPS) Survey. (http://www.who.int/chp/steps/en/. Accessed: 01/15/2018).
		World Health Organization Regional Office for Europe, Ministry of Health, Labour and Social Protection - Republic of Moldova (2017). KAP Survey: Knowledge, Attitudes, and Practices Regarding Alcohol Consumption phase I-IV. Copenhagen, Denmark: World Health Organization Regional Office for Europe.
EUR	Romania	ESPAD Group (2020). ESPAD Report 2019: Results from the European School Survey Project on Alcohol and Other Drugs. Luxembourg: EMCDDA Joint Publications, Publications Office of the European Union.
		European Monitoring Centre for Drugs and Drug Addiction, National Anti-Drug Agency (2011). Romania National Report to the EMCDDA 2011. Lisbon, Portugal: European Monitoring Centre for Drugs and Drug Addiction.
		European Monitoring Centre for Drugs and Drug Addiction (2016). ESPAD Report 2015. Results from the European School Survey Project on Alcohol and Other Drugs. http://www.espad.org/sites/espad.org/files/ESPAD_report_2015.pdf. Accessed: 06/15/2017
		European Monitoring Centre for Drugs and Drug Addiction (2020). Statistical Bulletin 2020 – prevalence of drug use: Alcohol Data tables - lifetime prevalence, last year prevalence (https://www.emcdda.europa.eu/data/stats2020/gps. Accessed: 07/21/2020).
		European Monitoring Centre for Drugs and Drug Addiction, European School Survey Project on Alcohol and Other Drugs (ESPAD), Pompidou Group, Council of Europe, Swedish Council for Information on Alcohol and Other Drugs (Can) (2012). ESPAD report 2011: Substance Use Among Students in 36 European Countries. Stockholm, Sweden: Swedish Council for Information on Alcohol and Other Drugs (CAN).
		Inchley J, Currie D, Budisavljevic S, Torsheim T, Jåstad A, Cosma A, et al. (2020). Spotlight on adolescent health and well-being. Findings from the 2017/2018 Health Behaviour in School-aged Children (HBSC) survey in Europe and Canada. International report. Volume 2. Key data. Copenhagen, Denmark: WHO Regional Office for Europe.
		Moskalewicz J, Room R, Thom B (2016). Comparative monitoring of alcohol epidemiology across the EU: Baseline assessment and suggestions for future action. Synthesis report. Warszawa, Poland: Joint Action on Reducing Alcohol Related Harm (RARHA).

Region	Country	Sources of alcohol patterns of consumption data by WHO Member State
		Statistical Office of the European Union (Eurostat) (2019). European Health Interview Survey Wave 2 data table: Frequency of alcohol consumption by sex, age and educational attainment level. (https://ec.europa.eu/eurostat/databrowser/view/hlth_ehis_al1e/default/table?lang=en. Accessed: 08/17/2020).
		Statistical Office of the European Union (Eurostat) (2019). European Health Interview Survey Wave 2 data table: Frequency of heavy episodic drinking by sex, age and educational attainment level. (https://ec.europa.eu/eurostat/databrowser/view/hlth_ehis_al3e/default/table?lang=en. Accessed: 08/17/2020).
EUR	Russian Federation	Abe S, Stickley A, Roberts B, Richardson E, Abbott P, Rotman D, et al. (2013). Changing patterns of fruit and vegetable intake in countries of the former Soviet Union, Public Health Nutrition.16(11):1924-1932.
		Inchley J, Currie D, Budisavljevic S, Torsheim T, Jåstad A, Cosma A, et al. (2020). Spotlight on adolescent health and well-being. Findings from the 2017/2018 Health Behaviour in School-aged Children (HBSC) survey in Europe and Canada. International report. Volume 2. Key data. Copenhagen, Denmark: WHO Regional Office for Europe.
		National Research University Higher School of Economics, ZAO "Demoscope", Carolina Population Center (2014). Russian Longitudinal Monitoring Survey 2008. (https://www.hse.ru/en/rlms/. Accessed: 06/26/2017).
		National Research Institute of Public Health, Russian Academy of Medical Sciences (2013). Study on global AGEing and adult health (SAGE) Wave 1: Russian Federal National Report. (https://www.who.int/healthinfo/sage/national_reports/en/. Accessed: 08/11/2020).
		Perlman FJ (2010). Drinking in transition: trends in alcohol consumption in Russia 1994-2004, BMC Public Health.10:691.
		Popkin B (2016). RLMS-HSE Household and Individual Data 1994-2019. (https://dataverse.unc.edu/file.xhtml?fileId=7498721&version=11.0. Accessed: 07/03/2020).
		Roberts B, Stickley A, Gilmore AB, Danishevski K, Kizilova K, Bryden A, et al. (2013). Knowledge of the health impacts of smoking and public attitudes towards tobacco control in the former Soviet Union, Tobacco Control.22(6):e12.
		Rose R (2010). New Russia barometer XV, April 2007. (https://discover.ukdataservice.ac.uk/Catalogue/?sn=6441&type=Data%20catalogue. Accessed: 06/28/2017).
		Stickley A, Koyanagi A, Roberts B, Murphy A, Kizilova K, Mckee M (2015). Male solitary drinking and hazardous alcohol use in nine countries of the former Soviet Union, Drug and Alcohol Dependence.150:105-111.
		Vlasoff T, Laatikainen T, Korpelainen V, Uhanov M, Pokusajeva S, Tossavainen K, et al. (2015). Trends and educational differences in non-communicable disease risk factors in Pitkaranta, Russia, from 1992 to 2007, Scandanavian Journal of Public Health.43(1):91-98.
EUR	San Marino	-
EUR	Serbia	Boričić K, Vasić M, Grozdanov J, Rakić JG, Šulović MZ, Knežević NJ, et al. (2014). Results of the National Health Survey of the Republic of Serbia 2013. Belgrade: Republic of Serbia. Ministry of Health, The Institute of Public Health of Serbia.
		ESPAD Group (2020). ESPAD Report 2019: Results from the European School Survey Project on Alcohol and Other Drugs. Luxembourg: EMCDDA Joint Publications, Publications Office of the European Union.
		European Monitoring Centre for Drugs and Drug Addiction (2016). ESPAD report 2015. Results from the European School Survey Project on Alcohol and Other Drugs. (http://www.espad.org/sites/espad.org/files/ESPAD_report_2015.pdf. Accessed: 06/15/2017).
		Inchley J, Currie D, Budisavljevic S, Torsheim T, Jåstad A, Cosma A, et al. (2020). Spotlight on adolescent health and well-being. Findings from the 2017/2018 Health Behaviour in School-aged Children (HBSC) survey in Europe and Canada. International report. Volume 2. Key data. Copenhagen, Denmark: WHO Regional Office for Europe.
		Kilibarda B, Mravcik V, Sieroslawski J, Gudelj Rakić J, Martens MS (2014). National survey on life styles of citizens in Serbia 2014 - Key findings on substance use and gambling. Serbia: Institute of Public Health of Serbia.
EUR	Slovakia	ESPAD Group (2020). ESPAD Report 2019: Results from the European School Survey Project on Alcohol and Other Drugs. Luxembourg: EMCDDA Joint Publications, Publications Office of the European Union.
		European Monitoring Centre for Drugs and Drug Addiction (2016). ESPAD report 2015. Results from the European School Survey Project on Alcohol and Other Drugs. (http://www.espad.org/sites/espad.org/files/ESPAD_report_2015.pdf. Accessed: 06/15/2017).
		European Monitoring Centre for Drugs and Drug Addiction (2020). Statistical Bulletin 2020 – prevalence of drug use: Alcohol Data tables - lifetime prevalence, last year prevalence (https://www.emcdda.europa.eu/data/stats2020/gps. Accessed: 07/21/2020).
		European Monitoring Centre for Drugs and Drug Addiction, European School Survey Project on Alcohol and Other Drugs (ESPAD), Pompidou Group, Council of Europe, Swedish Council for Information on Alcohol and Other Drugs (Can) (2012). ESPAD report 2011: Substance Use Among Students in 36 European Countries. Stockholm, Sweden: Swedish Council for Information on Alcohol and Other Drugs (CAN).
		Inchley J, Currie D, Budisavljevic S, Torsheim T, Jåstad A, Cosma A, et al. (2020). Spotlight on adolescent health and well-being. Findings from the 2017/2018 Health Behaviour in School-aged Children (HBSC) survey in Europe and Canada. International report. Volume 2. Key data. Copenhagen, Denmark: WHO Regional Office for Europe.
		Statistical Office of the European Union (Eurostat) (2019). European Health Interview Survey Wave 2 data table: Frequency of alcohol consumption by sex, age and educational attainment level. (https://ec.europa.eu/eurostat/databrowser/view/hlth_ehis_al1e/default/table?lang=en. Accessed: 08/17/2020).
		Statistical Office of the European Union (Eurostat) (2019). European Health Interview Survey Wave 2 data table: Frequency of heavy episodic drinking by sex, age and educational attainment level. (https://ec.europa.eu/eurostat/databrowser/view/hlth_ehis_al3e/default/table?lang=en. Accessed: 08/17/2020).
EUR	Slovenia	Artnik B, Bajt M, Bilban M, Borovničar A, Brguljan Hitij J, Djomba JK, et al. (2012). Zdravje in vedenjski slog prebivalcev Slovenije: trendi v raziskavah CINDI 2001-2004-2008. Ljubljana: Inštitut za varovanje zdravja.
		ESPAD Group (2020). ESPAD Report 2019: Results from the European School Survey Project on Alcohol and Other Drugs. Luxembourg: EMCDDA Joint Publications, Publications Office of the European Union.
		European Monitoring Centre for Drugs and Drug Addiction (2016). ESPAD report 2015. Results from the European School Survey Project on Alcohol and Other Drugs. (http://www.espad.org/sites/espad.org/files/ESPAD_report_2015.pdf. Accessed: 06/15/2017).

Region	Country	Sources of alcohol patterns of consumption data by WHO Member State
		European Monitoring Centre for Drugs and Drug Addiction (2020). Statistical Bulletin 2020 – prevalence of drug use: Alcohol Data tables - lifetime prevalence, last year prevalence (https://www.emcdda.europa.eu/data/stats2020/gps. Accessed: 07/21/2020).
		European Monitoring Centre for Drugs and Drug Addiction, European School Survey Project on Alcohol and Other Drugs (ESPAD), Pompidou Group, Council of Europe, Swedish Council for Information on Alcohol and Other Drugs (Can) (2012). ESPAD report 2011: Substance Use Among Students in 36 European Countries. Stockholm, Sweden: Swedish Council for Information on Alcohol and Other Drugs (CAN).
		Hovnik KM, Zorko M, Macur M (2015). Uporaba tobaka, alkohola in prepovedanih drog med prebivalci Slovenije ter neenakosti in kombinacije te uporabe. Lubljana: Nacionalni inštituta za javno zdravje.
		Inchley J, Currie D, Budisavljevic S, Torsheim T, Jåstad A, Cosma A, et al. (2020). Spotlight on adolescent health and well-being. Findings from the 2017/2018 Health Behaviour in School-aged Children (HBSC) survey in Europe and Canada. International report. Volume 2. Key data. Copenhagen, Denmark: WHO Regional Office for Europe.
		Statistical Office of the European Union (Eurostat) (2019). European Health Interview Survey Wave 2 data table: Frequency of alcohol consumption by sex, age and educational attainment level. (https://ec.europa.eu/eurostat/databrowser/view/hlth_ehis_al1e/default/table?lang=en. Accessed: 08/17/2020).
		Statistical Office of the European Union (Eurostat) (2019). European Health Interview Survey Wave 2 data table: Frequency of heavy episodic drinking by sex, age and educational attainment level. (https://ec.europa.eu/eurostat/databrowser/view/hlth_ehis_al3e/default/table?lang=en. Accessed: 08/17/2020).
		Tomšič S, Bric TK, Korošec A, Zakotnik JM (2014). Izzivi v izboljševanju vedenjskega sloga in zdravja - desetletje CINDI raziskav v Sloveniji. Ljubljana: Nacionalni inštitut za javno zdravje.
		Vinko M, Bric TK, Korošec A, Tomšič S, Vrdelja M (2018). Kako skrbimo za zdravje? Z zdravjem povezan vedenjski slog prebivalcev Slovenije 2016. (https://www.nijz.si/sites/www.nijz.si/files/publikacije-datoteke/kako_skrbimo_za_zdravje_splet_3007_koncna.pdf. Accessed: 06/10/2020).
EUR	Spain	Bloomfield K, Allamani A, Beck F, Bergmark KH, Csemy L, Eisenbach-Stangl I, et al. (2005). Gender, culture and alcohol problems : a multi-national study. An EU concerted Action. Project final report. Berlin, Germany: Charite Campus Benjamin Franklin.
		ESPAD Group (2020). ESPAD Report 2019: Results from the European School Survey Project on Alcohol and Other Drugs. Luxembourg: EMCDDA Joint Publications, Publications Office of the European Union.
		Galán I, González MJ, Valencia-Martin JL (2014). Patrones de consumo de alcohol en España: un país en transición, Revista espanola de salud publica.88(4):529-40.
		Inchley J, Currie D, Budisavljevic S, Torsheim T, Jåstad A, Cosma A, et al. (2020). Spotlight on adolescent health and well-being. Findings from the 2017/2018 Health Behaviour in School-aged Children (HBSC) survey in Europe and Canada. International report. Volume 2. Key data. Copenhagen, Denmark: WHO Regional Office for Europe.
		Instituto Nacional de Estadística (2017). Encuesta nacional de salud 2017: Resultados. Determinantes de la salud, cifras absolutas:Consumo de bebidas alcohólicas. (https://www.ine.es/dynt3/inebase/index.htm?type=pcaxis&path=/t15/p419/a2017/p03/&file=pcaxis. Accessed: 10/16/2020).
		León-Muñoz LM, Sánchez-Alonso F, Valencia-Martín J, López-García E, Rodríguez-Artalejo F (2014). Patterns of alcohol consumption in the older population of Spain, 2008-2010, Journal of Academic Nutrition and Diet.115(2):213-214.
		Llorens N, Barrio G, Sanchez A, Suelves JM (2011). Effects of socialization and family factors on adolescent excessive drinking in Spain, Prevention Science.12(2):150-161.
		Ministerio de Sanidad, Servicios Sociales e Igualdad (2013). Encuesta Nacional de Salud. España 2011/12. Serie Informes monográficos nº 1. Consumo de alcohol. (https://www.mscbs.gob.es/estadEstudios/estadisticas/encuestaNacional/encuestaNac2011/informesMonograficos/ENSE2011_12_MONOGRAFICO_1_ALCOHOL4.pdf. Accessed: 10/28/2019).
		Moskalewicz J, Room R, Thom B (2016). Comparative monitoring of alcohol epidemiology across the EU: Baseline assessment and suggestions for future action. Synthesis report. Warszawa, Poland: Joint Action on Reducing Alcohol Related Harm (RARHA).
		Observatorio Español de las Drogas y las Adicciones (2019). INFORME 2020 Alcohol, tabaco y drogas ilegales en España: Encuesta sobre uso de drogas en Enseñanzas Secundarias en España (ESTUDES), 1994-2018/2019. (https://pnsd.sanidad.gob.es/profesionales/sistemasInformacion/sistemaInformacion/pdf/ESTUDES_2020_Informe.pdf. Accessed: 01/21/2021).
		Plan Nacional sobre Drogas (2011). EDADES 2011 encuesta sobre alcohol y drogas en España. (http://www.pnsd.msssi.gob.es/profesionales/sistemasInformacion/sistemaInformacion/pdf/4_EDADES_2011_Informe.pdf. Accessed: 01/12/2018).
		Plan Nacional sobre Drogas (2013). EDADES 2013 encuesta sobre alcohol y drogas en España. (http://www.pnsd.msssi.gob.es/profesionales/sistemasInformacion/sistemaInformacion/pdf/2015_Informe_EDADES.pdf Accessed: 01/12/2018).
		Plan Nacional sobre Drogas (2016). EDADES 2015-2016 encuesta sobre alcohol y drogas en España. (http://www.pnsd.msssi.gob.es/profesionales/sistemasInformacion/sistemaInformacion/pdf/2015_EDADES__Informe_.pdf Accessed: 01/12/2018).
		Plan Nacional sobre Drogas (2018). EDADES 2017 Encuesta sobre alcohol y drogas en España. (https://pnsd.sanidad.gob.es/profesionales/sistemasInformacion/sistemaInformacion/pdf/EDADES_2017_Informe.pdf. Accessed: 08/12/2019).
		Plan Nacional sobre Drogas (2020). EDADES 2019/2020: Encuesta sobre alcohol, drogas y otras adicciones en España/ ESDAM 2019/2020 Principales resultados (https://pnsd.sanidad.gob.es/profesionales/sistemasInformacion/sistemaInformacion/pdf/EDADES_2019-2020_resumenweb.pdf. Accessed: 01/21/2021).
		Soler-Vila H, Galán I, Valencia-Martín JL, León-Muñoz LM, Guallar-Castillón P, Rodríguez-Artalejo F (2014). Binge Drinking in Spain, 2008–2010, Alcoholism: Clinical and Experimental Research.38(3):810-9. https://doi.org/10.1111/acer.12275.

Region	Country	Sources of alcohol patterns of consumption data by WHO Member State
		Statistical Office of the European Union (Eurostat) (2019). European Health Interview Survey Wave 2 data table: Frequency of alcohol consumption by sex, age and educational attainment level. (https://ec.europa.eu/eurostat/databrowser/view/hlth_ehis_al1e/default/table?lang=en. Accessed: 08/17/2020).
		Statistical Office of the European Union (Eurostat) (2019). European Health Interview Survey Wave 2 data table: Frequency of heavy episodic drinking by sex, age and educational attainment level. (https://ec.europa.eu/eurostat/databrowser/view/hlth_ehis_al3e/default/table?lang=en. Accessed: 08/17/2020).
		The International GENACIS-Project (2003). GENACIS - gender, alcohol and culture: an international study. (http://www.genacis.org/. Accessed: 01/10/2018).
EUR	Sweden	Abidi, L., Nilsen, P., Karlsson, N., Skagerström, J., O'Donnell, A. (2020). Conversations about alcohol in healthcare – cross-sectional surveys in the Netherlands and Sweden. (https://doi.org/10.1186/s12889-020-8367-8. Accessed: 11/13/2020).
		Andersson B, Hibell B (2003). Alcohol and drug use among European 17–18 year old students. ESPAD 2003. Stockholm, Sweden: Modintryckoffset AB.
		Andersson A, Andersson C, Holmgren K, Ma°Rdby AC, Hensing G (2012). Participation in leisure activities and binge drinking in adults: Findings from a Swedish general population sample, Addiction Research & Theory.20(2):172-182.
		Bloomfield K, Allamani A, Beck F, Bergmark KH, Csemy L, Eisenbach-Stangl I, et al. (2005). Gender, culture and alcohol problems : a multi-national study. An EU concerted Action. Project final report. Berlin, Germany: Charite Campus Benjamin Franklin.
		Eliasen M, Kjær SK, Munk C, Nygård M, Sparén P, Tryggvadottir L, et al. (2009). The relationship between age at drinking onset and subsequent binge drinking among women, European Journal of Public Health.19(4):378-382.
		ESPAD Group (2020). ESPAD Report 2019: Results from the European School Survey Project on Alcohol and Other Drugs. Luxembourg: EMCDDA Joint Publications, Publications Office of the European Union.
		European Monitoring Centre for Drugs and Drug Addiction (2020). Statistical Bulletin 2020 – prevalence of drug use: Alcohol Data tables - lifetime prevalence, last year prevalence (https://www.emcdda.europa.eu/data/stats2020/gps. Accessed: 07/21/2020).
		European Monitoring Centre for Drugs and Drug Addiction, European School Survey Project on Alcohol and Other Drugs (ESPAD), Pompidou Group, Council of Europe, Swedish Council for Information on Alcohol and Other Drugs (Can) (2012). ESPAD report 2011: Substance use among students in 36 European countries. Stockholm, Sweden: Swedish Council for Information on Alcohol and Other Drugs (CAN).
		Hibell B, Andersson B, Bjarnason T, Kokkevi A, Morgan M, Narusk A (1997). The 1995 ESPAD report. Alcohol and Other Drug Use among Students in 26 European Countries. Stockholm, Sweden: The Swedish Council for Information on Alcohol and Other Drugs.
		Inchley J, Currie D, Budisavljevic S, Torsheim T, Jåstad A, Cosma A, et al. (2020). Spotlight on adolescent health and well-being. Findings from the 2017/2018 Health Behaviour in School-aged Children (HBSC) survey in Europe and Canada. International report. Volume 2. Key data. Copenhagen, Denmark: WHO Regional Office for Europe.
		Moskalewicz J, Room R, Thom B (2016). Comparative monitoring of alcohol epidemiology across the EU: Baseline assessment and suggestions for future action. Synthesis report. Warszawa, Poland: Joint Action on Reducing Alcohol Related Harm (RARHA).
		Statistical Office of the European Union (Eurostat) (2019). European Health Interview Survey Wave 2 data table: Frequency of alcohol consumption by sex, age and educational attainment level. (https://ec.europa.eu/eurostat/databrowser/view/hlth_ehis_al1e/default/table?lang=en. Accessed: 08/17/2020).
		Statistical Office of the European Union (Eurostat) (2019). European Health Interview Survey Wave 2 data table: Frequency of heavy episodic drinking by sex, age and educational attainment level. (https://ec.europa.eu/eurostat/databrowser/view/hlth_ehis_al3e/default/table?lang=en. Accessed: 08/17/2020).
		Statistiska Centralbyran (2016). Teknisk Rapport - En beskrivning av genomförande och metoder. (https://www.folkhalsomyndigheten.se/folkhalsorapportering-statistik/statistikdatabaser-och-visualisering/nationella-folkhalsoenkaten/levnadsvanor/alkoholvanor/. Accessed: 06/28/2017).
		The Public Health Agency of Sweden (2016a). Alcohol, narcotics, doping, tobacco and gambling. (https://www.folkhalsomyndigheten.se/the-public-health-agency-of-sweden/living-conditions-and-lifestyle/alcohol-narcotics-doping-tobacco-and-gambling/. Accessed: 10/11/2017).
		The Public Health Agency of Sweden (2016b). Alkoholvanor [drinking habits] Alkoholvanor – nationella resultat och tidsserier 2016 [Drinking habits - National Results and Time Series 2016]. (https://www.folkhalsomyndigheten.se/folkhalsorapportering-statistik/statistikdatabaser-och-visualisering/nationella-folkhalsoenkaten/levnadsvanor/alkoholvanor/. Accessed: 07/20/2017).
		The International GENACIS-Project (2000). GENACIS - gender, alcohol and culture: an international study. (http://www.genacis.org/. Accessed: 01/11/2018).
		The International GENACIS-Project (2002). GENACIS - gender, alcohol and culture: an international study. (http://www.genacis.org/. Accessed: 01/11/2018).
EUR	Switzerland	Bachmann N, Burla L, Kohler D (2015). Gesundheit in der Schweiz –Fokus chronische Erkrankungen - Nationaler Gesundheitsbericht 2015. Bern, Switzerland: Hogrefe Verlag.
		Bloomfield K, Allamani A, Beck F, Bergmark KH, Csemy L, Eisenbach-Stangl I, et al. (2005). Gender, culture and alcohol problems : a multi-national study. An EU concerted Action. Project final report. Berlin, Germany: Charite Campus Benjamin Franklin.
		Gmel G, Kuendig H, Notari L, Gmel C (2014). Suchtmonitoring Schweiz - Konsum von Alkohol, Tabak und illegalen Drogen in der Schweiz im Jahr 2013. Lausanne, Schweiz: Schweiz, S.
		Gmel G, Kuendig H, Notari L, Gmel C (2015). Suchtmonitoring Schweiz - Konsum von Alkohol, Tabak und illegalen Drogen in der Schweiz im Jahr 2014. (https://www.suchtmonitoring.ch/docs/library/gmel_8tznggnu81d0.pdf. Accessed: 10/30/2019).
		Gmel G, Kuendig H, Notari L, Gmel C (2016). Suchtmonitoring Schweiz - Konsum von Alkohol, Tabak und illegalen Drogen in der Schweiz im Jahr 2015. (https://www.suchtmonitoring.ch/docs/library/gmel_thezckxvvkq4.pdf. Accessed: 10/30/2019).
		Gmel G, Kuendig H, Notari L, Gmel C (2017). Suchtmonitoring Schweiz - Konsum von Alkohol, Tabak und illegalen Drogen in der Schweiz im Jahr 2016. (https://www.suchtmonitoring.ch/docs/library/gmel_5lbj5rqv9y5i.pdf. Accessed: 10/30/2019).

Region	Country	Sources of alcohol patterns of consumption data by WHO Member State
		Gmel G, Kuendig H, Notari L, Gmel C, Flury R (2013). Suchtmonitoring Schweiz - Konsum von Alkohol in der Schweiz im Jahr 2012. (https://www.suchtmonitoring.ch/docs/library/gmel_ewas4ahd54vo.pdf. Accessed: 10/30/2019).
		Gmel G, Notari L, Georges A, Wikcki M (2012). Alkohol, Suchtmonitoring Schweiz / Jahresbericht– Daten 2011. (https://www.suchtmonitoring.ch/docs/library/gmel_mlfzur6ih7hz.pdf. Accessed: 11/21/2019).
		Inchley J, Currie D, Budisavljevic S, Torsheim T, Jåstad A, Cosma A, et al. (2020). Spotlight on adolescent health and well-being. Findings from the 2017/2018 Health Behaviour in School-aged Children (HBSC) survey in Europe and Canada. International report. Volume 2. Key data. Copenhagen, Denmark: WHO Regional Office for Europe.
		Office fédéral de la statistique (2019). Consommation d'alcool en 2017. Neuchâtel: Office fédéral de la statistique.
		Schweizerische Eidgenossenschaft, Federal Department of Home Affaires, Federal Statistical Office (2013). Swiss Health Survey 2012 - Overview. Neuchâtel: Schweizerische Eidgenossenschaft.
		The International GENACIS-Project (1997). GENACIS - gender, alcohol and culture: an international study. (http://www.genacis.org/. Accessed: 01/11/2018).
EUR	Tajikistan	Centers for Disease Control and Prevention, UNICEF, World Health Organization (2006). GSHS Country Report : Global School-based Student Health Survey Tajikistan. Atlanta, Georgia: Centers for Disease Control and Prevention, UNICEF, World Health Organization.
EUR	The former Yugoslav Republic of Macedonia	ESPAD Group (2020). ESPAD Report 2019: Results from the European School Survey Project on Alcohol and Other Drugs. Luxembourg: EMCDDA Joint Publications, Publications Office of the European Union.
		European Monitoring Centre for Drugs and Drug Addiction (2016). ESPAD report 2015. Results from the European School Survey Project on Alcohol and Other Drugs. (http://www.espad.org/sites/espad.org/files/ESPAD_report_2015.pdf Accessed: 06/15/2017).
		Inchley J, Currie D, Budisavljevic S, Torsheim T, Jåstad A, Cosma A, et al. (2020). Spotlight on adolescent health and well-being. Findings from the 2017/2018 Health Behaviour in School-aged Children (HBSC) survey in Europe and Canada. International report. Volume 2. Key data. Copenhagen, Denmark: WHO Regional Office for Europe.
		Ministry of Health, Ministry of Education and Science, and Ministry of Labour and Social Policy of the Government of Republic of Macedonia, UNICEF (2012). Republic of Macedonia Multiple Indicator Cluster Survey 2011. (https://www.unicef.org/northmacedonia/reports/multiple-indicator-cluster-survey-2011. Accessed: 06/24/2019).
EUR	Turkiye	European Monitoring Centre for Drugs and Drug Addiction (2020). Statistical Bulletin 2020 – prevalence of drug use: Alcohol Data tables - lifetime prevalence, last year prevalence (https://www.emcdda.europa.eu/data/stats2020/gps. Accessed: 07/21/2020).
		General Directorate of Health Research (2015). Sağlık İstatistikleri Yıllığı 2014 [Health Statistics Yearbook 2014] In: Kose, M. R., Güler, C. & Yentür, G. K. (eds.). Ankara: General Directorate of Health Research.
		Republic of Turkey, Ministry of Interior, Turkish National Police, Anti-Smuggling and Organized Crime Department (2010). Turkey - new developments, trends and in-depth information on selected issues. (http://www.emcdda.europa.eu/attachements.cfm/att_142576_EN_TR-NR2010.pdf Accessed: 07/19/2017).
		Statistical Office of the European Union (Eurostat) (2019). European Health Interview Survey Wave 2 data table: Frequency of alcohol consumption by sex, age and educational attainment level. (https://ec.europa.eu/eurostat/databrowser/view/hlth_ehis_al1e/default/table?lang=en. Accessed: 08/17/2020).
		Statistical Office of the European Union (Eurostat) (2019). European Health Interview Survey Wave 2 data table: Frequency of heavy episodic drinking by sex, age and educational attainment level. (https://ec.europa.eu/eurostat/databrowser/view/hlth_ehis_al3e/default/table?lang=en. Accessed: 08/17/2020).
		Türkiye İstatistik Kurumu (2014). TurkSTAT User Survey Results 2014. (www.turkstat.gov.tr/PreIstatistikTablo.do?istab_id=2395. Accessed: 07/19/2017).
		Türkiye İstatistik Kurumu (2020). The percentage of individuals' status of consuming alcoholic drinks by sex and age groups – data table generated online. (https://data.tuik.gov.tr/Kategori/GetKategori?p=saglik-ve-sosyal-koruma-101&dil=2. Accessed: 06/11/2020).
		Ünal B, Ergör G, Dinç-Horasan G, Kalaça S, Sözmen K. (2013). Chronic diseases and risk factors survey in Turkey. Ankara, Turkey: Ministry of Health.
		Üner S, Balcılar M, Ergüder T (2018). National Household Health Survey – Prevalence of Noncommunicable Disease Risk Factors in Turkey 2017 (STEPS). Ankara, Turkey: World Health Organization Country Office in Turkey.
EUR	Turkmenistan	World Health Organization (2013c). STEPwise approach to surveillance (STEPS) Survey. (http://www.who.int/chp/steps/en/. Accessed: 01/15/2018).
		World Health Organization (2018). STEPwise approach to surveillance (STEPS) Survey. (http://www.who.int/chp/steps/en/. Accessed: 07/02/2020).
EUR	Ukraine	Abe S, Stickley A, Roberts B, Richardson E, Abbott P, Rotman D, et al. (2013). Changing patterns of fruit and vegetable intake in countries of the former Soviet Union, Public Health Nutrition.16(11):1924-1932.
		ESPAD Group (2020). ESPAD Report 2019: Results from the European School Survey Project on Alcohol and Other Drugs. Luxembourg: EMCDDA Joint Publications, Publications Office of the European Union.
		European Monitoring Centre for Drugs and Drug Addiction, European School Survey Project on Alcohol and Other Drugs (ESPAD), Pompidou Group, Council of Europe & Swedish Council for Information on Alcohol and Other Drugs (Can) (2012). ESPAD report 2011: substance use among students in 36 European countries. Stockholm, Sweden: Swedish Council for Information on Alcohol and Other Drugs (CAN).
		Inchley J, Currie D, Budisavljevic S, Torsheim T, Jåstad A, Cosma A, et al. (2020). Spotlight on adolescent health and well-being. Findings from the 2017/2018 Health Behaviour in School-aged Children (HBSC) survey in Europe and Canada. International report. Volume 2. Key data. Copenhagen, Denmark: WHO Regional Office for Europe.
		Krasovsky KS (2016). Alcohol consumption and alcohol related harm in Ukraine / Economy and legislation of health care. (http://irbis-nbuv.gov.ua/cgi-bin/irbis_nbuv/cgiirbis_64.exe?C21COM=2&I21DBN=UJRN&P21DBN=UJRN&IMAGE_FILE_DOWNLOAD=1&Image_file_name=PDF/eprozd_2016_1_4.pdf. Accessed: 12/06/2017).
		State Statistics Service, Ukrainian Center for Social Reforms (2013). Ukraine Multiple Indicator Cluster Survey 2012, Final Report. Kyiv, Ukraine: State Statistics Committee, Ukrainian Center for Social Reforms.

Region	Country	Sources of alcohol patterns of consumption data by WHO Member State
		World Health Organization (2019d). STEPwise approach to surveillance (STEPS) Survey. (http://www.who.int/chp/steps/en/. Accessed: 11/12/2020).
EUR	United Kingdom of Great Britain and Northern Ireland	Bellis MA, Hughes K, Jones L, Morleo M, Nicholls J, McCoy E, et al. (2015). Holidays, celebrations, and commiserations: measuring drinking during feasting and fasting to improve national and individual estimates of alcohol consumption, BMC medicine.13:113. https://dx.doi.org/10.1186/s12916-015-0337-0.
		Bloomfield K, Allamani A, Beck F, Bergmark KH, Csemy L, Eisenbach-Stangl I, et al. (2005). Gender, culture and alcohol problems : a multi-national study. An EU concerted Action. Project final report. Berlin, Germany: Charite Campus Benjamin Franklin.
		Case P, Ng Fat L, Shelton N (2019). Exploring the characteristics of newly defined at-risk drinkers following the change to the UK low risk drinking guidelines: a retrospective analysis using Health Survey for England data, BMC public health.19(1):902. https://dx.doi.org/10.1186/s12889-019-7240-0.
		Lifestyle Statistics Team, Health and Social Care Information Centre. (2013). Statistics on Alcohol: England, 2013. Health and Social Care Information Centre. https://digital.nhs.uk/data-and-information/publications/statistical/statistics-on-alcohol
		Lifestyle Statistics Team, Health and Social Care Information Centre. (2015). Statistics on Alcohol: England, 2015. Health and Social Care Information Centre. https://digital.nhs.uk/data-and-information/publications/statistical/statistics-on-alcohol
		Stats Tean, NHS Digital. (2017). Statistics on Alcohol: England, 2017. NHS Digital. https://digital.nhs.uk/data-and-information/publications/statistical/statistics-on-alcohol
		Stats Tean, NHS Digital. (2018). Statistics on Alcohol: England, 2018. NHS Digital. https://digital.nhs.uk/data-and-information/publications/statistical/statistics-on-alcohol
		Inchley J, Currie D, Budisavljevic S, Torsheim T, Jåstad A, Cosma A, et al. (2020). Spotlight on adolescent health and well-being. Findings from the 2017/2018 Health Behaviour in School-aged Children (HBSC) survey in Europe and Canada. International report. Volume 2. Key data. Copenhagen, Denmark: WHO Regional Office for Europe. http://www.hbsc.org/
		Moskalewicz J, Room R, Thom B (2016). Comparative monitoring of alcohol epidemiology across the EU: Baseline assessment and suggestions for future action. Synthesis report. Warszawa, Poland: Joint Action on Reducing Alcohol Related Harm (RARHA).
		NatCen Social Research (2015). Welsh Health Survey, 2013. London: NatCen Social Research. https://natcen.ac.uk/our-research/research/welsh-health-survey/
		ScotCen Social Research (2016). Scottish Health Survey, 2013, 3rd Edition. (http://doi.org/10.5255/UKDA-SN-7594-3. Accessed: 06/15/2017).
		Statistical Office of the European Union (Eurostat) (2019). European Health Interview Survey Wave 2 data table: Frequency of alcohol consumption by sex, age and educational attainment level. (https://ec.europa.eu/eurostat/databrowser/view/hlth_ehis_al1e/default/table?lang=en. Accessed: 08/17/2020).
		Statistical Office of the European Union (Eurostat) (2019). European Health Interview Survey Wave 2 data table: Frequency of heavy episodic drinking by sex, age and educational attainment level. (https://ec.europa.eu/eurostat/databrowser/view/hlth_ehis_al3e/default/table?lang=en. Accessed: 08/17/2020).
		The International GENACIS-Project (2000). GENACIS - gender, alcohol and culture: an international study. (http://www.genacis.org/. Accessed: 01/11/2018).
EUR	Uzbekistan	World Health Organization (2014b). STEPwise approach to surveillance (STEPS) Survey. (http://www.who.int/chp/steps/en/. Accessed: 01/12/2018).
SEAR	Bangladesh	World Health Organization (2010). STEPwise approach to surveillance (STEPS) Survey. (https://extranet.who.int/ncdsmicrodata/index.php/catalog/STEPS. Accessed: 07/02/2020).
		World Health Organization (2020a). STEPwise approach to surveillance (STEPS) Survey. (https://extranet.who.int/ncdsmicrodata/index.php/catalog/770. Accessed: 11/12/2020).
		World Health Organization, Centers for Disease Control and Prevention (2014). Global School-based Student Health Survey – Bangladesh 2014 Fact Sheet. (https://nada.searo.who.int/index.php/catalog/33. Accessed: 06/22/2020)
		Zaman MM, Bhuiyan MR, Huq SM, Rahman MM, Sinha DN, Fernando T (2014). Dual use of tobacco among Bangladeshi men, Indian Journal of Cancer.51(Supplement 1):S46-S9. http://dx.doi.org/10.4103/0019-509X.147481.
SEAR	Bhutan	Dorji L, National Statistics Bureau of Bhutan (2012). Alcohol use and abuse in Bhutan. Bhutan: National Statistics Bureau.
		Ministry of Health (2012). National Health Survey. Thimpu, Bhutan: Ministry of Health.
		World Health Organization (2007). STEPwise approach to surveillance (STEPS) Survey. (http://www.who.int/chp/steps/en/. Accessed: 01/11/2018).
		World Health Organization (2014b). STEPwise approach to surveillance (STEPS) Survey. (http://www.who.int/chp/steps/en/. Accessed: 01/12/2018).
SEAR	Democratic People's Republic of Korea	-
SEAR	India	Ambekar A, Agrawal A, Rao R, Mishra AK, Khandelwal SK, Chadda RK (2019). Magnitude of Substance Use in India. New Delhi: Ministry of Social Justice and Empowerment, Government of India.
		Arokiasamy P, Parasuraman S, Sekher TV, Lhungdim H (2013). Study on global AGEing and adult health (SAGE) Wave 1 - India National Report. World Health Organization.
		Greenfield TK, Bloomfield K, Wilsnack SC (2014a). GENAHTO Project (Gender and Alcohol’s Harm to Others). (http://genahto.org/. Accessed: 07/02/2020).
		Kumar K, Kumar S, Singh AK (2018). Prevalence and socio-demographic correlates of alcohol consumption: Survey findings from five states in India, Drug and alcohol dependence.185:381-90.
		National Family Health Survey. (2009). NFHS-4 Fact Sheets for Key Indicators Based on Final Data. (http://rchiips.org/nfhs/factsheet_NFHS-4.shtml. Accessed: 06/16/2017).
		Thakur J, Jeet G, Nangia R, Singh D, Grover S, Lyngdoh T, et al. (2019). Non-communicable diseases risk factors and their determinants: A cross-sectional state-wide STEPS survey, Haryana, North India, PLOS ONE.14(11): e0208872. https://doi.org/10.1371/journal.pone.0208872.
		Thakur JS, Jeet G, Pal A, Singh S, Singh A, Deepti SS, et al. (2016). Profile of Risk Factors for Non-Communicable Diseases in Punjab, Northern India: Results of a State-Wide STEPS Survey, PLOS ONE.11(7): e0157705. https://doi.org/10.1371/journal.pone.0157705.

Region	Country	Sources of alcohol patterns of consumption data by WHO Member State
		The International GENACIS-Project (2003). GENACIS - gender, alcohol and culture: an international study. (http://www.genacis.org/. Accessed: 01/10/2018).
SEAR	Indonesia	Ministry of Home Affairs, National Development Planning Agency, Statistics Indonesia & United Nations Children's Fund 2013. Indonesia - West Papua Multiple Indicator Cluster Survey 2011. New York, USA: United Nations Children's Fund.
		Statistics Indonesia (Badan Pusat Statistik—BPS), National Population and Family Planning Board (BKKBN), Kementerian Kesehatan (Kemenkes—MOH), ICF Internationa (2013). Indonesia Demographic and Health Survey 2012. Jakarta, Indonesia: BPS, BKKBN, Kemenkes, and ICF International
SEAR	Maldives	World Health Organization (2011b). STEPwise approach to surveillance (STEPS) Survey. (http://www.who.int/chp/steps/en/. Accessed: 01/12/2018).
SEAR	Myanmar	Bjertness MB, Htet AS, Meyer HE, Htike MM, Zaw KK, Oo WM, et al. (2016). Prevalence and deteminants of hypertention in Myanmar - a nationwide cross-sectional study, BMC Public Health.16:590.
		World Health Organization (2014b). STEPwise approach to surveillance (STEPS) Survey. (http://www.who.int/chp/steps/en/. Accessed: 01/12/2018).
SEAR	Nepal	Central Bureau of Statistics (2012). Nepal Multiple Indicator Cluster Survey 2010, Mid- and Far Western Regions, Final Report. Kathmandu, Nepal: Central Bureau of Statistics and UNICEF Nepal.
		Central Bureau of Statistics (2015). Nepal Multiple Indicator Cluster Survey 2014, Final Report. Kathmandu, Nepal: Central Bureau of Statistics and UNICEF Nepal.
		Central Bureau of Statistics (2020). Nepal Multiple Indicator Cluster Survey 2019, Key Indicators. (https://www.unicef.org/nepal/media/9076/file/NMICS_2019_-_Key_findings.pdf).
		World Health Organization (2013c). STEPwise approach to surveillance (STEPS) Survey. (http://www.who.int/chp/steps/en/. Accessed: 01/15/2018).
		World Health Organization (2019a). STEPwise approach to surveillance (STEPS) Survey. (https://extranet.who.int/ncdsmicrodata/index.php/catalog/STEPS. Accessed: 07/02/2020).
SEAR	Sri Lanka	Alcohol and Drug Information Center (2017). Trend survey July 2015. (http://adicsrilanka.org/wp-content/uploads/2017/06/Spot-Survey-July-2015.pdf. Accessed: 04/04/2018).
		Greenfield TK, Bloomfield K, Wilsnack SC (2014b). GENAHTO Project (Gender and Alcohol's Harm to Others). (http://genahto.org/. Accessed: 07/02/2020).
		Research and Evaluation Programme - Alcohol and Drug Information Centre (2014). Trend Survey on Alcohol 2014. Sri Lanka: Research and Evaluation Programme - Alcohol and Drug Information Centre.
		Research and Evaluation Programme - Alcohol and Drug Information Centre, Sri Lanka (2019). Alcohol Spot Survey July 2016. (https://adicsrilanka.org/2019/09/07/july-2016-alcohol-report/. Accessed: 11/13/2020).
		Research and Evaluation Programme - Alcohol and Drug Information Centre, Sri Lanka (2019). Spot Survey 2017 Report on Alcohol Consumption Trends. (https://adicsrilanka.org/2019/09/07/july-2017-alcohol-report/. Accessed: 11/13/2020).
		Research and Evaluation Programme - Alcohol and Drug Information Centre, Sri Lanka (2020). Trends Survey on Alcohol 2019. (https://adicsrilanka.org/2020/06/20/alcohol-trend-survey-2019/. Accessed 11/13/2020).
		Somatunga LC, Ratnayake LVR, Wijesinghe WMDNK, Yapa YMMM, Cooray MPNS (2014). National alcohol use prevalence survey in Sri Lanka, Journal of the Postgraduate Institute of Medicine.1(1):1–12.
		The International GENACIS-Project (2002). GENACIS - gender, alcohol and culture: an international study. (http://www.genacis.org/. Accessed: 01/11/2018).
		World Health Organization (2006c). STEPwise approach to surveillance (STEPS) Survey. (http://www.who.int/chp/steps/en/. Accessed: 01/12/2018).
		World Health Organization (2014b). STEPwise approach to surveillance (STEPS) Survey. (http://www.who.int/chp/steps/en/. Accessed: 01/12/2018).
		World Health Organization, Centers for Disease Control and Prevention (2016). Global School-based Student Health Survey – Sri Lanka 2016 Fact Sheet. (https://www.who.int/ncds/surveillance/gshs/SRH2016_fact_sheet.pdf. Accessed: 07/31/2020).
SEAR	Thailand	Bureau of Non-communicable Diseases, Department of Disease Control, Ministry of Public Health (2015). Core Table: Behavioural Risk Factor Surveillance System 2015 (BRFSS 2015). (http://www.thaincd.com/2016/media-detail.php?id=10520&tid=&gid=1-015-005. Accessed: 10/22/2019).
		Greenfield TK, Bloomfield K, Wilsnack SC (2013d). GENAHTO Project (Gender and Alcohol's Harm to Others). (http://genahto.org/. Accessed: 07/02/2020).
		Intarut N, Pukdeesamai P (2017). Socioeconomic Inequality in Concurrent Tobacco and Alcohol Consumption. Asian Pacific Journal for Cancer Prevention.18(7): 1913-1917.
		National Statistical Office (2011). Cigarette Smoking and Drinking Behaviour Survey: Alcohol Drinking Habit. (http://web.nso.go.th/en/survey/health/cigareetts_11.htm. Accessed: 06/11/2020).
		National Statistical Office (2014). Cigarette Smoking and Drinking Behaviour Survey: Statistical Tables. (http://web.nso.go.th/en/survey/health/cigareetts_14.htm. Accessed: 06/28/2017).
		National Statistical Office (2018). The smoking and drinking behavior survey 2017. (http://www.nso.go.th/sites/2014en/Survey/social/health/SmokingDrinking/2017/Full%20Report.pdf. Accessed: 11/05/2020).
		Saengow U (2019). Drinking abstinence during a 3-month abstinence campaign in Thailand: weighted analysis of a national representative survey, BMC public health.19(1):1688. https://dx.doi.org/10.1186/s12889-019-8051-z.
		Sirirassamee T, Sirirassamee B (2015). Health risk behavior among Thai youth: national survey 2013, Asia-Pacific journal of public health.27(1):76-84. https://dx.doi.org/10.1177/1010539514548759.
		Tanaree A, Assanangkornchai S, Kittirattanapaiboon P (2017). Pattern and risk of developing alcohol use disorders, illegal substance use and psychiatric disorders after early onset of alcohol use: Results of the Thai National Mental Health Survey 2013, Drug and Alcohol dependence.170(ebs, 7513587):102-11. https://dx.doi.org/10.1016/j.drugalcdep.2016.11.001.
		World Health Organization, Centers for Disease Control and Prevention (2015). Global School-based Student Health Survey – Thailand 2015 Fact Sheet. (https://www.who.int/ncds/surveillance/gshs/2015-Thailand-GSHS-Fact-Sheet.pdf. Accessed: 07/31/2020).
SEAR	Timor-Leste	General Directorate of Statistics (GDS), Ministry of Health, ICF (2018). Timor-Leste Demographic and Health Survey 2016. Dili, Timor-Leste and Rockville, Maryland, USA: GDS and ICF.

Region	Country	Sources of alcohol patterns of consumption data by WHO Member State
		World Health Organization (2014b). STEPwise approach to surveillance (STEPS) Survey. (http://www.who.int/chp/steps/en/. Accessed: 01/12/2018).
		World Health Organization, Centers for Disease Control and Prevention (2015). Global School-based Student Health Survey – Timor-Leste 2015 Fact Sheet. (https://www.who.int/ncds/surveillance/gshs/GSHS_2015_Timor-Leste_Fact_Sheet.pdf. Accessed: 07/31/2020).
WPR	Australia	Australian Bureau of Statistics (2015). National Health Survey: First Results, 2014-2015. (http://www.abs.gov.au/ausstats/abs@.nsf/Lookup/by%20Subject/4364.0.55.001~2014-15~Main%20Features~Alcohol%20consumption~25. Accessed: 07/04/2017).
		Australian Bureau of Statistics (2018). National Health Survey: First Results, 2017-2018. (https://www.abs.gov.au/statistics/health/health-conditions-and-risks/national-health-survey-first-results/latest-release. Accessed: 05/09/2020).
		Australian Institute of Health and Welfare (2011). 2010 National Drug Strategy Household Survey Report. Drug statistics series no 25. Canberra, Australia: Australian Institute of Health and Welfare.
		Australian Institute of Health and Welfare (2016). Trends in alcohol availability, use and treatment 2003–04 to 2014–15. Canberra, Australia: Australian Institute of Health and Welfare.
		Greenfield TK, Bloomfield K, Wilsnack SC (2008). GENAHTO Project (Gender and Alcohol's Harm to Others). (http://genahto.org/. Accessed: 07/02/2020).
		The International GENACIS-Project (1994). GENACIS - gender, alcohol and culture: an international study. (http://www.genacis.org/. Accessed: 01/10/2018).
		The International GENACIS-Project (2007). GENACIS - gender, alcohol and culture: an international study. (http://www.genacis.org/. Accessed: 01/10/2018).
WPR	Brunei Darussalam	World Health Organization (2016). STEPwise approach to surveillance (STEPS) Survey. (https://extranet.who.int/ncdsmicrodata/index.php/catalog/STEPS. Accessed: 07/02/2020).
		World Health Organization, Centers for Disease Prevention and Control (2014). Global School-based Student Health Survey – Brunei Darussalam 2014 Fact Sheet. (http://www.who.int/chp/gshs/brunei/en/. Accessed: 06/27/2017).
WPR	Cambodia	Schunert T, Khann S, Kao S, Pot C, Saupe LB, Sek S, et al. (2012). Cambodian Mental Health Survey 2012. (http://tpocambodia.org/wp-content/uploads/2015/09/Cambodian-Mental-Health-Survey-2012-RUPP.pdf. Accessed: 06/16/17).
		World Health Organization (2010). STEPwise approach to surveillance (STEPS) Survey. (http://www.who.int/chp/steps/en/. Accessed: 01/12/2018).
		World Health Organization, Centers for Disease Control and Prevention (2014). Global School-based Student Health Survey – Cambodia 2013 Fact Sheet. (https://www.who.int/ncds/surveillance/gshs/2013_Cambodia_GSHS_Fact_Sheet.pdf. Accessed: 06/22/2020).
WPR	China	Ge S, Wei Z, Liu T, Wang J, Li H, Feng J, et al. (2018). Alcohol Use and Cognitive Functioning Among Middle-Aged and Older Adults in China: Findings of the China Health and Retirement Longitudinal Study Baseline Survey, Alcoholism, clinical and experimental research.42(10):2054-60. https://dx.doi.org/10.1111/acer.13861.
		Lee YH, Wang Z, Chiang TC, Liu CT (2017). Beverage Intake, Smoking Behavior, and Alcohol Consumption in Contemporary China-A Cross-Sectional Analysis from the 2011 China Health and Nutrition Survey. International Journal of Environmental Research and Public Health.14(5):493. https://doi.org/10.3390/ijerph14050493.
		Millwood IY, Li L, Smith M, Guo Y, Yang L, Bian Z, et al. (2013). Alcohol consumption in 0.5 million people from 10 diverse regions of China: prevalence, patterns and socio-demographic and health-related correlates, International Journal of Epidemiology.42(3):816-27.
		World Health Organization (2012c). Study on global AGEing and adult health (SAGE) Wave 1 - China National Report. Geneva, Switzerland: World Health Organization.
		Zhao Z, Wang L, Zhang M, Zhang X, Huang Z, Li C, et al. (2020). Geographic Distribution of Alcohol Use Among Chinese Adults—China, 2015. China CDC Weekly. 2(7):98-103.
WPR	Cook Islands	Te Marae Ora Ministry of Health (2014). Cook Islands Global Health Survey 2010 Report. Cook Islands: Te Marae Ora Ministry of Health, World Health Organization, Centers for Disease Control and Prevention.
		World Health Organization (2003a). STEPwise approach to surveillance (STEPS) Survey. (http://www.who.int/chp/steps/en/. Accessed: 01/11/2018).
		World Health Organization (2013c). STEPwise approach to surveillance (STEPS) Survey. (http://www.who.int/chp/steps/en/. Accessed: 01/15/2018).
WPR	Fiji	World Health Organization (2002). STEPwise approach to surveillance (STEPS) Survey. (http://www.who.int/ncds/surveillance/steps/en/. Accessed: 01/11/2018).
		World Health Organization (2011b). STEPwise approach to surveillance (STEPS) Survey. (http://www.who.int/chp/steps/en/. Accessed: 01/12/2018).
		World Health Organization, Centers for Disease Control and Prevention (2010). Global School-based Student Health Survey – Fiji 2010 Fact Sheet. https://www.who.int/ncds/surveillance/gshs/Fiji_2010_GSHS_FS.pdf. Accessed: 06/14/2018).
		World Health Organization, Centers for Disease Control and Prevention (2020). Global School-based Student Health Survey – Fiji 2016 Fact Sheet. (https://www.who.int/ncds/surveillance/gshs/fiji/en/. Accessed: 06/22/2020)
WPR	Japan	ISSP Research Group (2009). International Social Survey Programme: Health and Health Care - ISSP 2011. Cologne: GESIS Data Archive.
		Kinjo A, Kuwabara Y, Minobe R, Maezato H, Kimura M, Higuchi S, et al. (2018). Different socioeconomic backgrounds between hazardous drinking and heavy episodic drinking: Prevalence by sociodemographic factors in a Japanese general sample, Drug and Alcohol Dependence.193:55-62. https://dx.doi.org/10.1016/j.drugalcdep.2018.08.015.
		Midorikawa H, Tachikawa H, Aiba M, Arai T, Watanabe T, Tamiya N (2019). Factors associated with high-risk drinking in older adults: Evidence from a national survey in Japan, Geriatrics & Gerontology International.19(12):1260-7. https://dx.doi.org/10.1111/ggi.13808.
		The International GENACIS-Project. (2001). GENACIS - gender, alcohol and culture: an international study. (http://www.genacis.org/. Accessed: 01/11/2018).

Region	Country	Sources of alcohol patterns of consumption data by WHO Member State
WPR	Kiribati	Centers for Disease Control and Prevention, World Health Organization (2014a). Kiribati Global School-based Student Health Survey 2011. Geneva, Switzerland: World Health Organization.
WPR	Kiribati	Kiribati National Statistics Office (2019). Kiribati Social Development Indicator Survey 2018-19: Survey Findings Report. South Tarawa, Kiribati: National Statistics Office.
WPR	Kiribati	Statistics Office, Ministry of Finance (2002). Report on the 2000 Census of Population. Bairiki, Tarawa: Statistics Office Ministry of Finance.
WPR	Kiribati	World Health Organization (2004). STEPwise approach to surveillance (STEPS) Survey. (http://www.who.int/ncds/surveillance/steps/en/. Accessed: 01/12/2018).
WPR	Kiribati	World Health Organization (2016). STEPwise approach to surveillance (STEPS) Survey. (https://extranet.who.int/ncdsmicrodata/index.php/catalog/STEPS. Accessed: 07/02/2020).
WPR	Lao People's Democratic Republic	Greenfield TK, Bloomfield K, Wilsnack SC (2013c). GENAHTO Project (Gender and Alcohol's Harm to Others). (http://genahto.org/. Accessed: 07/02/2020).
WPR	Lao People's Democratic Republic	Lao Statistics Bureau (2018). Lao Social Indicator Survey II 2017, Survey Findings Report. Vientiane, Lao PDR: Lao Statistics Bureau, UNICEF.
WPR	Lao People's Democratic Republic	World Health Organization (2008). STEPwise approach to surveillance (STEPS) Survey. (http://www.who.int/chp/steps/en/. Accessed: 01/12/2018).
WPR	Lao People's Democratic Republic	World Health Organization (2013c). STEPwise approach to surveillance (STEPS) Survey. (http://www.who.int/chp/steps/en/. Accessed: 01/15/2018).
WPR	Malaysia	Centers for Disease Control and Prevention, Ministry of Health, World Health Organization (2012). Malaysia Global School-based Student Health Survey 2012. Geneva, Switzerland: World Health Organization.
WPR	Malaysia	Institute for Public Health (2011). National Health and Morbidity Survey 2011 (NHMS 2011). Vol. II: Non-Communicable Diseases. Kuala Lumpur, Malaysia: Institute for Public Health
WPR	Malaysia	Institute for Public Health (2015). National Health and Morbidity Survey 2015 (NHMS 2015). Vol. II: Non-Communicable Diseases, Risk Factors & Other Health Problems. Malaysia, Kuala Lumpur: Institute for Public Health.
WPR	Malaysia	Institute for Public Health, National Institutes of Health, Ministry of Health Malaysia (2020). National Health and Morbidity Survey (NHMS) 2019. Vol. I: NCDs – Non-Communicable Diseases: Risk Factors and other Health Problems. Kuala Limpur, Malaysia: Institute for Public Health, National Institutes of Health, Ministry of Health.
WPR	Marshall Islands	-
WPR	Micronesia (Federated States of)	World Health Organization (2002). STEPwise approach to surveillance (STEPS) Survey. (http://www.who.int/ncds/surveillance/steps/en/. Accessed: 01/11/2018).
WPR	Micronesia (Federated States of)	World Health Organization (2006c). STEPwise approach to surveillance (STEPS) Survey. (http://www.who.int/chp/steps/en/. Accessed: 01/12/2018).
WPR	Micronesia (Federated States of)	World Health Organization (2008). STEPwise approach to surveillance (STEPS) Survey. (http://www.who.int/chp/steps/en/. Accessed: 01/12/2018).
WPR	Micronesia (Federated States of)	World Health Organization (2009b). STEPwise approach to surveillance (STEPS) Survey. (http://www.who.int/chp/steps/en/. Accessed: 01/12/2018).
WPR	Mongolia	Centers for Disease Control and Prevention, World Health Organization (2013). Mongolia Global School-based Student Health Survey 2013. Geneva, Switzerland: World Health Organization.
WPR	Mongolia	Demaio AR, Dugee O, de Courten M, Bygbjerg IC, Enkhtuya P, Meyrowitsch DW (2013). Exploring knowledge, attitudes, and practices related to alcohol in Mongolia: a national population-based survey, BMC public health.13:178. https://dx.doi.org/10.1186/1471-2458-13-178.
WPR	Mongolia	NSO (2019). Social Indicator Sample Survey-2018, Survey Findings Report. Ulaanbaatar, Mongolia: National Statistical Office of Mongolia.
WPR	Mongolia	National Statistical Office of Mongolia, United Nations Children's Fund (UNICEF) (2013). Mongolia multiple indicator cluster survey 2010. New York, USA: United Nations Children's Fund.
WPR	Mongolia	World Health Organization (2009b). STEPwise approach to surveillance (STEPS) Survey. (http://www.who.int/chp/steps/en/. Accessed: 01/12/2018).
WPR	Mongolia	World Health Organization (2013c). STEPwise approach to surveillance (STEPS) Survey. (http://www.who.int/chp/steps/en/. Accessed: 01/15/2018).
WPR	Mongolia	World Health Organization (2019c). STEPwise approach to surveillance (STEPS) Survey. (http://www.who.int/chp/steps/en/. Accessed: 11/12/2020).
WPR	Nauru	World Health Organization (2004). STEPwise approach to surveillance (STEPS) Survey. (http://www.who.int/ncds/surveillance/steps/en/. Accessed: 01/12/2018).
WPR	Nauru	World Health Organization, Centers for Disease Control and Prevention (2011). Global School-based Student Health Survey – Nuaru 2011 Fact Sheet. (https://www.who.int/ncds/surveillance/gshs/Nauru_GSHS_FS_2011.pdf. Accessed: 02/27/2017).
WPR	New Zealand	Greenfield TK, Bloomfield K, Wilsnack SC (2009). GENAHTO Project (Gender and Alcohol's Harm to Others). (http://genahto.org/. Accessed: 07/02/2020).
WPR	New Zealand	Health Promotion Agency (2017) Key Results: Young People aged 15-24 years. Attitudes and Behaviour towards Alcohol Survey 2013/14 to 2015/16. Wellington, New Zealand: Health Promotion Agency.
WPR	New Zealand	Ministry of Health (2010). The 2007/08 New Zealand Alcohol and Drug use Survey: Online data tables. (http://www.health.govt.nz/publication/2007-08-new-zealand-alcohol-and-drug-use-survey-online-data-tables. Accessed: 06/26/2017).
WPR	New Zealand	Ministry of Health (2019). New Zealand Health Survey Annual Data Explorer [Topic: Alcohol Use, Indicators: Past-year drinkers, Heavy episodic drinking, Years: 2011/12-2018/19]. (https://minhealthnz.shinyapps.io/nz-health-survey-2018-19-annual-data-explorer/_w_63a7807d/#!/explore-indicators. Accessed: 10/21/2020).
WPR	Niue	World Health Organization (2011b). STEPwise approach to surveillance (STEPS) Survey. (http://www.who.int/chp/steps/en/. Accessed: 01/12/2018).
WPR	Palau	Ministry of Health (2014). National Health Profile 2013. (http://www.palauhealth.org/files/2013%20National%20Health%20Profile_%20Final%20121214.pdf. Accessed: 06/27/2020).

Region	Country	Sources of alcohol patterns of consumption data by WHO Member State
		Ministry of Health (2017). Palau Hybrid Survey Final Report. (http://www.palauhealth.org/files/Palau%20Hybrid%20Report_2017_FINAL.pdf. Accessed: 05/14/2020).
		World Health Organization (2011b). STEPwise approach to surveillance (STEPS) Survey. (http://www.who.int/chp/steps/en/. Accessed: 01/12/2018).
WPR	Papua New Guinea	Rarau P, Pulford J, Gouda H, Phuanukoonnon S, Bullen C, Scragg R, et al. (2019). Socio-economic status and behavioural and cardiovascular risk factors in Papua New Guinea: A cross-sectional survey, PLoS ONE. 14(1): e0211068. https://doi.org/10.1371/journal.pone.0211068.
		World Health Organization (2007). STEPwise approach to surveillance (STEPS) Survey. (http://www.who.int/chp/steps/en/. Accessed: 01/11/2018).
WPR	Philippines	Food and Nutrition Research Institute (2013). Burden of selected risk factors to non communicable diseases (NCDs) among Filipino adults. Taguig, Philippines: Certification International.
		Department of Science and Technology - Food and Nutrition Research Institute (DOST-FNRI) (2016). Philippine Nutrition Facts and Figures 2015: Clinical and Health Survey. (http://enutrition.fnri.dost.gov.ph/site/uploads/2015_CLINICAL_AND_HEALTH_SURVEY.pdf. Accessed: 10/03/2019).
		World Health Organization, Centers for Disease Control and Prevention (2011). Philippines Global School-based Student Health Survey 2011. Geneva, Switzerland: World Health Organization.
		World Health Organization, Centers for Disease Control and Prevention (2015). Global School-based Student Health Survey – Philippines 2015 Fact Sheet. (https://www.who.int/ncds/surveillance/gshs/PIH2015_fact_sheet.pdf. Accessed: 07/31/2020).
WPR	Republic of Korea	Korea Disease Control and Prevention Agency (2008). National Health and Nutrition Survey 2007. (https://knhanes.cdc.go.kr/. Accessed: 01/03/2021).
		Korea Disease Control and Prevention Agency (2009). National Health and Nutrition Survey 2008. (https://knhanes.cdc.go.kr/. Accessed: 01/03/2021).
		Korea Disease Control and Prevention Agency (2010). National Health and Nutrition Survey 2009. (https://knhanes.cdc.go.kr/. Accessed: 01/02/2021).
		Korea Disease Control and Prevention Agency (2011). National Health and Nutrition Survey 2010. (https://knhanes.cdc.go.kr/. Accessed: 01/02/2021).
		Korea Disease Control and Prevention Agency (2012). National Health and Nutrition Survey 2011. (https://knhanes.cdc.go.kr/. Accessed: 01/02/2021).
		Korea Disease Control and Prevention Agency (2013). National Health and Nutrition Survey 2012. (https://knhanes.cdc.go.kr/. Accessed: 12/31/2020).
		Korea Disease Control and Prevention Agency (2014). National Health and Nutrition Survey 2013. (https://knhanes.cdc.go.kr/. Accessed: 12/31/2020).
		Korea Disease Control and Prevention Agency (2015). National Health and Nutrition Survey 2014. (https://knhanes.cdc.go.kr/. Accessed: 12/20/2020).
		Korea Disease Control and Prevention Agency (2016). National Health and Nutrition Survey 2015. (https://knhanes.cdc.go.kr/. Accessed: 12/20/2020).
		Korea Disease Control and Prevention Agency (2017). National Health and Nutrition Survey 2016. (https://knhanes.cdc.go.kr/. Accessed: 12/20/2020).
		Korea Disease Control and Prevention Agency (2019). National Health and Nutrition Survey 2017. (https://knhanes.cdc.go.kr/. Accessed: 11/13/2020).
		Korea Disease Control and Prevention Agency (2020). National Health and Nutrition Survey 2018. (https://knhanes.cdc.go.kr/. Accessed: 11/13/2020).
		Noh J-W, Park H, Kim M, Kwon YD (2018). Gender Differences and Socioeconomic Factors Related to Osteoporosis: A Cross-Sectional Analysis of Nationally Representative Data, Journal of women's health.27(2):196-202. https://dx.doi.org/10.1089/jwh.2016.6244.
WPR	Samoa	Barnes SS, Small CR, Lauilefue TA, Bennett J, Yamada S (2010). Alcohol consumption and gender in rural Samoa, Substance Abuse and Rehabilitation.1:1-4.
		Centers for Disease Control and Prevention, World Health Organization (2014b). Samoa Global School-based Student Health Survey 2011-2012. Geneva, Switzerland: Centers for Disease Control and Prevention, World Health Organization.
		World Health Organization (2002). STEPwise approach to surveillance (STEPS) Survey. (http://www.who.int/ncds/surveillance/steps/en/. Accessed: 01/11/2018).
		World Health Organization (2013c). STEPwise approach to surveillance (STEPS) Survey. (http://www.who.int/chp/steps/en/. Accessed: 01/15/2018).
WPR	Singapore	Epidemiology and Disease Control Division and Policy, Research & Surveillance Group, Ministry of Health and Health Promotion Board (2020). National Population Heath Survey 2019 (Household Interview). Republic of Singapore: Ministry of Health.
		Epidemiology and Disease Control Division, Ministry of Health, Singapor (2005). National Health Survey 2004. (https://www.moh.gov.sg/docs/librariesprovider5/resources-statistics/reports/nhs_2004(part5).pdf. Accessed: 10/31/2020).
		Epidemiology and Disease Control Division, Ministry of Health, Singapore (2011). National Health Survey 2010 Singapore. (https://www.moh.gov.sg/content/dam/moh_web/Publications/Reports/2011/NHS2010%20-%20low%20res.pdf. Accessed: 06/27/2017).
		Lee YY, Wang P, Abdin E, Chang S, Shafie S, Sambasivam R, et al. (2020). Prevalence of binge drinking and its association with mental health conditions and quality of life in Singapore, Addictive Behaviors.100:106114.
		Ong LT (2018). RE: NHSS 2013 data provided by Ministry of Health, Singapore. (Accessed: 02/01/2018).
WPR	Solomon Islands	Quinn B (2016). Alcohol, other substance use and related harms among young people in the Soloman Islands. West Honiara, Solomon Islands: Save the Children Australia.
		World Health Organization (2006c). STEPwise approach to surveillance (STEPS) Survey. (http://www.who.int/chp/steps/en/. Accessed: 01/12/2018).

Region	Country	Sources of alcohol patterns of consumption data by WHO Member State
		World Health Organization, Centers for Disease Control and Prevention (2011). Global School-based Student Health Survey – Solomon Islands 2011 Fact Sheet. (https://www.who.int/ncds/surveillance/gshs/2011_GSHS_FS_Solomon_Islands.pdf. Accessed: 06/28/2017).
WPR	Tonga	World Health Organization (2004). STEPwise approach to surveillance (STEPS) Survey. (http://www.who.int/ncds/surveillance/steps/en/. Accessed: 01/12/2018).
		World Health Organization (2011b). STEPwise approach to surveillance (STEPS) Survey. (http://www.who.int/chp/steps/en/. Accessed: 01/12/2018).
		World Health Organization, Centers for Disease Control and Prevention (2017). Global School-based Student Health Survey – Tonga 2017 Fact Sheet. (https://www.who.int/ncds/surveillance/gshs/TOH2017_fact_sheet.pdf. Accessed: 07/31/2020).
		World Health Organization, Centers for Disease Control and Prevention, Massey University Auckland (2012). Tonga 2010 Global School-based Student Health Survey. Tonga: World Health Organization.
WPR	Tuvalu	World Health Organization, Centers for Disease Control and Prevention (2013). Global School-based Student Health Survey – Tuvalu 2013 Fact Sheet. Funafuti, Tuvalu: Ministry of Health.
WPR	Vanuatu	World Health Organization (2005b). STEPwise approach to surveillance (STEPS) Survey. (http://www.who.int/ncds/surveillance/steps/en/. Accessed: 01/12/2018).
		World Health Organization (2011b). STEPwise approach to surveillance (STEPS) Survey. (http://www.who.int/chp/steps/en/. Accessed: 01/12/2018).
		World Health Organization, Centers for Disease Control and Prevention (2011). Global School-based Student Health Survey – Vanuatu 2011 Fact Sheet. (https://www.cdc.gov/gshs/countries/westpacific/vanuatu.htm. Accessed: 06/16/2017).
		World Health Organization, Centers for Disease Control and Prevention (2016). Global School-based Student Health Survey – Vanuatu 2016 Fact Sheet. (https://www.who.int/ncds/surveillance/gshs/Vanuatu_2016_GSHS_FS.pdf?ua=1. Accessed: 07/31/2020).
WPR	Viet Nam	Giang KB, Van Minh H, Allebeck P (2013). Alcohol consumption and household expenditure on alcohol in a rural district in Vietnam, Global Health Action.6:18937.
		Greenfield TK, Bloomfield K, Wilsnack SC (2013e). GENAHTO Project (Gender and Alcohol's Harm to Others). (http://genahto.org/. Accessed: 07/02/2020).
		Van Bui T, Blizzard L, Ngoc Luong K, Van Truoong NL, Tran BQ, Otahal P, et al. (2015). Alcohol consumption in Vietnam, and the use of 'Standard Drinks' to measure alcohol intake, Alcohol and Alcoholism.51(2):1-10.
		World Health Organization (2009b). STEPwise approach to surveillance (STEPS) Survey. (http://www.who.int/chp/steps/en/. Accessed: 01/12/2018).
		World Health Organization (2015). STEPwise approach to surveillance (STEPS) Survey. (http://www.who.int/chp/steps/en/. Accessed: 01/12/2018).
		World Health Organization (2017a). Global School-based Student Health Survey (GSHS) Viet Nam. (http://www.who.int/chp/gshs/vietnam/en/. Accessed: 06/16/2017).

Modelling alcohol consumption for the estimation of alcohol-attributable mortality and morbidity

Alcohol consumption was modelled as 1) drinking status (current drinkers, former drinkers, lifetime abstainers), 2) average daily volume of alcohol consumption among current drinkers, modelled on the basis of *per capita* consumption, the prevalence of current drinkers, and the amount of alcohol consumed among current drinkers by age and sex), and 3) HED, defined as drinking 60 grams or more of pure alcohol on one occasion, modelled on the basis of the prevalence of current drinkers and the prevalence of HED among current drinkers. Data on alcohol consumption (drinking status and amount consumed by current drinkers) and HED were available by age group (15–19 years, 20–24 years, 25–34 years, 35–49 years, 50–64 years, and 65 years of age and older) and sex.

Drinking status prevalences were modeled using covariates for sex, age, IHME Global Burden of Disease (GBD) region, the time frame used to define current and former drinking (with the past year as the reference category), gross domestic product (GDP) adjusted for purchasing power parity, the prevalence of abstainers, the percentage of the population identifying as Muslim, the presence of a national ban on alcohol sales, and adult per capita alcohol consumption. Interaction terms were included to account for the hypothesized interaction between age and sex.

Average daily volume of alcohol consumption among current drinkers was modeled using covariates for sex, age, IHME GBD region, GDP (adjusted for purchasing power parity), the prevalence of abstainers, and adult per capita alcohol consumption. Interaction terms were also used to model the hypothesized interaction between age and sex.

Heavy episodic drinking among current drinkers was modeled using covariates for sex, age, IHME GBD region, the time frame used to measure heavy episodic drinking (with the past month or 28 days as the reference category), the threshold used to define heavy episodic drinking (with 60 g of alcohol per occasion as the reference), GDP (adjusted for purchasing power parity), the prevalence of abstainers, and adult per capita consumption. Interaction terms were again included to model the hypothesized interaction between age and sex.

The amount of alcohol consumed by current drinkers was adjusted using a correction factor of 0.8. This correction factor was used to account for 1) alcohol that was not consumed, and 2) the underreporting of alcohol consumption in medical observation studies from which the RR estimates used in this study were obtained.[1] A study by Stockwell and colleagues found that cohort studies of the relationship between alcohol consumption and all-cause mortality had a coverage rate (when compared to *per capita* consumption) of 61·71% (ranging from 29·19% for Russia to 96·53% for Japan).[2] The adjustment of survey data can be justified by the observation that the underreporting of alcohol consumption in medical epidemiology studies is much less than in population surveys; population-level surveys underestimate alcohol consumption because, on average, such surveys ask many fewer questions that are used to measure alcohol consumption compared to the number of such questions asked in medical epidemiology studies.[3-5] Furthermore, the undercoverage of population surveys is also affected by recruitment biases;[6] however, the adjustment of survey data assumes that the undercoverage of alcohol consumption is constant by age and sex.

Average daily alcohol consumption among current drinkers was modelled using a Gamma distribution in accordance with the methodology outlined by Rehm and colleagues and Kehoe and colleagues.[7,8] This methodology was developed using data from over 60 individual surveys conducted in both developing and developed countries. Firstly, this method assumes that the average daily alcohol consumed among current drinkers can be accurately modelled using a Gamma distribution, which was the case in the surveys examined by both Rehm and Kehoe and their respective colleagues. Secondly, this method assumes that the standard deviation of the Gamma distribution of alcohol consumption can be predicted on the basis of the mean consumption of alcohol. Both Rehm and Kehoe and their respective colleagues observed a strong correlation between the mean and the standard deviation of the Gamma distribution (an r of 0·971). Therefore, based on the mean alcohol consumed (μ) by age and sex, the standard deviation (σ) was estimated according to Formula S1 (the coefficient of sex is 1 for women and 0 for men in Formula S1).

[*Formula S1*]

$$\hat{\sigma}_{shifted=}(1.171 + 0.087 * sex) * \hat{\mu}_{shifted}$$

Modelling alcohol-attributable mortality

Mortality and morbidity data, deaths, Years of Life Lost (YLL), Years lived with Disability (YLD) and Disability Adjusted Life Years (DALYs) lost were obtained from the WHO's Global Health Observatory by cause, age, sex, and year (2000 to 2019). To match age-standardization data, deaths, YLL, YLD and DALYs lost were aggregated into the following age groups: 0 to 4, 5 to 9, 10 to 14, 15 to 19, 20 to 24, 25 to 29, 30 to 34, 35 to 39, 40 to 44, 45 to 49, 50 to 54, 55 to 59, 60 to 64, 65 to 69, 70 to 74, 75 to 79, 80 to 84, and 85 years of age and older. The causes of death which were extracted are presented in Table S6.

Using Formula S2, estimates of motor vehicle deaths due to alcohol consumption were stratified into those involving the driver and those involving others, based on the fractions of motor vehicle deaths that involved drivers and that involved people other than the driver as obtained from the WHO's road traffic deaths database. This method estimates the number of deaths (D) among drivers (d) using the fraction (F) of injury events in a country that occurred among drivers by sex (indexed by i) and age (indexed by p including people 15 years of age and older). The method of estimating the number of motor vehicle accident (MVA) injuries involving drivers assumed that all MVA injuries to drivers occurred among people aged 15 years or older (i.e., all MVA injuries occurring among people 0-14 years of age involved people other than the driver). For countries where data were not available, the fractions of injuries among drivers and among people other than the driver were imputed as the regional averages. To estimate the number of YLL, YLD, and DALYs lost among drivers, the fraction of deaths among drivers compared to all MVA deaths (by age and sex) were used. All other MVA deaths, YLLs, YLDs, and DALYs lost affected people other than the driver.

[Formula S2]

$$Dd_{p,i} = \frac{F_i \cdot F_p \cdot \sum_{p=1}^{pn} \sum_{i=1}^{in} D_{p,i}}{D_{p,i}}$$

Estimation of alcoholic cardiomyopathy mortality and morbidity

Deaths, YLL, YLD, and DALYs lost due to alcoholic cardiomyopathy (ICD-10 code: I42.6) are not estimated specifically by the WHO but are estimated by the IHME's annual statistics. With respect to the WHO data, alcoholic cardiomyopathy is, contained in the larger category of cardiomyopathy, myocarditis and endocarditis mortality and morbidity (ICD-10 codes: I30–I33, I38, I40, I42). Accordingly, we applied the percentage of cardiomyopathy, myocarditis, and endocarditis deaths that were from alcoholic cardiomyopathy as reported by the IHME to the WHO's Global Health estimates of mortality and morbidity from cardiomyopathy, myocarditis, and endocarditis to estimate alcoholic cardiomyopathy deaths.

Mortality and morbidity attributable to alcohol consumption

The number of deaths, YLL, YLD, and DALYs lost attributable to alcohol consumption were estimated using a Levin-based population-attributable fraction (PAF) methodology.[9] The association of alcohol and mortality is complex: alcohol has a protective effect (when compared to lifetime abstainers) on ischaemic heart disease, ischaemic stroke, and diabetes for drinkers who consume low volumes of alcohol and do not engage in HED.[10-14] However, the overall protective effect of alcohol depends on the risks of diseases and injuries associated or not associated with alcohol (i.e., competing risks) [15]. Therefore, in accordance with the methods of previous comparative risk assessment studies, the mortality and morbidity attributable to alcohol consumption were estimated using a counterfactual scenario (i.e., theoretical minimum risk) of lifetime abstention (i.e., no historical consumption of alcohol) [16]. For diseases and injuries where alcohol is a necessary cause (i.e. AUDs (ICD-10 codes: F10, G72.1, Q86.0 and X45)), the attributable fraction was assumed to be 1. For diseases and injuries where alcohol is a potential component cause (i.e., where alcohol raises the risk of disease or injury occurrence, but the

disease or injury may still occur in the absence of alcohol), a PAF is used to estimate the fraction of deaths, YLL, YLD, and DALYs lost attributable to alcohol.

Estimation of alcohol-attributable fractions

With respect to noncommunicable diseases (other than cancer), no latency period was used in the estimation of the attributable fractions. For cancer mortality and morbidity attributable to alcohol consumption, a latency period of 10 years between the consumption of alcohol and the diagnosis and/or death from cancer was chosen, based on an observed approximate latency period of 11–12 years for breast, colorectal, oral cavity, oesophageal (squamous cell carcinoma), and pharyngeal cancers, and 8–9 years for laryngeal and liver cancers [17]).

The alcohol-attributable fractions (AAFs; i.e., the PAFs for alcohol) were estimated by combining data on the prevalence of former drinkers (P_{FD}) and current drinkers (P_{CD}) with the corresponding relative risks (RR) using Formula S3. Alcohol consumption among current drinkers (x) was modelled using an upper integration limit of 150 grams of pure alcohol per day. The upper limit of 150 grams per day was based on the observation that very heavy consumers of alcohol do not sustain alcohol consumption above 150 grams per day for prolonged periods of time [18].

[Formula S3]

$$AAF = \frac{P_{FD}(RR_{FD} - 1) + \int_{>0}^{150} P_{CD}(x)(RR_{CD}(x) - 1)dx}{P_{FD}(RR_{FD} - 1) + \int_{>0}^{150} P_{CD}(x)(RR_{CD}(x) - 1)dx + 1}$$

The fraction of ischaemic stroke, ischaemic heart disease, and injuries in the AAF are estimated on the basis of formulas S4 and S5. These formulae incorporate the prevalences of both former drinkers and

current drinkers combined with the corresponding RRs. In the case of current drinkers, Formula S5 also accounts for the patterns of alcohol consumption (i.e., the prevalence of current drinkers who engage in HED (P_{CD_HED}) and who do not engage in HED (P_{CDN_HED})).

[Formula S4]

$$AAF = \frac{P_{FD}(RR_{FD} - 1) + P_{CD}(RR_{CD} - 1)}{P_{FD}(RR_{FD} - 1) + P_{CD}(RR_{CD} - 1) + 1}$$

[Formula S5]

$$P_{CD}(RR_{CD} - 1) = \int_{>0}^{60} P_{CDN\_HED}(x) RR_{CDN\_HED}(x) dx + \int_{>0}^{60} P_{CD\_HED}(x) RR_{CD\_HED}(x) dx + \int_{60}^{150} P_{CD}(x) RR_{CD\_HED}(x) dx - P_{CD}$$

The AAF for MVAs affecting the driver was applied to the mortality and morbidity estimates. The AAF for MVAs affecting people other than the driver (non-drivers (nd)) was estimated using Formula S6 below, and used data on the deaths and AAFs for MVAs affecting the driver (d) by sex (indexed by p) and age (indexed by i). This method assumes that accidents involving an intoxicated driver also involve an equal number of passengers, as compared to accidents involving non-intoxicated drivers. This method also does not account for non-intoxicated drivers killed or injured by intoxicated drivers.

[Formula S6]

$$AAF_{nd} = \frac{\sum_{p=1}^{pn} \sum_{i=1}^{in} D_{p,i} \cdot AAF_{p,i}}{\sum_{p=1}^{pn} \sum_{i=1}^{in} D_{p,i}}$$

Estimation of alcohol-attributable fractions

Estimates of uncertainty (i.e., 95% Uncertainty Intervals (UIs)) were derived using 1000 simulated estimates generated through a Monte Carlo-type approach. As outlined in the main paper, each simulated estimate of alcohol consumption— spanning APC, drinking status, heavy episodic drinking (HED) —was produced based on the respective underlying uncertainty distributions. To characterize uncertainty in alcohol consumption (measured in grams per day), the shape parameter of the gamma distribution was simulated based on the error estimate published by Kehoe and colleagues (who found the shape parameter of the gamma distribution to be consistent across all surveys for men and women separately).[7,8] The scale of the simulated gamma distribution was determined based on APC, the prevalence of current drinkers and the simulated scale function of the gamma distribution.

Simulated estimates of the alcohol-attributable burden of disease were derived from the simulated alcohol consumption estimates, in conjunction with the corresponding simulated relative risk functions. Since error estimates for health outcomes (such as deaths, YLL, YLD, and DALYs) and population data were unavailable, these sources of error were not incorporated into the simulations.

To construct the 95% UIs the 2.5th and 97.5th percentiles of the 1000 simulations were utilized.

Table S6. Causes and sources of relative risks and causality

Cause code	WHO Global Health Observatory Category	ICD-10 coding	Relative risk*	Causality
10	I. Communicable, maternal, perinatal, and nutritional conditions	A00−B99, D50−53, D64.9, E00−02, E40−46, E50−64, G00−04, G14, H65−66, J00−J22, N70−N73, O00−O99, P00−P96, U04		
20	A. Infectious and parasitic diseases	A00−B99, G00−04, G14, N70−73, P37.3, P37.4		
30[1]	1 Tuberculosis	A15−19, B90	19	20
100	3 HIV/AIDS	B20−24	21	22,23
380[1]	B. Respiratory infections	H65−66, J00−22, P23, U04		
390	1 Lower respiratory infections	J09−22, P23, U04	24	24-26
600	II. Noncommunicable diseases	C00−97, D00−48, D55−64 (minus D64.9), D65−89, E03−07, E10–34, E65–88, F01–99, G06–98 (minus G14), H00–61, H68–93, I00–99, J30–98, K00–92, L00–98, M00–99, N00–64, N75–98, Q00–99, X41–42, X44, X45, R95		
610	A. Malignant neoplasms	C00–97		
620	1 Mouth and oropharynx cancers	C00–14		
621	a. Lip and oral cavity	C00–08	27	28,29
623	c. Other pharyngeal cancers	C09–10, C12–14	27	28,29
630	2 Oesophagus cancer	C15	27	28,29
650	4 Colon and rectum cancers	C18–21	30	28,29
660	5 Liver cancer	C22	31	28,29
700	9 Breast cancer	C50	32	28,29
710	10. Cervix uteri cancer	C53	21	22,23
753	19 Larynx cancer	C32	27	28,29
800	C. Diabetes mellitus	E10–14 (minus E10.2–10.29, E11.2–11.29, E12.2, E13.2–13.29, E14.2)	33	34,35
820	E. Mental and substance use disorders	F04–99, G72.1, Q86.0, X41–42, X44, X45		
860	4 Alcohol use disorders	F10, G72.1, Q86.0, X45		
940	F. Neurological conditions	F01–03, G06–98 (minus G14, G72.1)		
970	3 Epilepsy	G40–41	36	37-39
1100	H. Cardiovascular diseases	I00–99		
1120	2 Hypertensive heart disease	I10–15	40	41,42
1130[1]	3 Ischaemic heart disease	I20–25	43-45	46-48
1140	4 Stroke	I60–69		
1141[1]	a. Ischaemic stroke	G45–46.8, I63–63.9, I65–66.9, I67.2–67.848, I69.3–69.4	43,49	48,50,51
1142[1]	b. Haemorrhagic stroke	I60–62.9, I67.0–67.1, I69.0–69.298	52	48,50,51
1150	5 Cardiomyopathy, myocarditis, endocarditis	I30-33, I38, I40, I42		
1210	J. Digestive diseases	K20–92		
1230*	2 Cirrhosis of the liver	K70**, K74	53	54
1248*	8 Pancreatitis	K85–86	55	54,56-59
1510	III. Injuries			
1520	A. Unintentional injuries	V01–X40, X43, X46–59, Y40–86, Y88, Y89		
1530*	1 Road injury	V01–V04, V06, V09–V80, V87, V89, V99	60	61
1540*	2 Poisonings	X40, X43, X46–48, X49	60	61
1550*	3 Falls	W00–19	60	61
1560*	4 Fire, heat and hot substances	X00–19	60	61
1570*	5 Drowning	W65–74	60	61
1575*	6 Exposure to mechanical forces	W20–38, W40–43, W45, W46, W49–52, W75, W76	60	61
1590*	8 Other unintentional injuries	Rest of V, W39, W44, W53–64, W77–99, X20–29, X50–59, Y40–86, Y88, Y89	60	61
1600	B. Intentional injuries			
1610*	1 Self-harm	X60–84, Y870	60	61
1620*	2 Interpersonal violence	X85–Y09, Y871	60	61

* For Belarus, Estonia, Latvia, Lithuania, Moldova, Russia, and Ukraine, RRs from the Russian cohort study by Zaridze and colleagues were used to model mortality and morbidity from tuberculosis, lower respiratory infections, ischaemic heart disease, ischaemic stroke, haemorrhagic stroke, liver cirrhosis, pancreatitis, road injuries, other unintentional injuries, self-harm ,and interpersonal violence attributable to alcohol consumption.[62,63]

** Includes ICD-10 code: K70 (alcoholic liver disease), and ICD-10 code: K74 (fibrosis and cirrhosis of the liver)

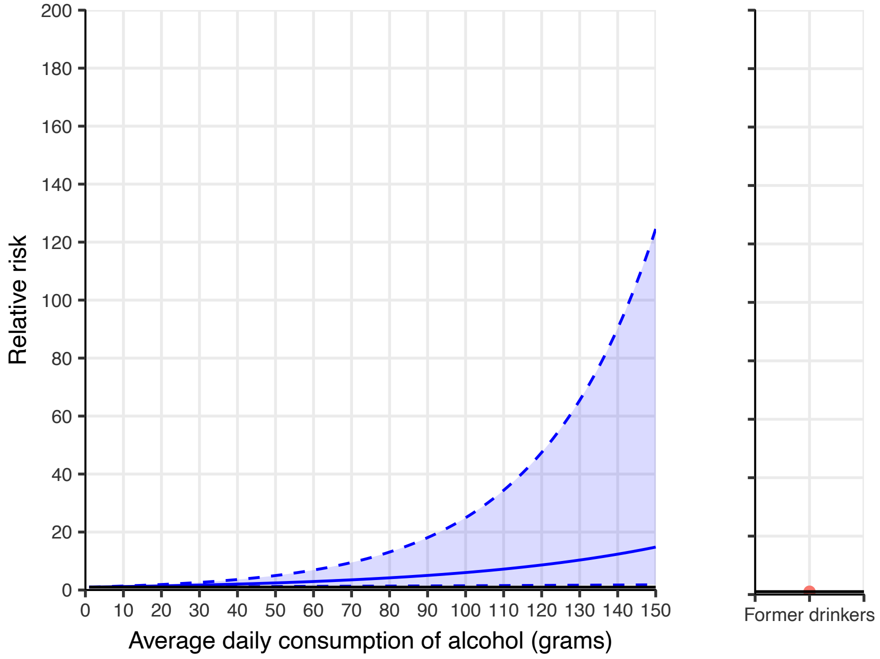

Figure S2. Relative risk for tuberculosis among males

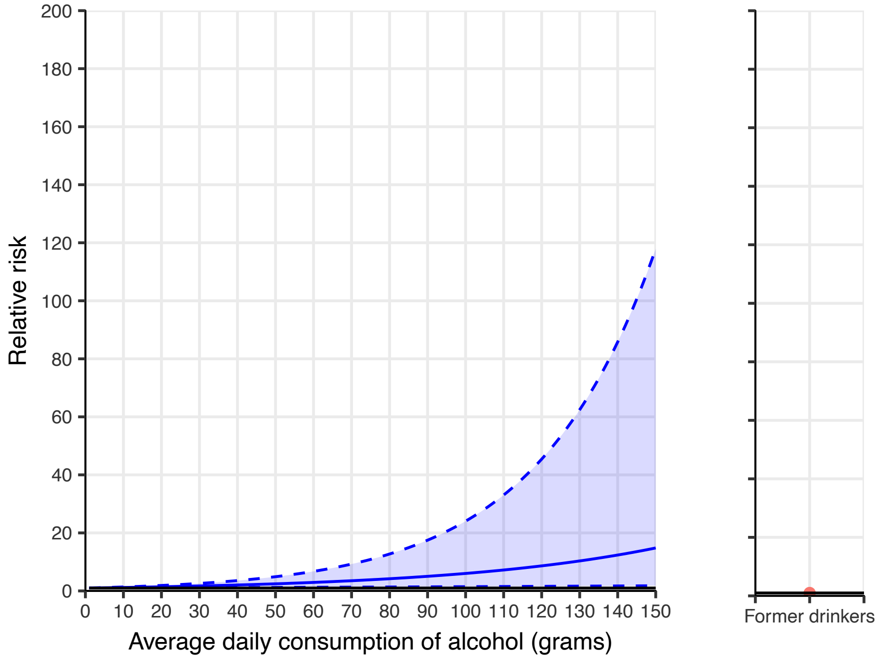

Figure S3. Relative risk for tuberculosis among females

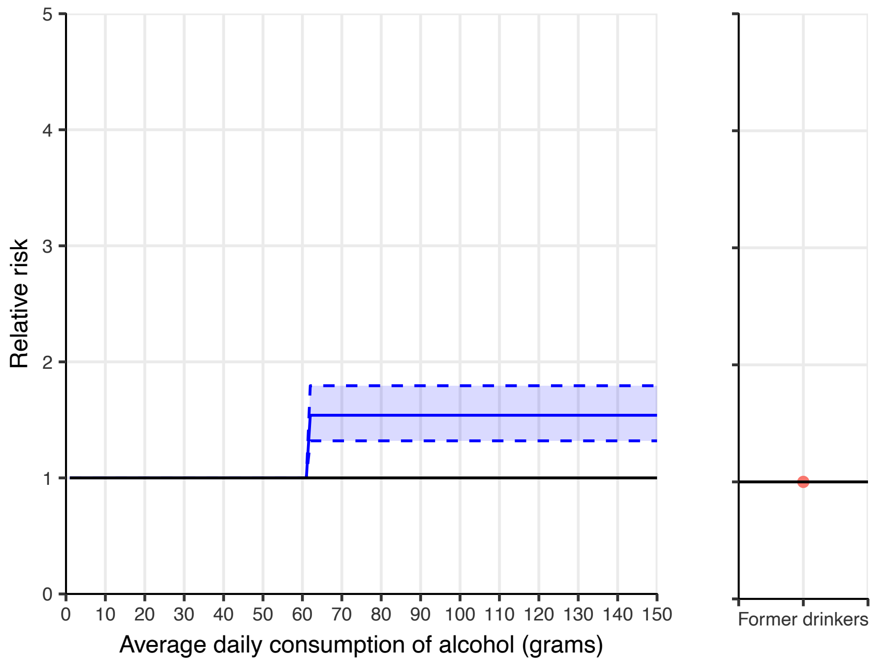

Figure S4. Relative risk for sexually transmitted diseases among males

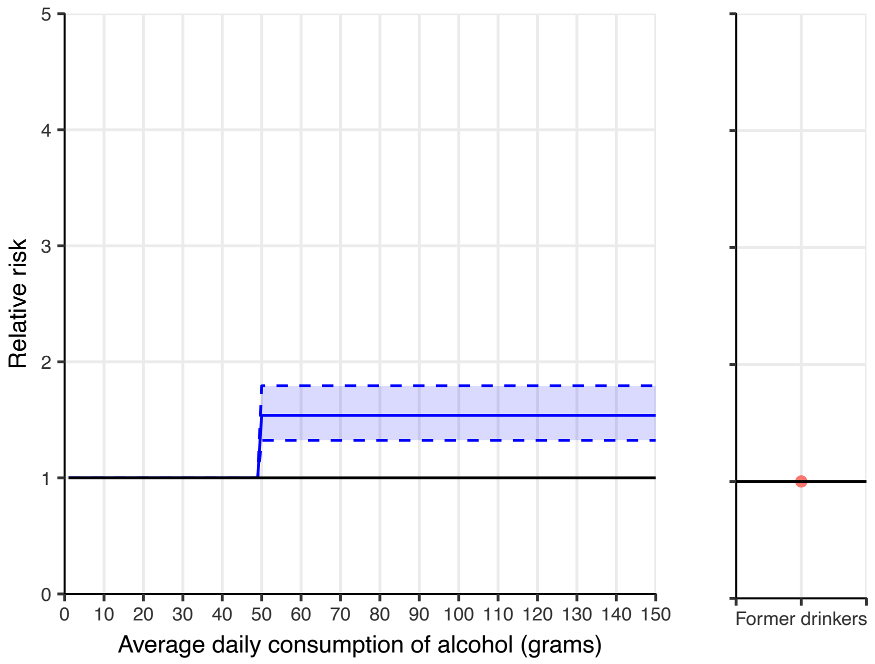

Figure S5. Relative risk for sexually transmitted diseases among females

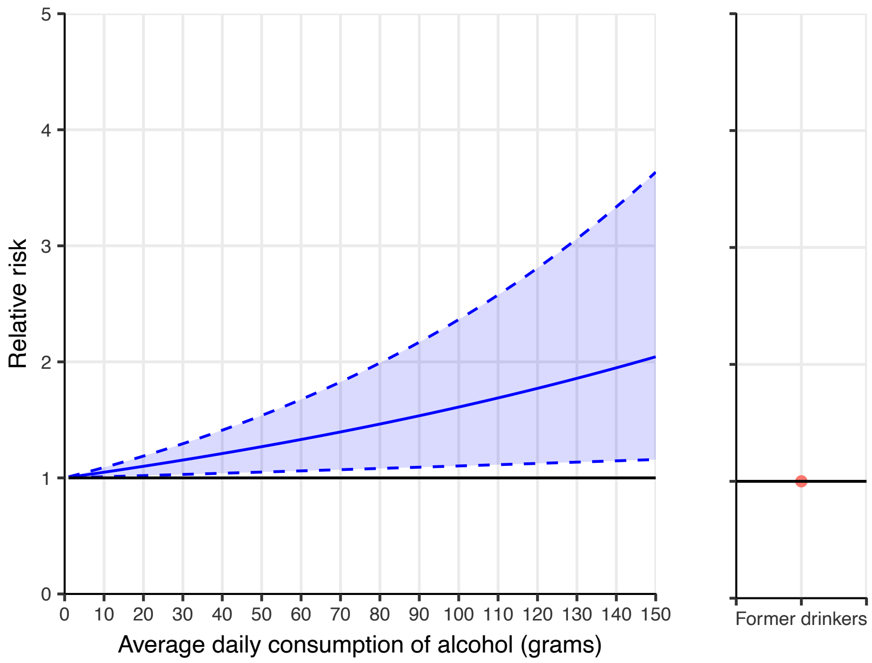

Figure S6. Relative risk for lower respiratory infections among males

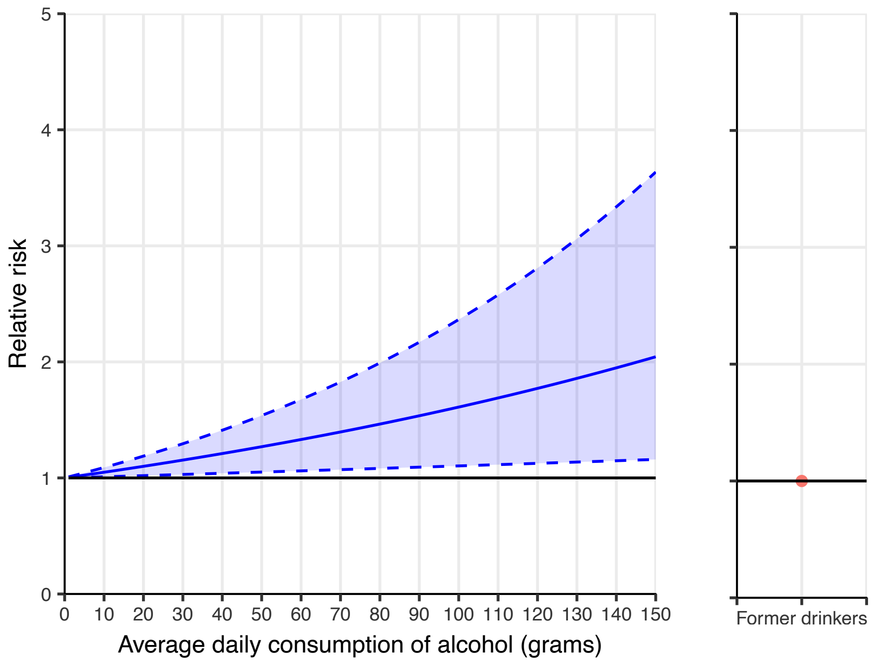

Figure S7. Relative risk for lower respiratory infections among females

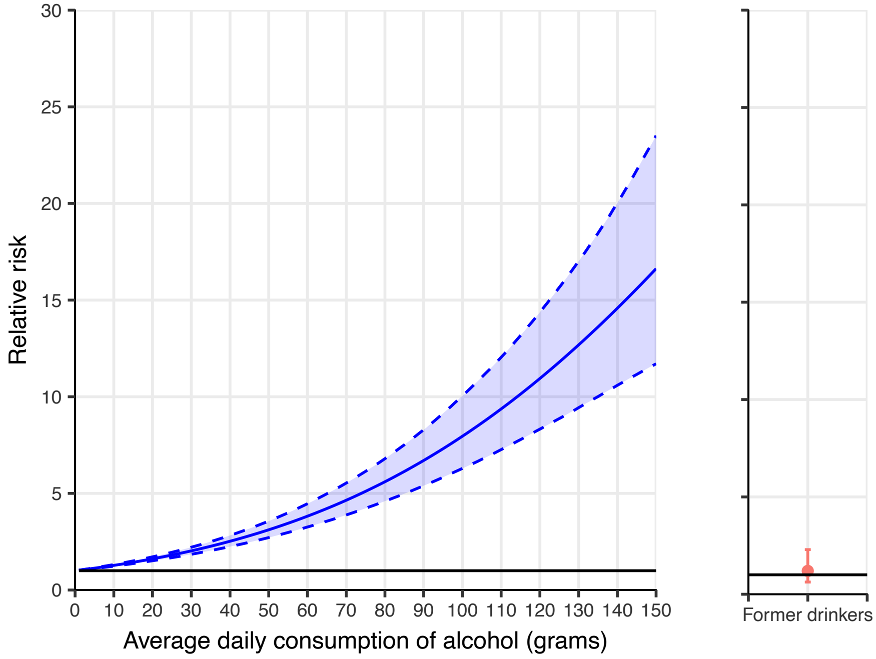

Figure S8. Relative risk for mouth and oropharynx cancers among males

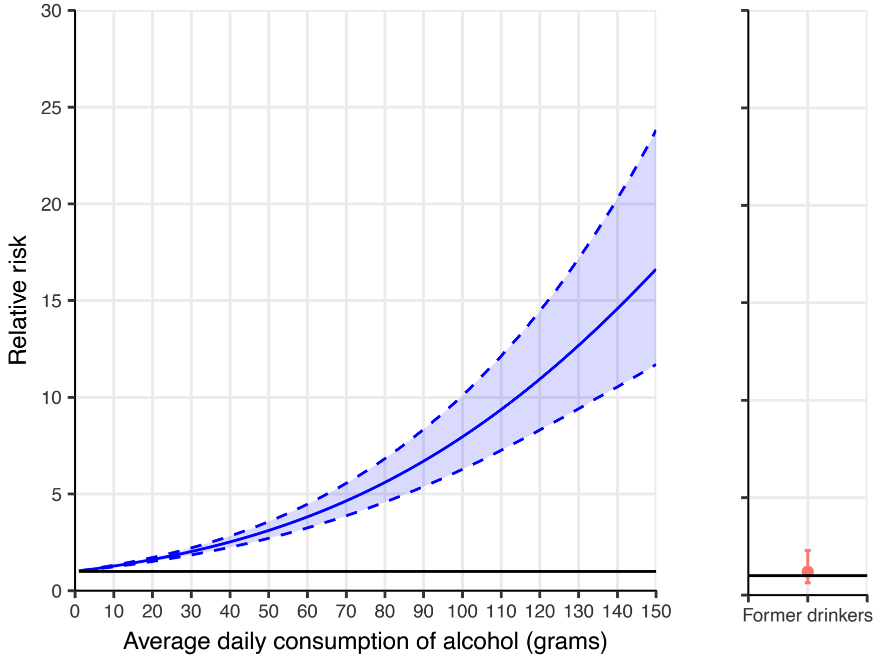

Figure S9. Relative risk for mouth and oropharynx cancers among females

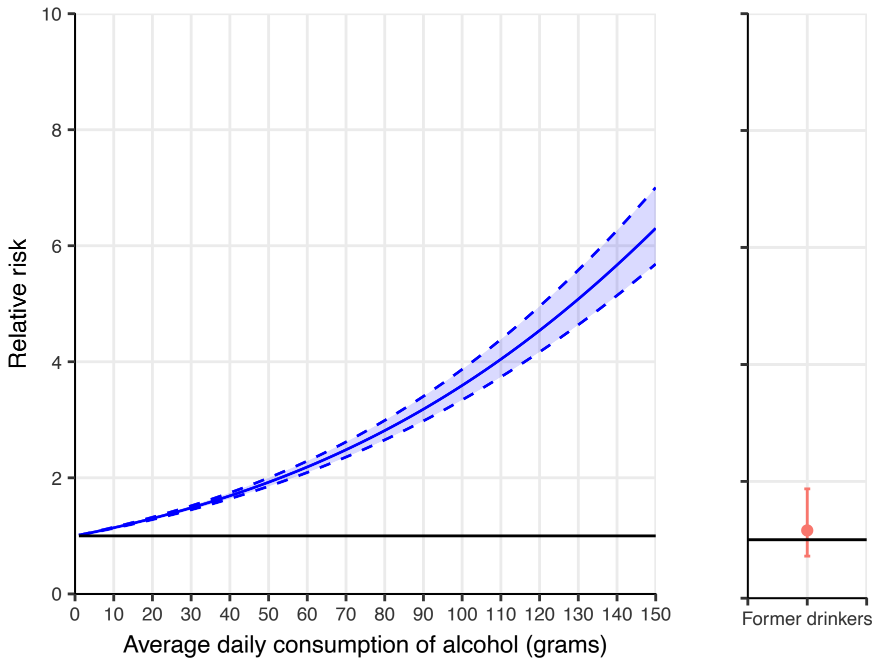

Figure S10. Relative risk for mouth and oesophagus cancer among males

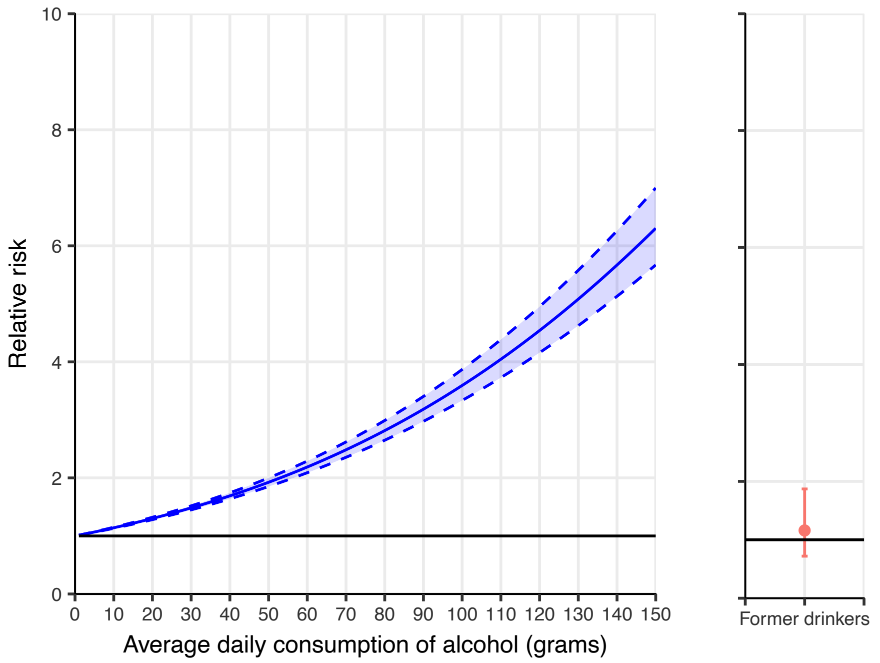

Figure S11. Relative risk for mouth and oesophagus cancer among females

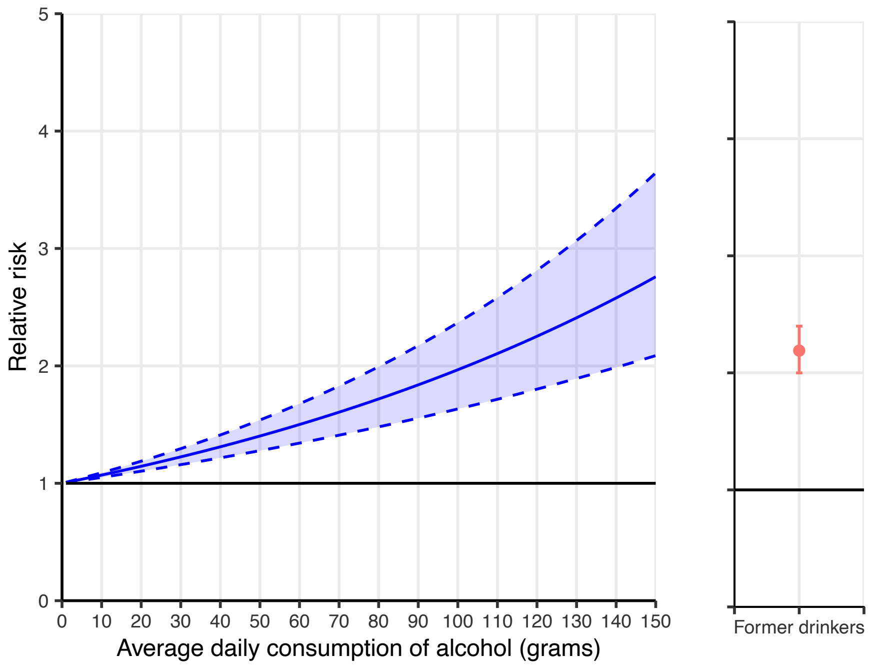

Figure S12. Relative risk for colon and rectal cancer among males

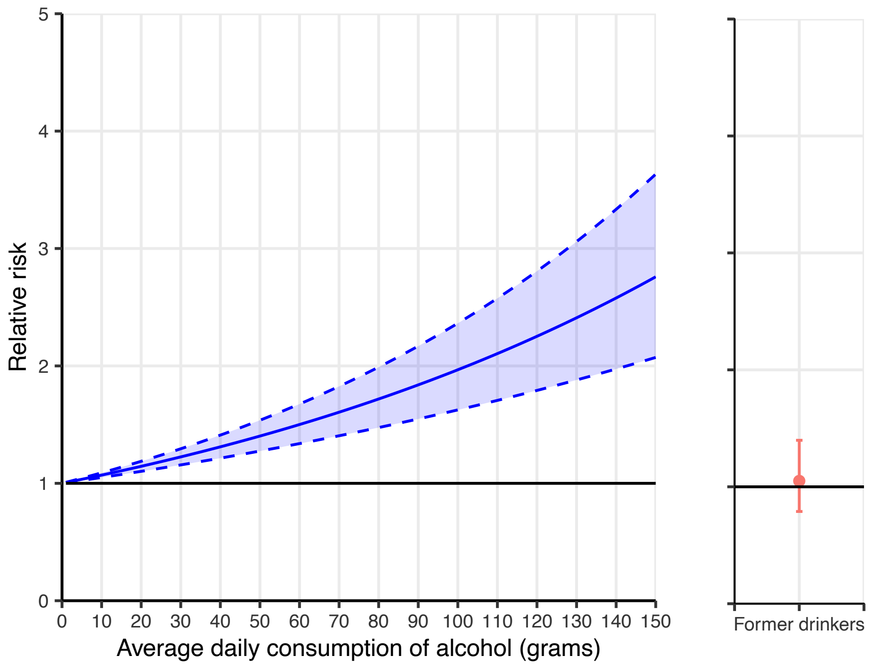

Figure S13. Relative risk for colon and rectal cancer among females

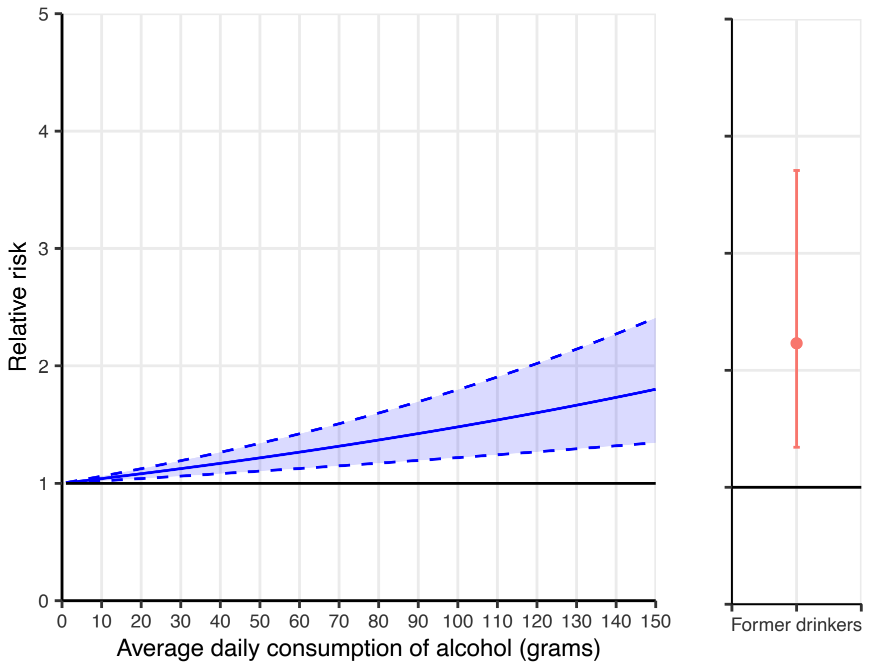

Figure S14. Relative risk for liver cancer among males

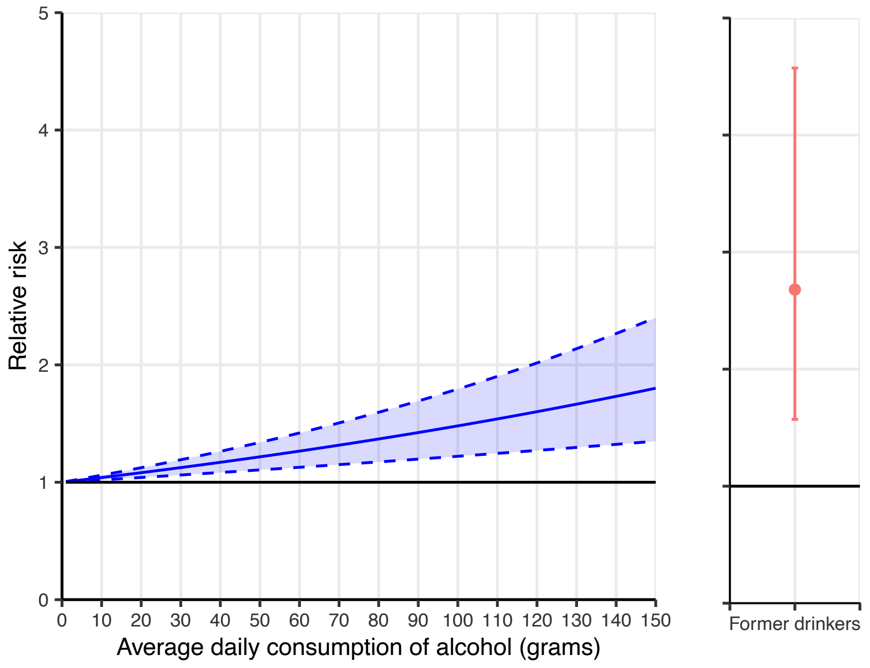

Figure S15. Relative risk for liver cancer among females

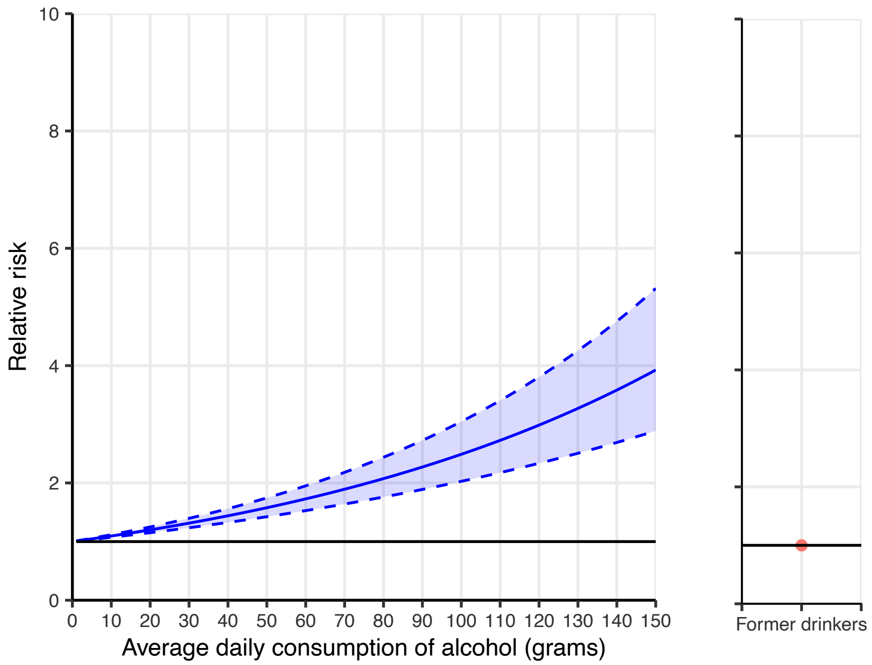

Figure S16. Relative risk for breast cancer among females

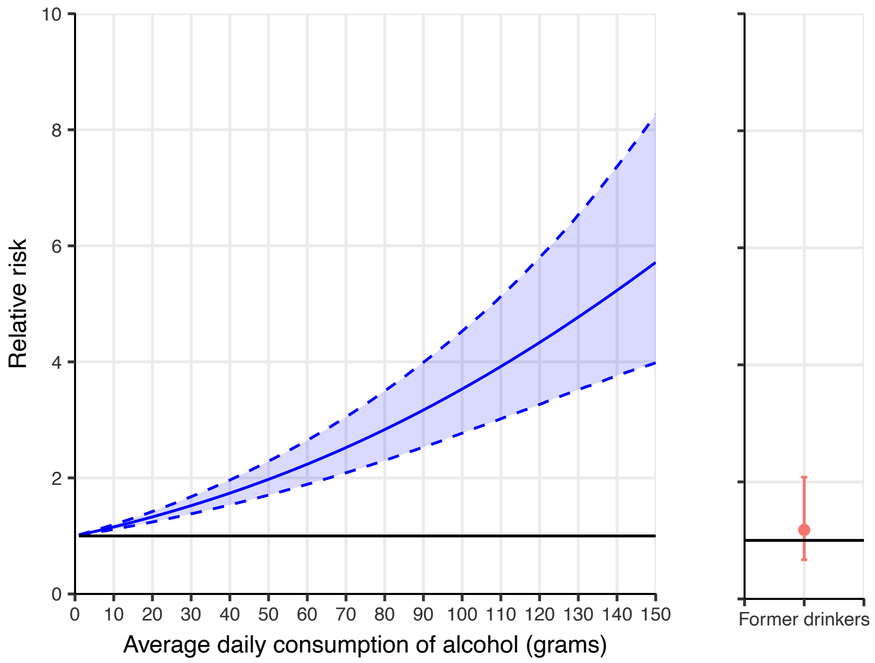

Figure S17. Relative risk for larynx cancer among males

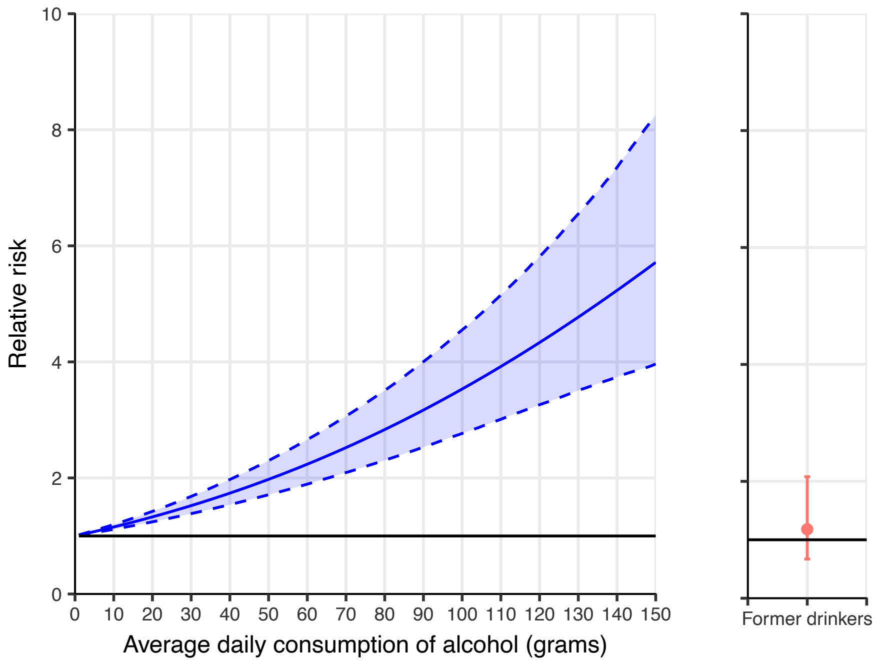

Figure S18. Relative risk for larynx cancer among females

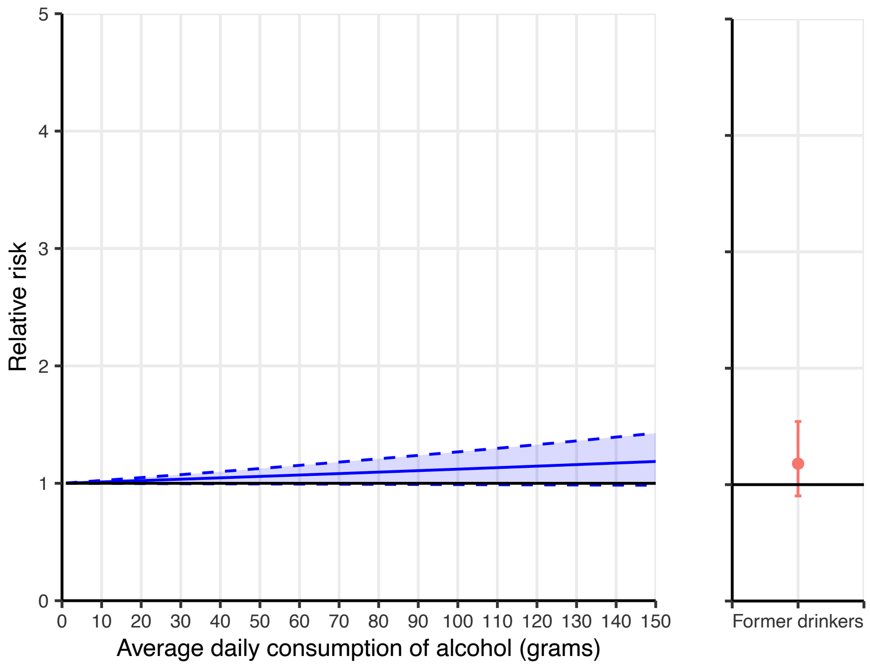

Figure S19. Relative risk for diabetes mellitus among males

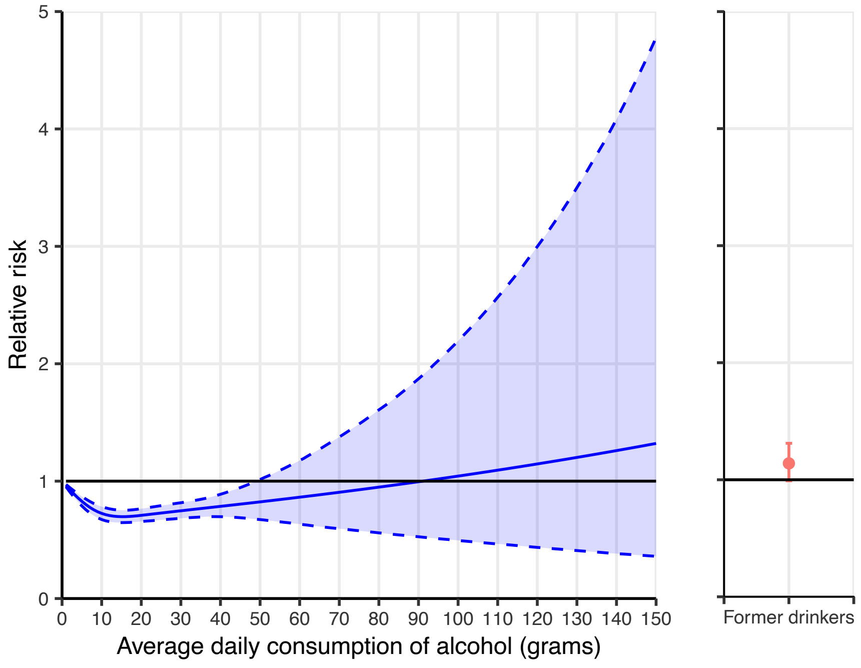

Figure S20. Relative risk for diabetes mellitus among females

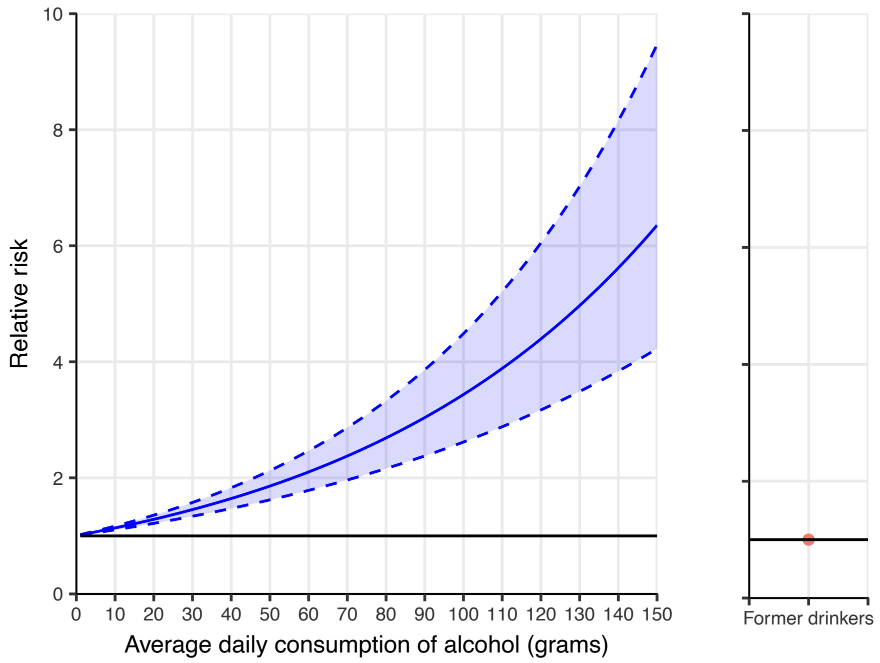

Figure S21. Relative risk for epilepsy among males

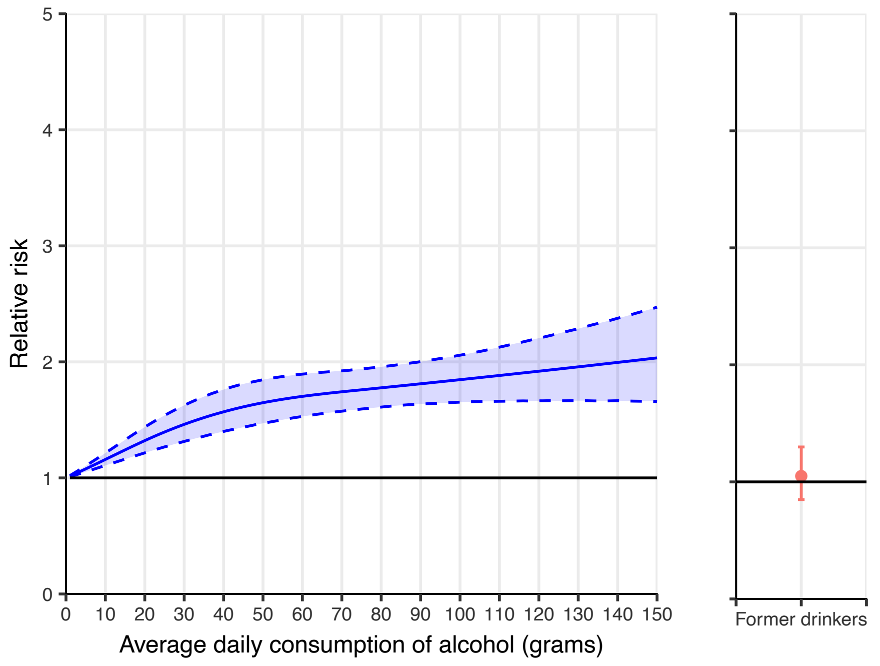

Figure S22. Relative risk for epilepsy among females

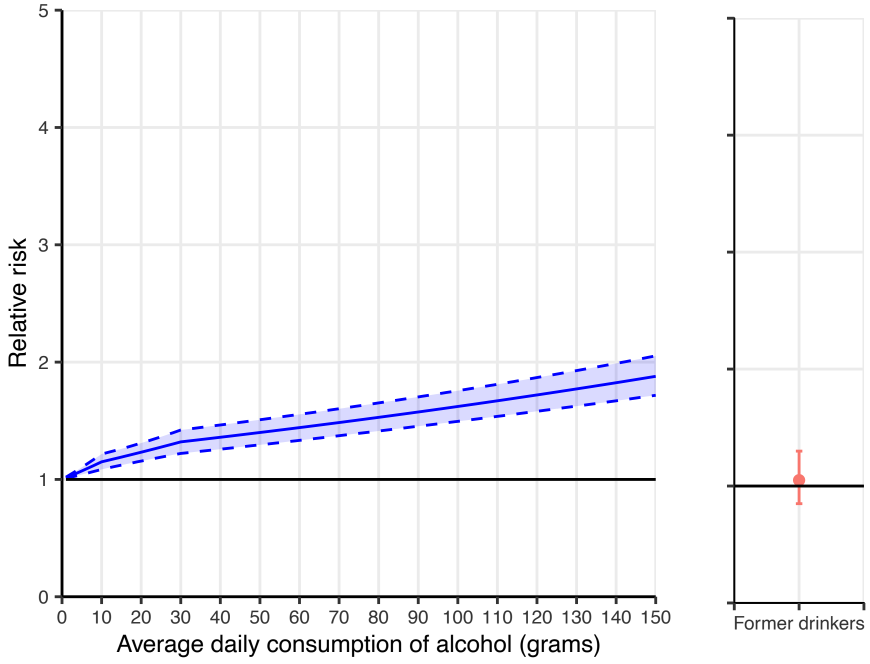

Figure S23. Relative risk for hypertensive heart disease among males

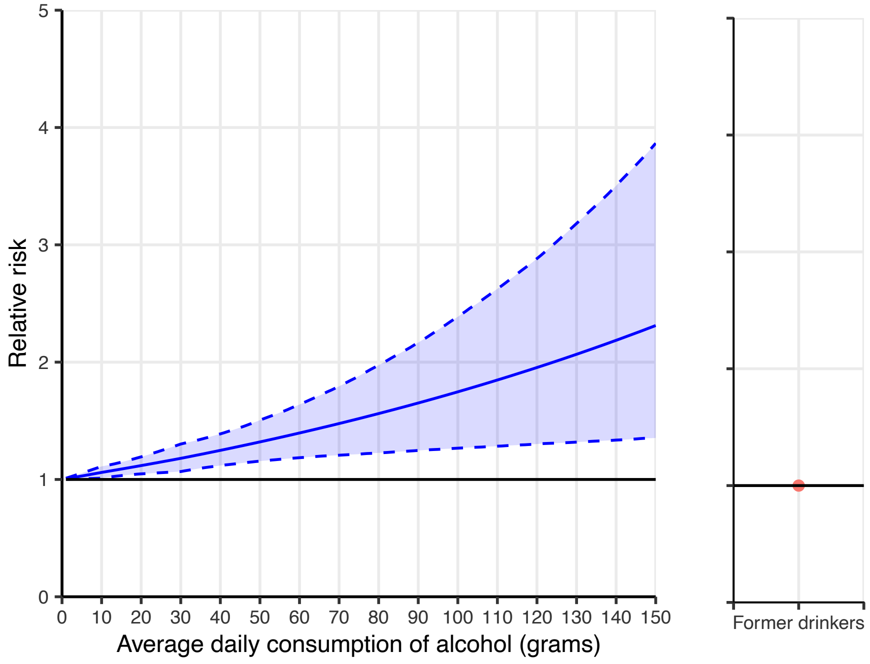

Figure S24. Relative risk for hypertensive heart disease among females

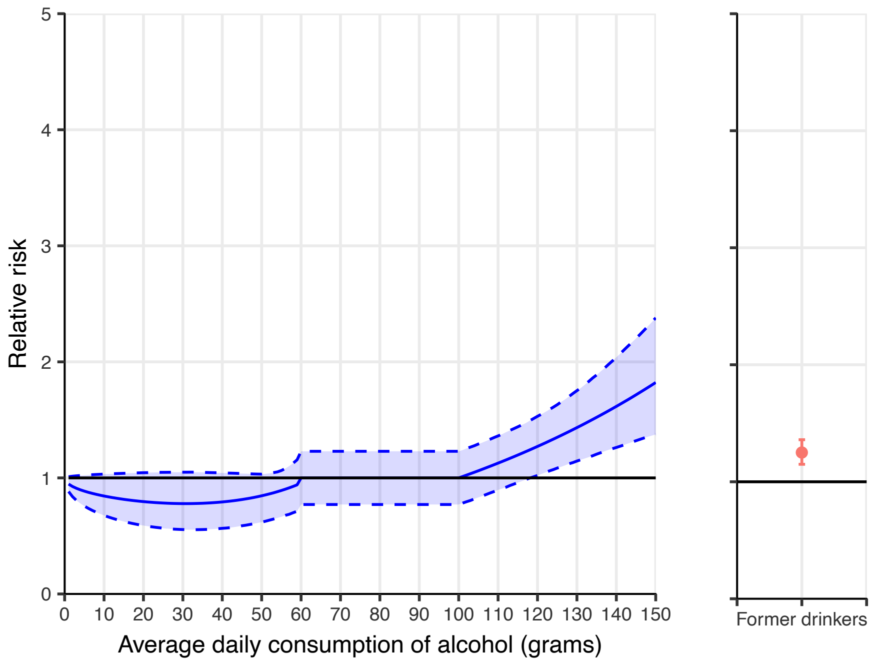

Figure S25. Relative risk for ischaemic heart disease among males 15 to 34 years of age

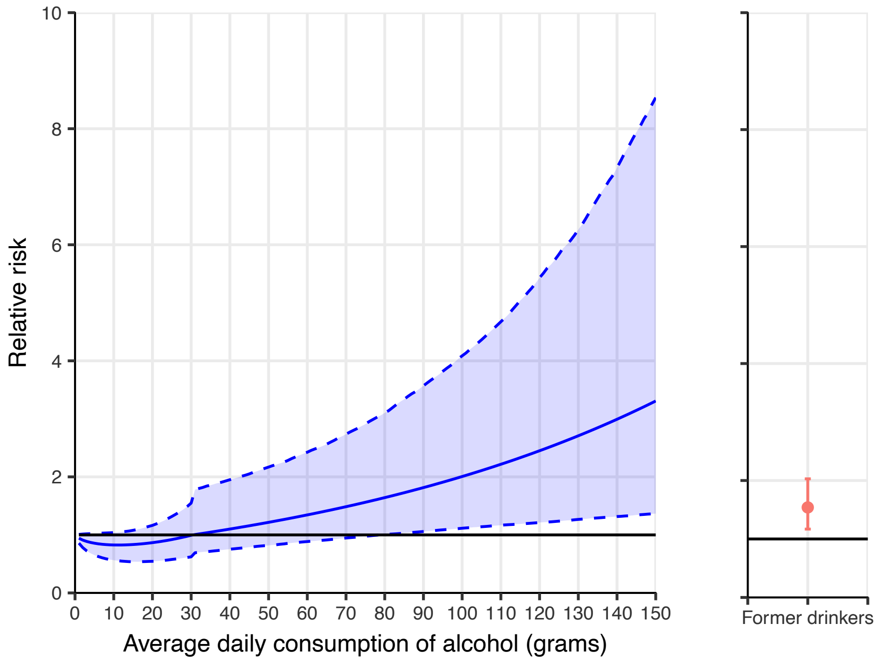

Figure S26. Relative risk for ischaemic heart disease among females 15 to 34 years of age

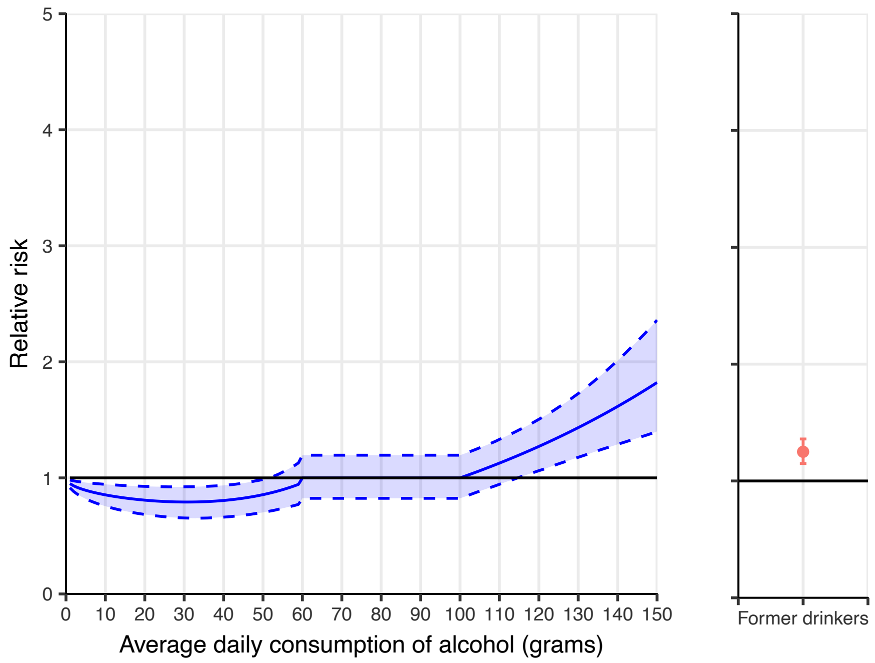

Figure S27. Relative risk for ischaemic heart disease among males 35 to 64 years of age

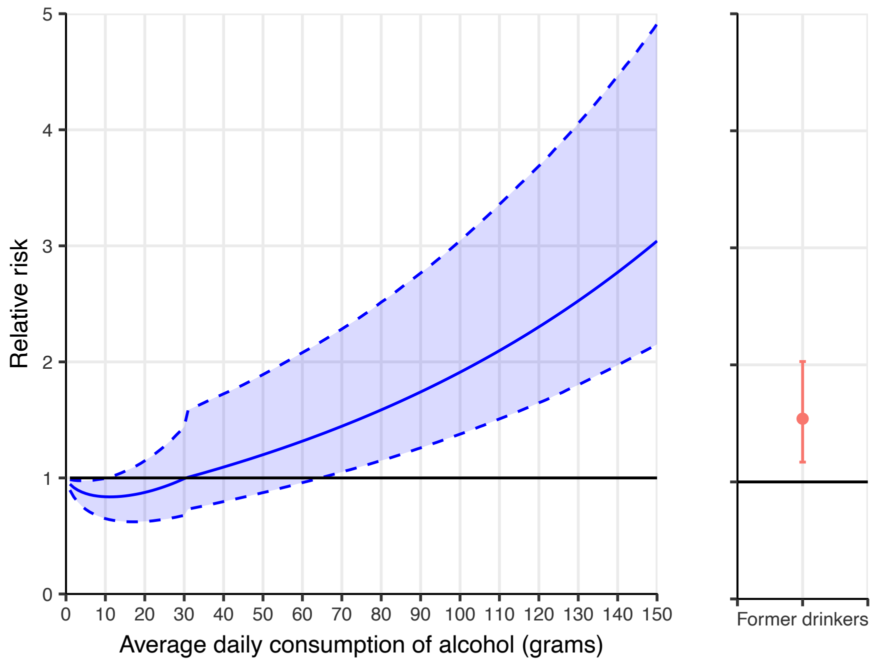

Figure S28. Relative risk for ischaemic heart disease among females 35 to 64 years of age

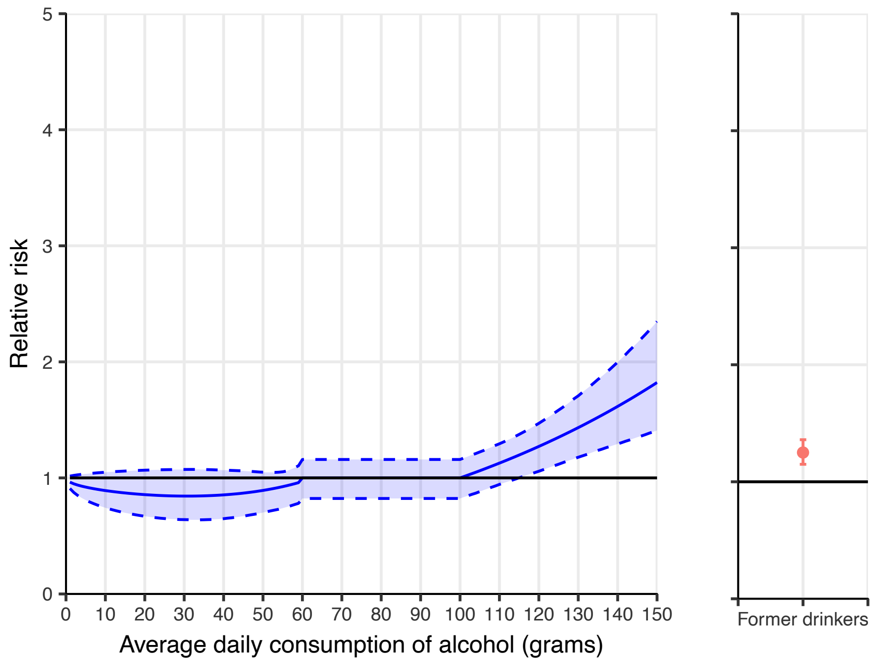

Figure S29. Relative risk for ischaemic heart disease among males 65 years of age and older

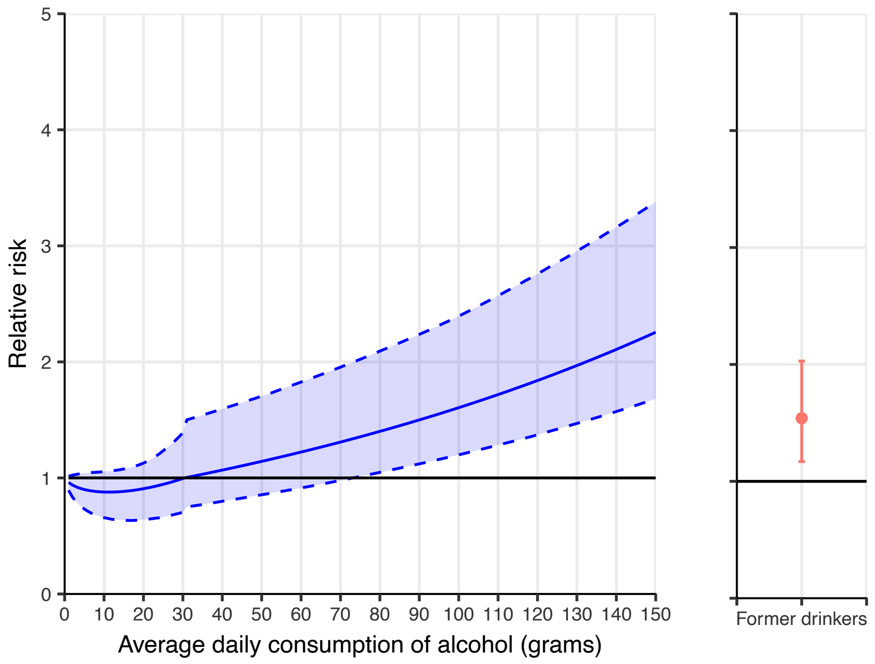

Figure S30. Relative risk for ischaemic heart disease among females 65 years of age and older

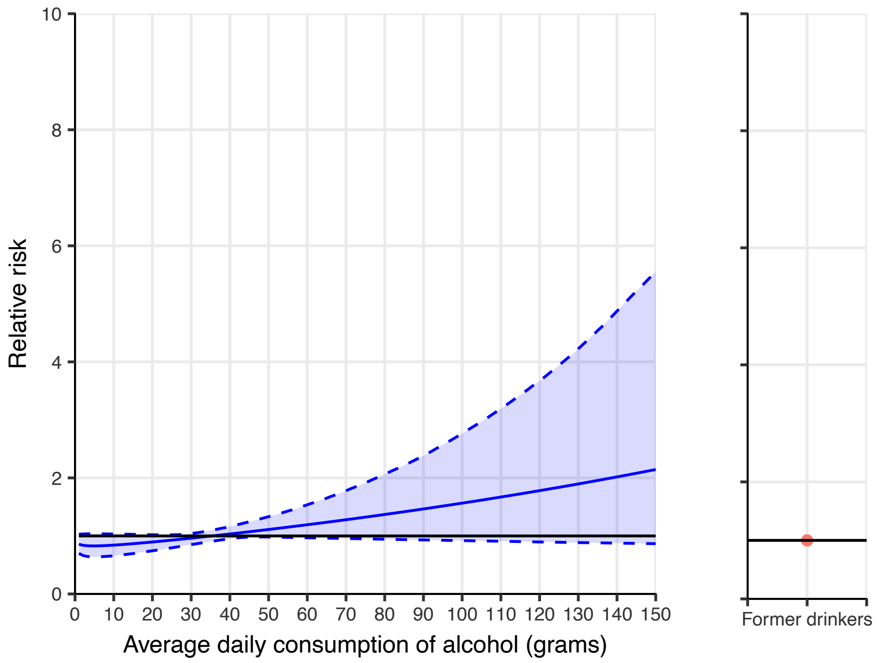

Figure S31. Relative risk for ischaemic stroke among males 15 to 34 years of age

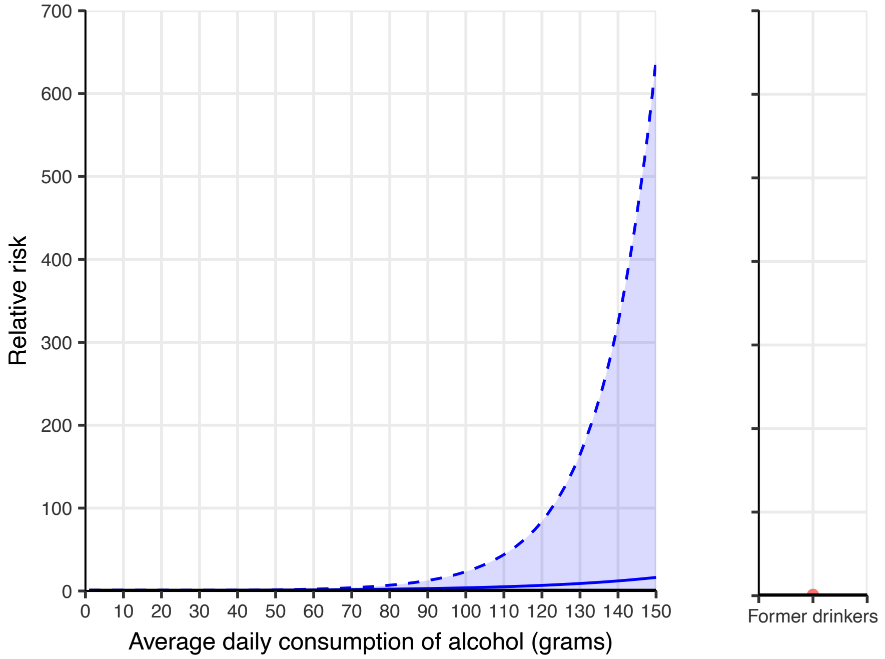

Figure S32. Relative risk for ischaemic stroke among females 15 to 34 years of age

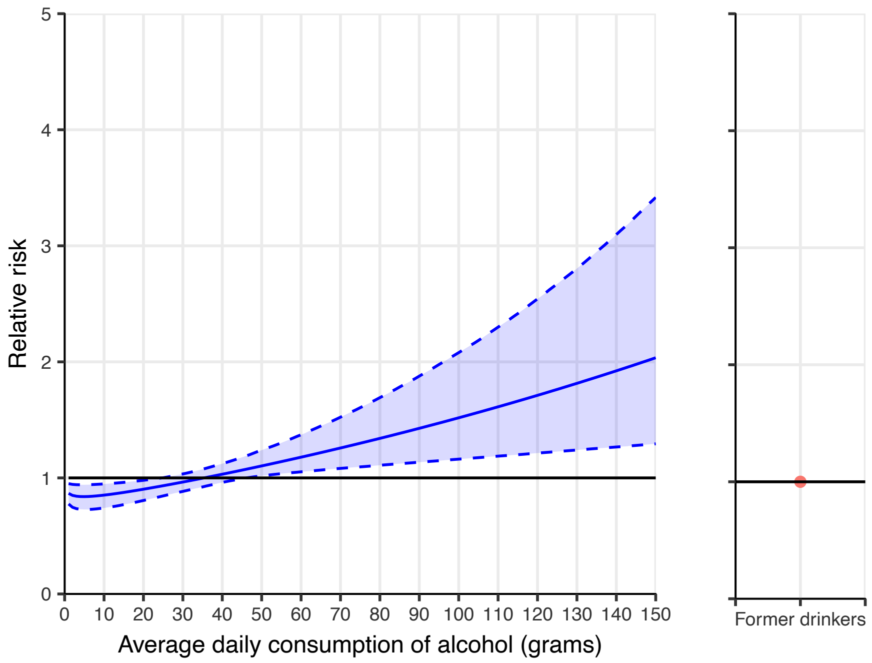

Figure S33. Relative risk for ischaemic stroke among males 35 to 64 years of age

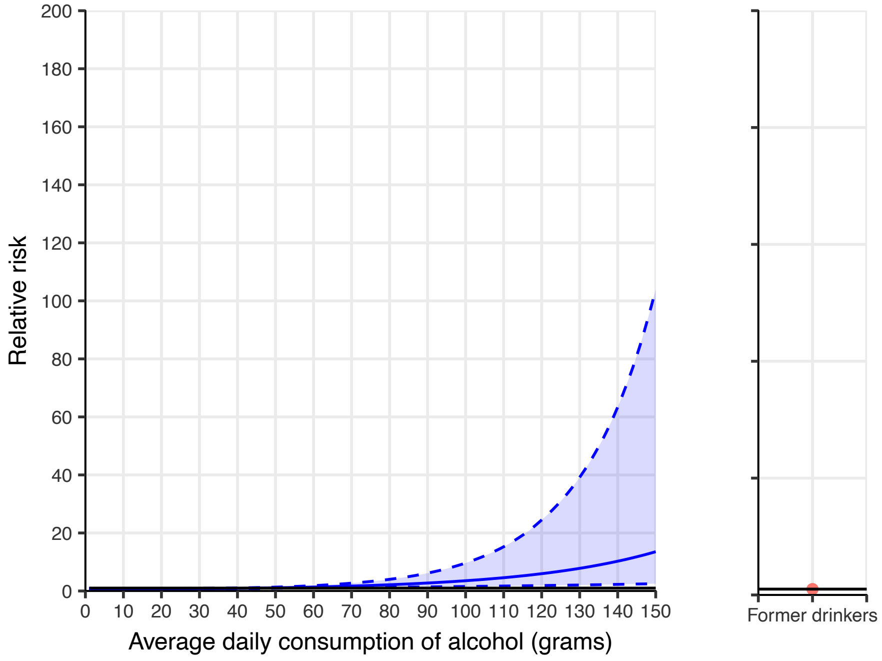

Figure S34. Relative risk for ischaemic stroke among females 35 to 64 years of age

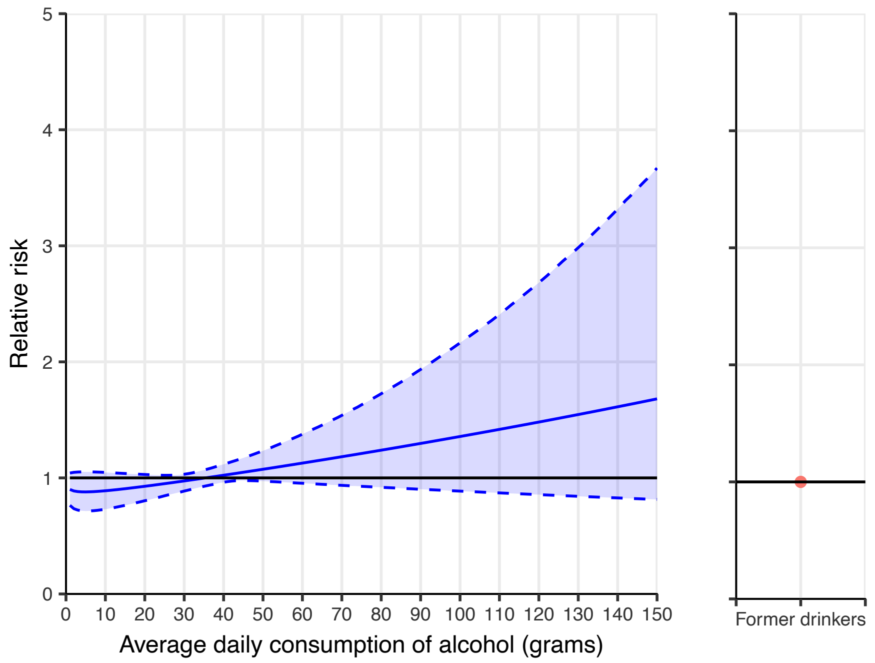

Figure S35. Relative risk for ischaemic stroke among males 65 years of age and older

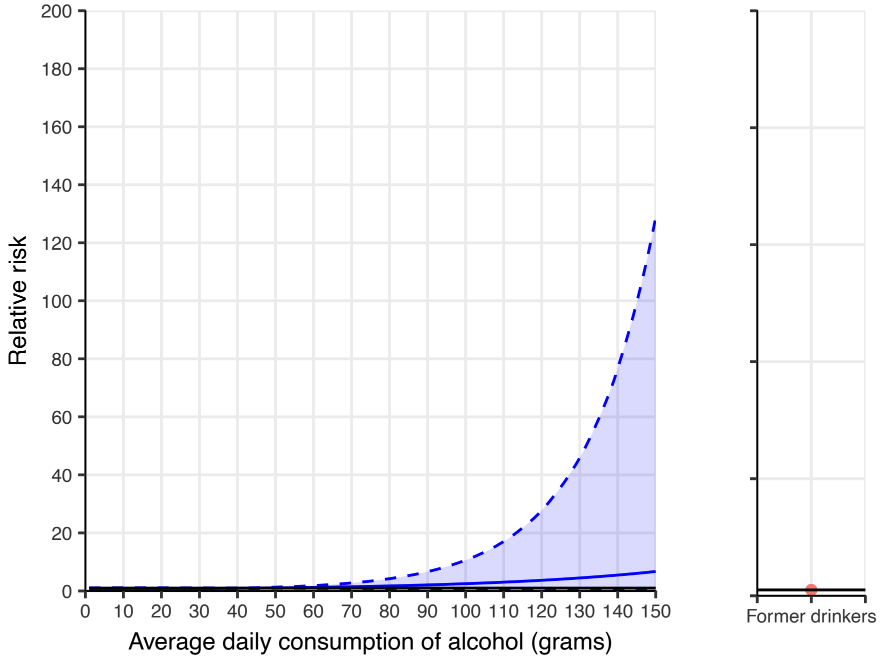

Figure S36. Relative risk for ischaemic stroke among females 65 years of age and older

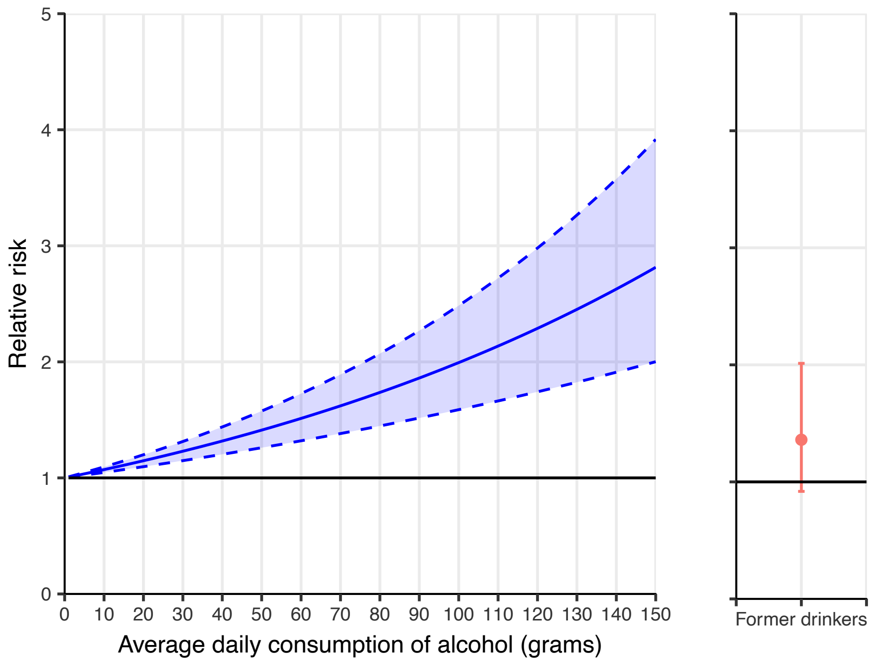

Figure S37. Relative risk for haemorrhagic stroke among males

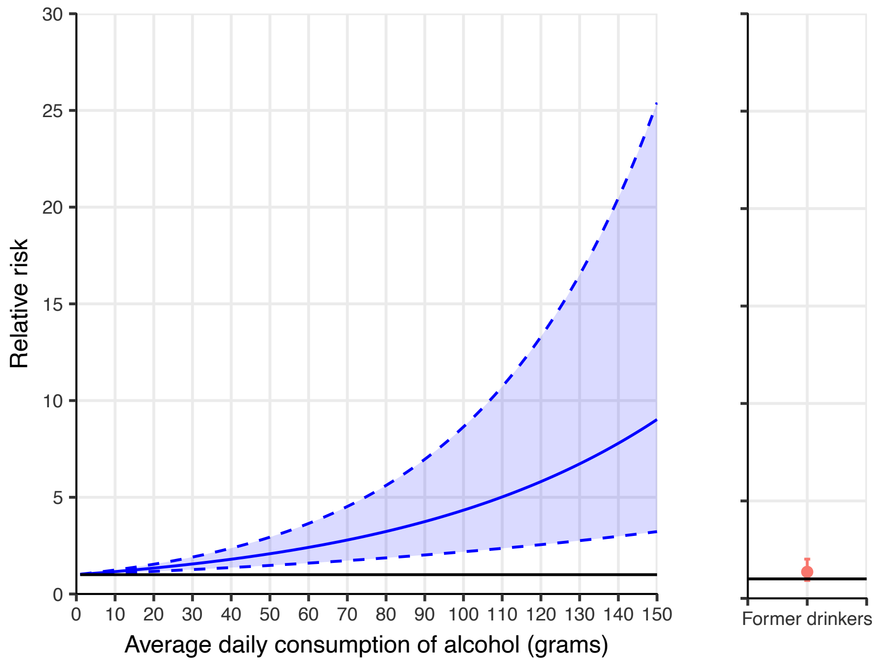

Figure S38. Relative risk for haemorrhagic stroke among females

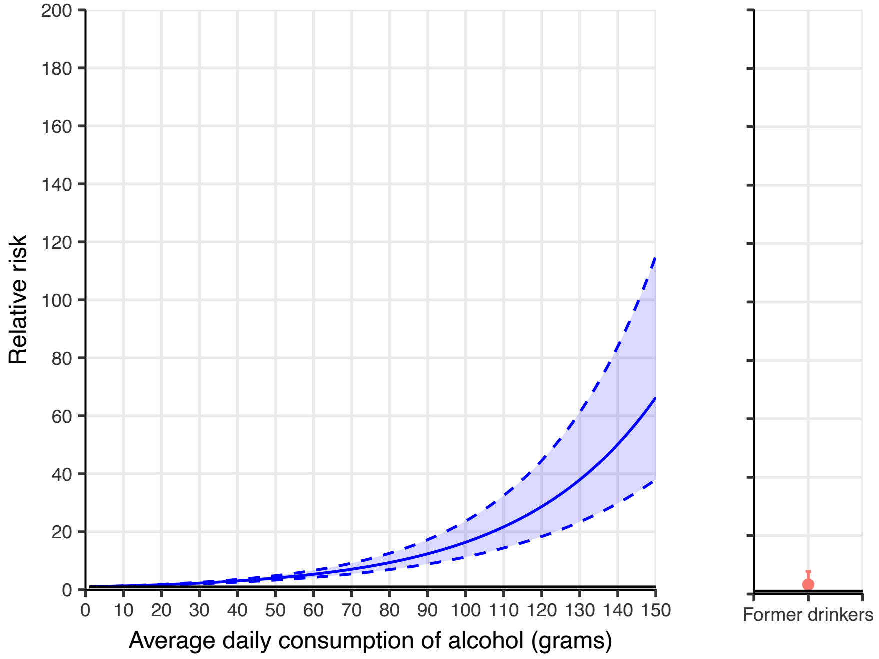

Figure S39. Relative risk for cirrhosis of the liver among males

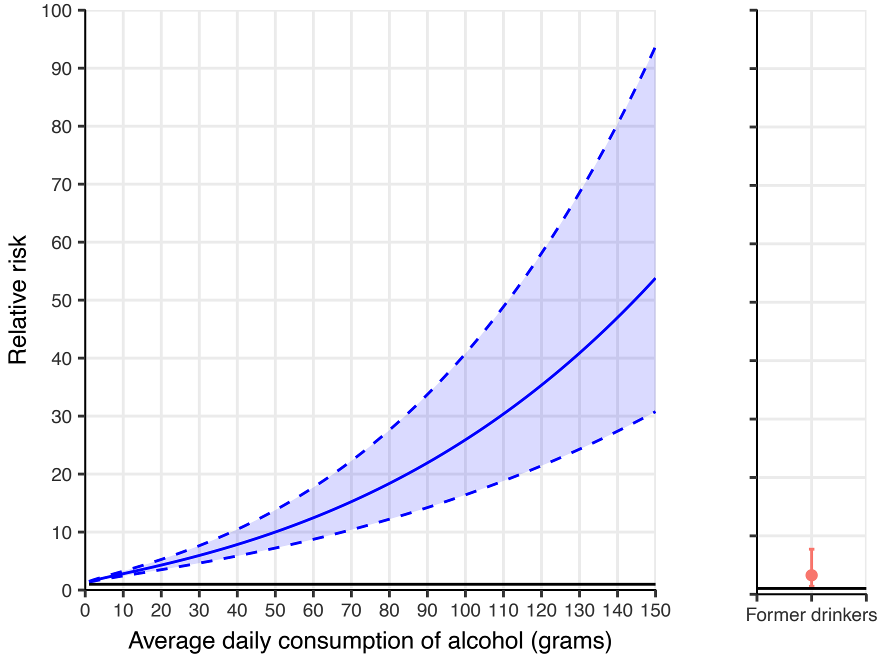

Figure S40. Relative risk for cirrhosis of the liver among females

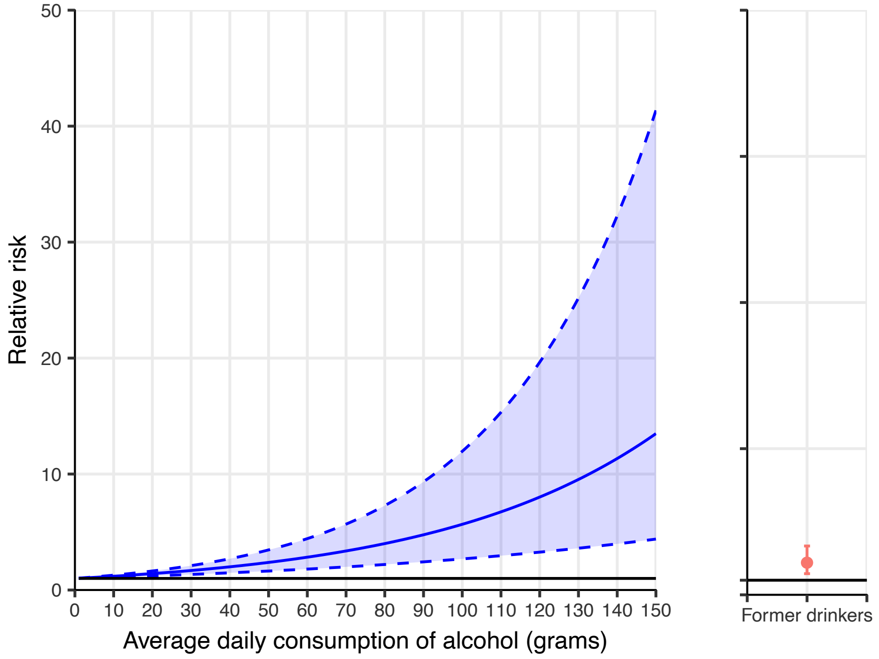

Figure S41. Relative risk for pancreatitis among males

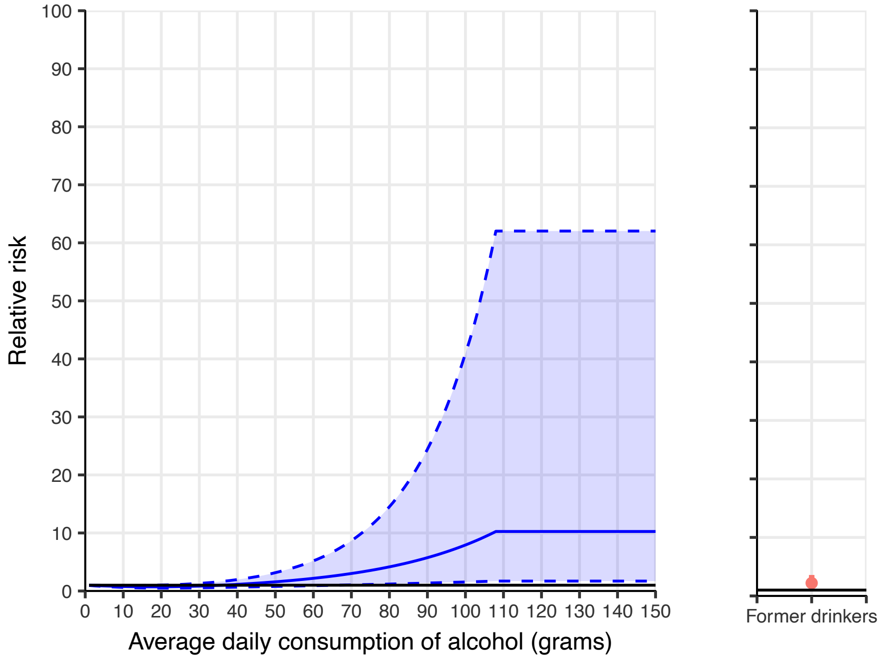

Figure S42. Relative risk for pancreatitis among females

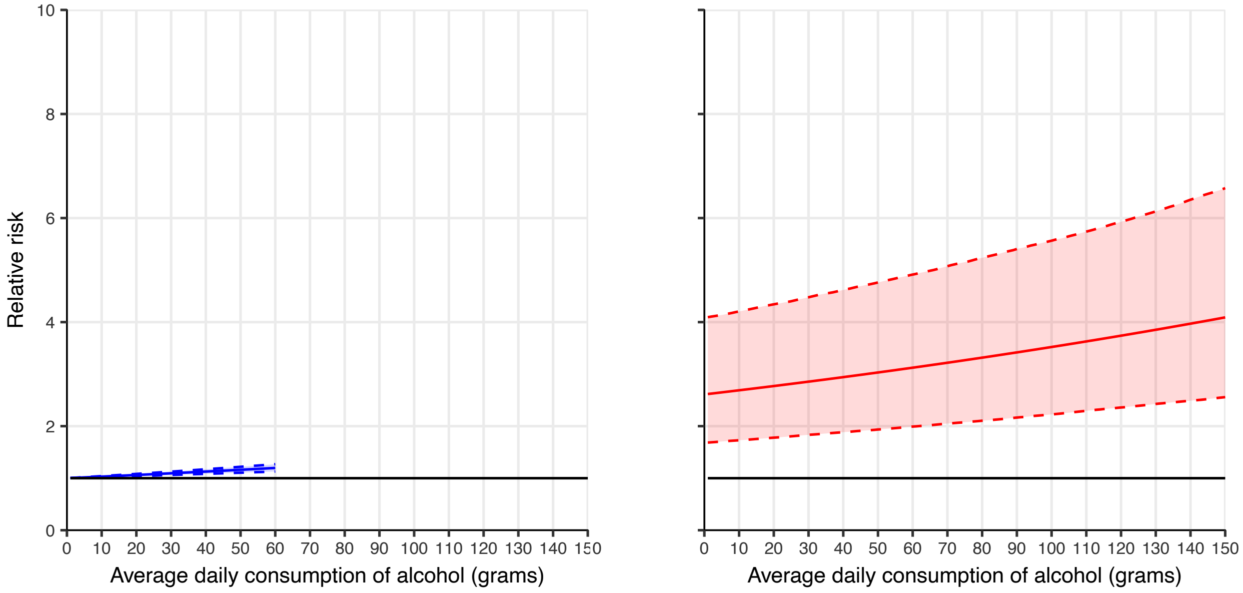

Figure S43. Relative risk for road injury among males and females

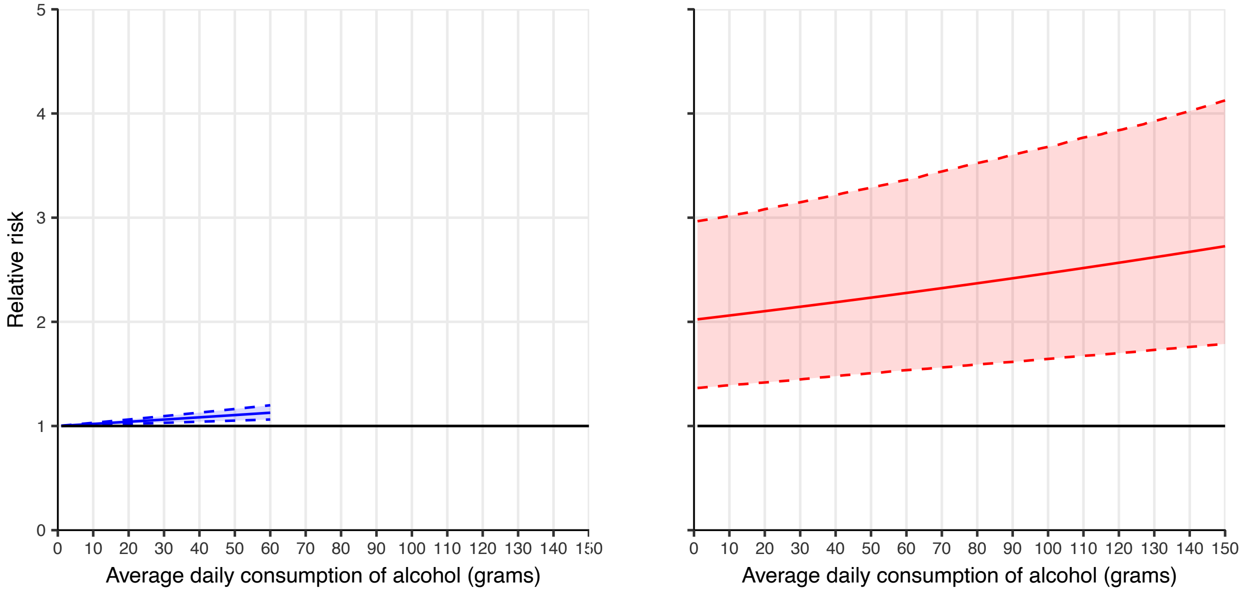

Figure S44. Relative risk for other unintentional injuries among males and females

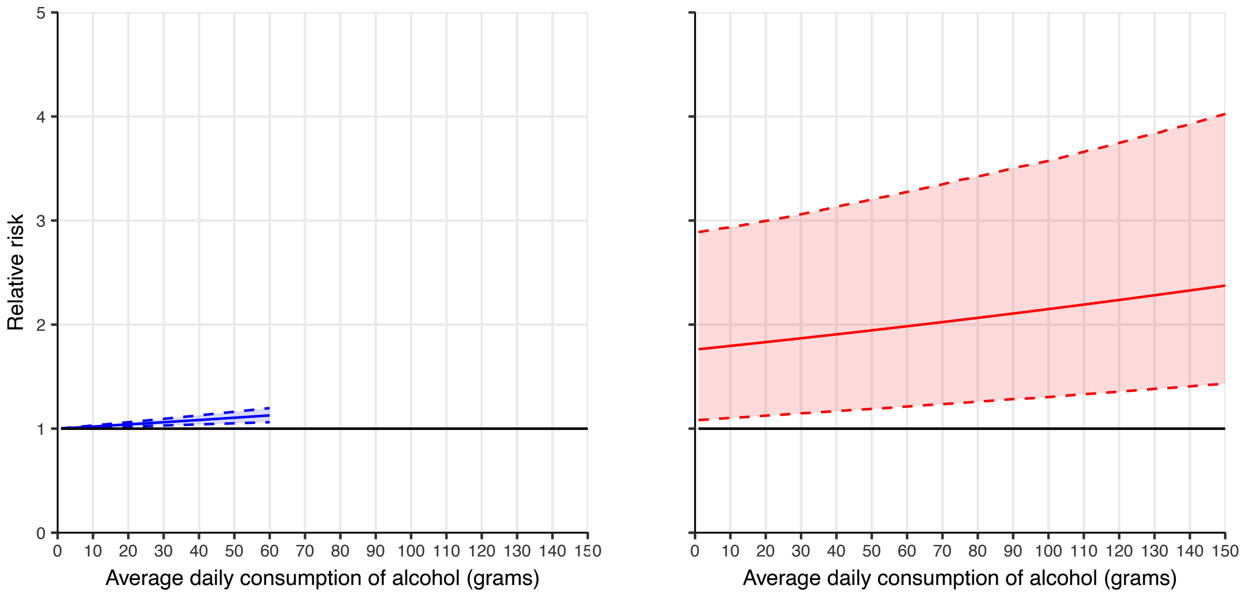

Figure S45. Relative risk for intentional injuries among males and females

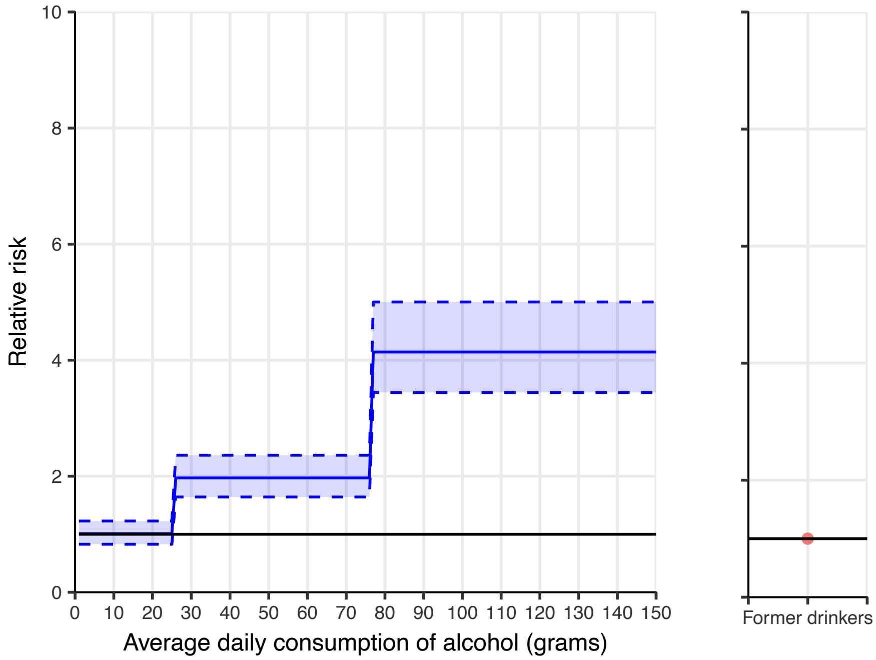

Figure S46. Relative risk for tuberculosis among males for Russia

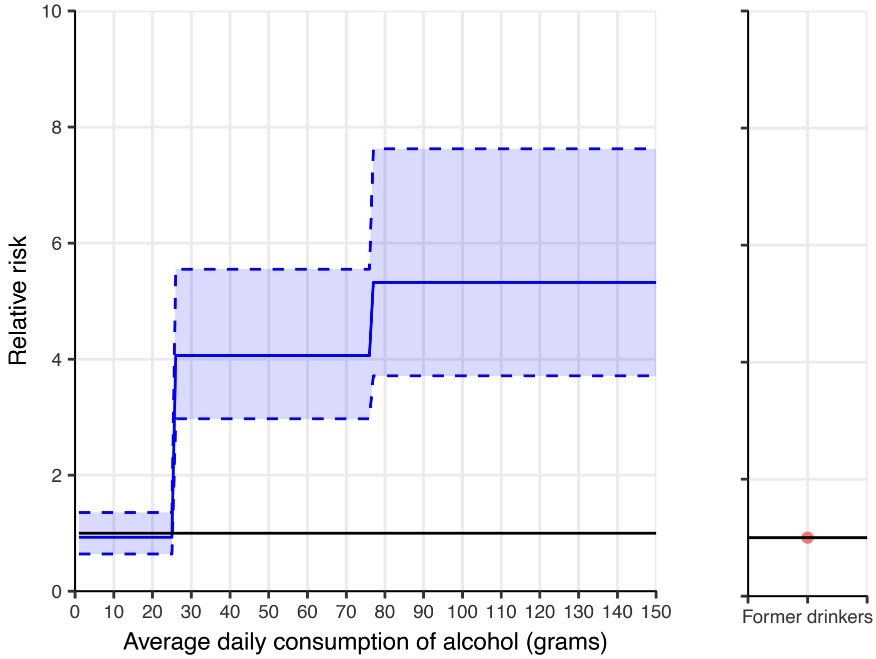

Figure S47. Relative risk for tuberculosis among females for Russia

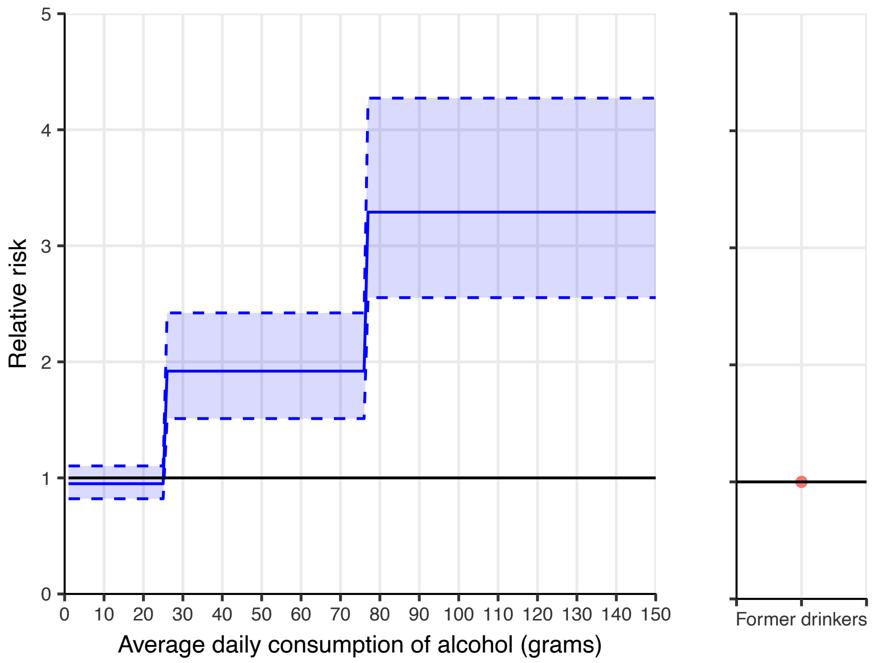

Figure S48. Relative risk for lower respiratory infections among males for Russia

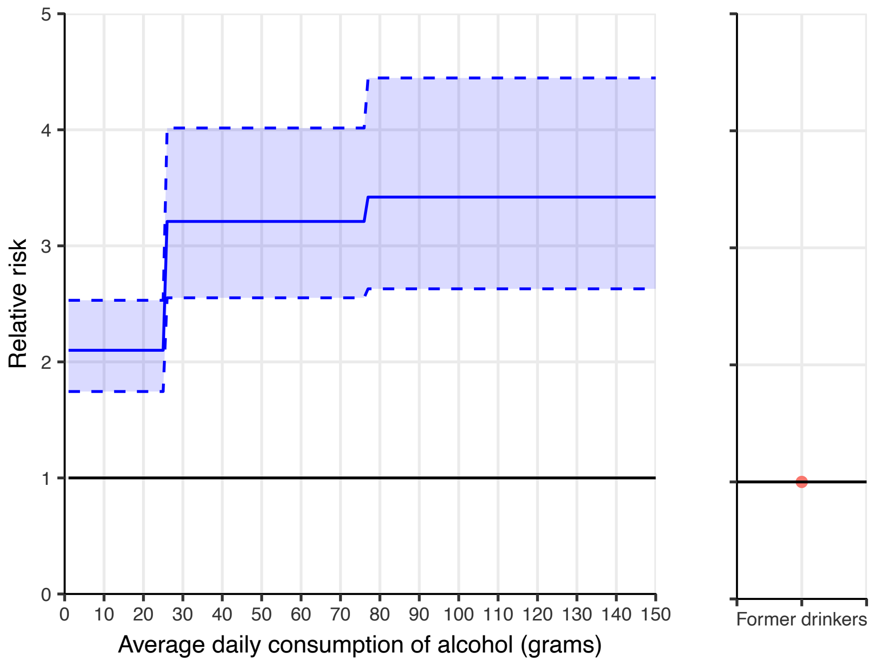

Figure S49. Relative risk for lower respiratory infections among females for Russia

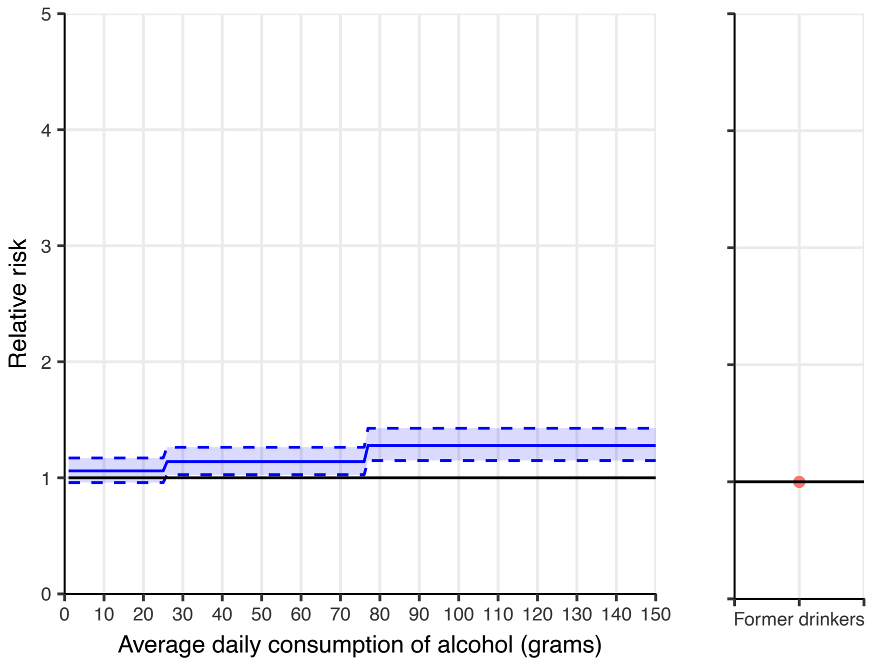

Figure S50. Relative risk for ischaemic stroke among males for Russia

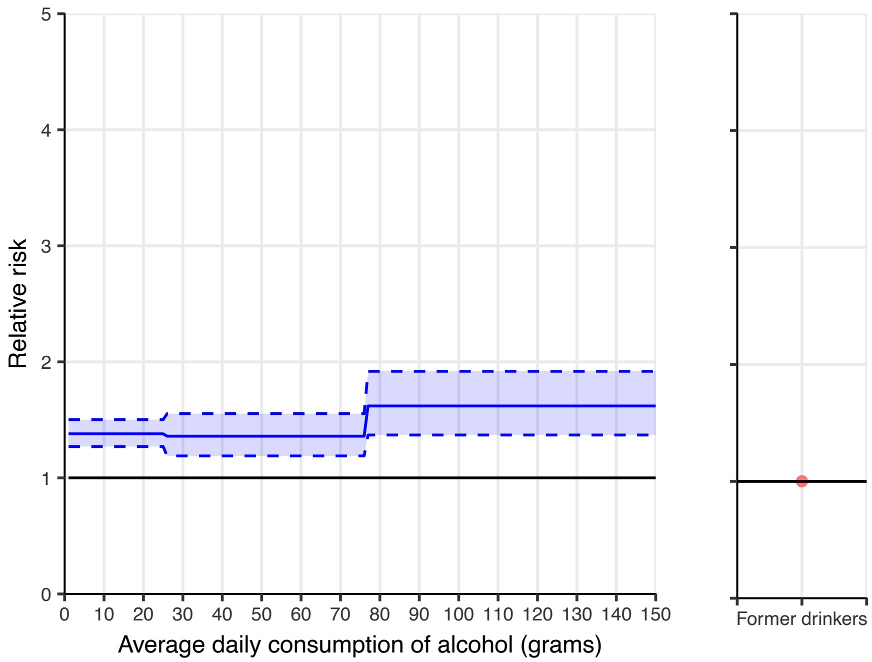

Figure S51. Relative risk for ischaemic stroke among females for Russia

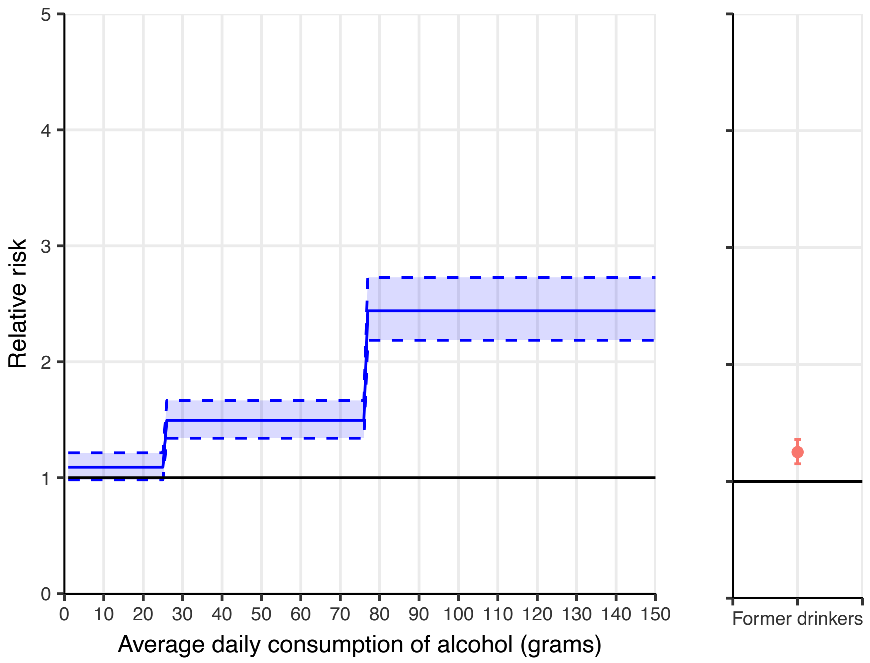

Figure S52. Relative risk for ischaemic heart disease among males for Russia

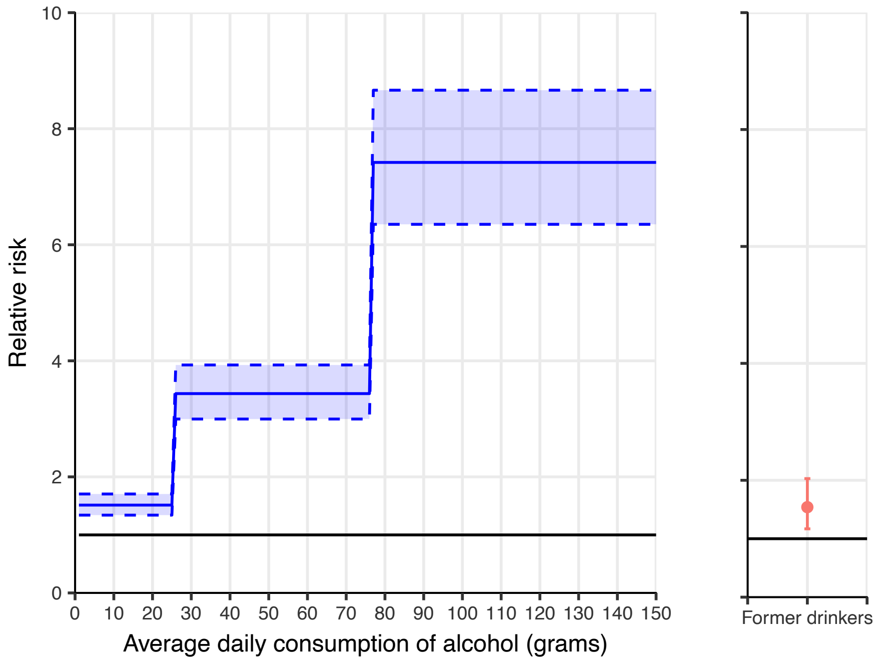

Figure S53. Relative risk for ischaemic heart disease among females for Russia

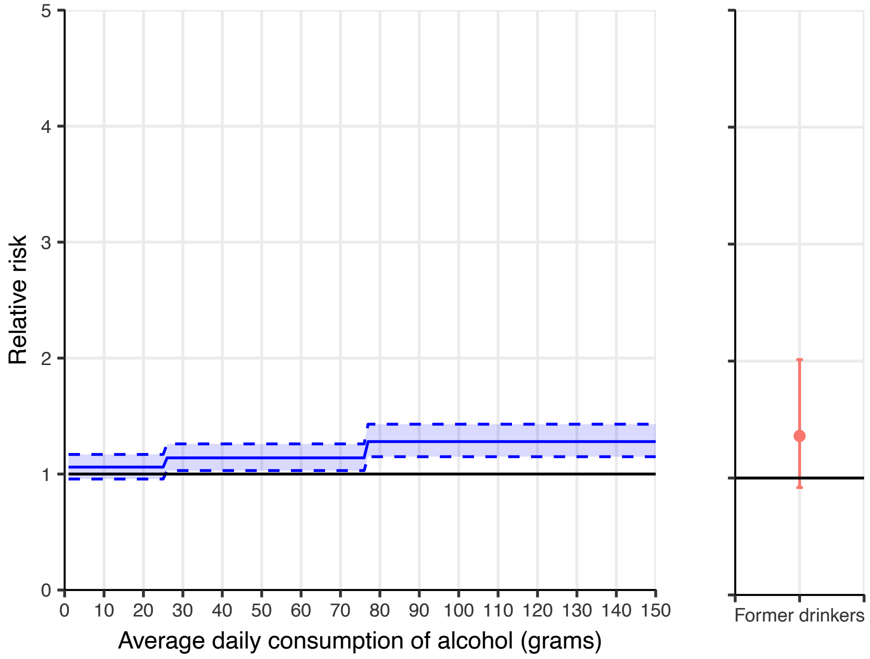

Figure S54. Relative risk for haemorrhagic stroke among males for Russia

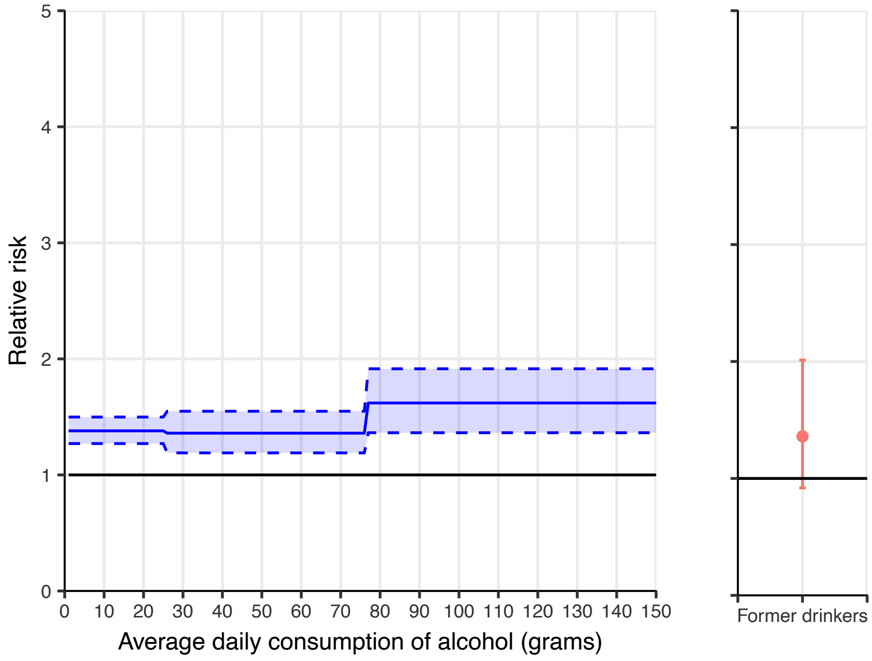

Figure S55. Relative risk for haemorrhagic stroke among females for Russia

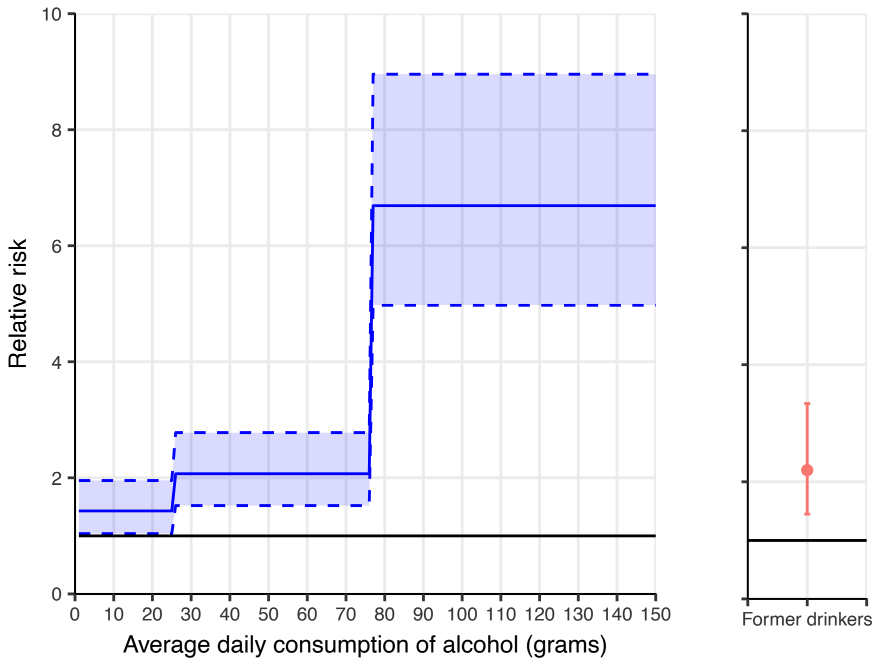

Figure S56. Relative risk for pancreatitis among males for Russia

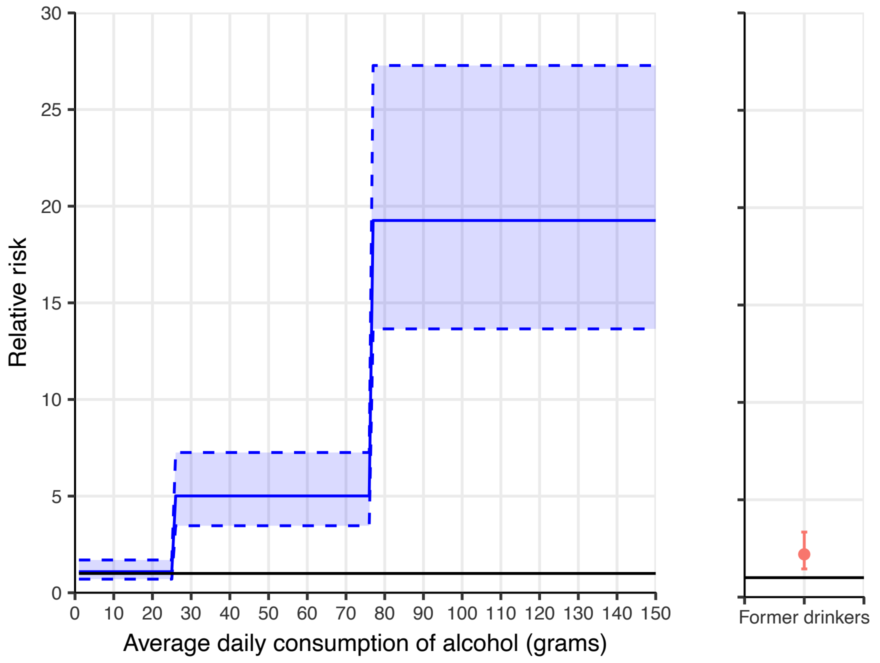

Figure S57. Relative risk for pancreatitis among females for Russia

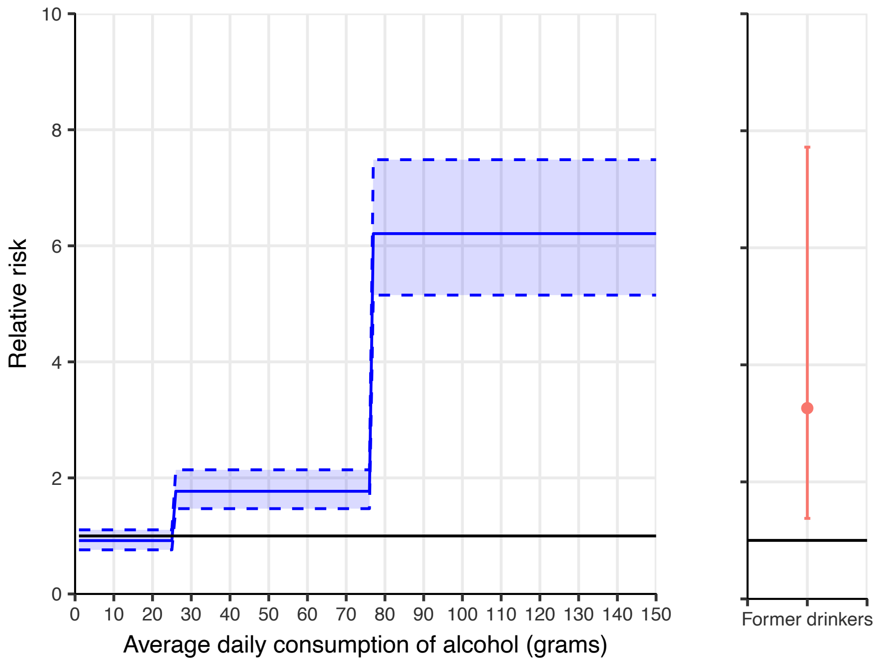

Figure S58. Relative risk for cirrhosis of the liver among males for Russia

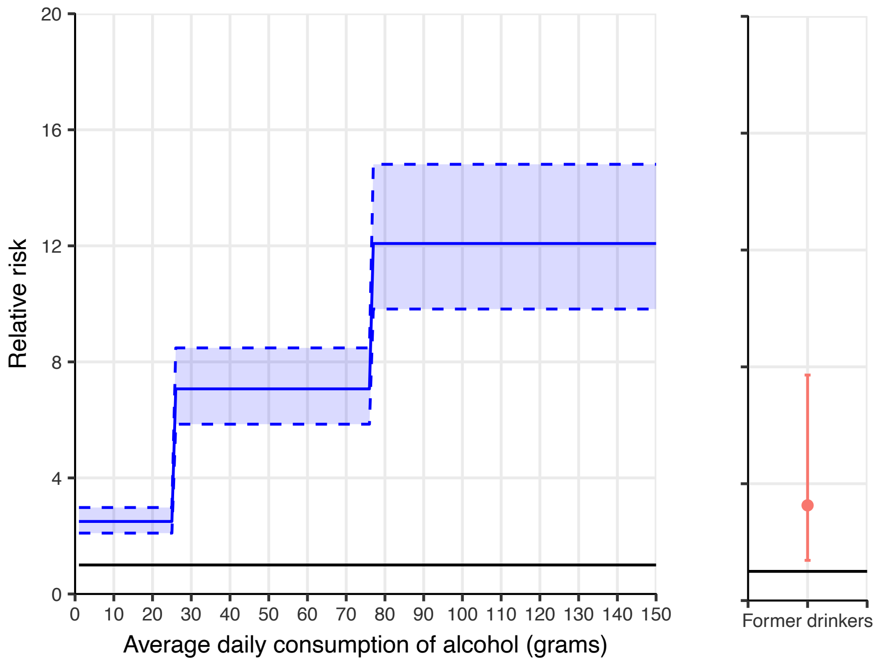

Figure S59. Relative risk for cirrhosis of the liver among females for Russia

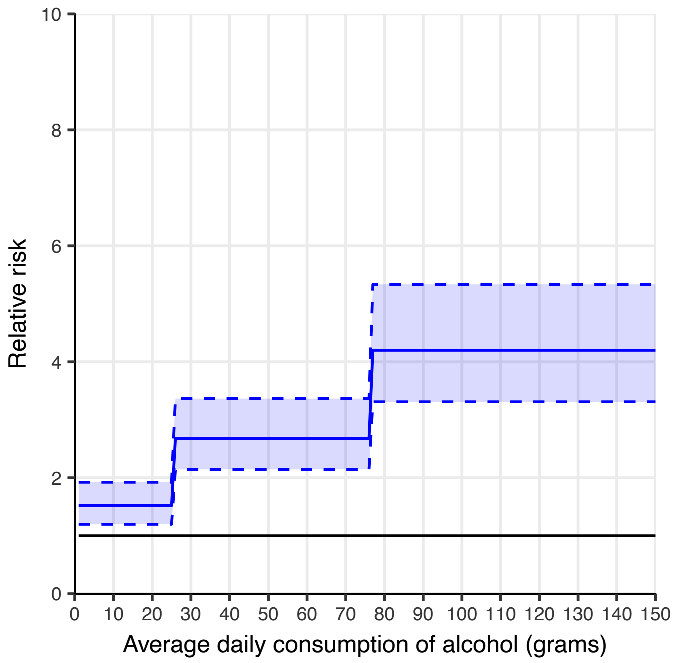

Figure S60. Relative risk for road injury among males for Russia

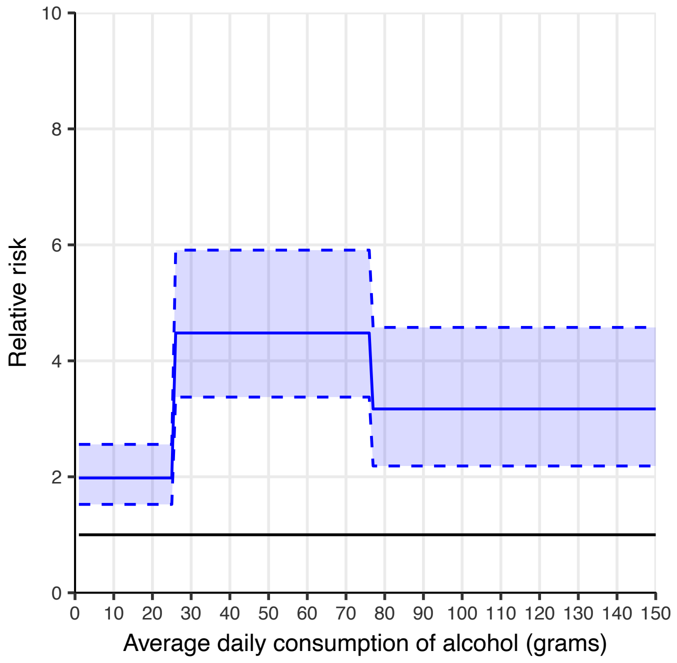

Figure S61. Relative risk for road injury among females for Russia

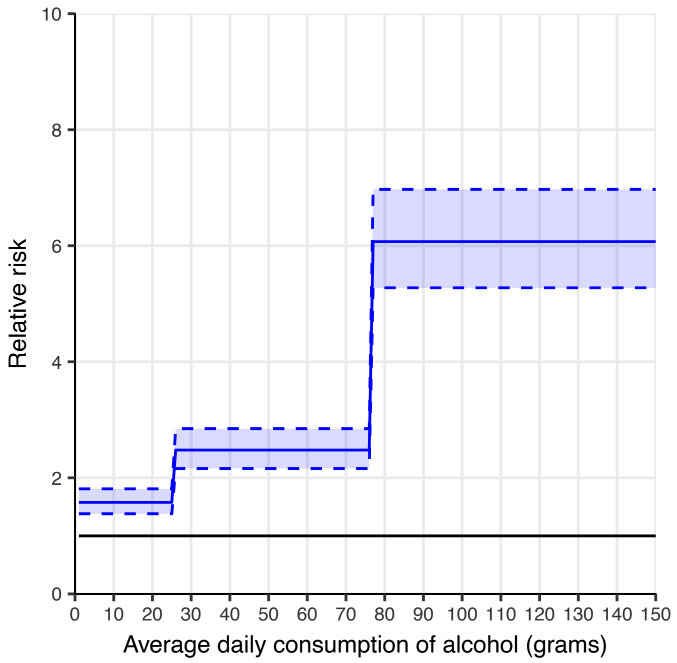

Figure S62. Relative risk for other unintentional injuries among males for Russia

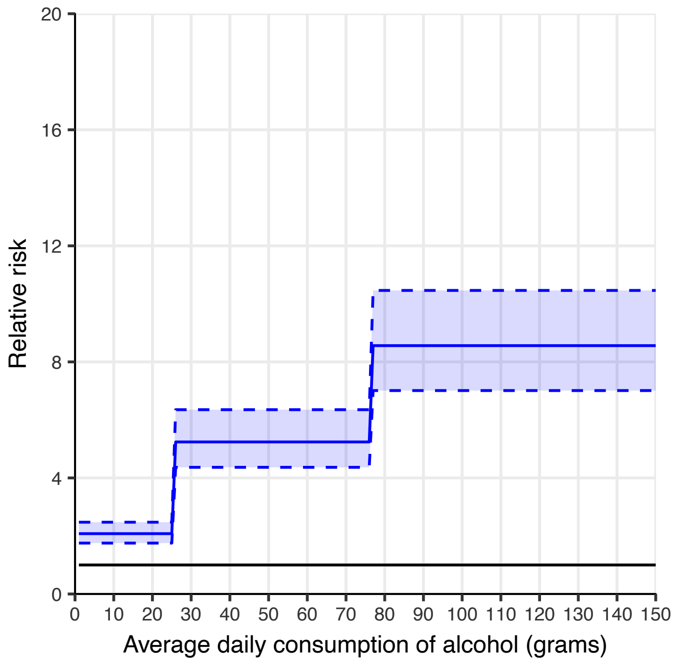

Figure S63. Relative risk for other unintentional injuries among females for Russia

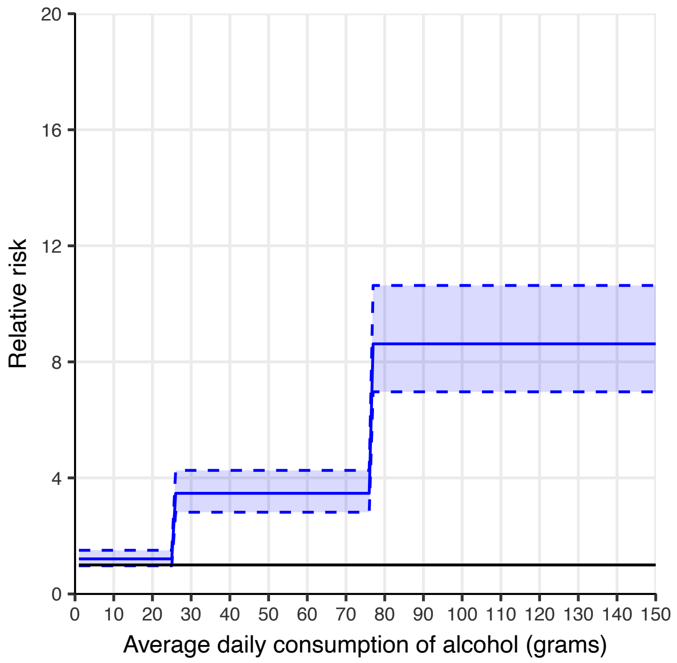

Figure S64. Relative risk for suicide among males

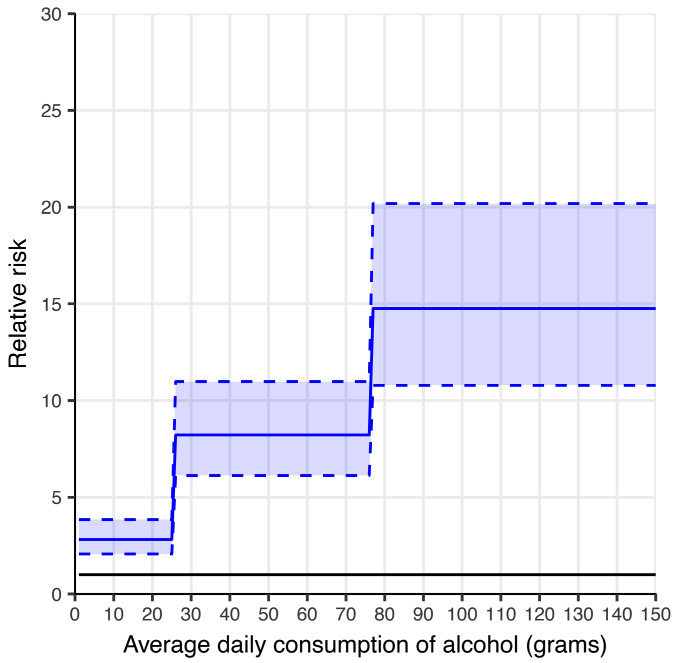

Figure S65. Relative risk for suicide among females

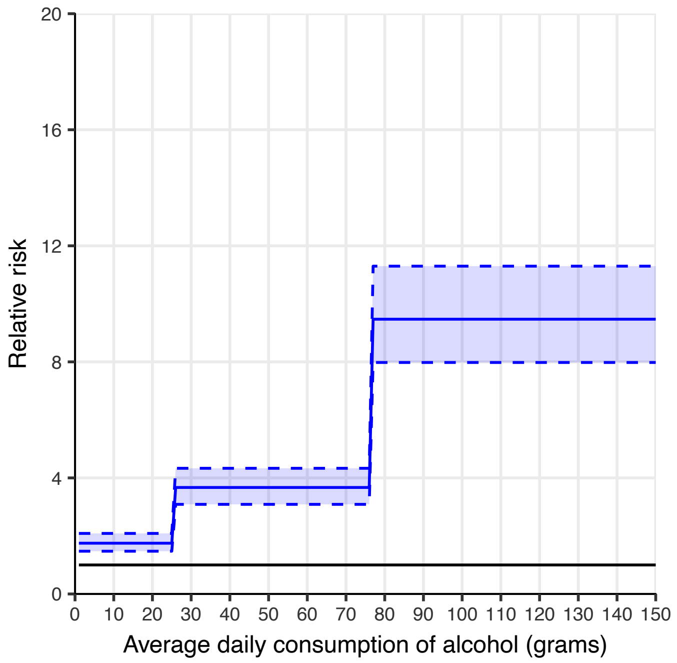

Figure S66. Relative risk for assault among males

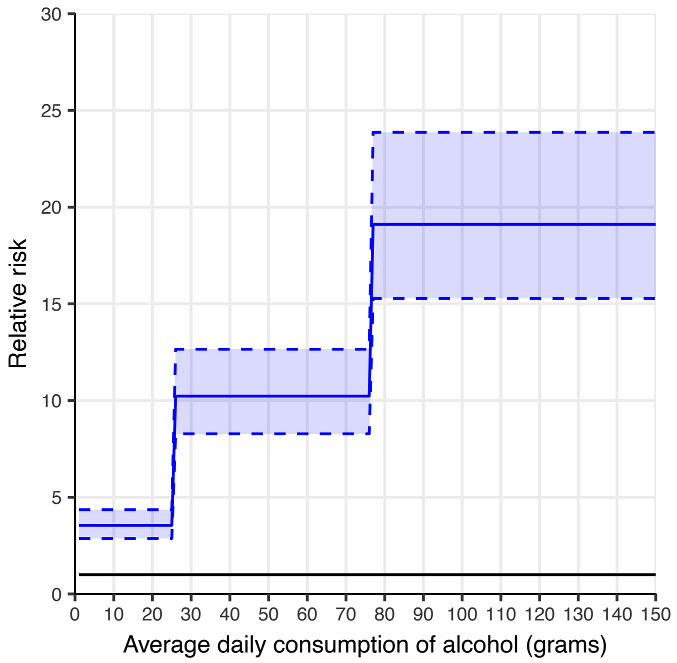

Figure S67. Relative risk for assault among females

Table S7. List of countries by global burden of disease region

Region	Countries
Central Sub-Saharan Africa	Angola, Central African Republic, Congo, Democratic Republic of the Congo, Equatorial Guinea, Gabon
Eastern Sub-Saharan Africa	Burundi, Comoros, Djibouti, Eritrea, Ethiopia, Kenya, Madagascar, Mozambique, Malawi, Rwanda, Somalia, Uganda, United Republic of Tanzania, Zambia
North Africa and Middle East	Afghanistan, Bahrain, Algeria, Egypt, Iran (Islamic Republic of), Iraq, Jordan, Kuwait, Lebanon, Libya, Morocco, Oman, Qatar, Saudi Arabia, Sudan, Syrian Arab Republic, Tunisia, Turkiye, United Arab Emirates, Yemen
Southern Sub-Saharan Africa	Botswana, Eswatini, Lesotho, Namibia, South Africa, Zimbabwe
Western Sub-Saharan Africa	Benin, Burkina Faso, Cote d'Ivoire, Cameroon, Cabo Verde, Ghana, Guinea, Gambia, Guinea Bissau, Liberia, Mali, Mauritania, Niger, Nigeria, Senegal, Sierra Leone, Sao Tome and Principe, Chad, Togo
Andean Latin America	Bolivia, Ecuador, Peru
Central Latin America	Colombia, Costa Rica, El Salvador, Guatemala, Honduras, Mexico, Nicaragua, Panama, Venezuela (Bolivarian Republic of)
Tropical Latin America	Brazil, Paraguay
Southern Latin America	Argentina, Chile, Uruguay
High-income North America	Canada, United States of America
Central Asia	Armenia, Azerbaijan, Georgia, Kazakhstan, Kyrgyzstan, Mongolia, Tajikistan, Turkmenistan, Uzbekistan
East Asia	China, Democratic People's Republic of Korea
High-income Asia Pacific	Brunei Darussalam, Japan, Republic of Korea, Singapore
South Asia	Bangladesh, Bhutan, India, Nepal, Pakistan
Southeast Asia	Cambodia, Indonesia, Lao People's Democratic Republic, Maldives, Myanmar, Mauritius, Malaysia, Philippines, Seychelles, Sri Lanka, Thailand, Timor Leste, Viet Nam
Australasia	Australia, New Zealand
Caribbean	Antigua and Barbuda, Bahamas, Belize, Barbados, Cuba, Dominican Republic, Grenada, Guyana, Haiti, Jamaica, Saint Lucia, Suriname, Trinidad and Tobago, Saint Vincent and the Grenadines
Central Europe	Albania, Bulgaria, Bosnia and Herzegovina, Czechia, Croatia, Hungary, Montenegro, North Macedonia, Poland, Romania, Serbia, Slovakia, Slovenia
Eastern Europe	Belarus, Estonia, Lithuania, Latvia, Republic of Moldova, Russian Federation, Ukraine
Western Europe	Austria, Belgium, Cyprus, Germany, Denmark, Spain, Finland, France, Greece, Ireland, Iceland, Israel, Italy, Luxembourg, Malta, Netherlands, Norway, Portugal, Sweden, Switzerland, United Kingdom of Great Britain and Northern Ireland

Oceania	Fiji, Kiribati, Micronesia (Federated States of), Papua New Guinea, Tonga, Samoa, Solomon Islands, Vanuatu

Table S8. List of countries by human development index grouping

HDI region	Countries
Low	Afghanistan, Benin, Burkina Faso, Burundi, Central African Republic, Chad, Democratic Republic of the Congo, Djibouti, Eritrea, Ethiopia, Gambia, Guinea, Guinea Bissau, Haiti, Lesotho, Liberia, Madagascar, Malawi, Mali, Mozambique, Niger, Nigeria, Pakistan, Rwanda, Senegal, Sierra Leone, Sudan, Togo, Uganda, United Republic of Tanzania, Yemen
Medium	Angola, Bangladesh, Bhutan, Cabo Verde, Cambodia, Cameroon, Comoros, Congo, Cote d'Ivoire, El Salvador, Equatorial Guinea, Eswatini, Ghana, Guatemala, Honduras, India, Iraq, Kenya, Kiribati, Kyrgyzstan, Lao People's Democratic Republic, Mauritania, Micronesia (Federated States of), Morocco, Myanmar, Namibia, Nepal, Nicaragua, Papua New Guinea, Sao Tome and Principe, Solomon Islands, Syrian Arab Republic, Tajikistan, Timor Leste, Vanuatu, Zambia, Zimbabwe
High	Algeria, Armenia, Azerbaijan, Barbados, Belize, Bolivia, Bosnia and Herzegovina, Botswana, Brazil, China, Colombia, Cuba, Dominican Republic, Ecuador, Egypt, Fiji, Gabon, Guyana, Indonesia, Iran (Islamic Republic of), Jamaica, Jordan, Lebanon, Libya, Maldives, Mexico, Mongolia, North Macedonia, Paraguay, Peru, Philippines, Republic of Moldova, Saint Lucia, Saint Vincent and the Grenadines, Samoa, South Africa, Sri Lanka, Suriname, Tonga, Tunisia, Turkmenistan, Ukraine, Uzbekistan, Venezuela (Bolivarian Republic of), Viet Nam,
Very high	Albania, Antigua and Barbuda, Argentina, Australia, Austria, Bahamas, Bahrain, Belarus, Belgium, Brunei Darussalam, Bulgaria, Canada, Chile, Costa Rica, Croatia, Cyprus, Czechia, Denmark, Estonia, Finland, France, Georgia, Germany, Greece, Grenada, Hungary, Iceland, Ireland, Israel, Italy, Japan, Kazakhstan, Kuwait, Latvia, Lithuania, Luxembourg, Malaysia, Malta, Mauritius, Montenegro, Netherlands, New Zealand, Norway, Oman, Panama, Poland, Portugal, Qatar, Republic of Korea, Romania, Russian Federation, Saudi Arabia, Serbia, Seychelles, Singapore, Slovakia, Slovenia, Spain, Sweden, Switzerland, Thailand, Trinidad and Tobago, Turkiye, United Arab Emirates, United Kingdom of Great Britain and Northern Ireland, United States of America, Uruguay

Supplemental Results

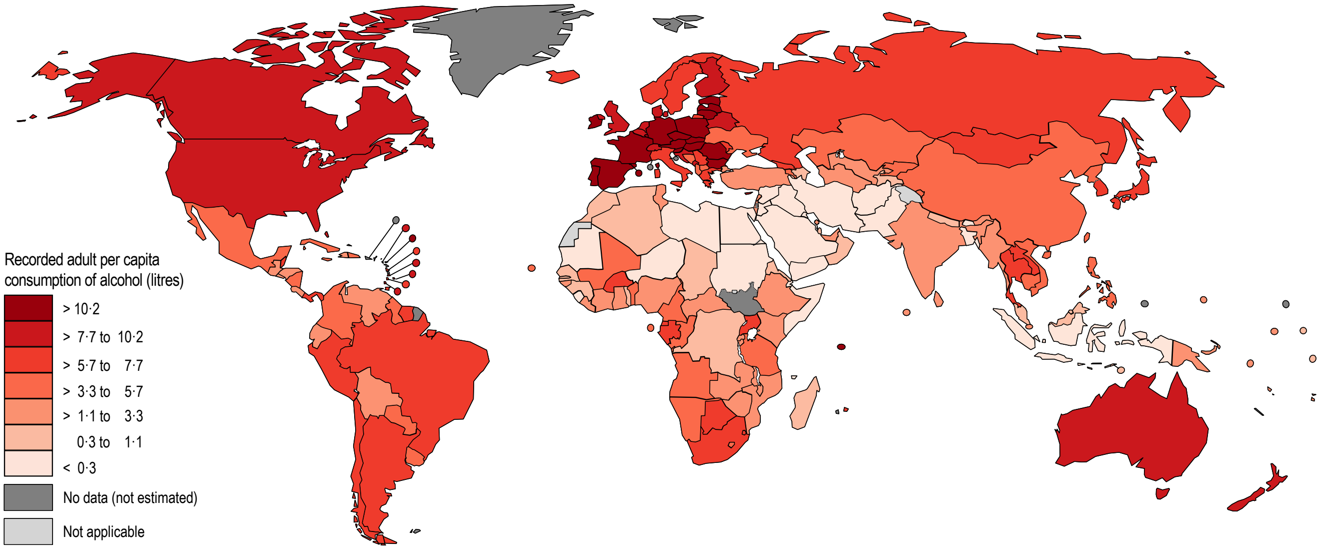

Figure S68. Recorded adult per capita consumption of alcohol in 2019

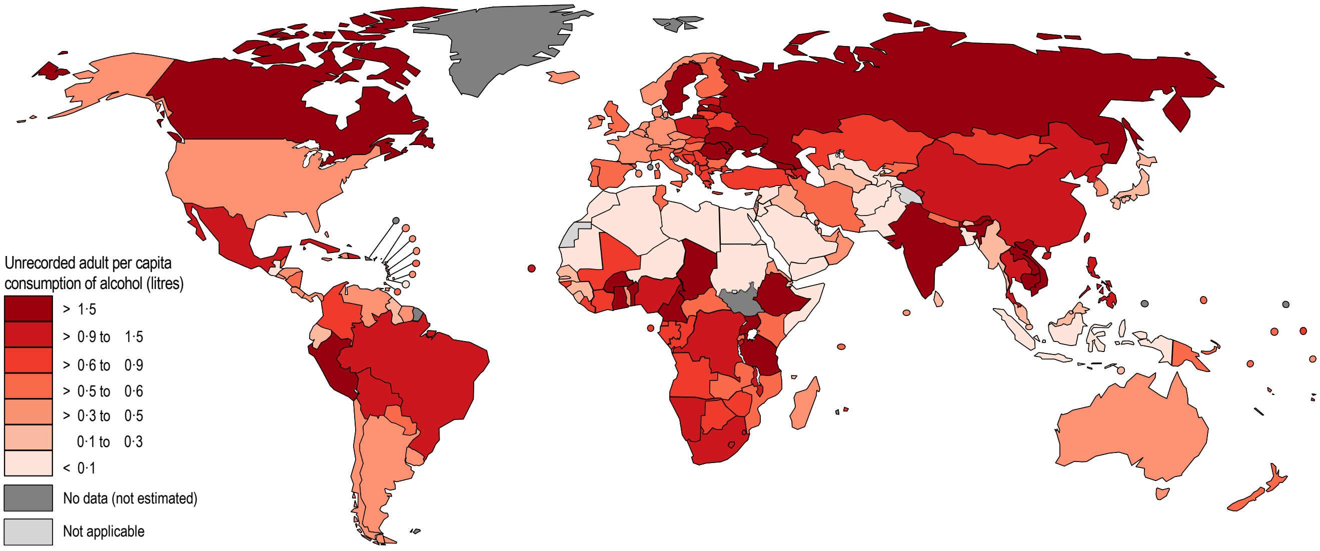

Figure S69. Unrecorded adult per capita consumption of alcohol in 2019

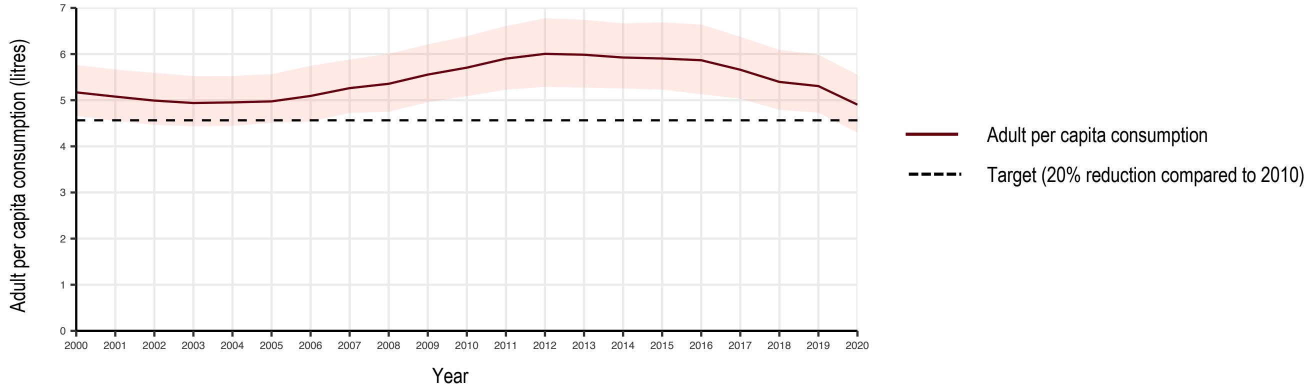

Figure S70. Change in *per capita* consumption of alcohol among adults from 2000 to 2020

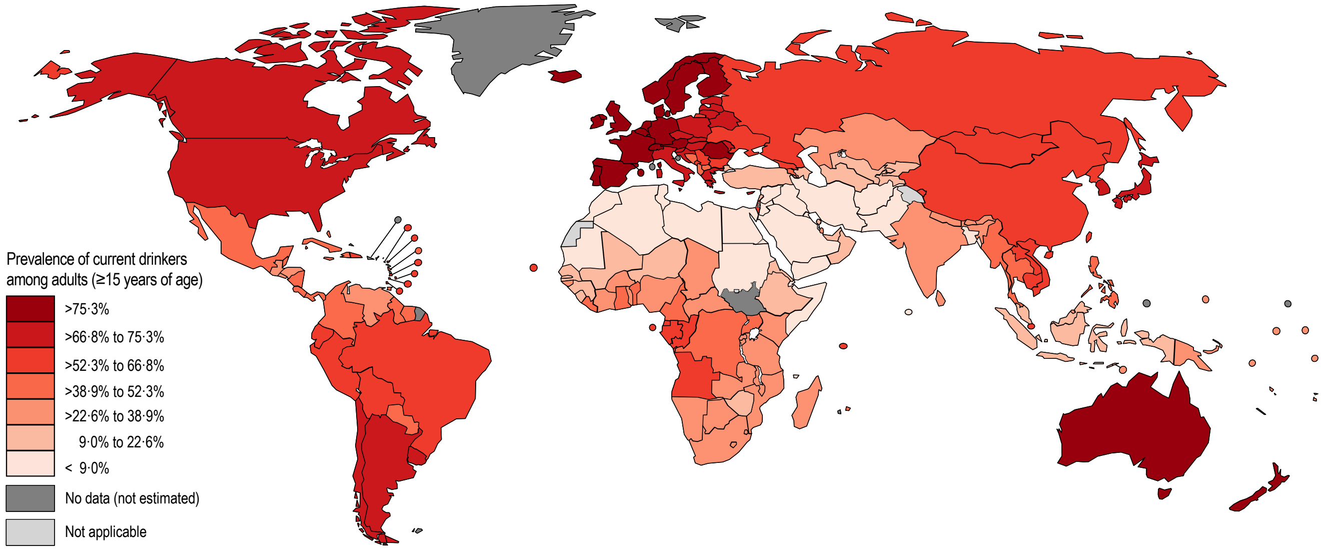

Figure S71. Prevalence of current drinkers among adults in 2019 by country

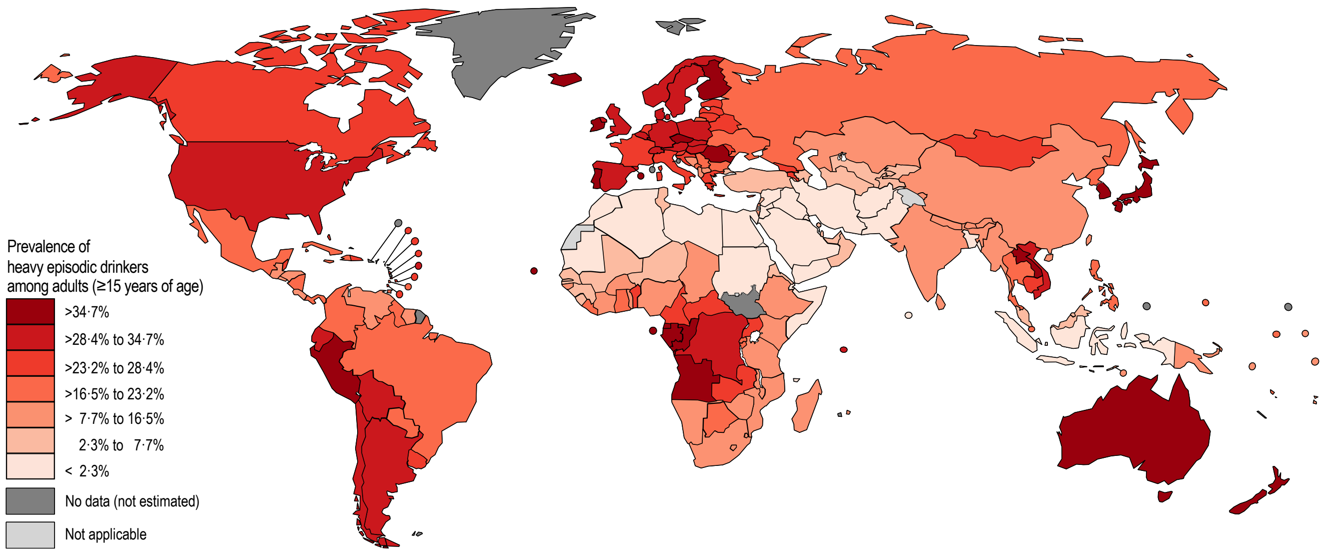

Figure S72. Prevalence of heavy episodic drinking in 2019, by country

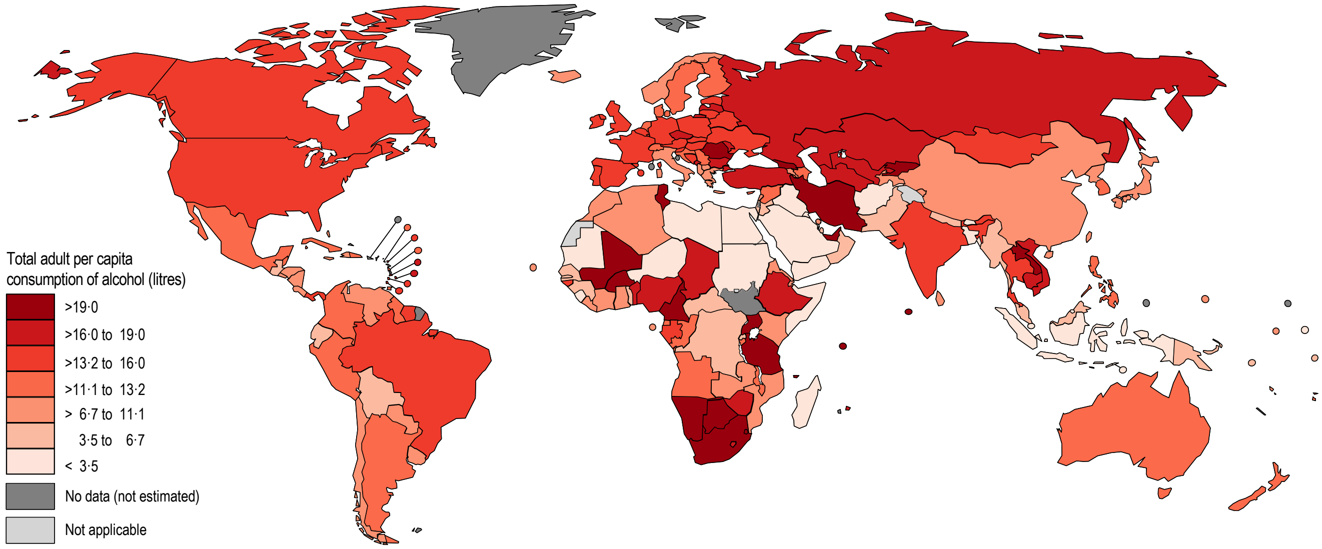

Figure S73. Adult per capita consumption of alcohol in 2019 among drinkers by country

Table S9. Alcohol consumption in 2019, by region

Region	APC*			Current drinkers (%)	Heavy episodic drinkers (age-standardised) (%)
	Total	Recorded	Unrecorded		
Global	5·5 (4·9, 6·2)	4·3 (3·8, 4·8)	1·2 (0·8, 1·6)	43·8 (42·8, 44·8)	16·7 (14·5, 18·9)
Global Burden of Disease Region					
Central Asia	3·7 (3·0, 4·5)	3·0 (2·4, 3·7)	0·7 (0·4, 1·0)	21·4 (20·3, 22·5)	7·9 (7·6, 8·1)
Central Europe	11·7 (10·3, 13·2)	10·2 (9·1, 11·4)	1·9 (1·1, 2·7)	70·7 (70·1, 71·3)	34·7 (34·0, 35·5)
Eastern Europe	10·1 (7·7, 12·5)	7·0 (5·3, 8·7)	3·1 (1·3, 4·8)	61·2 (59·7, 62·6)	21·1 (19·3, 22·9)
Australasia	10·1 (7·6, 12·5)	9·4 (7·2, 11·8)	0·5 (0·0, 1·2)	80·4 (79·4, 81·5)	39·5 (37·0, 42·1)
High-income Asia Pacific	6·9 (5·2, 8·8)	6·6 (5·0, 8·3)	0·3 (0·0, 0·8)	69·0 (67·9, 70·2)	43·2 (42·1, 44·4)
High-income North America	9·6 (7·1, 12·2)	8·8 (6·5, 11·2)	0·6 (0·1, 1·4)	70·9 (69·7, 72·2)	32·7 (31·2, 34·3)
Southern Latin America	7·5 (5·8, 9·4)	7·1 (5·5, 8·9)	0·4 (0·0, 1·0)	70·6 (69·1, 72·1)	30·0 (28·1, 32·0)
Western Europe	10·3 (9·3, 11·4)	9·7 (8·8, 10·7)	0·5 (0·3, 0·8)	77·4 (77·0, 77·8)	33·6 (33·0, 34·2)
Andean Latin America	5·7 (4·1, 7·3)	4·4 (3·2, 5·7)	1·2 (0·3, 2·3)	64·5 (63·5, 65·4)	34·0 (32·0, 36·0)
Caribbean	5·4 (4·4, 6·6)	4·7 (3·8, 5·6)	0·9 (0·3, 1·6)	50·1 (49·1, 51·2)	23·4 (22·7, 24·1)
Central Latin America	4·8 (3·4, 6·1)	4·1 (3·0, 5·1)	0·8 (0·2, 1·6)	41·8 (40·5, 43·2)	17·2 (16·4, 18·0)
Tropical Latin America	7·6 (5·1, 10·1)	6·3 (4·2, 8·3)	1·3 (0·0, 2·9)	57·9 (54·1, 61·7)	20·8 (18·7, 22·8)
North Africa and Middle East	0·6 (0·4, 1·0)	0·4 (0·3, 0·6)	0·2 (0·1, 0·5)	6·2 (5·9, 6·4)	1·3 (1·2, 1·4)
South Asia	3·9 (2·1, 5·8)	2·4 (1·2, 3·6)	1·5 (0·0, 3·1)	25·5 (23·4, 27·6)	9·9 (9·0, 10·8)
East Asia	5·7 (3·5, 7·9)	4·8 (3·0, 6·6)	0·9 (0·0, 2·2)	61·2 (56·9, 65·4)	14·8 (4·2, 25·4)
Oceania	1·8 (0·9, 3·0)	1·4 (0·6, 2·1)	0·5 (0·1, 1·3)	27·9 (26·4, 29·5)	15·5 (14·9, 16·1)
Southeast Asia	3·9 (3·2, 4·7)	2·5 (2·0, 3·0)	1·3 (0·8, 2·0)	31·9 (31·0, 32·9)	13·2 (12·8, 13·6)
Central Sub-Saharan Africa	3·4 (2·3, 4·6)	2·5 (1·7, 3·2)	1·0 (0·2, 1·9)	46·5 (44·9, 48·0)	36·7 (35·4, 38·1)
Eastern Sub-Saharan Africa	4·7 (3·8, 5·6)	2·7 (2·2, 3·2)	2·0 (1·3, 2·8)	27·1 (26·2, 27·9)	12·4 (12·2, 12·7)
Southern Sub-Saharan Africa	7·7 (5·6, 10·0)	6·5 (4·7, 8·3)	1·3 (0·2, 2·5)	30·3 (28·6, 32·0)	12·9 (12·2, 13·7)
Western Sub-Saharan Africa	4·2 (3·2, 5·3)	2·6 (1·9, 3·4)	1·6 (0·9, 2·4)	27·2 (26·3, 28·2)	12·8 (12·4, 13·2)
Human Development Index grouping					
Very high	2·9 (2·5, 3·5)	1·8 (1·5, 2·1)	1·2 (0·8, 1·6)	19·8 (19·3, 20·3)	9·9 (9·7, 10·1)
High	4·1 (2·5, 5·8)	2·6 (1·5, 3·7)	1·6 (0·3, 3·0)	28·9 (27·0, 30·7)	11·8 (11·1, 12·6)
Medium	5·1 (3·9, 6·3)	4·0 (3·0, 5·0)	1·0 (0·5, 1·8)	48·2 (46·0, 50·5)	14·2 (8·6, 19·8)
Low	8·7 (8·0, 9·5)	7·8 (7·1, 8·5)	0·9 (0·6, 1·2)	64·0 (63·6, 64·4)	29·4 (28·9, 29·9)

* Three-year averages

Table S10. Alcohol consumption by sex in 2019

Region	Current drinkers (%)			Heavy episodic drinkers (age-standardised) (%)		
	Total	Male	Female	Total	Male	Female
Global	43·8 (42·8, 44·8)	52·2 (51·3, 53·2)	35·4 (34·3, 36·5)	16·7 (14·5, 18·9)	23·5 (20·4, 26·6)	9·7 (8·4, 10·9)
Global Burden of Disease Region						
Central Asia	21·4 (20·3, 22·5)	28·4 (27·0, 29·9)	14·9 (14·1, 15·6)	7·9 (7·6, 8·1)	12·0 (11·6, 12·3)	4·2 (4·0, 4·3)
Central Europe	70·7 (70·1, 71·3)	79·4 (78·9, 79·9)	62·7 (61·9, 63·4)	34·7 (34·0, 35·5)	46·3 (45·4, 47·1)	23·1 (22·4, 23·8)
Eastern Europe	61·2 (59·7, 62·6)	73·2 (71·9, 74·5)	51·2 (49·6, 52·7)	21·1 (19·3, 22·9)	31·8 (29·2, 34·4)	10·7 (9·7, 11·8)
Australasia	80·4 (79·4, 81·5)	86·4 (85·6, 87·2)	74·7 (73·4, 76·0)	39·5 (37·0, 42·1)	52·1 (49·3, 54·9)	27·0 (24·7, 29·3)
High-income Asia Pacific	69·0 (67·9, 70·2)	77·6 (76·7, 78·6)	60·8 (59·5, 62·2)	43·2 (42·1, 44·4)	55·5 (54·3, 56·6)	30·0 (28·8, 31·2)
High-income North America	70·9 (69·7, 72·2)	78·5 (77·5, 79·6)	63·6 (62·2, 65·0)	32·7 (31·2, 34·3)	44·3 (42·6, 46·1)	20·7 (19·4, 22·1)
Southern Latin America	70·6 (69·1, 72·1)	77·8 (76·6, 79·1)	63·6 (61·9, 65·3)	30·0 (28·1, 32·0)	40·9 (38·6, 43·3)	18·4 (16·8, 20·0)
Western Europe	77·4 (77·0, 77·8)	84·8 (84·5, 85·1)	70·3 (69·9, 70·8)	33·6 (33·0, 34·2)	45·4 (44·7, 46·2)	21·7 (21·2, 22·2)
Andean Latin America	64·5 (63·5, 65·4)	71·8 (70·9, 72·6)	57·4 (56·4, 58·3)	34·0 (32·0, 36·0)	44·9 (42·7, 47·1)	22·2 (20·4, 24·0)
Caribbean	50·1 (49·1, 51·2)	60·3 (59·3, 61·3)	40·2 (39·1, 41·3)	23·4 (22·7, 24·1)	32·9 (32·0, 33·9)	13·9 (13·3, 14·5)
Central Latin America	41·8 (40·5, 43·2)	51·4 (50·1, 52·8)	32·7 (31·4, 34·0)	17·2 (16·4, 18·0)	24·8 (23·8, 25·8)	9·8 (9·2, 10·3)
Tropical Latin America	57·9 (54·1, 61·7)	66·9 (63·3, 70·5)	49·4 (45·4, 53·4)	20·8 (18·7, 22·8)	29·6 (26·9, 32·3)	11·7 (10·3, 13·2)
North Africa and Middle East	6·2 (5·9, 6·4)	8·9 (8·5, 9·2)	3·3 (3·1, 3·5)	1·3 (1·2, 1·4)	2·2 (2·1, 2·3)	0·5 (0·4, 0·5)
South Asia	25·5 (23·4, 27·6)	33·8 (31·3, 36·3)	16·9 (15·2, 18·6)	9·9 (9·0, 10·8)	15·1 (13·9, 16·4)	4·8 (4·3, 5·3)
East Asia	61·2 (56·9, 65·4)	73·3 (69·7, 76·9)	48·8 (43·9, 53·8)	14·8 (4·2, 25·4)	21·5 (6·8, 36·2)	7·2 (1·2, 13·3)
Oceania	27·9 (26·4, 29·5)	36·2 (34·4, 37·9)	19·2 (17·9, 20·6)	15·5 (14·9, 16·1)	22·2 (21·4, 23·1)	8·6 (8·1, 9·0)
Southeast Asia	31·9 (31·0, 32·9)	39·8 (38·6, 41·0)	24·2 (23·5, 25·0)	13·2 (12·8, 13·6)	18·8 (18·3, 19·2)	7·6 (7·4, 7·9)
Central Sub-Saharan Africa	46·5 (44·9, 48·0)	56·4 (54·7, 58·0)	36·8 (35·3, 38·4)	36·7 (35·4, 38·1)	48·4 (47·0, 49·8)	25·8 (24·5, 27·0)
Eastern Sub-Saharan Africa	27·1 (26·2, 27·9)	35·2 (34·2, 36·3)	19·2 (18·4, 19·9)	12·4 (12·2, 12·7)	19·0 (18·6, 19·4)	6·6 (6·4, 6·8)
Southern Sub-Saharan Africa	30·3 (28·6, 32·0)	40·7 (38·8, 42·7)	21·0 (19·4, 22·5)	12·9 (12·2, 13·7)	20·4 (19·3, 21·6)	6·6 (6·1, 7·1)
Western Sub-Saharan Africa	27·2 (26·3, 28·2)	35·5 (34·3, 36·6)	19·0 (18·3, 19·8)	12·8 (12·4, 13·2)	19·4 (18·8, 19·9)	6·7 (6·4, 6·9)
Human Development Index grouping						
Very high	19·8 (19·3, 20·3)	71·3 (70·9, 71·6)	57·0 (56·5, 57·4)	9·9 (9·7, 10·1)	39·5 (39·0, 40·1)	18·9 (18·5, 19·3)
High	28·9 (27·0, 30·7)	58·1 (56·2, 60·1)	38·4 (35·8, 41·0)	11·8 (11·1, 12·6)	20·4 (12·5, 28·3)	7·6 (4·4, 10·7)
Medium	48·2 (46·0, 50·5)	25·7 (25·1, 26·3)	14·0 (13·6, 14·4)	14·2 (8·6, 19·8)	14·7 (14·4, 15·0)	5·5 (5·4, 5·7)
Low	64·0 (63·6, 64·4)	37·8 (35·6, 40·0)	19·7 (18·2, 21·2)	29·4 (28·9, 29·9)	17·7 (16·6, 18·8)	6·1 (5·6, 6·5)

Table S11. Alcohol-attributable burden of disease in 2019, by cause for males

Cause	Alcohol-attributable burden*		Age-adjusted alcohol-attributable burden per 100 000 people*		Population-attributable fraction (%)*	
	Deaths (1000s)	DALYs lost (100 000s)	Deaths	DALYs lost	Deaths	DALYs lost
All Causes	2 018·4 (1 679·8, 2 388·9)	927·2 (796·3, 1 080·3)	52·2 (43·3, 62·0)	2 381·3 (2 044·3, 2 775·7)	6·7 (5·6, 7·9)	6·9 (5·9, 8·0)
Communicable, maternal, perinatal and nutritional conditions	245·1 (94·8, 402·9)	102·1 (33·9, 176·3)	6·4 (2·5, 10·4)	263·5 (87·7, 455·0)	4·6 (1·8, 7·6)	2·8 (0·9, 4·9)
Tuberculosis	168·7 (29·7, 326·1)	80·3 (14·1, 155·1)	4·3 (0·8, 8·4)	207·0 (36·3, 399·8)	22·7 (4·0, 43·9)	20·6 (3·6, 39·8)
STDs excluding HIV	0·0 (0·0, 0·1)	0·2 (0·1, 0·2)	0·0 (0·0, 0·0)	0·4 (0·2, 0·6)	0·2 (0·1, 0·2)	0·6 (0·3, 0·8)
HIV/AIDS	13·4 (8·2, 18·6)	7·1 (4·4, 9·9)	0·3 (0·2, 0·5)	18·4 (11·2, 25·6)	3·5 (2·2, 4·9)	3·3 (2·0, 4·6)
Lower respiratory infections	63·0 (17·5, 107·6)	14·5 (4·8, 23·9)	1·7 (0·5, 2·9)	37·7 (12·3, 62·4)	4·6 (1·3, 7·9)	2·5 (0·8, 4·1)
Noncommunicable diseases	1 169·1 (932·8, 1 382·4)	472·3 (421·4, 516·3)	30·1 (23·9, 35·9)	1 206·3 (1 073·9, 1 321·1)	5·4 (4·3, 6·4)	5·8 (5·2, 6·3)
Malignant neoplasms	324·1 (280·3, 365·6)	85·6 (73·9, 96·6)	8·4 (7·3, 9·5)	217·4 (187·6, 245·6)	6·2 (5·3, 7·0)	6·4 (5·5, 7·2)
Lip and oral cavity	46·6 (37·3, 54·1)	13·8 (10·9, 16·1)	1·2 (1·0, 1·4)	34·8 (27·6, 40·6)	36·8 (29·5, 42·7)	36·0 (28·5, 41·9)
Other pharynx	39·4 (31·2, 45·7)	11·5 (9·1, 13·4)	1·0 (0·8, 1·2)	29·0 (22·8, 33·7)	41·8 (33·0, 48·5)	42·1 (33·1, 48·9)
Oesophagus cancer	76·8 (58·8, 93·4)	19·8 (15·2, 23·8)	2·0 (1·5, 2·4)	50·1 (38·6, 60·3)	22·6 (17·3, 27·5)	23·0 (17·7, 27·7)
Colon and rectum cancers	91·8 (75·7, 109·6)	21·7 (17·6, 26·3)	2·4 (2·0, 2·9)	55·8 (45·4, 67·6)	18·1 (14·9, 21·6)	17·5 (14·2, 21·2)
Liver cancer	46·8 (28·1, 71·2)	12·5 (7·6, 19·1)	1·2 (0·7, 1·8)	31·9 (19·3, 48·6)	11·8 (7·1, 17·9)	11·1 (6·7, 16·9)
Breast cancer						
Cervix uteri cancer						
Larynx cancer	22·6 (16·7, 28·3)	6·3 (4·6, 7·8)	0·6 (0·4, 0·7)	15·8 (11·6, 19·7)	23·6 (17·3, 29·5)	24·0 (17·6, 29·8)
Diabetes mellitus	17·7 (0·8, 36·2)	9·5 (0·8, 19·1)	0·5 (0·0, 1·0)	24·3 (1·9, 49·1)	2·4 (0·1, 5·0)	2·6 (0·2, 5·3)
Alcohol use disorders	133·9 (133·9, 133·9)	153·2 (153·2, 153·2)	3·4 (3·4, 3·4)	393·3 (393·3, 393·3)	100·0 (100·0, 100·0)	100·0 (100·0, 100·0)
Epilepsy	11·2 (8·3, 14·1)	9·8 (7·2, 12·3)	0·3 (0·2, 0·4)	25·4 (18·7, 31·8)	17·1 (12·6, 21·5)	13·6 (10·0, 17·1)
Cardiovascular diseases	242·8 (47·1, 441·8)	62·9 (27·4, 96·6)	6·3 (1·1, 11·8)	160·7 (67·2, 249·8)	2·6 (0·5, 4·7)	2·9 (1·2, 4·4)
Hypertensive heart disease	48·2 (34·2, 61·2)	10·3 (7·4, 12·9)	1·3 (0·9, 1·7)	26·7 (19·1, 33·6)	9·5 (6·7, 12·1)	9·7 (6·9, 12·1)
Ischaemic heart disease	27·5 (154·7, 198·3)	4·6 (27·5, 32·2)	0·7 (4·3, 5·4)	11·7 (72·4, 85·5)	0·6 (3·2, 4·1)	0·4 (2·5, 2·9)
Ischaemic stroke	-23·8 (-66·2, 15·5)	-4·8 (-11·6, 2·6)	0·7 (1·8, 0·4)	12·7 (31·5, 7·0)	1·6 (4·5, 1·0)	1·6 (3·8, 0·9)
Haemorrhagic stroke	166·8 (105·8, 239·6)	44·8 (29·0, 64·4)	4·4 (2·8, 6·3)	114·6 (74·1, 164·8)	9·9 (6·3, 14·2)	10·1 (6·5, 14·5)
Alcoholic cardiomyopathy	24·1 (24·1, 24·1)	8·0 (8·0, 8·0)	0·6 (0·6, 0·6)	20·4 (20·4, 20·4)	100·0 (100·0, 100·0)	100·0 (100·0, 100·0)
Digestive diseases	439·4 (359·2, 506·4)	151·4 (124·1, 172·2)	11·2 (9·2, 13·0)	385·3 (315·7, 438·4)	30·1 (24·6, 34·7)	28·4 (23·3, 32·4)
Cirrhosis of the liver**	415·0 (335·6, 480·3)	142·5 (114·8, 163·6)	10·6 (8·6, 12·3)	362·5 (291·9, 416·2)	47·9 (38·8, 55·5)	48·7 (39·2, 55·9)
Pancreatitis	24·4 (17·1, 32·2)	8·9 (6·3, 11·6)	0·6 (0·4, 0·8)	22·8 (16·0, 29·9)	36·0 (25·2, 47·4)	36·9 (26·0, 48·4)
Injuries	604·1 (448·5, 803·5)	352·8 (263·4, 467·3)	15·7 (11·6, 20·8)	911·5 (680·4, 1 208·1)	20·2 (15·0, 26·8)	20·1 (15·0, 26·7)
Unintentional injuries	427·3 (319·1, 592·4)	262·2 (196·5, 359·5)	11·1 (8·3, 15·4)	676·8 (506·8, 927·7)	20·5 (15·3, 28·4)	20·8 (15·6, 28·5)
Road injury	239·9 (156·9, 370·1)	146·5 (96·0, 223·9)	6·2 (4·1, 9·6)	378·5 (247·9, 578·3)	25·1 (16·4, 38·7)	25·5 (16·7, 38·9)
Poisonings	9·0 (6·2, 12·5)	4·2 (2·8, 5·7)	0·2 (0·2, 0·3)	10·8 (7·2, 14·8)	17·1 (11·7, 23·7)	14·4 (9·6, 19·8)
Falls	62·1 (36·6, 93·9)	43·0 (26·4, 62·0)	1·6 (1·0, 2·5)	110·8 (68·0, 159·9)	16·5 (9·7, 24·9)	19·8 (12·2, 28·6)
Fire, heat and hot substances	9·4 (6·5, 12·8)	6·4 (4·1, 8·8)	0·2 (0·2, 0·3)	16·3 (10·6, 22·6)	16·1 (11·2, 22·0)	15·7 (10·2, 21·8)
Drowning	24·1 (15·0, 34·8)	11·0 (6·8, 15·9)	0·6 (0·4, 0·9)	28·6 (17·5, 41·2)	14·9 (9·3, 21·6)	12·0 (7·4, 17·3)
Exposure to mechanical forces	14·5 (8·6, 21·2)	16·5 (10·1, 23·3)	0·4 (0·2, 0·5)	42·4 (26·0, 60·0)	16·3 (9·7, 23·8)	19·6 (12·0, 27·7)
Other unintentional injuries	68·5 (42·2, 98·0)	34·7 (21·2, 49·3)	1·8 (1·1, 2·6)	89·5 (54·6, 127·2)	17·6 (10·8, 25·1)	15·9 (9·8, 22·6)
Intentional injuries	176·7 (69·1, 280·2)	90·6 (34·2, 144·4)	4·6 (1·8, 7·2)	234·7 (88·3, 374·3)	19·4 (7·6, 30·7)	18·4 (6·9, 29·4)
Self-harm	104·8 (45·8, 161·5)	47·9 (20·4, 74·1)	2·7 (1·2, 4·2)	123·6 (52·7, 191·2)	21·5 (9·4, 33·2)	22·0 (9·4, 34·0)
Interpersonal violence	71·9 (23·9, 118·5)	42·7 (13·9, 70·4)	1·9 (0·6, 3·1)	111·1 (35·9, 183·3)	18·9 (6·3, 31·2)	18·5 (6·0, 30·5)

DALYs: Disability-adjusted life years

* Negative values represent deaths and DALYs avoided due to alcohol consumption (i.e., negative numbers indicate there would be an increase in the number of deaths and/or DALYs lost under the counterfactual scenario of everyone being a lifetime abstainer)

** Includes ICD-10 code: K70 (alcoholic liver disease), and ICD-10 code: K74 (fibrosis and cirrhosis of the liver)

Table S12. Alcohol-attributable burden of disease in 2019, by cause for females

Cause	Alcohol-attributable burden*		Age-adjusted alcohol-attributable burden per 100 000 people*		Population-attributable fraction (%)*	
	Deaths (1000s)	DALYs lost (100 000s)	Deaths	DALYs lost	Deaths	DALYs lost
All Causes	606·0 (466·3, 799·3)	231·7 (206·3, 284·1)	13·6 (11·0, 17·6)	578·2 (516·6, 708·4)	2·4 (1·8, 3·2)	2·0 (1·8, 2·4)
Communicable, maternal, perinatal and nutritional conditions	38·9 (19·6, 74·8)	16·2 (6·6, 35·6)	0·9 (0·4, 1·9)	42·3 (16·4, 94·7)	0·8 (0·4, 1·6)	0·5 (0·2, 1·1)
Tuberculosis	21·4 (4·2, 56·2)	11·7 (2·3, 31·2)	0·6 (0·1, 1·5)	31·4 (6·0, 83·8)	4·6 (0·9, 12·2)	4·4 (0·8, 11·6)
STDs excluding HIV	0·0 (0·0, 0·1)	0·1 (0·0, 0·1)	0·0 (0·0, 0·0)	0·2 (0·1, 0·3)	0·1 (0·1, 0·2)	0·3 (0·2, 0·4)
HIV/AIDS	2·0 (1·2, 3·2)	1·2 (0·7, 2·0)	0·1 (0·0, 0·1)	3·3 (1·9, 5·3)	0·7 (0·4, 1·1)	0·7 (0·4, 1·1)
Lower respiratory infections	15·5 (7·1, 25·5)	3·2 (1·6, 5·1)	0·3 (0·1, 0·5)	7·5 (3·9, 11·8)	1·3 (0·6, 2·1)	0·7 (0·3, 1·1)
Noncommunicable diseases	447·7 (308·2, 621·8)	132·4 (110·7, 162·7)	9·8 (7·1, 13·1)	322·0 (276·0, 389·7)	2·4 (1·6, 3·3)	1·7 (1·4, 2·1)
Malignant neoplasms	76·8 (61·8, 98·0)	21·0 (17·1, 26·2)	1·7 (1·4, 2·2)	49·9 (40·8, 62·3)	1·9 (1·5, 2·4)	1·9 (1·6, 2·4)
Lip and oral cavity	6·1 (4·0, 9·6)	1·7 (1·1, 2·6)	0·1 (0·1, 0·2)	4·0 (2·7, 6·2)	9·4 (6·2, 14·7)	9·5 (6·3, 14·5)
Other pharynx	2·9 (1·9, 4·6)	0·9 (0·6, 1·3)	0·1 (0·0, 0·1)	2·1 (1·3, 3·2)	10·7 (7·0, 16·7)	11·1 (7·2, 17·0)
Oesophagus cancer	6·2 (3·7, 10·0)	1·5 (0·9, 2·3)	0·1 (0·1, 0·2)	3·5 (2·2, 5·4)	5·1 (3·0, 8·1)	5·3 (3·3, 8·2)
Colon and rectum cancers	14·1 (6·1, 23·8)	3·4 (1·7, 5·5)	0·3 (0·1, 0·5)	7·8 (3·9, 12·6)	3·4 (1·5, 5·8)	3·7 (1·8, 5·9)
Liver cancer	17·4 (7·7, 31·5)	3·9 (1·8, 7·1)	0·4 (0·2, 0·7)	9·1 (4·1, 16·4)	9·7 (4·3, 17·5)	8·9 (4·0, 16·1)
Breast cancer	27·6 (20·5, 35·5)	8·8 (6·5, 11·3)	0·6 (0·5, 0·8)	21·3 (15·8, 27·4)	4·4 (3·2, 5·6)	4·5 (3·3, 5·8)
Cervix uteri cancer	1·4 (0·8, 2·4)	0·5 (0·3, 0·9)	0·0 (0·0, 0·1)	1·4 (0·8, 2·4)	0·5 (0·3, 0·8)	0·5 (0·3, 0·9)
Larynx cancer	1·0 (0·6, 1·8)	0·3 (0·2, 0·5)	0·0 (0·0, 0·0)	0·7 (0·4, 1·1)	6·2 (3·5, 10·6)	6·5 (3·8, 10·8)
Diabetes mellitus	-22·9 (-31·5, -12·3)	13·9 (18·4, 8·2)	0·5 (0·7, 0·3)	32·6 (43·1, 19·4)	3·0 (4·1, 1·6)	4·1 (5·4, 2·4)
Alcohol use disorders	22·6 (22·6, 22·6)	39·9 (39·9, 39·9)	0·5 (0·5, 0·5)	103·4 (103·4, 103·4)	100·0 (100·0, 100·0)	100·0 (100·0, 100·0)
Epilepsy	1·9 (1·4, 2·5)	2·1 (1·6, 2·8)	0·0 (0·0, 0·1)	5·4 (4·0, 7·2)	3·8 (2·8, 5·0)	3·6 (2·7, 4·7)
Cardiovascular diseases	230·8 (94·2, 393·3)	40·2 (19·8, 66·5)	4·7 (2·1, 7·8)	89·9 (46·4, 147·7)	2·7 (1·1, 4·6)	2·4 (1·2, 4·0)
Hypertensive heart disease	8·9 (4·1, 14·5)	1·6 (0·8, 2·6)	0·2 (0·1, 0·3)	3·5 (1·7, 5·6)	1·4 (0·6, 2·3)	1·4 (0·7, 2·3)
Ischaemic heart disease	180·7 (54·3, 300·7)	27·1 (9·9, 44·9)	3·5 (1·2, 5·8)	57·6 (21·3, 96·5)	4·5 (1·3, 7·4)	3·8 (1·4, 6·4)
Ischaemic stroke	41·8 (115·0, 49·8)	9·9 (21·0, 5·2)	0·8 (2·1, 0·9)	21·6 (44·1, 8·9)	2·6 (7·2, 3·1)	3·3 (7·0, 1·7)
Haemorrhagic stroke	78·0 (41·2, 123·7)	19·8 (10·9, 31·2)	1·7 (0·9, 2·7)	46·4 (25·6, 73·1)	5·4 (2·9, 8·6)	5·8 (3·2, 9·1)
Alcoholic cardiomyopathy	5·0 (5·0, 5·0)	1·6 (1·6, 1·6)	0·1 (0·1, 0·1)	4·0 (4·0, 4·0)	100·0 (100·0, 100·0)	100·0 (100·0, 100·0)
Digestive diseases	138·5 (110·6, 179·0)	43·1 (35·1, 54·4)	3·2 (2·6, 4·1)	105·9 (86·1, 133·2)	14·0 (11·1, 18·0)	12·6 (10·3, 15·9)
Cirrhosis of the liver**	135·3 (106·8, 174·6)	42·1 (33·9, 53·0)	3·2 (2·5, 4·1)	103·6 (83·6, 130·0)	30·2 (23·9, 39·0)	31·3 (25·2, 39·4)
Pancreatitis	3·2 (1·1, 6·2)	0·9 (0·3, 1·8)	0·1 (0·0, 0·1)	2·3 (0·8, 4·4)	7·9 (2·6, 15·2)	8·5 (3·1, 16·4)
Injuries	119·4 (97·2, 164·7)	83·1 (67·8, 111·9)	2·9 (2·4, 4·0)	213·9 (173·8, 287·7)	8·5 (6·9, 11·7)	9·8 (8·0, 13·2)
Unintentional injuries	93·3 (75·4, 132·4)	68·6 (55·5, 94·8)	2·3 (1·8, 3·2)	175·5 (142·1, 242·3)	8·8 (7·1, 12·4)	10·5 (8·5, 14·5)
Road injury	57·6 (43·1, 87·1)	40·9 (31·0, 60·9)	1·5 (1·1, 2·2)	106·7 (80·4, 158·9)	17·9 (13·4, 27·0)	19·2 (14·5, 28·5)
Poisonings	1·8 (1·2, 2·9)	1·0 (0·6, 1·5)	0·0 (0·0, 0·1)	2·5 (1·6, 4·0)	5·6 (4·0, 9·3)	5·1 (3·4, 8·2)
Falls	13·0 (7·5, 23·7)	12·4 (7·5, 20·1)	0·3 (0·2, 0·5)	29·7 (18·1, 47·4)	4·2 (2·4, 7·7)	7·5 (4·6, 12·1)
Fire, heat and hot substances	3·0 (2·0, 4·6)	2·5 (1·5, 4·0)	0·1 (0·0, 0·1)	6·5 (3·9, 10·4)	5·5 (3·7, 8·3)	6·1 (3·7, 9·7)
Drowning	3·1 (1·9, 5·5)	1·3 (0·8, 2·2)	0·1 (0·0, 0·1)	3·4 (2·0, 5·7)	4·2 (2·5, 7·5)	3·1 (1·8, 5·3)
Exposure to mechanical forces	1·3 (0·8, 2·3)	2·7 (1·7, 4·2)	0·0 (0·0, 0·1)	7·0 (4·3, 10·7)	4·1 (2·4, 7·1)	7·6 (4·7, 11·7)
Other unintentional injuries	13·5 (8·0, 21·6)	7·8 (4·7, 12·1)	0·3 (0·2, 0·5)	19·7 (11·9, 30·7)	5·6 (3·3, 9·0)	5·9 (3·6, 9·2)
Intentional injuries	26·1 (11·6, 46·1)	14·4 (5·9, 25·9)	0·7 (0·3, 1·2)	38·4 (15·3, 69·0)	7·8 (3·5, 13·7)	7·4 (3·0, 13·3)
Self-harm	18·4 (8·0, 32·6)	8·9 (3·5, 15·9)	0·5 (0·2, 0·8)	23·5 (9·2, 42·2)	8·5 (3·7, 15·1)	8·9 (3·5, 15·9)
Interpersonal violence	7·7 (3·6, 13·1)	5·6 (2·3, 9·9)	0·2 (0·1, 0·3)	14·8 (6·0, 26·5)	8·3 (3·8, 14·1)	7·9 (3·3, 14·0)

DALYs: Disability-adjusted life years

* Negative values represent deaths and DALYs avoided due to alcohol consumption (i.e., negative numbers indicate there would be an increase in the number of deaths and/or DALYs lost under the counterfactual scenario of everyone being a lifetime abstainer)

** Includes ICD-10 code: K70 (alcoholic liver disease), and ICD-10 code: K74 (fibrosis and cirrhosis of the liver)

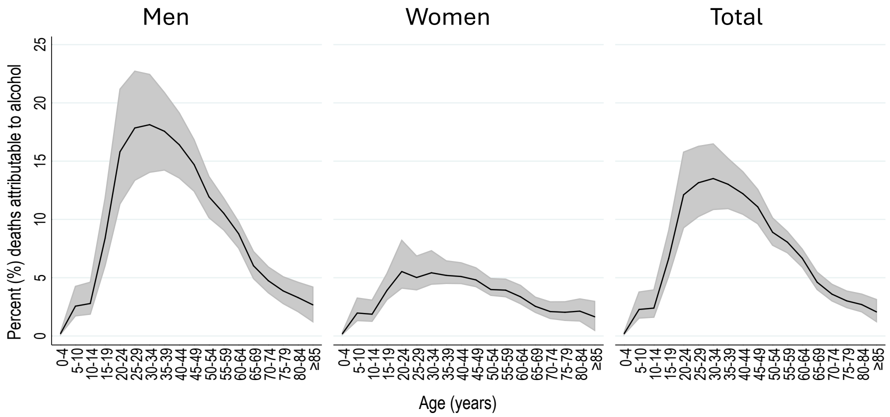

Figure S74. Percent of all deaths attributable to alcohol consumption by age

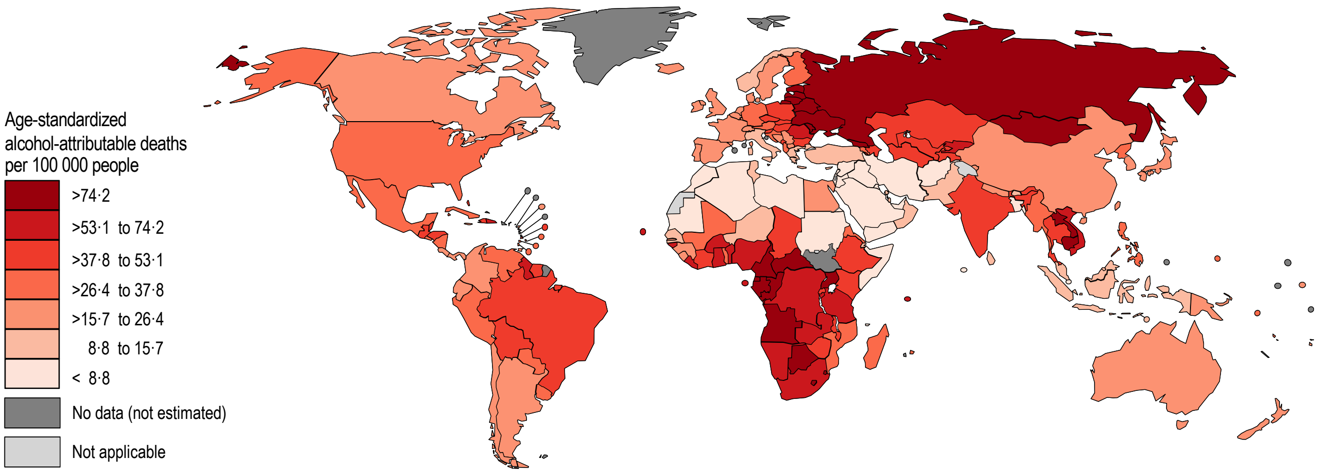

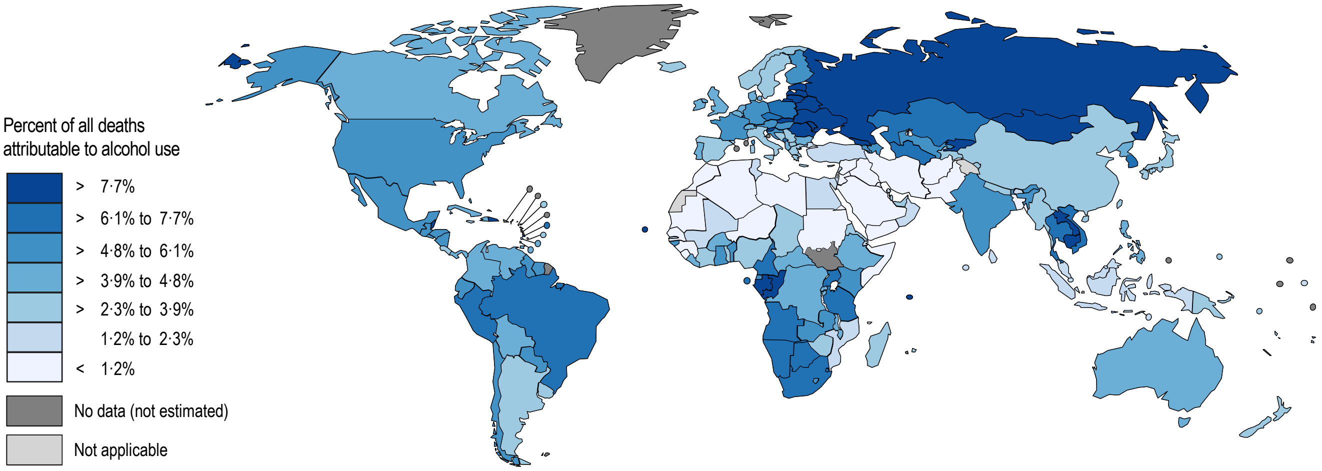

Figure S75. Alcohol-attributable deaths per 100 000 people, and the percent of all deaths attributable to alcohol consumption by country in 2019

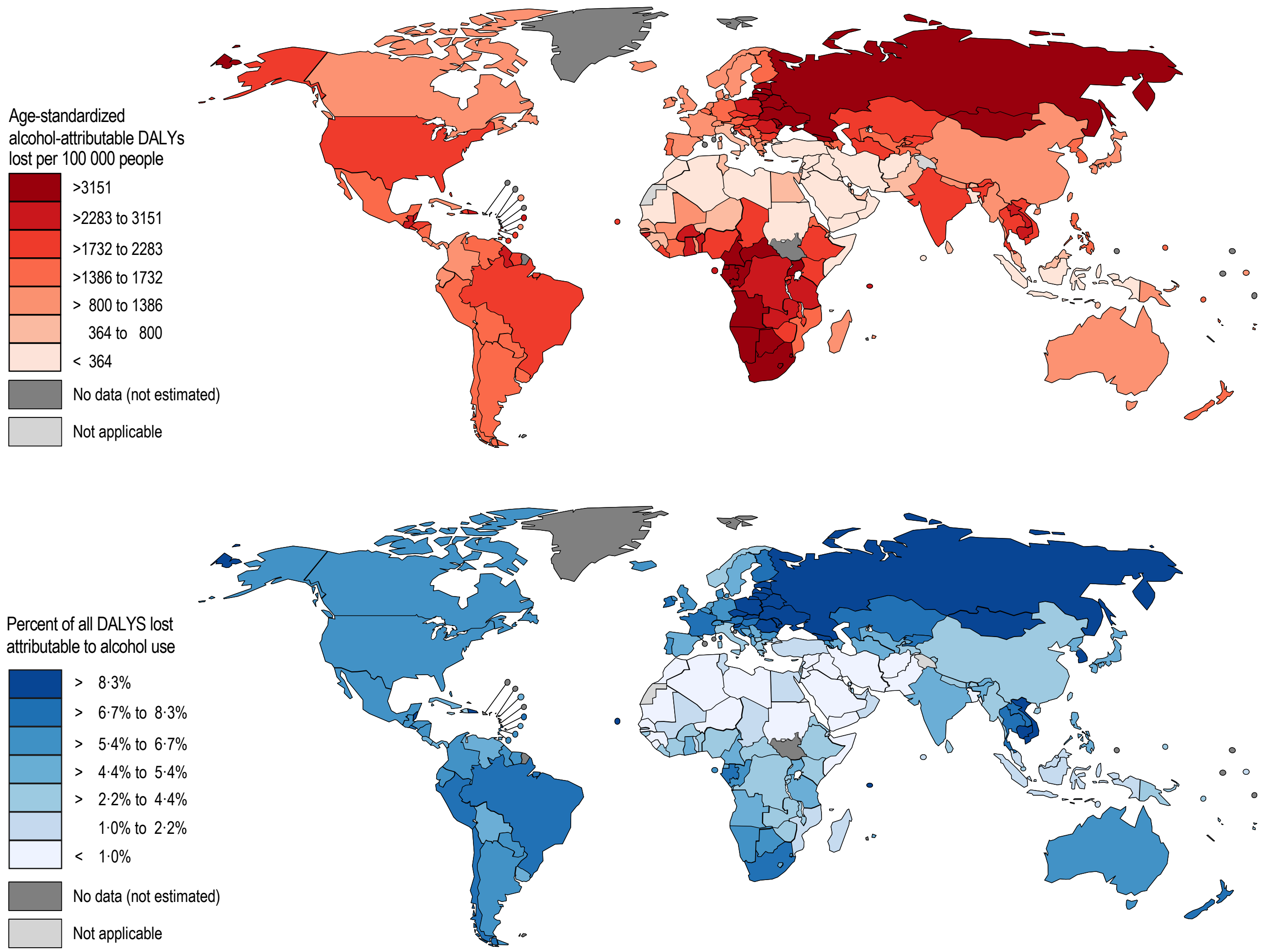

Figure S76. Alcohol-attributable years of life lost per 100 000 people, and the percent of all years of life lost attributable to alcohol consumption by country in 2019

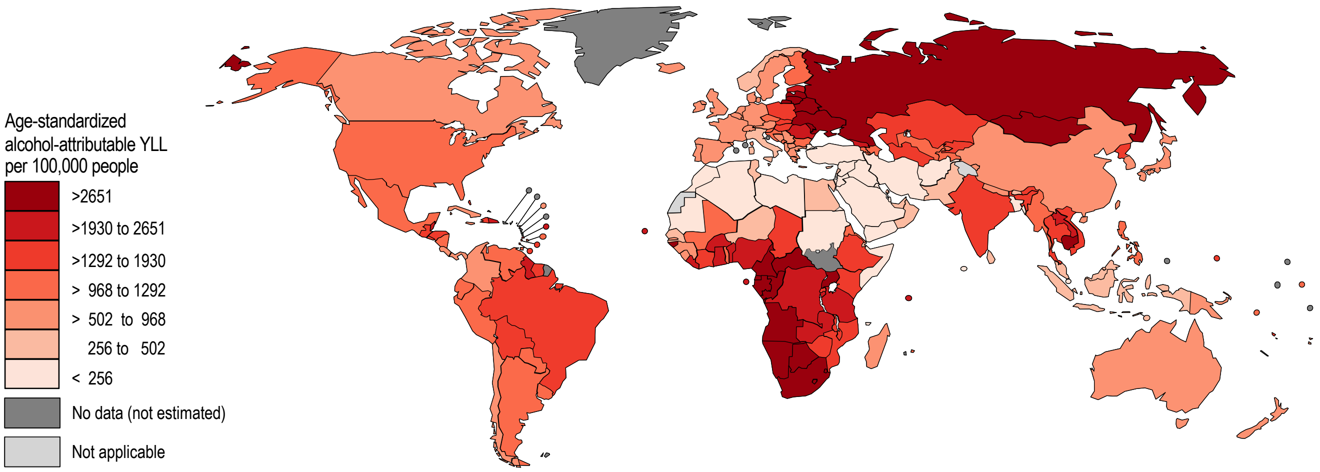

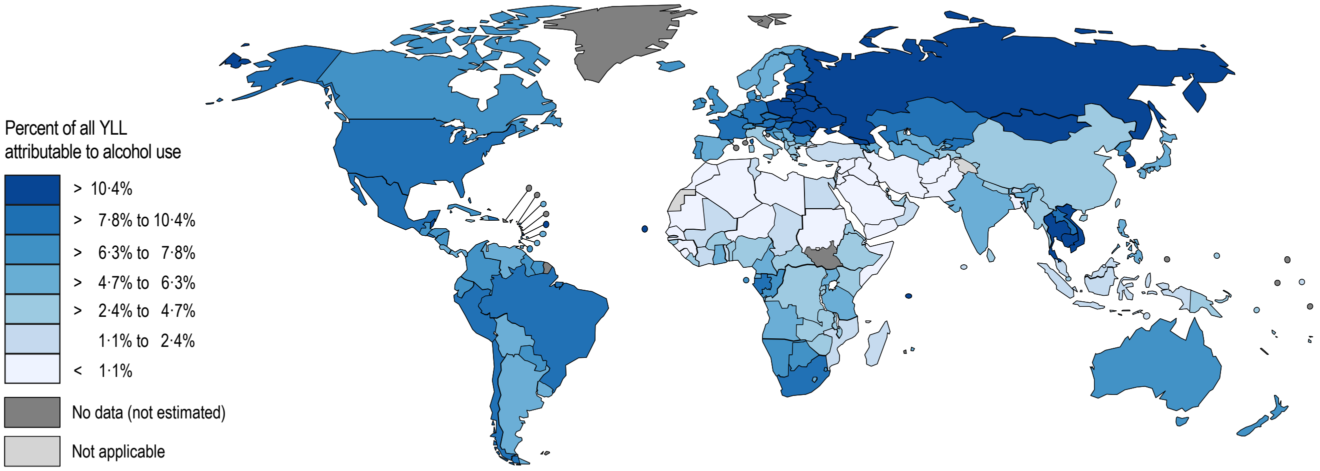

Figure S77. Alcohol-attributable years lived with disability per 100 000 people, and the percent of all years lived with disability attributable to alcohol consumption by country in 2019

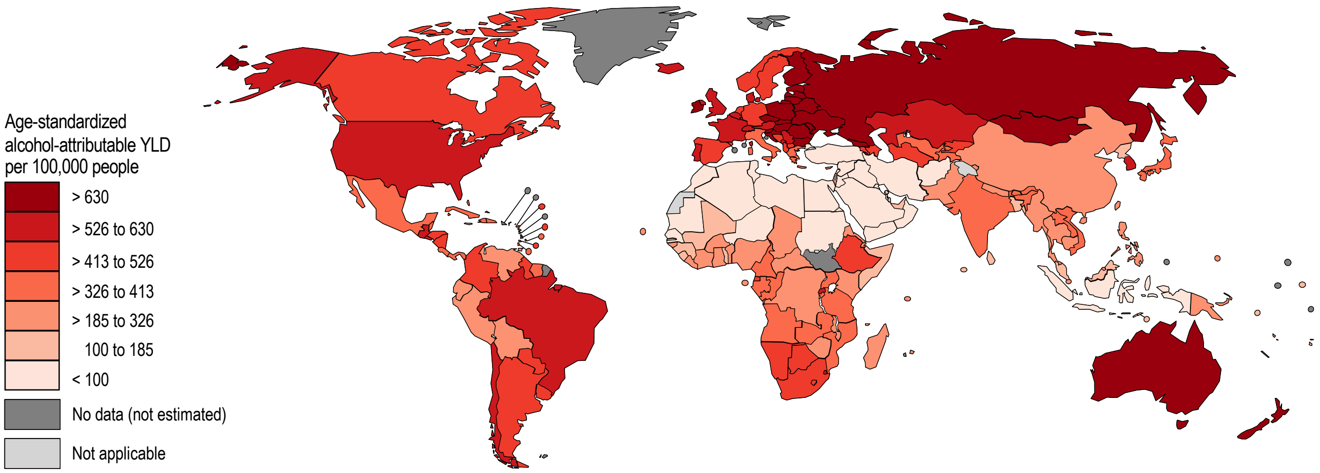

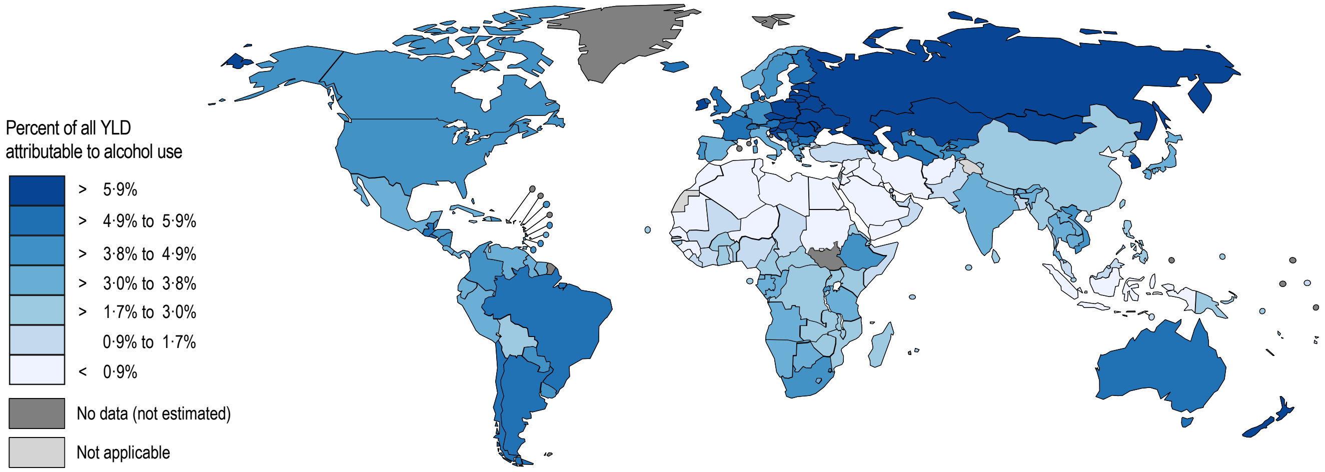

Figure S78. Alcohol-attributable disability adjusted years of life lost per 100 000 people, and the percent of all disability adjusted years of life lost attributable to alcohol consumption by country in 2019

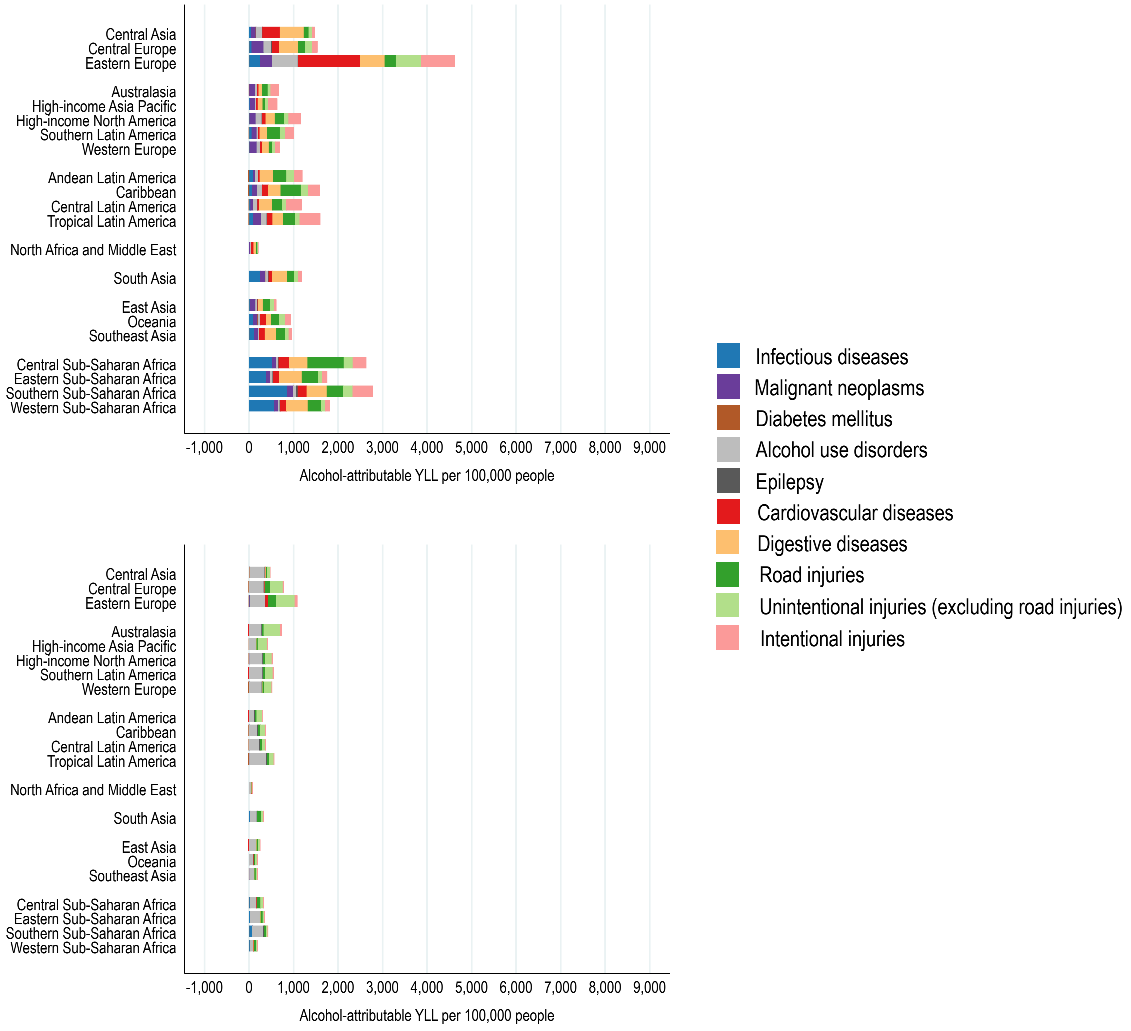

Figure S79. Alcohol-attributable years of life lost (YLL) and years with disability (YLD) per 100 000 people in 2019 by region and cause

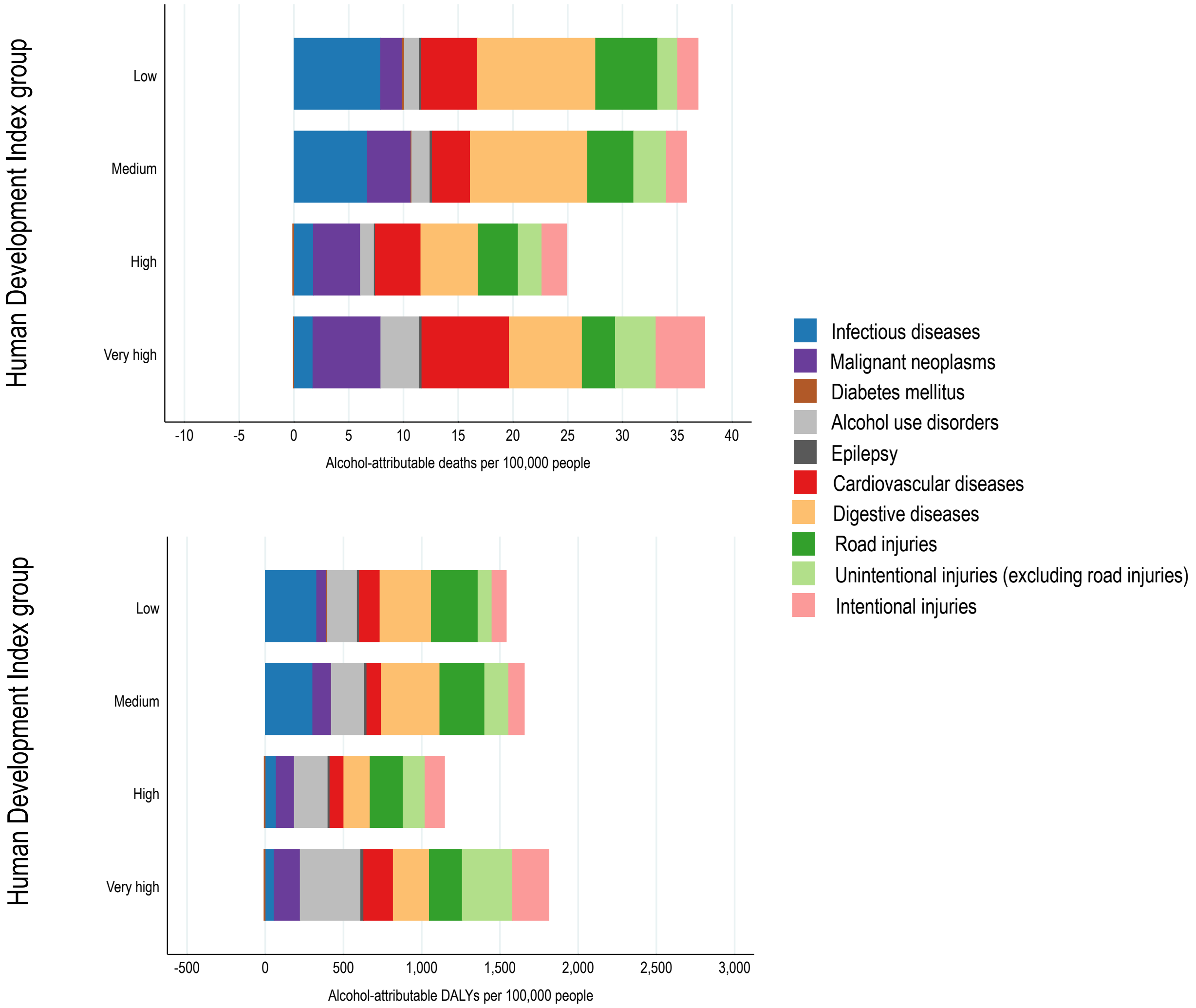

Figure S80. Alcohol-attributable deaths and disability adjusted years of life lost (DALYs) per 100 000 people in 2019 by human development index group and cause

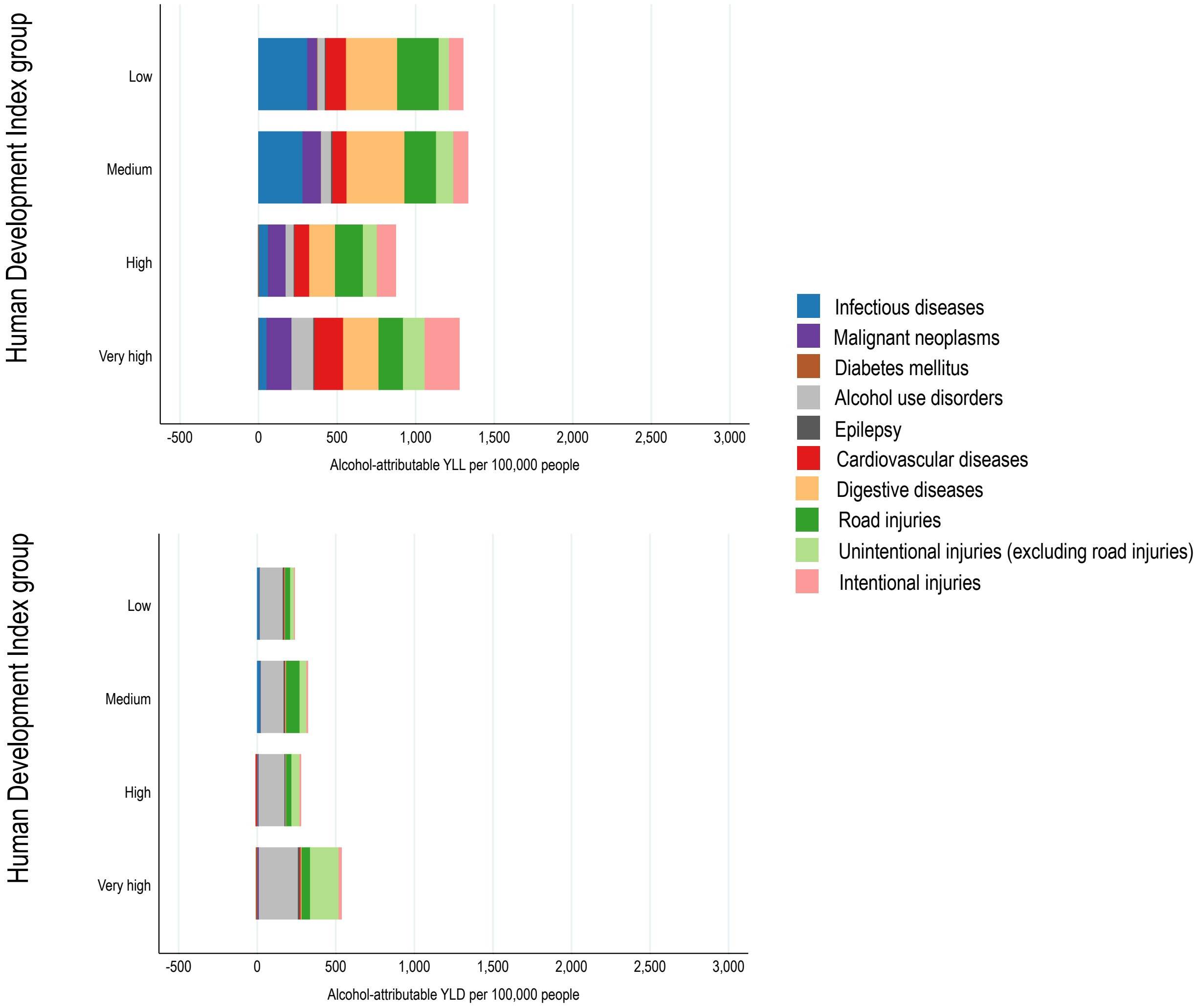

Figure S81. Alcohol-attributable years of life lost (YLL) and years with disability (YLD) per 100 000 people in 2019 by human development index group and cause

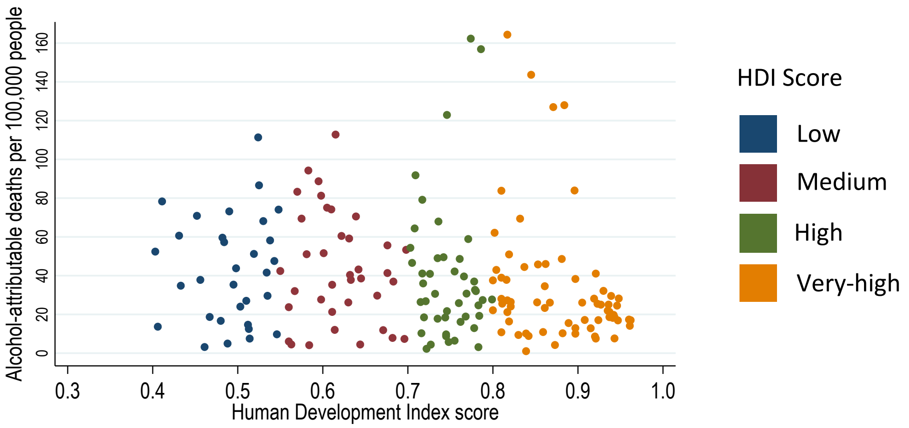

Figure S82. Alcohol-attributable deaths per 100 000 people in 2019 by human development index score

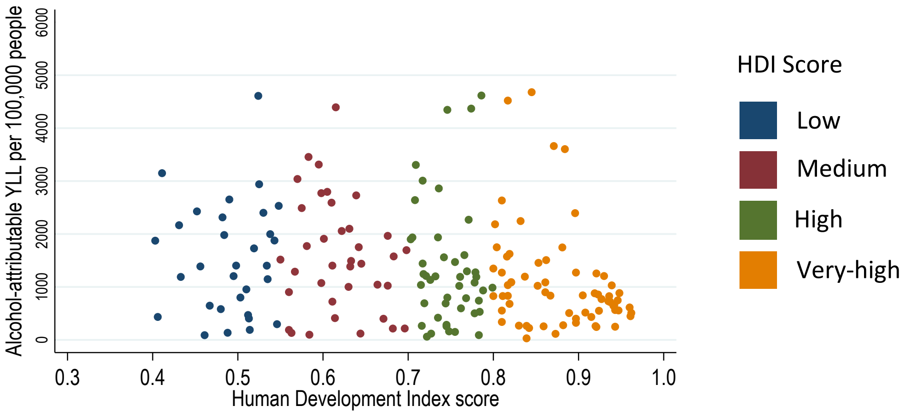

Figure S83. Alcohol-attributable years of life lost (YLL) per 100 000 people in 2019 by human development index score

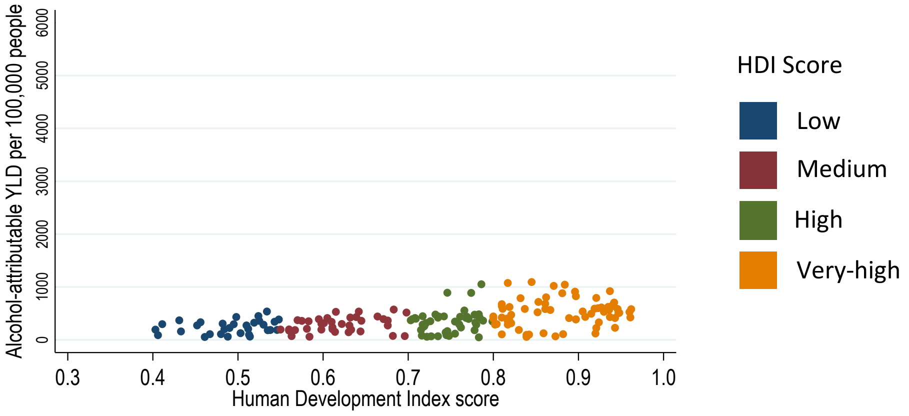

Figure S84. Alcohol-attributable years with disability (YLD) per 100 000 people in 2019 by human development index score

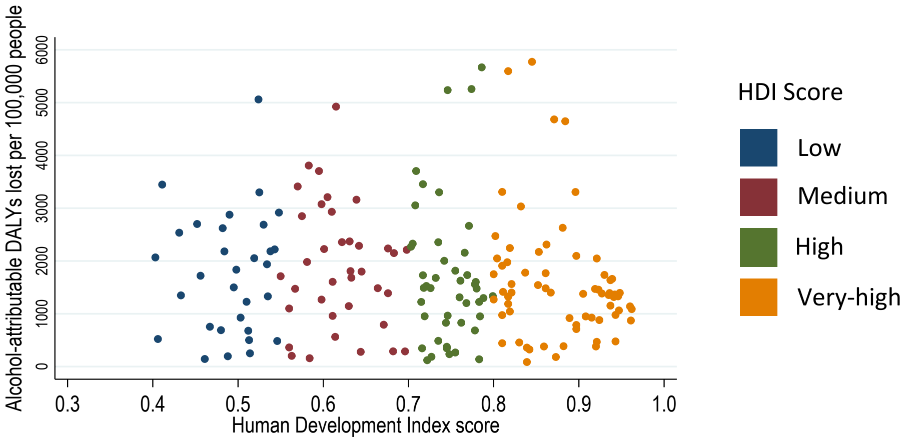

Figure S85. Alcohol-attributable disability adjusted years of life lost (DALYs) per 100 000 people in 2019 by human development index score

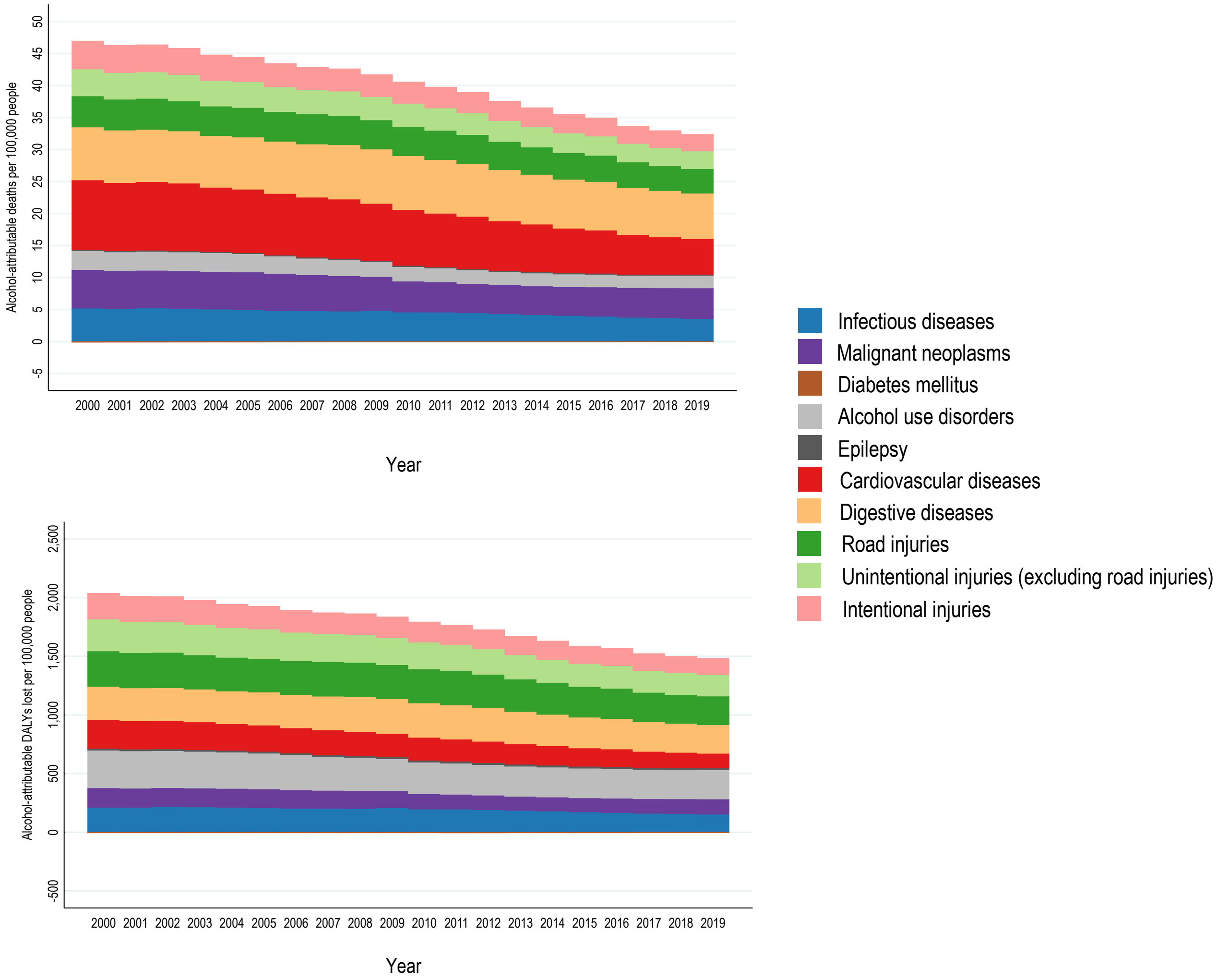

Figure S86. Alcohol-attributable deaths and disability adjusted years of life lost (DALYs) per 100 000 people by year and cause

Table S13. Burden of disease in the present study as compared to the IHME global burden of disease study

Outcome	Present study	IHME global burden of disease study	Difference (present study versus GBD study)
All causes	2624·4	2624·4	47.3%
Infectious diseases	284·1	136·8	107.6%
Noncommunicable diseases			
Malignant neoplasms	400·9	331·4	21.0%
Diabetes mellitus	-5·2	27·1	-
Alcohol use disorders	156·5	159·2	-1.7%
Epilepsy	13·2	11·9	10.6%
Cardiovascular diseases	473·6	375·3	26.2%
Digestive diseases	578·0	551·6	4.8%
Injuries	723·5	187·9	285.0%

References

1. Gmel G, Rehm J. Measuring alcohol consumption. *Contemporary Drug Problems* 2004; **31**: 467.
2. Stockwell T, Zhao J, Sherk A, Rehm J, Shield K, Naimi T. Underestimation of alcohol consumption in cohort studies and implications for alcohol's contribution to the global burden of disease. *Addiction* 2018; **113**(12): 2245-9.
3. King ES, Heymann H. The effect of reduced alcohol on the sensory profiles and consumer preferences of white wine. *Journal of Sensory Studies* 2014; **29**(1): 33-42.
4. Rehm J. Measuring alcohol consumption: how about adopting usual epidemiological standards? *Addiction* 1998; **93**(7): 970.
5. Feunekes GI, van't Veer P, van Staveren WA, Kok FJ. Alcohol intake assessment: the sober facts. *American journal of epidemiology* 1999; **150**(1): 105-12.
6. Shield KD, Rehm J. Difficulties with telephone-based surveys on alcohol consumption in high-income countries: the Canadian example. *International Journal of Methods in Psychiatric Research* 2012; **21**(1): 17-28.
7. Rehm J, Kehoe T, Gmel G, Stinson F, Grant B, Gmel G. Statistical modeling of volume of alcohol exposure for epidemiological studies of population health: the US example. *Population Health Metrics* 2010; **8**(1): 1-12.
8. Kehoe T, Gmel G, Shield KD, Gmel G, Rehm J. Determining the best population-level alcohol consumption model and its impact on estimates of alcohol-attributable harms. *Population Health Metrics* 2012; **10**(1): 6.
9. Levin ML. The occurrence of lung cancer in man. *Acta - Unio Internationalis Contra Cancrum* 1953; **9**(3): 531-41.
10. Guiraud V, Amor MB, Mas J-L, Touze E. Triggers of ischemic stroke: a systematic review. *Stroke* 2010; **41**(11): 2669-77.
11. Leong DP, Smyth A, Teo KK, et al. Patterns of alcohol consumption and myocardial infarction risk: observations from 52 countries in the INTERHEART case–control study. *Circulation* 2014; **130**(5): 390-8.
12. Roerecke M, Rehm J. Irregular heavy drinking occasions and risk of ischemic heart disease: a systematic review and meta-analysis. *American journal of epidemiology* 2010; **171**(6): 633-44.
13. Roerecke M, Rehm J. Alcohol consumption, drinking patterns, and ischemic heart disease: a narrative review of meta-analyses and a systematic review and meta-analysis of the impact of heavy drinking occasions on risk for moderate drinkers. *BMC Med* 2014; **12**: 182.
14. Knott C, Bell S, Britton A. Alcohol consumption and the risk of type 2 diabetes: a systematic review and dose-response meta-analysis of more than 1.9 million individuals from 38 obersvational studies. *Diabetes Care* 2015; **38**(9): 1804-12.
15. Shield KD, Gmel G, Gmel G, et al. Life-time risk of mortality due to different levels of alcohol consumption in seven European countries: implications for low-risk drinking guidelines. *Addiction* 2017; **112**(9): 1535-44.
16. Ezzati M, Lopez A, Rodgers A, Murray CJL. Comparative quantification of health risks. Global and regional burden of disease attributable to selected major risk factors. Geneva, Switzerland: World Health Organization; 2004.
17. Grundy A, Poirier AE, Khandwala F, McFadden A, Friedernreich CM, Brenner DR. Cancer inicidence attributable to alcohol consumption in Alberta in 2012. *CMAJ Open* 2016; **4**(3): E507.

18. Gmel G, Shield KD, Kehoe-Chan TAK, Rehm J. The effects of capping the alcohol consumption distribution and relative risk functions on the estimated number of deaths attributable to alcohol consumption in the European Union in 2004. *BMC Medical Research Methodology* 2013; **13**(1): 24.
19. Imtiaz S, Shield KD, Roerecke M, Samokhvalov AV, Lönnroth K, Rehm J. Alcohol consumption as a risk factor for tuberculosis: meta-analyses and burden of disease. *Eur Respir J* 2017; **50**(1): 1700216.
20. Rehm J, Samokhvalov AV, Neuman MG, et al. The association between alcohol use, alcohol use disorders and tuberculosis (TB). A systematic review. *BMC Public Health* 2009; **9**(450).
21. Rehm J, Probst C, Shield KD, Shuper PA. Does alcohol use have a causal effect on HIV incidence and disease progression? A review of the literature and a modeling strategy for quantifying the effect. *Popul Health Metr* 2017; **15**(1): 4.
22. Rehm J, Probst C, Shield K, Shuper P. Does alcohol use have a causal effect on HIV incidence and disease progression? A review of the literature and a modeling strategy for quantifying the effect. *Popul Health Metr* 2017; **15**(4).
23. Scott-Sheldon LA, Carey KB, Cunningham K, Johnson BT, Carey MP, Team MR. Alcohol use predicts sexual decision-making: a systematic review and meta-analysis of the experimental literature. *AIDS Behav* 2016; **20**(1): 19-39.
24. Samokhvalov A, Irving H, Rehm J. Alcohol consumption as a risk factor for pneumonia: a systematic review and meta-analysis. *Epidemiol Infect* 2010; **138**(12): 1789-95.
25. Traphagen N, Tian Z, Allen-Gipson D. Chronic ethanol exposure: pathogenesis of pulmonary disease and dysfunction. *Biomolecules* 2015; **5**(4): 2840-53.
26. Simet SM, Sisson JH. Alcohol's effects on lung health and immunity. *Alcohol Res* 2015; **37**(2): 199.
27. Bagnardi V, Rota M, Botteri E, et al. Alcohol consumption and site-specific cancer risk: a comprehensive dose-response meta-analysis. *Br J Cancer* 2015; **112**(3): 580-93.
28. International Agency for Research on Cancer. IARC monographs on the evaluation of carcinogenic risks to humans: volume 100E - personal habits and indoor combustions. Lyon, France: International Agency for Research on Cancer, 2009.
29. International Agency for Research on Cancer. IARC monographs on the evaluation of carcinogenic risks to humans: volume 96 - alcohol consumption and ethyl carbamate. Lyon, France: International Agency for Research on Cancer, 2007.
30. Vieira A, Abar L, Chan D, et al. Foods and beverages and colorectal cancer risk: a systematic review and meta-analysis of cohort studies, an update of the evidence of the WCRF-AICR Continuous Update Project. *Annals of Oncology* 2017; **28**(8): 1788-802.
31. World Cancer Research Fund/American Institute for Cancer Research. Diet, nutrition, physical activity and cancer: a global perspective. Continuous Update Project expert report 2018. . London, United Kingdom, 2018.
32. Sun Q, Xie W, Wang Y, et al. Alcohol consumption by beverage type and risk of breast cancer: a dose-response meta-analysis of prospective cohort studies. *Alcohol and Alcoholism* 2020; **55**(3): 246-53.
33. Llamosas-Falcón L, Probst C B, C, Jiang H, et al. Alcohol consumption and diabetes mellitus: a systematic review and meta-analysis. In Preperation.

34. Knott C, Bell S, Britton A. Alcohol consumption and the risk of type 2 diabetes: a systematic review and dose-response meta-analysis of more than 1.9 million individuals from 38 observational studies. *Diabetes Care* 2015; **38**(9): 1804-12.
35. Rehm J, Baliunas D, Borges GL, et al. The relation between different dimensions of alcohol consumption and burden of disease: an overview. *Addiction* 2010; **105**(5): 817-43.
36. Samokhvalov AV, Irving H, Mohapatra S, Rehm J. Alcohol consumption, unprovoked seizures, and epilepsy: A systematic review and meta-analysis. *Epilepsia* 2010; **51**(7): 1177-84.
37. Bartolomei F, Suchet L, Barrie M, Gastaut J-L. Alcoholic epilepsy: a unified and dynamic classification. *Eur Neurol* 1997; **37**(1): 13-7.
38. Barclay G, Barbour J, Stewart S, Day C, Gilvarry E. Adverse physical effects of alcohol misuse. *Adv Psychiatr Treat* 2008; **14**(2): 139-51.
39. Leach JP, Mohanraj R, Borland W. Alcohol and drugs in epilepsy: pathophysiology, presentation, possibilities, and prevention. *Epilepsia* 2012; **53**: 48-57.
40. World Health Organization. Global status report on alcohol and health, 2018. Geneva, Switzerland: World Health Organization, 2018.
41. Puddey IB, Rakic V, Dimmitt S, Beilin L. Influence of pattern of drinking on cardiovascular disease and cardiovascular risk factors-a review. *Addiction* 1999; **94**(5): 649-63.
42. O'Keefe JH, Bhatti SK, Bajwa A, DiNicolantonio JJ, Lavie CJ. Alcohol and cardiovascular health: the dose makes the poison... or the remedy. *Mayo Clin Proc* 2014; **89**(3): 382-93.
43. Rehm J, Shield KD, Roerecke M, Gmel G. Modelling the impact of alcohol consumption on cardiovascular disease mortality for comparative risk assessments: an overview *BMC Public Health* 2016; **16**: 363.
44. Roerecke M, Rehm J. The cardioprotective association of average alcohol consumption and ischaemic heart disease: a systematic review and meta-analysis. *Addiction* 2012; **107**(7): 1246-60.
45. Roerecke M, Rehm J. Ischemic heart disease mortality and morbidity in former drinkers: a meta-analysis. *Am J Epidemiol* 2011; **173**(3): 245-58.
46. Roerecke M, Rehm J. Alcohol consumption, drinking patterns, and ischemic heart disease: a narrative review of meta-analyses and a systematic review and meta-analysis of the impact of heavy drinking occasions on risk for moderate drinkers. *BMC Med* 2014; **12**: 182.
47. Mukamal KJ, Rimm EB. Alcohol's effects on the risk for coronary heart disease. *Alcohol Res Health* 2001; **25**: 255-61.
48. Collins MA, Neafsey EJ, Mukamal KJ, et al. Alcohol in moderation, cardioprotection, and neuroprotection: epidemiological considerations and mechanistic studies. *Alcohol Clin Exp Res* 2009; **33**(2): 206-19.
49. Patra J, Taylor B, Irving H, et al. Alcohol consumption and the risk of morbidity and mortality from different stroke types - a systematic review and meta-analysis. *BMC Public Health* 2010; **10**(1): 258.
50. Puddey IB, Rakic V, Dimmitt SB, Beilin LJ. Influence of pattern of drinking on cardiovascular disease and cardiovascular risk factors - a review. *Addiction* 1999; **94**: 649-63.
51. Mazzaglia G, Britton R, Altmann DR, Chenet L. Exploring the relationship between alcohol consumption and non-fatal or fatal stroke: a systematic review. *Addiction* 2001; **96**: 1743-56.
52. Larsson SC, Wallin A, Wolk A, Markus HS. Differing association of alcohol consumption with different stroke types: a systematic review and meta-analysis. *BMC medicine* 2016; **14**(1): 1-11.

53. Roerecke M, Vafaei A, Hasan OSM, et al. Alcohol consumption and risk of liver cirrhosis: a systematic review and meta-analysis. *Am J Gastroenterol* 2019; **114**(10): 1574-86.
54. Gao B, Bataller R. Alcoholic liver disease: pathogenesis and new therapeutic targets. *Gastroenterology* 2011; **141**(5): 1572-85.
55. Samokhvalov AV, Rehm J, Roerecke M. Alcohol consumption as a risk factor for acute and chronic pancreatitis: a systematic review and a series of meta-analyses. *EBioMedicine* 2015; **2**(12): 1996-2002.
56. Braganza JM, Lee SH, McCloy RF, McMahon MJ. Chronic pancreatitis. *Lancet* 2011; **377**(9772): 1184-97.
57. Yadav D, Lowenfels AB. The epidemiology of pancreatitis and pancreatic cancer. *Gastroenterology* 2013; **144**(6): 1252-61.
58. Lankisch P, Apte M, Banks P. Acute pancreatitis. *Lancet* 2015; **386**(10008): 2058-.
59. Majumder S, Chari ST. Chronic pancreatitis. *Lancet* 2016; **387**(10031): 1957-66.
60. World Health Organization. Global status report on alcohol and health 2018. 2018. https://www.who.int/substance_abuse/publications/global_alcohol_report/en/ (accessed 11/14/2019.
61. World Health Organization. Alcohol and injuries: emergency department studies in an international perspective. Geneva, Switzerland: World Health Organization 2009.
62. Zaridze D, Brennan P, Boreham J, et al. Alcohol and cause-specific mortality in Russia: a retrospective case–control study of 48 557 adult deaths. *Lancet* 2009; **373**(9682): 2201-14.
63. Shield KD, Rehm J. Russia-specific relative risks and their effects on the estimated alcohol-attributable burden of disease. *BMC Public Health* 2015; **15**(482).
